# Expression-based analyses indicate a central role for hypoxia in driving tumor plasticity through microenvironment remodeling and chromosomal instability

**DOI:** 10.1038/s41540-018-0074-z

**Published:** 2018-10-24

**Authors:** Anqi Jing, Frederick S. Vizeacoumar, Sreejit Parameswaran, Bjorn Haave, Chelsea E. Cunningham, Yuliang Wu, Roland Arnold, Keith Bonham, Andrew Freywald, Jie Han, Franco J. Vizeacoumar

**Affiliations:** 1 Department of Electrical and Computer Engineering, University of Alberta, Edmonton, AB T6G2R3 Canada; 2 Department of Pathology and Laboratory Medicine, College of Medicine, University of Saskatchewan, Saskatoon, SK S7N 5E5 Canada; 3 Department of Biochemistry, Cancer Cluster, College of Medicine, University of Saskatchewan, Saskatoon, SK S7N 5E5 Canada; 4 Institute of Cancer and Genomic Sciences, University of Birmingham, Birmingham, United Kingdom; 5 Cancer Research, Saskatchewan Cancer Agency, Saskatoon, SK S7N 5E5 Canada

## Abstract

Can transcriptomic alterations drive the evolution of tumors? We asked if changes in gene expression found in all patients arise earlier in tumor development and can be relevant to tumor progression. Our analyses of non-mutated genes from the non-amplified regions of the genome of 158 triple-negative breast cancer (TNBC) cases identified 219 exclusively expression-altered (EEA) genes that may play important role in TNBC. Phylogenetic analyses of these genes predict a “punctuated burst” of multiple gene upregulation events occurring at early stages of tumor development, followed by minimal subsequent changes later in tumor progression. Remarkably, this punctuated burst of expressional changes is instigated by hypoxia-related molecular events, predominantly in two groups of genes that control chromosomal instability (CIN) and those that remodel tumor microenvironment (TME). We conclude that alterations in the transcriptome are not stochastic and that early-stage hypoxia induces CIN and TME remodeling to permit further tumor evolution.

## Tumorigenesis: Hypoxia affects CIN and TME genes simultaneously

Altered gene expression is universal in tumors, but does it impact tumor development and progression? A collaborative team lead by Franco Vizeacoumar and Jei Han from Canada’s University of Saskatchewan and University of Alberta respectively, analyzed expression of non-mutated and non-amplified genes in Triple-Negative Breast Cancer (TNBC) patients. This exclusively expression-altered genes, included those that regulate chromosomal instability (CIN) and remodeling of tumor microenvironment (TME). Further analyses predict hypoxia induced a “punctuated burst” of changes in expression patterns of genes involved in CIN and TME, driving early stages of tumor development. This work provides a novel strategy to co-inhibit CIN and TME genes to disrupt tumor development.

## Introduction

The Darwinian model of clonal selection, where a subset of genetic lesions drives tumor evolution and progression in a step-wise manner,^1–4^ is widely accepted as the mode of evolution of malignant cells under therapy or basal conditions.^5–7^ However, recent findings in prostate, pancreatic or triple-negative breast cancer (TNBC), challenge this paradigm and question if gradualism is indeed the single mode of evolution.^8–10^ It may be instead a punctuated burst of molecular alterations in the early stages of cancer, where changes in biological environment of growing tumors require massive adaptations in the molecular machinery of cancer cells.^11–13^ Usually, normal cells respond to stress by deploying repair or resistance tools to maintain their genetic integrity and assure survival.^14,15^ In contrast, cancer cells typically do not have intact repair tools, which lead to genetic instability. Chromosomal instability (CIN) is a form of genetic instability that causes changes in both the structure and number of chromosomes.^15–25^ For example, mutations in CIN genes like BRCA1/2 increase the number of deletions up to 50 bps, causing multiple defects within the genome.^26^ Progressive accumulation of CIN within a tumor allows development of cell populations with heterogeneous properties. Some of these cells will carry selective survival advantages and will be responsible for further tumor progression.^3^ Likewise, overexpression of APOBEC3, a member of the cytidine deaminase gene family, may generate frequent C > T base substitutions also leading to tumor heterogeneity and progression along the malignancy pathway.^27^ Understanding the sequence of molecular events essential for tumor evolution may not only benefit early detection of malignancies but may also allow the development of more effective treatment and even prevention strategies. While the role of accumulating genetic mutations in tumor progression has been extensively discussed, it is still not clear how alterations in gene expression patterns contribute to tumor evolution.

Changes to gene expression can be brought about by number of factors, including epigenetic modifications, translation regulation, and differences in mRNA, and protein stability.^28^ For example, increased activities of growth factor, chemokine and cytokine receptors can set off specific signaling cascades and subsequent changes in gene expression, without any direct involvement of genetic mutations. However, what are the most significant changes that occur within the transcriptome of cancer cells and how they may contribute to tumor evolution is not clear. Here, we use an aggressive malignancy, TNBC, as a model to explore the role of transcriptomic alterations during early stages that are caused not by genomic mutations, but exclusively by differential gene expression. We achieve this by focusing specifically on genes that are heavily upregulated in the non-amplified regions of the genome. We focused specifically on upregulated genes because direct inhibition of these molecules may provide viable cancer treatment/prevention options at early stages of tumor development. Remarkably, our analysis of RNA seq data in 158 TNBC cases revealed that there is indeed a punctuated burst of expressional changes in two major groups of genes controlled by hypoxia-related factors. These two groups included molecules that regulate CIN and remodel tumor microenvironment (TME). This not only reveals new potential targets for TNBC therapy, but also indicates a critical role for hypoxia in very early stages of tumor development.

## Results

### A multi-step process to identify differentially expressed genes in breast cancer

**Fig. 1:**
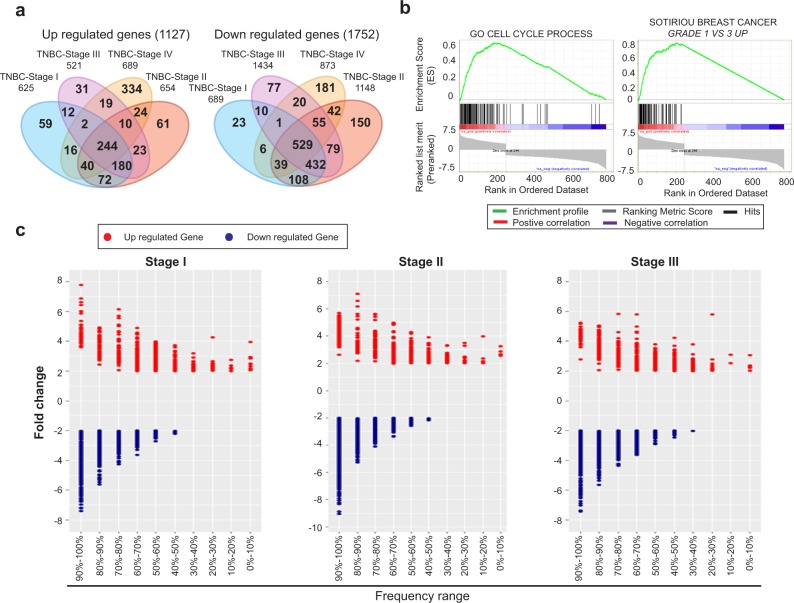
Identification of upregulated genes in TNBC. **a** Venn-diagram of differentially expressed genes in TNBC-stage-specific tumors. The number of up- and downregulated genes at each stage of tumor and at the intersection between different stages have been represented. **b** Gene set enrichment analysis for up/downregulated genes across all TNBC tumor stages. Gene Set Enrichment Analysis for 244 upregulated genes (left) and 529 downregulated genes (right) across four tumor stages along with previously identified, differentially upregulated genes from Sotiriou et al.^29^ **c** Frequency distribution of differential expression in TNBC-stage-specific tumors. Dot plot represents the fold-change and the frequency range of TNBC-stage-specific differentially expressed genes, where the red denotes upregulated gene and the blue denotes downregulated gene

To identify genes with aberrant expression patterns, we initially curated all the genes that are differentially regulated. We used the breast cancer-specific data from The Cancer Genome Atlas (TCGA) that represents the largest collection of patient samples with information on the mutation status, copy number aberrations (CNA), as well as gene expression patterns at different stages of tumor development. Gene expression in breast tumor samples was compared to the expression of the matching genes in normal samples using fold-change (FC) and false discovery rate (FDR) after Empirical Bayes moderated *t*-test with Benjamini-Hochberg correction. Genes were considered as upregulated genes if FDR ≤ 0.01 and FC ≤ 2. Downregulated genes were selected if FDR ≤ 0.01 and FC ≤ −2. Our initial analyses in overall breast cancer identified 586 genes that were upregulated and 1446 genes that were downregulated at multiple stages of cancer progression (Supplementary Fig. 1a). The overlap between all stages is presented in Supplementary Table 1a. We also ran a complementary analysis to identify differentially regulated genes in specifically in TNBC. We found 1127 genes to be upregulated and 1752 genes downregulated across multiple stages of TNBC (Fig. 1a). The overlap between all stages is shown in Supplementary Table 1b. The Gene Set Enrichment Analysis (GSEA) indicated that the upregulated genes in TNBC are enriched for molecules involved in cell-cycle regulation and chromatin organization (*p* < 0.001) (Fig. 1b). Results of our GSEA analysis of genes differentially upregulated in TNBC tumors correlated well with the previously reported, differentially regulated genes from an independent cohort (*p* < 0.001) (Fig. 1b),^29^ which provides an additional support for the relevance of our observations. While we found a higher abundance of downregulated genes, compared to the upregulated genes, no similar significant enrichment was observed within the pool of the downregulated genes. Similar results were obtained for overall breast cancer (Supplementary Fig. 1b). Taken together, these observations indicate that the application of our approach to the analysis of TCGA data allows identifying subsets of genes differentially regulated in TNBC tumors.

### Not all differentially expressed genes are equally deregulated across the population of breast cancer patients

While gene expression analysis to identify differentially regulated genes has been a common approach in cancer biology, we attempted to determine, how many of these genes are aberrantly expressed with high frequencies across the population of TNBC patients. We rationalized that common aberrations found in all patients should have arisen earlier in the development of the malignancy, compared to alterations that were found only in a subset of patients. Therefore, we have calculated a frequency of differential expression of each affected gene in TNBC tumors (Fig. 1c and Supplementary Fig. 1c). Throughout this analysis, we maintained a twofold change in the expression level as a minimum requirement for a gene to be considered differentially regulated. The frequency of changes in each differentially expressed gene is calculated as a percentage of patients in whom the gene is up- or downregulated. We found 254 genes were upregulated and 1197 genes were downregulated in almost 70% of the TNBC patients (Supplementary Table 2a, b). Similar results for overall breast cancer are presented Supplementary Table 3a, b. Unfortunately, there were only two patient samples that were available in TNBC-stage IV in TCGA dataset, which was not sufficient to minimize random effects. Therefore, we computed our analyses using the larger number of samples involved in the first three stages of TNBC.

**Fig. 2:**
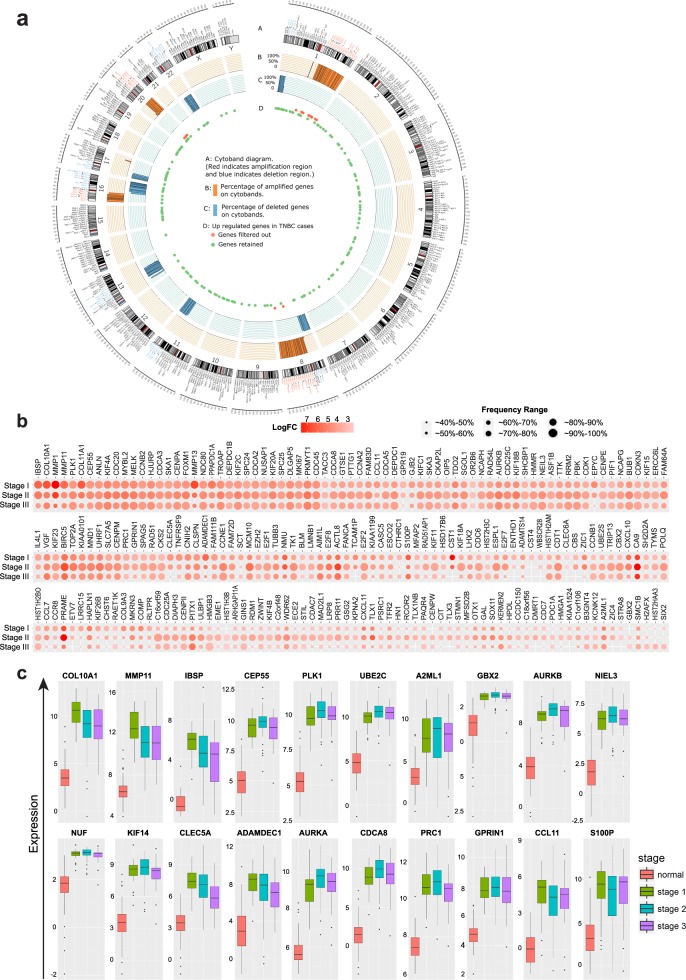
Elimination of amplified genes to identify 219 upregulated events. **a** Amplified chromosome cytobands and upregulated genes locus. Track A displays the cytoband diagram where the texts in red indicate identified amplified regions. Track B and C displays the frequency of genes showing amplification and deletion, respectively, in at least in 40% of patients in each cytoband. Genes in Fig. 1d were mapped to the Track D. **b** Fold-change and frequency distribution for genes showing upregulation in at least 70% of TNBC patients. Nodes in each column represent upregulated genes with their sizes indicating the frequency of samples and their colors representing the fold-change value in the specific tumor stage. **c** Box plots of Cluster 1 gene expression at various stages of TNBC tumor. The *y*-axis represents log2-transformed gene expression and *x*-axis denotes TNBC stages

Changes in gene expression may not only arise from aberrant expression from an endogenous promoter, but also from accompanying chromosomal amplifications, deletions and other types of mutations. To account for this, we isolated the differentially regulated genes exclusively from the non-amplified/deleted regions of the genome. We identified 77 amplified chromosome regions from the TCGA dataset based on CNA, including several previously reported regions in 1q, 8q, 16p, and 20q (Supplementary Table 4),^30^ as presented in the circos plot for TNBC (Fig. 2a) or overall breast cancer (Supplementary Fig. 2a). We further evaluated the concordance of amplification and gene expression by fold-change with FDR and Pearson’s correlation. We considered genes likely to be driven by CNA if their Pearson’s correlation coefficient between expression and CNA was greater than 0.3, or they show significant differential CNA-associated expression change (Supplementary Fig. 2b, c and Supplementary Table 5a, b). Subsequently, we filtered out from our analysis 20 genes from TNBC patients that were in amplified regions or had strong correlations with chromosome amplification.

We also used somatic mutational analyses of 560 breast cancer whole-genome sequencing database available at COSMIC to eliminate any gene that might be differentially expressed because of a mutation.^31^ By also excluding 13 genes whose loci information was ambiguous, we finally identified 219 exclusively expression-altered (EEA) genes that elevated their expression in TNBC (Fig. 2b) and, therefore, may represent good therapeutic targets. Interestingly, we observed multiple distinct patterns of upregulation with varying frequencies across different cancer stages (Fig. 2b). For example, some genes were constitutively upregulated across all stages (PLK1, UBE2C, or KIF4A). Similarly, certain genes were upregulated mostly at later stages (CCNE1, HMGB3, or NUF2). In contrast to this category, some genes were upregulated selectively at early stages but were gradually downregulated through the later stages (MMP1, MMP11, or MMP13). Among the 219 upregulation events, majority of changes occurred in chromosome 1 and 17 (Supplementary Fig. 2d). Surprisingly, although the expression of some initially upregulated genes gradually decreased, we did not observe any instance where their expression returned to normal levels (Fig. 2c). Importantly, while we find that not all upregulated genes are overexpressed in all breast tumors across all cancer stages, our analysis has generated an explicit set of genes that are overexpressed in over 70% of patients at all stages of both all breast cancer and TNBC tumors (Supplementary Table 6a, b).

### Lineage analysis of upregulation profiles reveals a punctuated pattern of evolution in early TNBC tumors

**Fig. 3:**
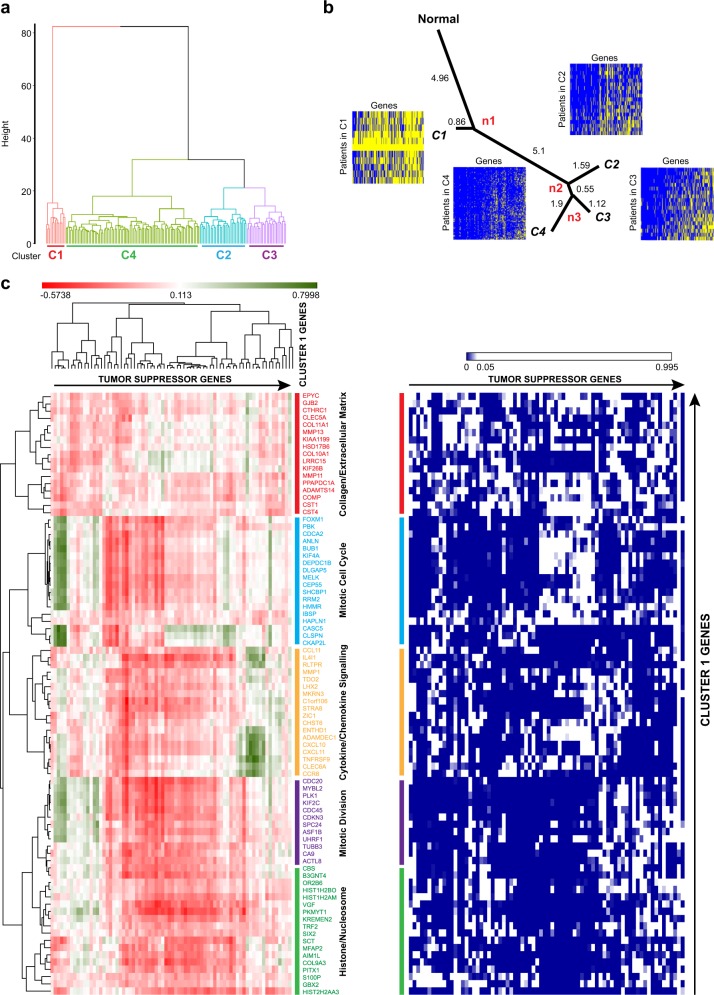
Identification of 83 upregulation events that occur in early stages. **a** Hierarchical clustering of 219 EEA genes in TNBC patients. Different colors show TNBC patients clustered into four clusters represented as Red for Cluster 1 (C1), Purple for Cluster 2 (C2), Blue for Cluster 3 (C3), and Green for Cluster 4 (C4). **b** The progression of gene upregulations in different TNBC clusters as shown by the phylogenetic tree. The figure shows the lineage of progression of gene upregulations from the normal to distinct subpopulations. Heat maps with genes in columns and TNBC samples in rows display the upregulation status (yellow: no upregulation; blue: upregulation) for different TNBC clusters. **c** Correlation clustergram of cluster 1 genes compared to known tumor suppressors. Red indicates negative correlation and green indicates positive correlation. The panel on the right represents, the significance of the correlation as a heat map. Blue indicates significance (<0.05) and white indicates lack of significance (>0.05)

Previous studies have used somatic mutations and CNA to understand tumor evolution.^8,10,11,13^ However, it is not clear, how alterations in gene expression may affect tumor progression. To further address this, we performed a progression-based analysis on the expression profiles of the newly identified EEA genes to describe how they may influence TNBC progression. First, based on the profile of upregulation status of EEA genes, we partitioned TNBC samples into groups so that the samples within a group have more similar upregulation profiles than other samples in different groups. Hierarchical clustering was applied to group tumor samples into clusters^32^. The cluster structure was graphically represented in Fig. 3a, which revealed that the most distinguishable cluster C1 is diverged at the highest overhang with the highest dissimilarities from the remaining samples. In addition, several distinguishable branches C2, C3, and C4 were also clustered. After identifying distinct tumor clusters, we constructed their distance tree to gain insights into the progression path in the context of accumulation of aberrant expression, analogous to a phylogenetic analysis. The construction of the distance tree was based on the neighbor-joining algorithm^33^ to display the lineage between the four clusters. Assuming that the tumor is derived from a single or a group of homogenous normal cells and the complexity of upregulation in a tumor increases with time, the history of its progression can be partially inferred by comparing homogeneous groups. We used the vector with 219 elements of all zeros representing no upregulations of EEA genes in normal status as the root vertex, and constructed a tree-like structure by neighbor-joining to evaluate the extent of the accumulation of upregulatory events for each cluster.

The phylogenetic lineage showed that cluster C1 has the shortest distance from normal samples, which suggests that tumors in C1 may be recorded at the early tumor stage and the upregulated genes in C1 may be of importance to tumor initiation and early progression (Fig. 3b). The lineage shows a large distance from normal cell to C1, indicating a large number of upregulatory events are required for successful tumor progression through very early stages. C2, C3, and C4 clusters diverged for relatively small distances from the common ancestor n2, which suggests less dissimilarity from C2 to C3 and C4, indicating that minimal changes in gene expression were required at later stages. By measuring the Cosine similarity between mean upregulation profile and subset vector (See Methods for details), we found that a burst of 83 upregulation events occurred earliest in C1 suggesting that the 83 EEA genes that may act as potential enabling factors within the early tumor evolution (Supplementary Table 7).

To confirm that the 83 upregulation events are relevant to breast cancer progression, we next inquired if these changes in gene expression correlate with the loss of expression of known tumor suppressors. Vogelstein and colleagues identified ~70 tumor suppressor genes that when inactivated by intragenic mutations can promote tumorigenesis.^34^ We found a strong negative correlation in the expression of the 83 EEA genes and the 74 tumor suppressors (Fig. 3c). In summary, our analysis revealed that a large number of EEA events appear at the earliest stage of tumor development with fewer subsequent events at later stages, reflecting an emerging pressure from rapidly changing biological environment within early progressing tumors.

### TME remodeling and CIN cooperatively drive TNBC

Since our phylogenetic analysis indicated that the 83 upregulated EEA genes are crucial early events in early tumorigenesis, we next explored the functionalities of these genes. Interestingly, we found a large subset of genes that are known to be involved in remodeling TME, including metalloproteinases (MMP1, MMP11, MMP13, ADAMDEC1, ADAMTS14), chemokine receptors and ligands (CXCL11, CXCL10, CCL11, CCR8), protease inhibitors (CST4, CST1), pH maintenance factors (CAIX), and different collagens (COL9A3, COL10A1). This emphasizes the critical role of extracellular matrix and TME remodeling at the early-stage of tumor progression. Similarly, we also identified several of the EEA genes including, FOXM1, PLK1, BUB1, KIF2C, CDCA2, CDC20, CDKN3, KNL1 to name a few, that are known for their role in CIN and tumor development.^35–43^ This may reflect a selective pressure for additional genetic alterations in early tumors that would allow their further evolution. In addition, cluster 1 included genes like DEPDC1B and HMMR that have known roles in both TME remodeling as well as CIN associated functions.^44–48^ Overall, our identification of cluster 1 genes indicates that a punctuated burst of expressional changes occurs simultaneously in both CIN and TME remodeling genes very early in tumor development. Supplementary Table 8a lists literature evidence for the role of cluster 1 genes in TME and CIN.

**Fig. 4:**
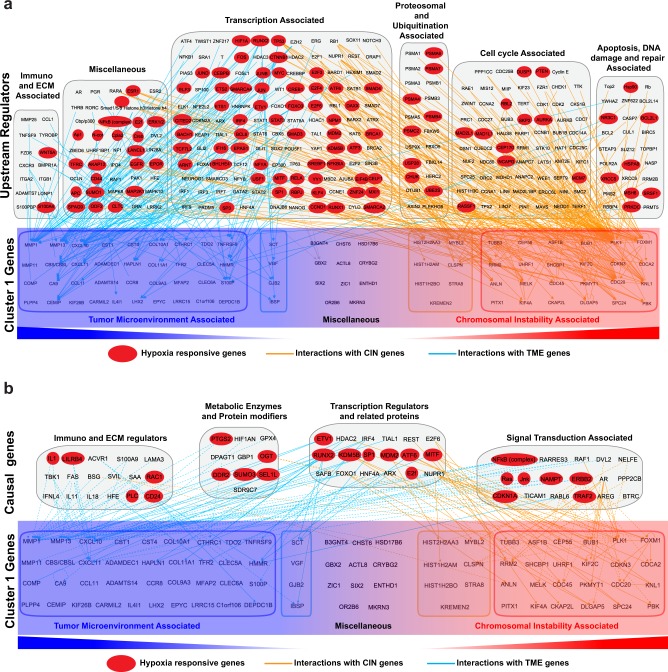
IPA analyses showing extensive interaction between hypoxia responsive genes with members of cluster 1 genes. **a** Upstream regulator analysis was performed with IPA for the cluster 1 genes and all the interactions retrieved are presented. Cluster 1 genes are classified into those that are associated with CIN or TME. The upstream genes that are hypoxia responsive, are highlighted in red. **b** Causal network analysis was performed with IPA for the cluster 1 genes and all the interactions retrieved are presented. Cluster 1 genes are classified into those that are associated with CIN or TME. The upstream genes that are hypoxia responsive, are highlighted in red

**Fig. 5:**
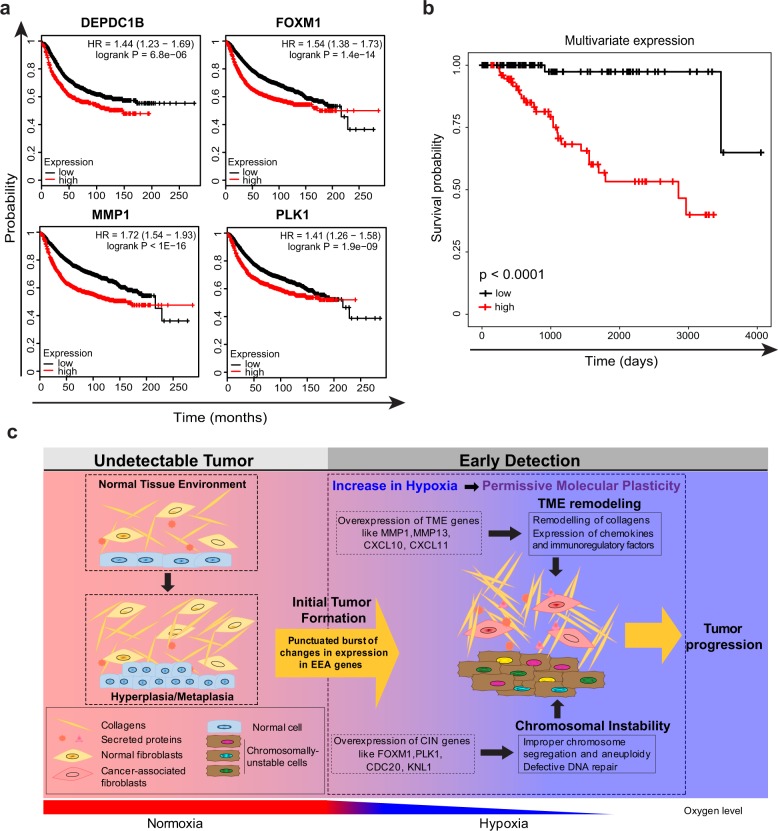
Survival plot and a model describing the role of cluster 1 genes in tumor evolution. **a** Representative relapse-free survival plots of breast cancer patients with low and high expression of cluster 1 genes. **b** Survival plots of TNBC patients with high and low multivariate gene expression score. **c** Schematic model showing the effect of simultaneous burst of CIN and TME-associated genes in response to hypoxia during early stages of cancer initiation

If both CIN and TME remodeling ensue simultaneously, we should ask what possible factors could drive such punctuated burst. To address this, we used recently published causal analyses tools^49^ available in the Ingenuity Pathway Analysis. In particular, we performed Upstream Regulator Analysis, and Causal Network Analysis to curate all interactions of cluster 1 genes (Fig. 4a, b). Interestingly, a large subset of direct upstream interactions as well as causal interactions of both the CIN and TME genes (cluster 1), are hypoxia responsive genes^50^ (Fig. 4a, b and Supplementary Table 8b, c). Invariably, almost 50% of the cluster 1 genes are also associated with poor prognosis (Fig. 5a and Supplementary Fig. 3). A multivariate expression score was derived from these 50% of the cluster 1 genes by the multivariate survival analysis to further evaluate their simultaneously effect on survival. TNBC patients were divided into two groups based on the multivariate expression score, one with high value multivariate expression and the other one with low expression. Survival probability curves of the two groups compared by the Kaplan–Meier method indicates a poor prognosis (Fig. 5b). This strongly suggests that very early in the course of tumor progression gradually increasing hypoxic conditions induce both CIN and TME remodeling to permit survival of cancer cells and their further evolution at later stages of malignancy.

## Discussion

Differential gene expression analyses have been traditionally used to examine fluctuations within the transcriptome in a given context for decades. This has been a powerful strategy to identify biomarkers and drug targets.^51–54^ However, tumor genome sequencing has provided new opportunities to re-examine these fluctuations in the context of tumor evolution. We rationalize that common aberrations detected across all patients arose earlier in the development of the malignancy compared to alterations that were found only in a subset of patients. Based on this, our strategy in this work is to explore the frequency of changes in the expression pattern of genes at different stages of TNBC progression. This is similar to previous studies that explored dynamic changes in mutations or CNA for a given patient at a given stage.^8–10,55,56^

Changes in gene expression, unless constitutively observed, are often ignored as stochastic noise, specifically those that arise from variations in transcriptional regulation or biochemical modifications within cells. Our analyses deliver several important observations. First, compared to mutational changes, alterations within the transcriptome are more common and occur at high frequency. For example, the highly significant mutations in genes like PIK3CA or KRAS are observed in ~30% of breast cancer patients. In contrast, overexpression of PLK1 or FOXM1 genes is observed in over 90% of patients (Supplementary Fig. 4). Second, more genes are downregulated compared to upregulated genes. Third, during tumor evolution, changes in expression pattern occur as punctuated bursts, where the initial singular burst results in simultaneous accumulation of overexpression of multiple EEA genes. Fourth, early changes in the expression of EEA molecules occur in genes that remodel TME and maintain chromosomal stability. This is most likely because survival within the progressively changing biological landscape during early stages requires cancer cells to both actively adjust to their microenvironment for their needs and to enhance CIN to facilitate their plasticity and adapt. Indeed, our unbiased genome-wide investigation reveals a strong functional connection between these two mechanisms and a crucial role of their coordinated effort in establishing early tumors. Interestingly, some TME genes, including MMP1, MMP11, and MMP13 proved to be upregulated at early stages and gradually downregulated through the later stages, although never achieving their normal levels. This suggests that their activities are essential at all stages of cancer progression, but their higher activity is required in early tumors, where the TME is not adjusted yet to the needs of malignant cells. Fifth, while we know that hypoxic TME can trigger tumor metastasis and invasion at later stages of cancer progression,^57,58^ our causal network analyses suggest that increasing hypoxia may be responsible for the cooperative induction of CIN and TME remodeling much earlier than previously appreciated (Fig. 5c). As hypoxic environment is also known to promote the propagation of tumor initiating cells (TICs),^59,60^ we suspect that the expressional changes of EEA genes may facilitate this process.

Although, CIN is nearly ubiquitous in cancer cells, and is considered as an important factor in tumor development,^61^ our findings indicate that hypoxic TME of early tumor may function as a trigger of genetic instability. This model is consistent with previous observations, showing that repeated cycles of hypoxia, can downregulate a number of DNA repair pathways in cancer cells, ultimately leading to genetic instability.^62,63^ In regards to this, the Glazer group has provided one of the first quantitative assessments of how genetic instability can be instigated by TME.^64^ Interestingly, several of the core EEA genes that maintain genome stability were experimentally shown to be involved in tumor development.^35–43^ Although some of these examples might be indicative of a direct role for CIN genes in tumorigenesis, in the context of our analyses, we suggest that overexpression of these genes may have enabled cancer cells to acquire properties that allowed them to survive at the early-stage of cancer and thus, to develop detectable tumors (Fig. 5c). This is particularly interesting in the light of apparent disagreements between the somatic mutation theory (SMT), where mutations are argued to be the primary cause of tumor evolution and tissue organization field theory (TOFT), that proposes a direct role for TME and its surrounding tissues in tumor development.^65,66^ In this context, our data agrees with a unified model, where tumor initiation is triggered by accumulated driver mutations, while its evolution depends on the interactions of cancer cells with TME (Fig. 5c).

In summary, our unbiased comprehensive analyses of the transcriptome directly link the early onset of hypoxia to the collective burst of CIN and TME remodeling factors in the initial stage of tumor progression, which highlights a therapeutic potential of targeting these molecules in TNBC tumors in their earliest detectable stage.

## Methods

### Number of patient samples analyzed

We collected data of breast cancer samples from the Cancer Genome Atlas (TCGA) with information on the copy number aberration, gene expression as well as tumor information. According to the stage information, 1078 samples were classified into four tumor stages; from stage I to stage IV with tumors in stage V not being considered in this study. According to the IHC markers, 158 samples were classified as TNBC tumors in which the ER, PR, and HER2 were all negative. With the tumor stage information, we classified TNBC tumors into TNBC-stage I, TNBC-stage II, TNBC-stage III, and TNBC-stage IV. Similarly, TNBC tumors in stage IV were excluded. Moreover, 114 normal samples were collected from TCGA for comparison with tumor sample data. The numbers of samples in each stage and TNBC-stage-specific samples are displayed in the Supplemental Table 9. No human or animal experiments were performed, which required prior approval.

### Fold-change and FDR calculation

We applied two criteria, fold-change and FDR calculation on the selection of differentially expressed genes. Fold-change is a biological assessment of changes in gene expression that is estimated by log2 (ratio), as represented in Equation 1, where the ratio of average expression of gene *i* in patients to the average expression in normal samples is calculated.

1 $${\mathrm{Fold}} - {\mathrm{change}}_{{\mathrm{gene}}\,i} = \log _2\frac{{{\mathrm{ave}}\left( {E_{{\mathrm{gene}}\,i}^{{\mathrm{patients}}}} \right)}}{{{\mathrm{ave}}\left( {E_{{\mathrm{gene}}\,i}^{{\mathrm{normal}}}} \right)}}$$

Empirical Bayes moderated t-test was applied to assess the statistical significance of differential expression. False discover rate (FDR) was obtained after Benjamini and Hochberg correction. We employed the Limma package^67^ to derive the two assessments of differentially expressed genes.

### Computing frequency of differential expression in stage-specific patients

After the identification of up/downregulated genes in each stage and TNBC-stage patients, next we aimed to evaluate the frequency of identified differential expression in stage-specific patients. For each tumor stage, we calculated the fold-change of identified up- or downregulated gene *i* by comparing the expression in patient *j* to the average expression in normal samples (Equation 2). Following this, the frequency of patients in which the fold-change of gene *i* is greater than 2 or less than −2 was calculated.

2 $${\mathrm{Fold}} - {\mathrm{change}}_{{\mathrm{gene}}\,i}^{{\mathrm{patient}}\,j} = \log \,2\frac{{E_{{\mathrm{gene}}\,i}^{{\mathrm{patient}}\,j}}}{{{\mathrm{ave}}\left( {E_{{\mathrm{gene}}\,i}^{{\mathrm{normal}}}} \right)}}$$

### Evaluating the concordance between copy number amplification and upregulated gene expression

As changes in gene expression may arise from the chromosomal amplification, here we aimed to evaluate the associations between copy number amplification (CNA) and upregulations in gene expression and identify the upregulations that are driven by CNA. We generated the CNA profile with patients (in rows) and genes (in columns) from the data obtained from TCGA. Only the data of patients whose CNA profile and upregulation status are available were considered for this study. Using a scoring system, genes getting amplified in a patient were represented as 1 or otherwise 0 and the amplified genes were grouped based on the profile scoring. To avoid patient heterogeneities, only genes showing amplification over 40% of patients were considered as cancer relevant amplified genes. CNA regions were identified by calculating the percentage of amplification of genes on each chromosome region, and regions with at least 40% of amplification genes were identified as CNA regions. Then we analyzed the concordance between CNA and upregulations in gene expression by two evaluation ways. Primarily, for each upregulated gene *i* at CNA regions, according to the gene *i*’s amplification status, the patient set ***S*** were grouped into two sets $\left\{ {S_i^1,S_i^2} \right\}$, where $S_i^1$ and $S_i^2$ denotes patients with and without gene ***i*** getting amplified, respectively. The fold CNA-associated change was calculated by comparing the difference between the log2 of mean expression of gene ***i*** in $S_i^1$ and $S_i^2$. Meanwhile empirical Bayes moderated *t*-test is applied on the two groups. To correct for multiple hypothesis testing, the *p*-value was converted to FDR by Benjamini-Hochberg correction. If gene ***i*** showed at least 1 positive fold CNA-associated change and FDR smaller than 0.01, it is considered to be associated strongly with CNA. Secondly, Pearson’s correlation coefficient was calculated to quantify the correlation between CNA and gene expression. If a gene showed the value of Pearson’s correlation coefficient larger than 0.3, it is considered as CNA-driven genes as well.

### TNBC clusters and neighboring-joining algorithm analyses

To identify the distinguishable TNBC subpopulations, which reveal similar upregulation profiles, we performed clustering analysis within TNBC patients. The binary matrix was generated with upregulation status in rows and patient in columns. If a gene is upregulated in a patient, it was indicated by 1, otherwise 0 using the scoring system described in the previous section. Then, we calculated pairwise Euclidian distance between patients and performed hierarchical clustering that clustered TNBC patients into distinguishable clusters. The mean upregulation profile for each cluster was generated to represent the upregulation status. Assuming that no gene upregulations appeared before the initiation of cancer, the profile with all zeros was generated to represent normal samples. The distance tree was constructed based on profiles in normal and mean profiles by Euclidian distance and neighbor-joining algorithm.

### Identifying genes in different clusters

To determine the appearance of upregulations in various subsets of the four subpopulations, we generated the four-dimensional binary vectors to represent each of the fifteen possible subsets of the four subpopulations, from (0, 0, 0, 1) to (1, 1, 1, 1). Four each gene *i*, the cosine similarities between mean profile *p*_*i*_ and each subset vector *v*_*i*_ is calculated by the Equation 3.

3 $${\rm{similarity}}\left( {p_i,v_j} \right) = \cos \left( \theta \right) = \frac{{p_i \cdot v_j}}{{\left\Vert {p_i} \right\Vert_2\left\Vert {v_j} \right\Vert_2}}$$

The gene was assigned to the subset vector with the maximum similarity to its mean profile.^68^ For example, if a gene has the maximum similarity with the subset vector (0, 1, 1, 1), it means it getting upregulated in subpopulations 2, 3, and 4.

### Multivariate survival analysis

We derived a multivariate expression score by multivariate Cox regression model, which evaluates the simultaneously effect of the multiple factors on survival. The regression coefficients in Cox regression relate to prognosis effect, thus we calculated the multivariate expression score as the sum of multiplications of the gene expression values by the respective regression coefficients. Based on the multivariate expression score, patients were divided into two groups using the median as the cutoff, one with high multivariate expression score and the other with low score. Survival analysis on the multivariate expression score was performed by Kaplan–Meier method.

### IPA and hypoxia analysis

IPA analysis was performed on the genes from cluster 1 as described in Kramer et al.^49^ The gene list was first annotated, and the dataset underwent various analyses including for core expression to study the interactions. The gene interactions were explored, built and different overlays including pathways, disease and function and molecule activity prediction were applied to obtain the required outputs. Comparison analysis was also performed among the different subpopulations (referred as clusters). Hypoxia analyses were performed using the hypoxia database (http://www.hypoxiadb.com). This database includes 72,000 manually curated entries taken on 3500 proteins extracted from 73 peer-reviewed publications selected from PubMed. As described in Khurana et al.^50^, it provides manually curated literature references to support the inclusion of the protein in the database and establish its association with hypoxia.

## Electronic supplementary material

Supplementary Information File

## Data Availability

The datasets analyzed during the current study are available in the TCGA databases. The authors declare that all the data generated during this study are included in this published article and its supplementary information files.

## Electronic supplementary material

**Supplementary information** accompanies the paper on the *npj Systems Biology and Applications* website (10.1038/s41540-018-0074-z).
