# Supplementary material for: Expression-based analyses indicate a central role for hypoxia in driving tumor plasticity through microenvironment remodeling and chromosomal instability
Source: NPJ Syst Biol Appl. 2018 Oct 24;4:38. doi: 10.1038/s41540-018-0074-z (PMC6200725; doi:10.1038/s41540-018-0074-z)
Supplement: Supplementary file 1 — Supplementary Information File [file 41540_2018_74_MOESM1_ESM.pdf]

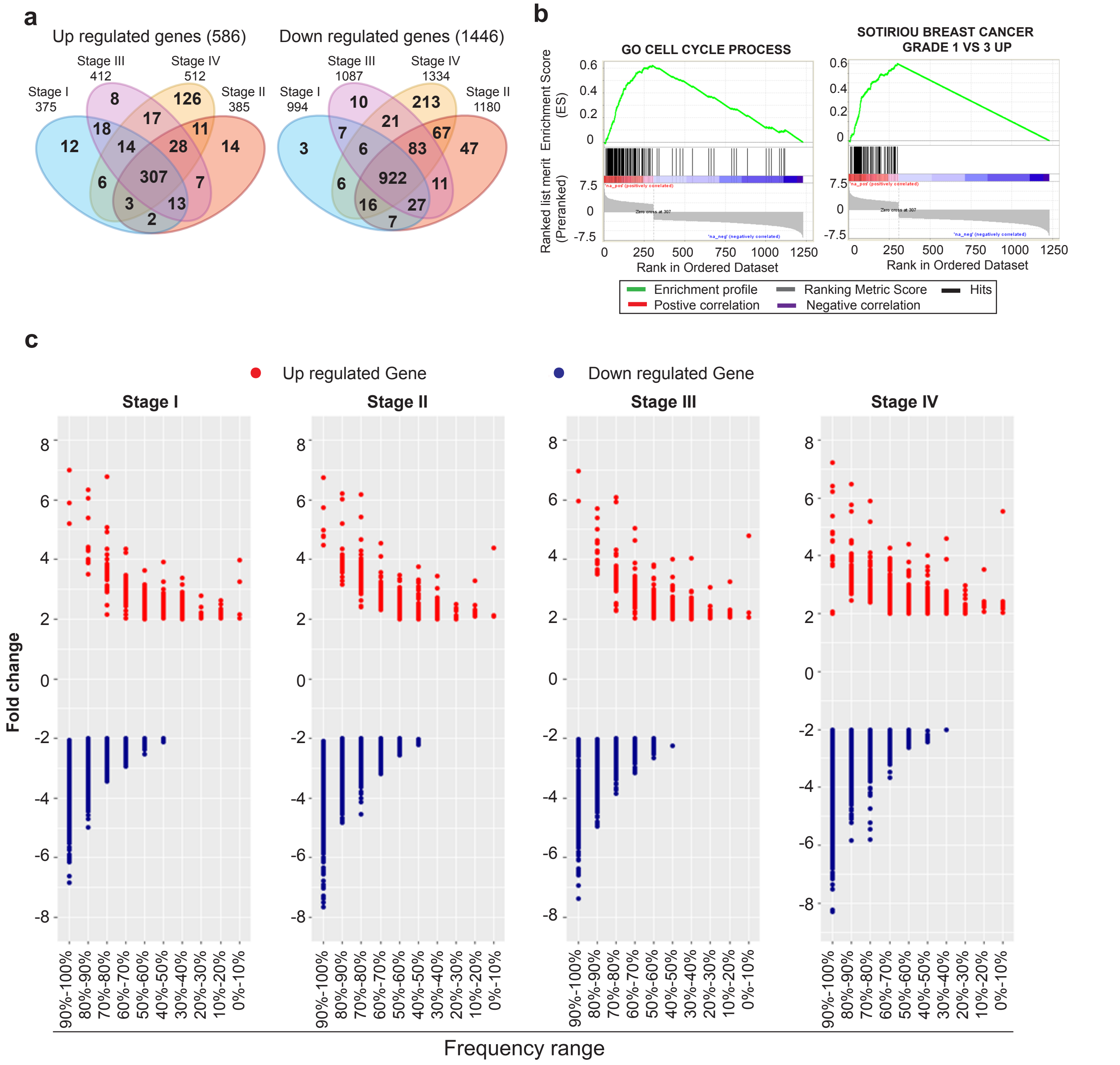
a
Up regulated genes (586)
Down regulated genes (1446)
Stage I
375
Stage III
412
Stage IV
512
Stage II
385
Stage I
994
Stage III
1087
Stage IV
1334
Stage II
1180
b
GO CELL CYCLE PROCESS
SOTIRIOU BREAST CANCER GRADE 1 VS 3 UP
Enrichment Score (ES)
Ranked list merit (Preranked)
Rank in Ordered Dataset
Enrichment profile
Postive correlation
Ranking Metric Score
Negative correlation
Hits
c
Up regulated Gene
Down regulated Gene
Stage I
Stage II
Stage III
Stage IV
Fold change
Frequency range

Jing *et al*., Supl Fig 2

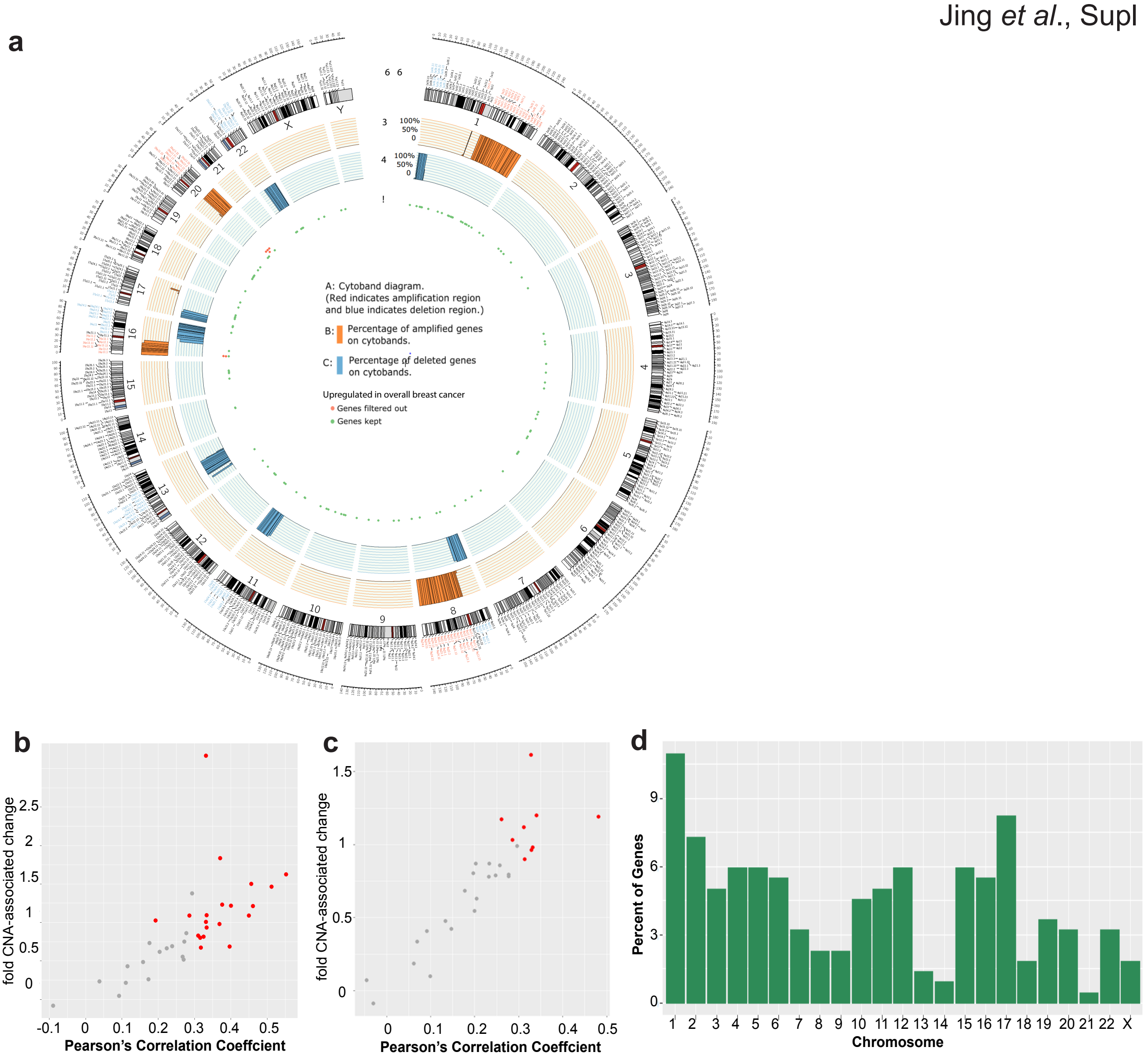

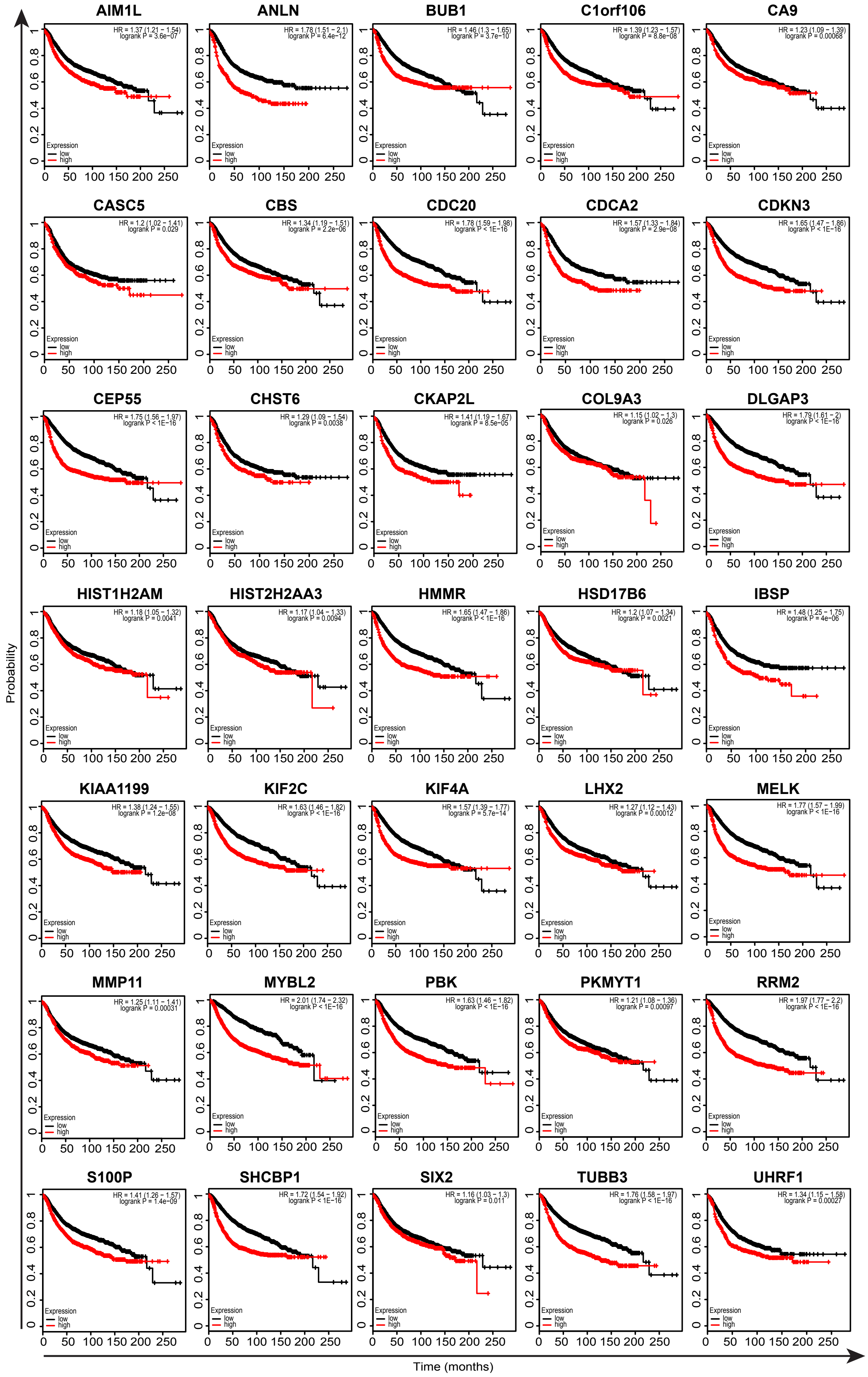
Probability
Time (months)
AIM1L
HR = 1.37 (1.21 − 1.54)
logrank P = 3.6e−07
ANLN
HR = 1.78 (1.51 − 2.1)
logrank P = 6.4e−12
BUB1
HR = 1.46 (1.3 − 1.65)
logrank P = 3.7e−10
C1orf106
HR = 1.39 (1.23 − 1.57)
logrank P = 8.8e−08
CA9
HR = 1.23 (1.09 − 1.39)
logrank P = 0.00068
CASC5
HR = 1.2 (1.02 − 1.41)
logrank P = 0.029
CBS
HR = 1.34 (1.19 − 1.51)
logrank P = 2.2e−06
CDC20
HR = 1.78 (1.59 − 1.98)
logrank P < 1E−16
CDCA2
HR = 1.57 (1.33 − 1.84)
logrank P = 2.9e−08
CDKN3
HR = 1.65 (1.47 − 1.86)
logrank P < 1E−16
CEP55
HR = 1.75 (1.56 − 1.97)
logrank P < 1E−16
CHST6
HR = 1.29 (1.09 − 1.54)
logrank P = 0.0038
CKAP2L
HR = 1.41 (1.19 − 1.67)
logrank P = 8.5e−05
COL9A3
HR = 1.15 (1.02 − 1.3)
logrank P = 0.026
DLGAP3
HR = 1.79 (1.61 − 2)
logrank P < 1E−16
HIST1H2AM
HR = 1.18 (1.05 − 1.32)
logrank P = 0.0041
HIST2H2AA3
HR = 1.17 (1.04 − 1.33)
logrank P = 0.0094
HMMR
HR = 1.65 (1.47 − 1.86)
logrank P < 1E−16
HSD17B6
HR = 1.2 (1.07 − 1.34)
logrank P = 0.0021
IBSP
HR = 1.48 (1.25 − 1.75)
logrank P = 4e−06
KIAA1199
HR = 1.38 (1.24 − 1.55)
logrank P = 1.2e−08
KIF2C
HR = 1.63 (1.46 − 1.82)
logrank P < 1E−16
KIF4A
HR = 1.57 (1.39 − 1.77)
logrank P = 5.7e−14
LHX2
HR = 1.27 (1.12 − 1.43)
logrank P = 0.00012
MELK
HR = 1.77 (1.57 − 1.99)
logrank P < 1E−16
MMP11
HR = 1.25 (1.11 − 1.41)
logrank P = 0.00031
MYBL2
HR = 2.01 (1.74 − 2.32)
logrank P < 1E−16
PBK
HR = 1.63 (1.46 − 1.82)
logrank P < 1E−16
PKMYT1
HR = 1.21 (1.08 − 1.36)
logrank P = 0.00097
RRM2
HR = 1.97 (1.77 − 2.2)
logrank P < 1E−16
S100P
HR = 1.41 (1.26 − 1.57)
logrank P = 1.4e−09
SHCBP1
HR = 1.72 (1.54 − 1.92)
logrank P < 1E−16
SIX2
HR = 1.16 (1.03 − 1.3)
logrank P = 0.011
TUBB3
HR = 1.76 (1.58 − 1.97)
logrank P < 1E−16
UHRF1
HR = 1.34 (1.15 − 1.58)
logrank P = 0.00027
Expression
low
high

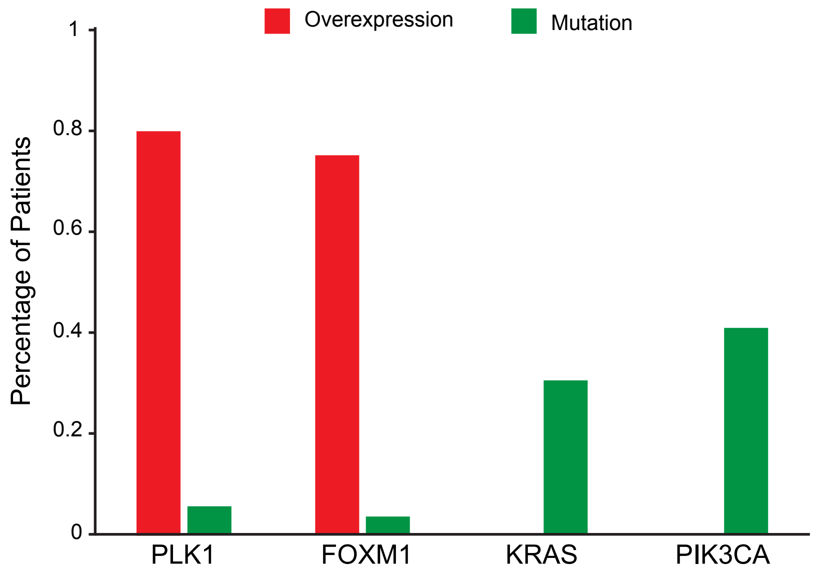
Overexpression
Mutation
Percentage of Patients
1
0.8
0.6
0.4
0.2
0
PLK1
FOXM1
KRAS
PIK3CA

SUPPLEMENTARY FIGURE LEGENDS:

Supplementary Figure 1: Identification of up-regulated genes in overall breast cancer. **(a)** Venn diagram of differentially expressed genes in overall (top) and ER+ patients (bottom) respectively. **(b)** Gene set enrichment analysis for up/down-regulated genes across all tumor stages. It shows the Gene Set Enrichment Analysis for 307 up-regulated genes (left) and 922 down-regulated genes (right) across four tumor stages along with previously identified, differentially up-regulated genes from Sotiriou et al [29]. **(c)** Frequency distribution of differential expression in overall patients. This plot represents the fold change and the frequency range of stage-specific differentially expressed genes, where the red denotes up-regulated gene and the blue denotes down-regulated gene.

Supplementary Figure 2: Expression pattern of up-regulated genes. **(a)** Amplified chromosome cytobands and up-regulated genes locus. Track A displays the cytoband diagram where the texts in red indicate identified amplified regions. Track B and C displays the frequency of genes showing amplification and deletion respectively at least in 40% of patients in each cytoband. Genes in supplementary figure 2a were mapped to the Track D. **(b and c)** The evaluation on the concordance between gene expression and amplification. Nodes represent up-regulated genes in overall breast cancer cases in amplified regions, showing Pearson's correlation coefficient and fold CNA-associated change. Genes in red were considered driven by copy number amplification either in overall breast cancer (B) or in TNBC (C). **(d)** Distribution of the 219 up-regulated events according to their chromosomal location.

Supplementary Figure 3: Relapse free survival plot in breast cancer patients having low and high expression of cluster 1 genes.

Supplementary Figure 4: Percentage of patients that either overexpress or carry mutations in some of the key cancer genes.

Supplementary Table 1a. Up/down regulated genes in overall patients

UpRegulatedGenes		DownRegulatedGenes	
**GeneName**	**GeneID**	**GeneName**	**GeneID**
COL10A1	1300	FHL1	2273
MMP11	4320	GLYAT	10249
PPAPDC1A	196051	PYGM	5837
IBSP	3381	TRDN	10345
NEK2	4751	MYH1	4619
HSD17B6	8630	TNNT3	7140
KIF4A	24137	FIGF	2277
GABRD	2563	MYOM1	8736
COL11A1	1301	MYOC	4653
PKMYT1	9088	LEP	3952
BMP8A	353500	HSPB7	27129
KIAA0101	9768	DEFB132	400830
UBE2T	29089	DES	1674
NUF2	83540	AMPD1	270
MMP13	4322	LOC283392	283392
CDK1	983	ACVR1C	130399
IQGAP3	128239	KIAA0408	9729
CDC25C	995	CA3	761
CKS2	1164	OXTR	5021
TPX2	22974	GYS2	2998
UHRF1	29128	GPAM	57678
ASF1B	55723	APOB	338
UBE2C	11065	GLRA4	441509
SPC25	57405	LHCGR	3973
HJURP	55355	ADH1B	125
PAFAH1B3	5050	C12orf39	80763
PLK1	5347	SMYD1	150572
TK1	7083	LOC729467	729467
PAQR4	124222	MYOM2	9172
NUSAP1	51203	LYVE1	10894
WISP1	8840	ADRA1A	148
CST1	1469	SLC2A4	6517
NUAK2	81788	HEPACAM	220296
CDKN3	1033	AQP7P1	375719
CCNB1	891	CKMT2	1160
ESM1	11082	KCNIP2	30819
COMP	1311	ADH1A	124
TROAP	10024	AQPEP	206338
CEP55	55165	AQP7P3	441432
CCL11	6356	GPD1	2819
GPRIN1	114787	SLC19A3	80704
NDC80	10403	HBB	3043
PROC	5624	HBA2	3040
AURKA	6790	ADIPOQ	9370
HMMR	3161	CSN1S1	1446
HAGHL	84264	LALBA	3906
CILP2	148113	CPA1	1357

WDR62	284403	CA4	762
KIF20A	10112	CD300LG	146894
MELK	9833	SCARA5	286133
LOC283867	283867	LPL	4023
ZWINT	11130	AQP7	364
OIP5	11339	ADH4	127
PTTG1	9232	KLB	152831
ADAMTS14	140766	PLIN1	5346
CLEC5A	23601	PLIN4	729359
CDCA8	55143	FRMD1	79981
KIF2C	11004	GYG2	8908
NCAPG	64151	SLC7A10	56301
PYCR1	5831	TUSC5	286753
KIF18B	146909	PCK1	5105
GJB2	2706	ANGPTL5	253935
TACC3	10460	HBA1	3039
BGN	633	GLP2R	9340
C16orf59	80178	HHATL	57467
BUB1B	701	SDPR	8436
RPLP0P2	113157	LRRC2	79442
FOXM1	2305	ATP1A2	477
DLGAP5	9787	HEPN1	641654
RRM2	6241	AOC3	8639
BUB1	699	ALDH1L1	10840
PBK	55872	RDH5	5959
SPAG5	10615	LIPE	3991
CHRNA6	8973	C14orf180	400258
SKA3	221150	RBP4	5950
TDO2	6999	CIDEC	63924
SKA1	220134	MRAP	56246
KIFC1	3833	LOC55908	55908
RAD51	5888	LGALS12	85329
CCNB2	9133	EPB42	2038
TOP2A	7153	SLC22A12	116085
CCDC64	92558	HSD17B13	345275
CKAP2L	150468	TNMD	64102
GTSE1	51512	HSPB6	126393
ANLN	54443	SORBS1	10580
DTL	51514	FAM180B	399888
NEIL3	55247	CRHBP	1393
EXO1	9156	CES1	1066
CDCA5	113130	GSTM5	2949
ASPM	259266	BMP3	651
CDC20	991	PRRT4	401399
UBE2S	27338	TRHDE	29953
CDCA3	83461	HPD	3242
SAMD14	201191	HSD3B2	3284
SPC24	147841	LOC572558	572558
RAD54L	8438	ANGPT4	51378
EPR1	8475	KCNJ16	3773

C16orf75	116028	ACACB	32
CCNE2	9134	CHRDL1	91851
INHBA	3624	TMEM132C	92293
CENPF	1063	C13orf36	400120
ERCC6L	54821	RXRG	6258
TLCD1	116238	KLHL31	401265
RECQL4	9401	PFKFB1	5207
KIF23	9493	ABCD2	225
BIRC5	332	CIDEA	1149
CENPM	79019	GPR144	347088
PRC1	9055	PDE3B	5140
KIF14	9928	LRRC3B	116135
LMNB1	4001	BTNL9	153579
AURKB	9212	ITIH5	80760
KIF11	3832	MESTIT1	317751
NCAPH	23397	NAALAD2	10003
FAM83D	81610	ACSM5	54988
FAM54A	113115	PCOLCE2	26577
DEPDC1B	55789	CASQ2	845
SLC24A2	25769	G0S2	50486
EZH2	2146	ALB	213
GRM8	2918	MME	4311
CTHRC1	115908	NPR1	4881
CST2	1470	TRPM3	80036
CCNA2	890	ANGPTL7	10218
MKI67	4288	SCN4A	6329
CTXN1	404217	PCDH9	5101
KIF26B	55083	ITGA7	3679
FN1	2335	PAPPA2	60676
CENPA	1058	KLHL33	123103
SHCBP1	79801	FABP4	2167
CNIH2	254263	KCNA4	3739
PLAC1	10761	PGA3	643834
CDC45	8318	SEL1L2	80343
KIF15	56992	GABRA4	2557
SGOL1	151648	PENK	5179
P4HA3	283208	CYP1A1	1543
GINS1	9837	CSN3	1448
LRRC15	131578	ADAMTS5	11096
DEPDC1	55635	CLEC3B	7123
SCT	6343	SVEP1	79987
ESCO2	157570	MAP1LC3C	440738
ESPL1	9700	PKHD1L1	93035
HIST1H2AM	8336	NPY2R	4887
EME1	146956	FAM196B	100131897
KIAA1199	57214	CDH20	28316
MYBL2	4605	MYBPC1	4604
MMP1	4312	TNS1	7145
PACSIN1	29993	B3GALT1	8708
EPYC	1833	MASP1	5648

PPEF1	5475	ABCA9	10350
C20orf103	24141	PDK4	5166
CDC6	990	GDF10	2662
E2F8	79733	CDO1	1036
E2F1	1869	CD36	948
CENPE	1062	GPX3	2878
SDS	10993	SPHKAP	80309
SALL4	57167	SAA2	6289
FAM64A	54478	ALDH3A1	218
MFAP2	4237	CLDN19	149461
HIST2H2AA3	8337	SGCG	6445
MARCH4	57574	PROKR1	10887
CCDC19	25790	CSF3	1440
GRM4	2914	DMD	1756
SLC16A3	9123	FXYD1	5348
FAM111B	374393	LOC728264	728264
TTK	7272	TNXB	7148
POLQ	10721	KLF15	28999
HCN2	610	ASPA	443
MATN3	4148	ABCB5	340273
RHPN1	114822	ACADL	33
TMEM90B	79953	ABCA8	10351
MCM10	55388	GPR109A	338442
KIF18A	81930	KY	339855
AIM1L	55057	PLXNA4	91584
RDM1	201299	SGK2	10110
CST4	1472	SLC16A7	9194
E2F7	144455	OVCH2	341277
HIST1H3H	8357	DLK1	8788
CCDC78	124093	LOC93432	93432
SBK1	388228	KIAA1239	57495
CDCA2	157313	HIF3A	64344
TUBB3	10381	EBF1	1879
RAB26	25837	GPIHBP1	338328
ADAM8	101	FMO2	2327
CLSPN	63967	IGSF10	285313
GPRC5A	9052	STAB2	55576
TFR2	7036	GCOM1	145781
HES6	55502	NMUR1	10316
HIST1H2BO	8348	NNAT	4826
FAM72D	728833	TIMP4	7079
HIST1H2BD	3017	CLCA4	22802
ORC6L	23594	FGFBP2	83888
CDT1	81620	AADAC	13
OLR1	4973	ITIH2	3698
KIAA1211	57482	CAV1	857
MND1	84057	RYR3	6263
PITX1	5307	ABCA10	10349
E2F2	1870	HPSE2	60495
S100P	6286	DPP6	1804

HIST2H3C	126961	AKR1C1	1645
FAM72A	729533	PPP1R1A	5502
TNFRSF18	8784	DCT	1638
ITPKA	3706	SAA1	6288
KREMEN2	79412	CLEC4G	339390
ISG15	9636	DPT	1805
DPEP1	1800	MMD	23531
CASC5	57082	SLCO1A2	6579
IGFL2	147920	IGFBP1	3484
CAMK2N2	94032	PROL1	58503
WBSCR28	135886	NTS	4922
HIST1H3D	8351	SLC17A7	57030
HTR1D	3352	MYH11	4629
METRN	79006	ANGPTL1	9068
ADM2	79924	TMEM100	55273
IL4I1	259307	GRIA4	2893
HIST1H2BH	8345	PKD1L2	114780
F12	2161	BHMT2	23743
SYNGR3	9143	MRGPRX2	117194
ORC1L	4998	DGAT2	84649
COL1A1	1277	PRG4	10216
OR2B6	26212	PAMR1	25891
NKX3-2	579	ATOH8	84913
IL21R	50615	KCNB1	3745
FBN2	2201	PPARG	5468
ZNF695	57116	GHR	2690
C9orf169	375791	CNN1	1264
HAPLN1	1404	CLEC4GP1	440508
C9orf140	89958	ANGPT1	284
ARTN	9048	P2RX6	9127
ACAN	176	CYP3A4	1576
RAMP1	10267	RNF186	54546
TMEM145	284339	SLC7A3	84889
CAMP	820	DSC1	1823
C19orf21	126353	RPE65	6121
DSCR6	53820	AQP4	361
NKAIN1	79570	TSLP	85480
SRMS	6725	IGFBP6	3489
B4GALNT4	338707	NIPSNAP3B	55335
MS4A15	219995	CXCL2	2920
GNG13	51764	LOC90586	90586
C6orf126	389383	CCDC3	83643
CXCL10	3627	TF	7018
CRB3	92359	KCNE1	3753
KRT8	3856	AKR1C2	1646
KLK4	9622	DMGDH	29958
CXCL11	6373	PI16	221476
IFI6	2537	C2orf84	653140
MMP9	4318	C4BPA	722
CNTNAP2	26047	LOC100124692	100124692

WT1	7490	PDE2A	5138
LRFN2	57497	ARHGAP20	57569
EDN2	1907	RERGL	79785
DQX1	165545	VIT	5212
HSH2D	84941	ADH1C	126
HIST1H4H	8365	ADCYAP1R1	117
EPN3	55040	C7orf58	79974
FAM57B	83723	TMC2	117532
TNFRSF9	3604	HS3ST4	9951
SPOCD1	90853	KBTBD12	166348
CNTD2	79935	CA6	765
CCR8	1237	COL25A1	84570
MUC1	4582	C10orf90	118611
MAPK8IP2	23542	C2orf40	84417
IGSF9	57549	SAMD5	389432
ARMC3	219681	NMNAT2	23057
C19orf51	352909	GPLD1	2822
S100A14	57402	CFD	1675
ZMYND10	51364	FOSB	2354
TJP3	27134	MLXIPL	51085
KRT19	3880	THRSP	7069
TNNT1	7138	FAM150B	285016
CPNE7	27132	PAK7	57144
LHX2	9355	PTPRQ	374462
CAPSL	133690	WIF1	11197
C20orf151	140893	CALB2	794
AP1M2	10053	LOC284578	284578
APOC2	344	CHL1	10752
JAKMIP1	152789	S100B	6285
VGF	7425	CES4	51716
MYT1	4661	KIT	3815
LOC400696	400696	GPBAR1	151306
C1orf230	284485	EDN3	1908
ADAMDEC1	27299	FAM3D	131177
GAL3ST2	64090	GSTA1	2938
ESRP1	54845	PIGR	5284
KISS1R	84634	ARHGAP36	158763
RAB25	57111	MAMDC2	256691
HIST1H2BG	8339	GPR146	115330
ACTL8	81569	CAPN11	11131
CHRNA1	1134	SYNM	23336
NEURL	9148	C2orf88	84281
PRSS8	5652	CRYAB	1410
NMU	10874	C18orf34	374864
HOXC11	3227	SCN3A	6328
HOXC13	3229	LRRN4CL	221091
CEACAM5	1048	SLC6A2	6530
CAPN9	10753	COL6A6	131873
FOXJ1	2302	PDE1C	5137
CDC20B	166979	MAOA	4128

BMPR1B	658	PAK3	5063
CEACAM6	4680	MMRN1	22915
PP14571	100130449	PIK3C2G	5288
MAPK15	225689	SLC14A2	8170
EEF1A2	1917	SMR3B	10879
HOXB13	10481	LEPR	3953
PYDC1	260434	DACT2	168002
CGA	1081	ANKRD53	79998
CA9	768	SLC7A14	57709
INSM1	3642	HAS3	3038
		PLAC9	219348
		NRXN1	9378
		GRIN2B	2904
		MMP27	64066
		CAPZA3	93661
		IRX6	79190
		ANO3	63982
		SPINK5	11005
		CPA2	1358
		DSG1	1828
		FABP7	2173
		C6	729
		CAV2	858
		HOXA5	3202
		EBF3	253738
		FZD4	8322
		BMP5	653
		RHOXF1	158800
		MOSC1	64757
		TMEM37	140738
		CMTM5	116173
		DRD2	1813
		APCDD1L	164284
		SPOCK3	50859
		CDR1	1038
		TMEM213	155006
		AFP	174
		LMOD1	25802
		NKAPL	222698
		ALDH1A2	8854
		C1QTNF9	338872
		FREM1	158326
		RELN	5649
		C14orf139	79686
		C3orf55	152078
		GFAP	2670
		GALNTL2	117248
		CLDND2	125875
		PLD5	200150
		SCN2A	6326

MYEOV	26579
SYN2	6854
NTRK2	4915
BMX	660
RNF150	57484
NLGN1	22871
D4S234E	27065
CNTFR	1271
ACTG2	72
XPNPEP2	7512
NEUROG2	63973
GRIA1	2890
PTN	5764
GRIK1	2897
GLRA3	8001
SLC26A3	1811
MEOX2	4223
PRRG3	79057
CCL14	6358
KLHL13	90293
SAA4	6291
TMEM195	392636
GPR133	283383
RNASE7	84659
DMRT2	10655
KCNA2	3737
CCDC141	285025
CLDN10	9071
RASGEF1C	255426
LIFR	3977
PALMD	54873
FAM107A	11170
FGF2	2247
AVPR2	554
CCDC69	26112
SFRP1	6422
EPHB1	2047
HTR2A	3356
TEPP	374739
CMA1	1215
CD209	30835
FGFBP1	9982
ALX4	60529
GABRA2	2555
LMX1A	4009
TTYH1	57348
IL6	3569
OLFM4	10562
CITED1	4435
COL17A1	1308

MUCL1	118430
PLA2G2A	5320
LRRTM2	26045
PGM5	5239
SIK2	23235
HLF	3131
NRG2	9542
LRRN3	54674
ART4	420
LPPR1	54886
PI3	5266
C8orf34	116328
NTF4	4909
C5orf23	79614
ROPN1B	152015
RSPO3	84870
PDZD2	23037
SHISA3	152573
LILRB5	10990
LOC286002	286002
KLK5	25818
HOXA4	3201
ENPP2	5168
SEMA3G	56920
SLC22A3	6581
ZBTB16	7704
CNKSR2	22866
SLC16A12	387700
ELANE	1991
ACSL1	2180
DLK2	65989
PTCHD3	374308
BBOX1	8424
MYO16	23026
SLC4A4	8671
CDH4	1002
CHST9	83539
ATP13A5	344905
B3GAT1	27087
RFX6	222546
MAB21L1	4081
GIPC2	54810
SSTR1	6751
F10	2159
FHL5	9457
RGS6	9628
SCN2B	6327
DNASE1L3	1776
BMP2	650
PLP1	5354

LOXL4	84171
AOC2	314
PTPRZ1	5803
CSRNP3	80034
PTGS2	5743
SOX10	6663
MARCO	8685
ROPN1	54763
OCA2	4948
SYCE1	93426
CDH19	28513
KLK7	5650
C4orf49	84709
LPHN3	23284
JPH2	57158
MYOCD	93649
SCN9A	6335
LOC201651	201651
LOC339524	339524
KCTD4	386618
GABRE	2564
PHKG1	5260
PTCHD1	139411
EFCAB1	79645
IL17B	27190
ADRB1	153
SOSTDC1	25928
EDAR	10913
STAC2	342667
KRT5	3852
KRT14	3861
C2orf82	389084
GSN	2934
ADRB2	154
HOXA6	3203
GPC3	2719
AKAP12	9590
RGS7BP	401190
AOX1	316
FAM89A	375061
NCRNA00092	100188953
NAT8L	339983
EMILIN3	90187
IL22RA2	116379
TAT	6898
KLHL29	114818
LOC286367	286367
DENND2A	27147
MATN2	4147
SYNPO2	171024

ADAM33	80332
GPR17	2840
BMPER	168667
SNTG2	54221
GPR12	2835
ACE2	59272
TSPAN8	7103
KLK8	11202
CLDN8	9073
HSPB2	3316
C1QTNF9B	387911
TPO	7173
ALDH1A1	216
CLDN5	7122
HOXA9	3205
KCNMB1	3779
LOC158376	158376
GLDN	342035
ASTN1	460
DDR2	4921
PSD2	84249
C1QTNF7	114905
CCL28	56477
TP53AIP1	63970
PI15	51050
FAM13A	10144
GNAI1	2770
PID1	55022
INMT	11185
IQSEC3	440073
AKAP6	9472
EGR1	1958
C11orf92	399948
NCAM1	4684
PIWIL2	55124
HSD11B1	3290
C1QL2	165257
LOC121838	121838
DCX	1641
GABRP	2568
HOXA7	3204
PHYHIP	9796
CNTNAP3	79937
ABCA6	23460
IL33	90865
TACR1	6869
AKR1C3	8644
CXCL3	2921
FLJ37543	285668
LMO3	55885

CXCL5	6374
SCGB3A1	92304
NRN1	51299
LOC145820	145820
KLF4	9314
MEOX1	4222
OSR1	130497
WSCD2	9671
C14orf49	161176
PTGFR	5737
LOC440173	440173
PROX1	5629
OGN	4969
PCDH11X	27328
KCNS1	3787
SLC6A14	11254
KLK6	5653
USP44	84101
TSHZ2	128553
CCL16	6360
C10orf108	414235
TMEM178	130733
SOD3	6649
ECM2	1842
STK32A	202374
CRTAC1	55118
TP63	8626
MUC15	143662
FOLR1	2348
LY6D	8581
TAC1	6863
C7orf41	222166
MRAS	22808
NECAB1	64168
EGFR	1956
CX3CL1	6376
PPP1R14A	94274
ODZ1	10178
SCN5A	6331
GFRA2	2675
PPARGC1A	10891
LCN6	158062
CLDN2	9075
OR2L13	284521
CCDC129	223075
VGLL1	51442
LGI4	163175
TGFBR3	7049
RGN	9104
COX7A1	1346

ODF3L1	161753
EBF2	64641
KCNA1	3736
BNC1	646
SLC27A6	28965
DEFB1	1672
GPR64	10149
AK5	26289
NRG1	3084
SLC13A2	9058
KL	9365
NDRG2	57447
GNAL	2774
LOC134466	134466
HOXA2	3199
STXBP6	29091
FOXP2	93986
TESC	54997
C2orf71	388939
PLCXD3	345557
CAPN6	827
PTX3	5806
IRX1	79192
TUBB2B	347733
NR4A1	3164
DBC1	1620
SCN4B	6330
ITPRIPL1	150771
ASAM	79827
ANK2	287
OXGR1	27199
LUZP2	338645
GLB1L3	112937
TFPI2	7980
ELF5	2001
SRPX	8406
TMTC1	83857
MT1M	4499
KLF14	136259
EMX2OS	196047
RGS2	5997
APOD	347
MRGPRX3	117195
DNER	92737
UGT8	7368
SCRG1	11341
CYP2A6	1548
C4orf7	260436
DST	667
AGAP11	119385

ADAMTS1	9510
CLIC5	53405
PDE11A	50940
C13orf33	84935
TCEAL2	140597
NPY5R	4889
CCL13	6357
IGSF1	3547
SPRY2	10253
FAM13C	220965
PRELP	5549
CADM3	57863
ATF3	467
FOS	2353
FGF9	2254
PPYR1	5540
CACNA2D1	781
TRIM29	23650
CLCA2	9635
HSD17B2	3294
MIA	8190
MUC6	4588
ANXA1	301
DLGAP2	9228
BAI3	577
RBP7	116362
C10orf10	11067
P2RY12	64805
SOX5	6660
C5orf27	202299
FGF10	2255
C1orf130	400746
SCN11A	11280
PLA2G4A	5321
SLC10A6	345274
FAT2	2196
DNAH9	1770
KCNH8	131096
ANKRD29	147463
TDRD10	126668
MYLK	4638
CASP12	120329
ID4	3400
CCDC85A	114800
HFM1	164045
KCNMB2	10242
SNCG	6623
PREX2	80243
RAB3C	115827
PDE8B	8622

MRC1	4360
KRT17	3872
HMGCS2	3158
APCDD1	147495
FLJ42875	440556
TRPM6	140803
PTH1R	5745
DOCK11	139818
ANGPTL4	51129
MAPK4	5596
KRT15	3866
NR3C2	4306
TSPAN7	7102
SCN3B	55800
FAM149A	25854
CLDN11	5010
SLC25A27	9481
DHDPSL	112817
PKNOX2	63876
EGR3	1960
SMOC1	64093
GPR88	54112
GPM6A	2823
LGR6	59352
PPP1R12B	4660
CORO2B	10391
PER1	5187
NCRNA00086	399668
ASXL3	80816
EGR2	1959
ADAMTS18	170692
CCL21	6366
LRP1B	53353
SLC34A2	10568
GPM6B	2824
FAM189A2	9413
PACRG	135138
HRASLS5	117245
HRCT1	646962
SLC28A3	64078
CACHD1	57685
C21orf34	388815
USHBP1	83878
C5orf4	10826
FAM70A	55026
DTX1	1840
CCBE1	147372
GREM2	64388
KLK10	5655
GPRASP1	9737

NCALD	83988
THSD7B	80731
PTH2R	5746
KCTD14	65987
GPR109B	8843
LRFN5	145581
PRSS12	8492
ZNF676	163223
DSG3	1830
KLK11	11012
ITM2A	9452
MAML2	84441
SV2B	9899
DARC	2532
RIMBP2	23504
PKP1	5317
SOX6	55553
IGJ	3512
SYT8	90019
GNG11	2791
EFHA2	286097
TCEAL7	56849
RBMS3	27303
VWCE	220001
IGF1	3479
SLC26A4	5172
CYP39A1	51302
GAS2L2	246176
SCN7A	6332
COL4A6	1288
WISP3	8838
ANXA8L2	244
TMEM220	388335
FAM162B	221303
ISM1	140862
WSCD1	23302
CPEB1	64506
RCAN1	1827
SEMA3D	223117
WDR86	349136
FIGN	55137
FRMD3	257019
SERPINB5	5268
ADAMTSL4	54507
KCNA6	3742
ABI3BP	25890
PDZRN4	29951
ARC	23237
PEAR1	375033
CREB5	9586

PCDHGA3	56112
SH3TC2	79628
HRNR	388697
VSIG2	23584
LDB2	9079
C9orf125	84302
TGFBR2	7048
EDNRB	1910
C10orf128	170371
DBX2	440097
ABCC6P1	653190
GDF5	8200
CTSG	1511
ODZ2	57451
CDKN1C	1028
BMP6	654
PRIMA1	145270
RASD1	51655
CNGA1	1259
PALM2-AKAP2	445815
NBLA00301	79804
HOXA3	3200
SEMA6D	80031
CDH23	64072
MFAP4	4239
STAC	6769
C7	730
DSC3	1825
LRRC70	100130733
KCNAB1	7881
TMOD1	7111
NFIB	4781
MT1X	4501
RGMA	56963
CNR1	1268
KIAA1045	23349
PTGIS	5740
AQP1	358
GGTA1	2681
RASSF9	9182
ABCB1	5243
GPR44	11251
ST6GALNAC1	55808
CPS1	1373
NGFR	4804
LAMB3	3914
TWIST2	117581
LCN10	414332
CNNM1	26507
SHE	126669

WASF3	10810
SLIT3	6586
NTRK3	4916
FGF1	2246
NAP1L2	4674
GRRP1	79927
DUSP1	1843
PCDHGA2	56113
MEG3	55384
CDKL2	8999
MAP7D3	79649
EFEMP1	2202
FAM126A	84668
LAMA3	3909
ZNF727	442319
PIP5K1B	8395
CPAMD8	27151
ADCY5	111
C16orf89	146556
PLAGL1	5325
PLEKHH2	130271
PVRL3	25945
L3MBTL4	91133
ALDH1A3	220
FERMT1	55612
SH3BGRL2	83699
PARK2	5071
BOC	91653
ADHFE1	137872
CTTNBP2	83992
DOC2B	8447
NOVA1	4857
LIMS2	55679
MET	4233
RUNX1T1	862
F3	2152
C8orf79	57604
STOX2	56977
C1orf95	375057
S1PR1	1901
MRGPRF	116535
HMGCLL1	54511
MID1	4281
CYP11A1	1583
TNFRSF10D	8793
CRYBG3	131544
ARHGEF4	50649
C4orf31	79625
PLSCR4	57088
ROBO3	64221

FAT4	79633
LHFP	10186
PROS1	5627
RIC3	79608
SMAD9	4093
TNNI3K	51086
CLIP4	79745
DCN	1634
PCDHGB6	56100
PPAP2B	8613
CYYR1	116159
COL14A1	7373
MRAP2	112609
PPP1R1B	84152
SELP	6403
PDGFD	80310
CCDC46	201134
FLRT2	23768
PCDHGB7	56099
ZNF204P	7754
NLGN4X	57502
CNTN1	1272
TINAGL1	64129
CXCL12	6387
PELI2	57161

Supplementary Table 1b. Up/down regulated genes in TNBC patients

UpRegulatedGenes		DownRegulatedGenes	
**GeneName**	**GeneID**	**GeneName**	**GeneID**
MMP11	4320	SCN4B	6330
COL10A1	1300	ANKRD29	147463
AURKA	6790	FIGF	2277
TPX2	22974	TGFBR3	7049
FAM72B	653820	NOVA1	4857
PRC1	9055	SDPR	8436
PLK1	5347	PAMR1	25891
CCNE1	898	SSTR1	6751
HJURP	55355	CA4	762
TACC3	10460	C5orf4	10826
CDCA8	55143	PGM5	5239
CDCA3	83461	ADAMTS5	11096
CDK1	983	CD300LG	146894
SKA1	220134	LMOD1	25802
GTSE1	51512	FXYD1	5348
UBE2T	29089	MME	4311
FAM54A	113115	JAM2	58494
PTTG1	9232	MAMDC2	256691
NUF2	83540	MATN2	4147
CEP55	55165	SCN2B	6327
CCNA2	890	ABCA10	10349
CENPA	1058	PDE2A	5138
FOXM1	2305	HOXA5	3202
FAM83D	81610	HOXA7	3204
KPNA2	3838	TMTC1	83857
C1orf135	79000	PDGFD	80310
CCNB1	891	KL	9365
FAM72D	728833	MAB21L1	4081
UBE2C	11065	MYH11	4629
CDCA5	113130	PARK2	5071
NDC80	10403	LOC572558	572558
ANLN	54443	SYNPO2	171024
HMGB3	3149	RERGL	79785
FAM72A	729533	ALDH1A2	8854
IQGAP3	128239	GPIHBP1	338328
KIF4A	24137	ADAM33	80332
DEPDC1	55635	SCARA5	286133
PIF1	80119	SH3BGRL2	83699
PAQR4	124222	ARHGAP20	57569
SCT	6343	LDB2	9079
STMN1	3925	ABCA8	10351
MFAP2	4237	AGTR1	185
GPRIN1	114787	DACH1	1602
NCAPG	64151	GPC3	2719

BUB1	699	FGF10	2255
SPAG5	10615	TSLP	85480
CDKN3	1033	CTTNBP2	83992
TROAP	10024	CAPN11	11131
CDC20	991	PEAR1	375033
KIF14	9928	BTNL9	153579
MYBL2	4605	ACOX2	8309
LRP8	7804	LRRC3B	116135
GJB2	2706	TPRG1	285386
CENPF	1063	MEOX2	4223
RAD51	5888	LIFR	3977
MELK	9833	TNXB	7148
UBE2S	27338	DIXDC1	85458
KIFC1	3833	ABCA9	10350
CCNB2	9133	GIPC2	54810
DEPDC1B	55789	NOSTRIN	115677
NEK2	4751	C6orf97	80129
KIF2C	11004	EMCN	51705
SKA3	221150	SPARCL1	8404
ORC6L	23594	OGN	4969
SPC24	147841	CORO2B	10391
CDCA2	157313	PPP1R12B	4660
CKAP2L	150468	CACHD1	57685
OIP5	11339	AGAP11	119385
MCM10	55388	SPRY2	10253
TDO2	6999	CCL14	6358
SGOL1	151648	NTRK2	4915
RAD54L	8438	LRRN4CL	221091
KIF20A	10112	CPA1	1357
EZH2	2146	C1QTNF9	338872
ZNF695	57116	RAPGEF3	10411
AURKB	9212	HLF	3131
E2F1	1869	TCEAL7	56849
CDC25C	995	C16orf89	146556
KIF18B	146909	BMX	660
H2AFX	3014	INMT	11185
TRIP13	9319	FAM13C	220965
CKS2	1164	ANGPTL1	9068
SHCBP1	79801	TPO	7173
TUBB3	10381	AVPR2	554
NMU	10874	TSPAN7	7102
NEIL3	55247	RAI2	10742
CBX2	84733	MAPT	4137
HN1	51155	THRB	7068
C9orf100	84904	DHDPSL	112817
MAD2L1	4085	ITIH5	80760
C15orf42	90381	C9orf125	84302

ASF1B	55723	CAV1	857
ERCC6L	54821	ADCY5	111
PTTG3P	26255	NKAPL	222698
DLGAP5	9787	MAOA	4128
FAM64A	54478	ATP1A2	477
MKI67	4288	SLC16A12	387700
CA9	768	ABLIM3	22885
TTK	7272	SCN3B	55800
TNFRSF9	3604	LOC134466	134466
TK1	7083	MYRIP	25924
CNIH2	254263	SEPP1	6414
RRM2	6241	TNMD	64102
IL4I1	259307	SEMA6D	80031
VGF	7425	PCDHGB7	56099
BLM	641	RNASE4	6038
STIL	6491	C21orf34	388815
AIM1L	55057	TPPP	11076
GINS1	9837	GHR	2690
CST1	1469	ATOH8	84913
KIF23	9493	NAV3	89795
SH2D2A	9047	SEMA3G	56920
BIRC5	332	ESR1	2099
CENPI	2491	KIAA1377	57562
PKMYT1	9088	FLRT2	23768
LOC339674	339674	MSRB3	253827
ECE2	9718	CXCL14	9547
E2F8	79733	PDK4	5166
CXCL11	6373	C1orf190	541468
ACTL8	81569	TTC28	23331
KIF18A	81930	SCN7A	6332
C16orf75	116028	MMRN2	79812
GRIN2D	2906	IGFBP6	3489
LHX2	9355	GRIA1	2890
HMGA1	3159	CHL1	10752
IL21R	50615	TMEM178	130733
PBK	55872	EDNRB	1910
INHBA	3624	AHNAK	79026
WISP1	8840	ADH1C	126
MND1	84057	RBMS3	27303
HIST2H3C	126961	PCDH19	57526
NKX3-2	579	BCL2	596
RECQL4	9401	PELI2	57161
ZWINT	11130	IGSF10	285313
E2F2	1870	LIMS2	55679
RELT	84957	PGR	5241
TFR2	7036	DLGAP2	9228
CILP2	148113	LRRTM2	26045

FOXP3	50943	MESTIT1	317751
BGN	633	SCN3A	6328
ESPL1	9700	FHL5	9457
GINS4	84296	NR3C2	4306
CCL7	6354	ZBTB16	7704
EME1	146956	ITM2A	9452
ESCO2	157570	GFRA1	2674
MFSD2B	388931	HOXA6	3203
A2ML1	144568	CHRDL1	91851
SLC16A3	9123	EFEMP1	2202
TLX1	3195	ZNF676	163223
CD80	941	CDO1	1036
KREMEN2	79412	LOC728264	728264
CCR8	1237	LRRC70	100130733
GBP5	115362	ABCB5	340273
LIMD2	80774	CXCL12	6387
C16orf59	80178	C18orf34	374864
ESM1	11082	GPR81	27198
HIST1H2AM	8336	LRRC2	79442
PRAME	23532	SRPX	8406
ADAM8	101	FREM1	158326
JAKMIP1	152789	NECAB1	64168
PSRC1	84722	BAI3	577
S100P	6286	NDN	4692
ACAN	176	IL33	90865
LOC645323	645323	SVEP1	79987
GABRD	2563	SEMA3C	10512
TIGIT	201633	ACADSB	36
KCNK12	56660	LEPR	3953
NFKBIL2	4796	ASPA	443
DBNDD1	79007	EBF3	253738
CAMP	820	DNAH9	1770
KIF4B	285643	CD36	948
CXCL9	4283	CLEC3B	7123
TLCD1	116238	HOXA2	3199
P2RY6	5031	KLHL29	114818
COL22A1	169044	NAP1L2	4674
ZBED2	79413	DMD	1756
FUT7	2529	TMEM132C	92293
TMEM132A	54972	ADAMTS15	170689
FSD1	79187	ABCA6	23460
C2orf48	348738	LOC284276	284276
EN1	2019	MYOC	4653
RCOR2	283248	SLC2A4	6517
HAGHL	84264	TENC1	23371
WIT1	51352	DST	667
HIST2H2AA3	8337	TSHZ2	128553

PYCR1	5831	ADRB2	154
CAMK2N2	94032	NPR1	4881
SAMD14	201191	CYBRD1	79901
GABRQ	55879	SCN4A	6329
TMEM171	134285	SH3D19	152503
CDKN2A	1029	C4orf12	404201
TYMP	1890	STK32B	55351
MKRN3	7681	CASQ2	845
HIST1H3H	8357	CCDC158	339965
ZIC1	7545	AQP7	364
CLDN9	9080	TNS1	7145
TERT	7015	KIAA0408	9729
HORMAD1	84072	DUSP1	1843
CXorf61	203413	ZCCHC24	219654
HIST1H2BJ	8970	ADH1B	125
GABBR2	9568	HEPACAM	220296
PGLYRP4	57115	FABP4	2167
B3GNT4	79369	NXPH3	11248
CCL20	6364	ACACB	32
HOXB13	10481	AQP7P1	375719
PITX1	5307	ENPP2	5168
ICOS	29851	ALDH1A1	216
HIST1H3D	8351	DACT2	168002
UMODL1	89766	EBF1	1879
C17orf93	360205	ANGPTL7	10218
CAPS	828	MYOCD	93649
NXPH4	11247	SOCS2	8835
PCSK1N	27344	COL14A1	7373
RHOF	54509	C10orf116	10974
PRSS27	83886	TAL1	6886
CXCR2P1	3580	CCDC85A	114800
INA	9118	TIMP4	7079
GSDMC	56169	BMPER	168667
SLC15A1	6564	DBX2	440097
SLC26A9	115019	AKAP12	9590
HPDL	84842	IGF1	3479
FOXD1	2297	PCDHGA2	56113
KCNG1	3755	MOCS1	4337
ARTN	9048	EFHA2	286097
NPW	283869	CES1	1066
TMEM92	162461	C7orf63	79846
TMSB15A	11013	CXCL2	2920
SERPINB7	8710	PRICKLE2	166336
PRR7	80758	THRSP	7069
IVL	3713	GALNTL2	117248
NCCRP1	342897	C2orf40	84417
DLL3	10683	HSD17B13	345275

SBSN	374897	FAM149A	25854
CTSL2	1515	FAM13A	10144
PLAC2	257000	C1QTNF9B	387911
S100A8	6279	PLSCR4	57088
MAGEA4	4103	CCDC152	100129792
CASP14	23581	SYBU	55638
MAGEA6	4105	RGS22	26166
ORM2	5005	SCUBE2	57758
KRT16	3868	MRGPRF	116535
S100A7A	338324	FAM70A	55026
MAGEA3	4102	C4orf49	84709
ORM1	5004	PHYHIP	9796
		ADH1A	124
		PHYHD1	254295
		LAMA2	3908
		GLYAT	10249
		PTPRB	5787
		HPSE2	60495
		PER1	5187
		DCLK1	9201
		CFD	1675
		DARC	2532
		PDE8B	8622
		FGF1	2246
		KCNE1	3753
		LYVE1	10894
		SLC7A2	6542
		MEOX1	4222
		CLIC5	53405
		TGFBR2	7048
		RUNX1T1	862
		PLIN4	729359
		SLC40A1	30061
		LONRF2	164832
		IGF2	3481
		PLIN1	5346
		MASP1	5648
		ANGPT4	51378
		TBC1D9	23158
		ACSM5	54988
		THSD7B	80731
		HEPN1	641654
		ZNF423	23090
		KCNJ16	3773
		EGR1	1958
		MYOM1	8736
		DCN	1634

ERBB4	2066
SPATA18	132671
TUSC5	286753
GPD1	2819
TRPM3	80036
ITGA7	3679
C14orf132	56967
HSPB8	26353
DOC2B	8447
ITPR1	3708
LOC100190938	100190938
NLGN1	22871
MAP1LC3C	440738
MAST4	375449
LOC283392	283392
PI16	221476
LAMA3	3909
FHL1	2273
NBLA00301	79804
COL25A1	84570
NTN4	59277
MFAP4	4239
PCDHGA9	56107
ADHFE1	137872
PDE11A	50940
CAV2	858
DEFB132	400830
GLDN	342035
PPARG	5468
TEK	7010
FOSB	2354
LPHN3	23284
ADRA2A	150
WSCD2	9671
LEP	3952
NAALAD2	10003
HBB	3043
SLC47A2	146802
C1QTNF7	114905
TNNT3	7140
PRTG	283659
B3GALT1	8708
GNAL	2774
CLSTN2	64084
CNN1	1264
MEG3	55384
LUZP2	338645

TMEM232	642987
P2RY12	64805
CIDEC	63924
NNAT	4826
FLJ42875	440556
PLEKHH2	130271
C14orf180	400258
FMOD	2331
FAT4	79633
HSPB2	3316
EDA2R	60401
KLF4	9314
RYR3	6263
DLG2	1740
EBF2	64641
ADIPOQ	9370
PLCXD3	345557
PDZRN4	29951
LIPE	3991
GPR109B	8843
COL6A6	131873
CMA1	1215
C7orf58	79974
AMIGO2	347902
TLL1	7092
RELN	5649
VIT	5212
ABCC6	368
GRM7	2917
GSTM5	2949
ABCB1	5243
LRFN5	145581
NPY2R	4887
FGF2	2247
FZD4	8322
FOS	2353
ADAMTS18	170692
ASXL3	80816
USP44	84101
EGR3	1960
AOX1	316
PREX2	80243
CPS1	1373
CES4	51716
GPAM	57678
RSPO3	84870
COL4A6	1288

MGLL	11343
SORBS1	10580
RDH5	5959
DYNC2H1	79659
RIMBP2	23504
PCDHGA3	56112
CCDC68	80323
CCDC36	339834
LOC283174	283174
CLDN19	149461
GPM6A	2823
RBP4	5950
AQPEP	206338
GPR44	11251
PTH1R	5745
MUSTN1	389125
CYP2A6	1548
ANK2	287
LPL	4023
RXRG	6258
ECM2	1842
MRAP	56246
SYNM	23336
VIPR2	7434
ANGPTL2	23452
PCK1	5105
PRDM16	63976
MAOB	4129
ACVR1C	130399
CTSG	1511
DMGDH	29958
PFKFB1	5207
GABRA2	2555
GFAP	2670
ACER2	340485
C6orf155	79940
CIDEA	1149
ODF3L1	161753
ANKRD30A	91074
KIAA1683	80726
PLP1	5354
BMP5	653
CPB1	1360
ASAM	79827
C9orf68	55064
WLS	79971
NBEA	26960

KCND3	3752
PAPPA2	60676
PPP1R1A	5502
FRMD1	79981
PCSK5	5125
MYLK	4638
PVRL3	25945
ST6GALNAC1	55808
AR	367
KLHL13	90293
TMEM100	55273
TBX15	6913
SCN11A	11280
ZNF483	158399
EMX2OS	196047
STEAP4	79689
MT1M	4499
SELP	6403
ACADL	33
TMEM195	392636
LGALS12	85329
HMGCLL1	54511
PTPRT	11122
ADH4	127
GPR133	283383
GPR109A	338442
C8orf34	116328
LRP1B	53353
AFF3	3899
ABCC6P1	653190
SLIT2	9353
TFF3	7033
C1orf95	375057
ODZ1	10178
OMD	4958
CNTN3	5067
HAS3	3038
SEMA3D	223117
KCNMA1	3778
RAB3C	115827
RGS6	9628
BHMT2	23743
PRRT4	401399
CITED1	4435
ISM1	140862
LIN7A	8825
SERPINA5	5104

SULT1C4	27233
BMP3	651
TMEM35	59353
KY	339855
DSCAML1	57453
C10orf108	414235
CADM2	253559
IL6ST	3572
GSTM2	2946
SLC25A18	83733
INPP4B	8821
KCNB1	3745
ALX4	60529
PRRG3	79057
XPNPEP2	7512
ERVFRDE1	405754
ODZ2	57451
MT1A	4489
ZNF385B	151126
SIGLECP3	284367
HTR2A	3356
CALN1	83698
NCAM2	4685
FCER1A	2205
PLXNA4	91584
ABCC8	6833
CACNA2D1	781
GPR17	2840
CDKL2	8999
KCNA6	3742
SEMA3E	9723
CYP4X1	260293
RBM24	221662
APOD	347
GREM2	64388
SAA1	6288
FOXP2	93986
SHISA6	388336
KCNA1	3736
LPPR1	54886
IQSEC3	440073
CYP4A11	1579
ANKRD43	134548
MS4A2	2206
PCOLCE2	26577
MOSC1	64757
D4S234E	27065

PENK	5179
FMO5	2330
NCRNA00093	100188954
CA12	771
HDC	3067
RANBP3L	202151
CLIC6	54102
RNF150	57484
SCN2A	6326
CADM3	57863
C3orf15	89876
CPA3	1359
PTHLH	5744
CAPN8	388743
FCN2	2220

Supplementary Table 2a. Up regulated genes in 70% of TNBC patients

Up regulated genes		Stage I			Stage II			Stage III		
GeneName	GeneID	FC	FDR	frequency	FC	FDR	frequency	FC	FDR	frequency
IBSP	3381	6.031	1.91E-56	1	5.3176	6.86E-64	0.96154	3.9548	2.37E-24	0.76
COL10A1	1300	6.6188	8.91E-47	1	5.2186	4.41E-48	0.875	5.0457	4.36E-28	0.8
MMP1	4312	7.7954	4.46E-38	1	7.0939	1.17E-58	0.88462	5.7968	9.64E-21	0.64
MMP11	4320	5.8746	2.35E-48	0.965517	4.5941	1.74E-55	0.82692	4.6024	7.28E-31	0.76
PLK1	5347	4.3148	1.57E-37	0.965517	4.8809	5.37E-88	0.99038	4.3848	1.01E-33	0.96
COL11A1	1301	6.4191	4.06E-36	0.965517	5.4609	8.41E-50	0.86538	4.2004	7.55E-16	0.72
CEP55	55165	4.3062	1.71E-34	0.965517	4.7751	2.04E-79	0.96154	4.0902	6.50E-28	0.88
UBE2C	11065	5.0656	1.98E-33	0.965517	5.6822	1.17E-80	0.99038	5.2466	6.61E-30	0.96
ANLN	54443	4.3108	3.92E-33	0.965517	4.5994	1.99E-71	0.95192	4.4102	7.11E-29	0.92
KIF4A	24137	4.917	1.52E-32	0.965517	5.4646	6.14E-80	0.99038	4.9905	9.04E-29	0.96
CDC20	991	4.5467	1.16E-31	0.965517	5.2862	1.82E-80	0.99038	4.6868	9.68E-28	0.88
MYBL2	4605	4.8159	1.42E-31	0.965517	5.4873	3.57E-79	0.99038	4.7776	2.81E-27	0.96
MELK	9833	4.8109	2.65E-31	0.965517	5.3026	1.90E-74	0.96154	4.7805	2.88E-27	0.92
CCNB2	9133	4.1586	6.86E-31	0.965517	4.6725	3.75E-75	0.96154	4.1902	3.76E-27	0.96
BUB1B	701	3.6759	2.10E-25	0.965517	4.1822	4.53E-64	0.94231	3.7637	8.40E-22	0.84
TPX2	22974	4.2399	1.09E-40	0.931034	4.7639	3.45E-88	0.98077	4.0418	3.22E-33	0.96
HJURP	55355	4.645	5.45E-37	0.931034	4.9402	2.71E-83	0.98077	4.5065	1.82E-30	0.92
CDCA3	83461	3.8804	1.79E-36	0.931034	4.3827	3.62E-81	0.97115	3.7862	8.15E-31	0.84
SKA1	220134	3.8639	7.36E-36	0.931034	4.4977	3.74E-80	0.97115	4.0468	3.10E-32	0.88
CENPA	1058	4.2475	1.91E-34	0.931034	4.8678	4.55E-81	0.97115	4.3819	7.82E-30	0.96
FOXM1	2305	4.396	3.45E-34	0.931034	5.1082	1.36E-80	0.99038	4.1454	8.07E-28	0.92
MMP13	4322	6.8793	5.88E-34	0.931034	5.6299	1.03E-41	0.77885	4.9854	1.59E-15	0.64
NDC80	10403	4.3209	3.58E-33	0.931034	4.7498	8.88E-75	0.98077	4.2725	1.43E-27	0.92
PPAPDC1A	196051	5.3643	8.35E-33	0.931034	4.5884	1.97E-48	0.81731	4.6051	1.17E-21	0.76
IQGAP3	128239	4.7561	1.45E-32	0.931034	5.1631	6.38E-75	0.98077	4.5343	6.18E-27	0.88
TROAP	10024	4.5445	1.16E-31	0.931034	5.0753	3.05E-80	0.98077	4.6022	3.52E-28	0.92

DEPDC1B	55789	3.926	7.23E-31	0.931034	4.231	4.59E-70	0.96154	3.5244	7.16E-23	0.88
NEK2	4751	5.1172	7.68E-31	0.931034	5.4284	1.03E-73	0.97115	4.8201	3.89E-25	0.96
KIF2C	11004	4.2497	9.14E-31	0.931034	5.0083	1.94E-80	0.98077	4.4117	3.84E-28	0.92
SPC24	147841	3.6928	1.00E-30	0.931034	4.4781	3.24E-75	0.96154	3.7697	2.31E-26	0.84
CDCA2	157313	4.1012	1.18E-30	0.931034	4.594	6.68E-69	0.95192	3.7745	7.37E-25	0.92
NUSAP1	51203	3.6017	2.93E-30	0.931034	3.8189	5.85E-67	0.95192	3.5581	5.31E-25	0.84
ASPM	259266	4.8313	1.41E-29	0.931034	4.9388	3.80E-65	0.95192	4.431	1.61E-22	0.88
EXO1	9156	4.3634	1.70E-29	0.931034	5.0406	2.31E-75	0.97115	4.1189	2.47E-24	0.96
KIF20A	10112	4.6292	2.38E-29	0.931034	4.9264	5.99E-71	0.98077	4.5322	4.86E-24	0.88
SPC25	57405	3.6733	6.20E-28	0.931034	4.0629	1.05E-68	0.97115	3.6768	1.63E-24	0.88
DLGAP5	9787	4.5353	2.54E-27	0.931034	4.9712	7.59E-69	0.98077	4.5859	7.50E-24	0.96
MKI67	4288	4.0196	3.90E-27	0.931034	4.3064	2.33E-63	0.92308	3.7101	2.70E-21	0.8
PKMYT1	9088	4.8373	1.68E-25	0.931034	5.5236	2.01E-70	0.98077	5.1633	3.36E-24	0.92
CDC45	8318	4.4403	6.12E-25	0.931034	4.9393	5.08E-67	0.99038	4.3496	2.35E-21	0.92
AURKA	6790	3.2813	9.27E-42	0.896552	3.8384	8.94E-91	0.96154	3.4456	2.68E-38	0.88
FAM72B	653820	3.3422	8.26E-39	0.896552	3.6947	2.06E-73	0.93269	3.1724	3.20E-31	0.84
TACC3	10460	3.0361	5.45E-37	0.896552	3.1091	4.44E-79	0.89423	2.871	4.06E-29	0.8
CDCA8	55143	3.5923	1.17E-36	0.896552	4.2442	3.49E-84	0.97115	3.6886	6.66E-32	0.84
GTSE1	51512	3.9429	9.71E-36	0.896552	4.3215	1.97E-79	0.99038	3.8764	2.57E-29	0.88
FAM54A	113115	3.4681	1.46E-35	0.896552	4.1189	8.58E-84	0.96154	3.376	1.22E-31	0.88
PTTG1	9232	3.7498	5.90E-35	0.896552	4.2291	3.12E-79	0.97115	3.9991	6.70E-32	0.88
NUF2	83540	4.7154	1.42E-34	0.896552	5.1967	1.15E-79	0.97115	4.6397	1.67E-29	0.92
CCNA2	890	3.5168	1.90E-34	0.896552	3.943	2.33E-75	0.96154	3.4241	1.05E-28	0.88
FAM83D	81610	4.1261	3.56E-34	0.896552	4.5929	5.19E-79	0.96154	4.0431	6.65E-29	0.84
CCL11	6356	4.2577	1.27E-33	0.896552	3.5497	2.32E-39	0.78846	3.8224	6.71E-24	0.84
CDCA5	113130	3.4833	3.02E-33	0.896552	4.1637	1.61E-84	0.97115	3.621	2.40E-30	0.88
DEPDC1	55635	4.1551	2.40E-32	0.896552	4.503	9.70E-71	0.94231	4.0006	5.63E-27	0.92
GPR19	2842	3.1252	7.18E-32	0.896552	3.0755	5.93E-53	0.78846	2.067	2.05E-15	0.52
KIF14	9928	4.6242	1.27E-31	0.896552	4.9856	5.05E-71	0.96154	4.4073	7.55E-26	0.92

GJB2	2706	4.1914	1.79E-31	0.896552	3.757	6.62E-43	0.77885	3.8815	4.48E-21	0.64
KIFC1	3833	4.0126	5.98E-31	0.896552	4.4064	2.97E-74	0.98077	3.7784	6.47E-25	0.88
SKA3	221150	3.8952	9.14E-31	0.896552	4.3031	4.98E-70	0.97115	3.8633	6.15E-27	0.92
ORC6L	23594	3.3156	9.59E-31	0.896552	3.8954	2.33E-75	0.95192	3.2603	1.67E-25	0.8
CKAP2L	150468	4.0383	1.83E-30	0.896552	4.3406	5.80E-66	0.95192	3.7222	6.47E-24	0.84
OIP5	11339	3.1995	2.53E-30	0.896552	3.6391	6.28E-72	0.94231	3.3201	3.89E-27	0.8
TDO2	6999	4.9274	3.53E-30	0.896552	4.5376	8.22E-46	0.82692	4.9629	7.37E-25	0.84
SGOL1	151648	4.0492	3.54E-30	0.896552	4.5004	4.04E-71	0.95192	3.606	1.29E-22	0.84
OR2B6	26212	2.7109	6.36E-30	0.896552	2.7493	3.59E-37	0.84615	2.4356	1.39E-20	0.76
NCAPH	23397	3.4675	1.11E-29	0.896552	3.9853	1.94E-72	0.94231	3.3479	4.13E-24	0.84
RAD54L	8438	3.8948	1.67E-29	0.896552	4.5998	2.66E-77	0.98077	3.9066	3.97E-26	0.88
AURKB	9212	4.6005	7.37E-29	0.896552	5.3122	5.80E-76	0.98077	4.657	1.70E-25	0.92
CDC25C	995	4.3701	1.20E-28	0.896552	4.74	4.64E-72	0.98077	4.2332	8.37E-24	0.88
KIF18B	146909	4.6765	1.47E-28	0.896552	5.2683	3.74E-74	0.98077	4.4834	8.86E-24	0.88
SHCBP1	79801	3.2467	2.60E-28	0.896552	3.613	6.70E-67	0.92308	3.2227	9.01E-24	0.84
HMMR	3161	4.0007	2.76E-28	0.896552	4.1638	2.57E-63	0.93269	3.7914	9.70E-23	0.92
NEIL3	55247	4.1549	3.35E-28	0.896552	4.6291	1.89E-68	0.96154	4.2708	3.63E-25	0.92
ASF1B	55723	3.2664	9.28E-28	0.896552	3.7224	7.72E-71	0.97115	3.2457	5.57E-24	0.88
TTK	7272	3.9256	5.84E-27	0.896552	4.4482	8.00E-66	0.95192	3.7135	2.51E-22	0.88
RRM2	6241	4.0892	1.28E-26	0.896552	4.4628	1.88E-67	0.98077	4.2187	1.21E-23	0.96
PBK	55872	3.911	7.42E-24	0.896552	4.5839	4.81E-64	0.94231	4.2364	2.40E-23	0.96
EPR1	8475	4.3048	9.58E-22	0.896552	4.9197	1.86E-50	0.94231	3.707	1.42E-12	0.8
CDK1	983	3.4631	4.65E-36	0.862069	3.8592	9.72E-81	0.96154	3.5686	5.31E-32	0.88
UBE2T	29089	3.5579	1.24E-35	0.862069	4.0179	1.17E-80	0.95192	3.5928	1.58E-32	0.92
EPYC	1833	4.8591	6.16E-34	0.862069	4.1089	2.21E-37	0.76923	2.763	1.04E-13	0.44
CENPE	1062	3.1648	6.92E-33	0.862069	3.5997	9.98E-68	0.92308	3.1829	2.07E-28	0.84
FAM72A	729533	3.6037	1.44E-32	0.862069	3.7934	5.80E-61	0.92308	3.1965	2.98E-23	0.76
PIF1	80119	3.1349	2.54E-32	0.862069	3.652	3.99E-69	0.90385	3.1572	1.48E-26	0.8
NCAPG	64151	3.7647	6.78E-32	0.862069	4.088	2.55E-73	0.97115	3.6266	4.64E-26	0.88

BUB1	699	4.4248	7.08E-32	0.862069	4.9008	5.15E-76	0.98077	4.1805	5.26E-26	0.92
CDKN3	1033	3.4839	1.10E-31	0.862069	3.7204	8.34E-71	0.95192	3.7707	2.67E-31	0.96
CENPF	1063	3.9647	2.10E-31	0.862069	4.1837	1.00E-68	0.95192	3.5445	1.33E-23	0.88
KIF15	56992	3.6128	3.79E-30	0.862069	3.9787	3.61E-69	0.93269	3.3911	1.24E-24	0.76
ZNF695	57116	3.8737	4.67E-29	0.862069	4.5797	1.49E-73	0.96154	3.4444	1.31E-21	0.8
ERCC6L	54821	3.1615	1.74E-27	0.862069	3.5492	9.90E-65	0.91346	3.1742	1.82E-24	0.88
FAM64A	54478	4.2453	2.70E-27	0.862069	4.7032	2.29E-68	0.97115	4.0186	2.15E-22	0.76
IL4I1	259307	3.2623	1.46E-26	0.862069	3.2779	1.36E-51	0.79808	2.812	2.29E-18	0.68
VGF	7425	3.9457	1.89E-26	0.862069	4.037	7.92E-47	0.82692	4.3669	3.31E-26	0.84
KIF23	9493	3.1218	5.44E-26	0.862069	3.6328	2.75E-63	0.91346	3.2239	7.32E-24	0.84
BIRC5	332	4.6273	1.07E-25	0.862069	5.4353	9.55E-71	0.96154	4.7625	2.03E-23	0.88
TOP2A	7153	4.1427	5.21E-25	0.862069	4.4314	4.49E-60	0.95192	3.707	1.83E-18	0.76
KIAA0101	9768	3.7211	1.51E-23	0.862069	4.0702	2.40E-59	0.93269	3.7709	1.14E-21	0.84
MND1	84057	3.6982	2.15E-23	0.862069	4.2054	1.78E-55	0.92308	3.7546	1.88E-20	0.84
UHRF1	29128	3.9969	3.83E-23	0.862069	4.4563	7.83E-61	0.96154	3.9793	1.43E-19	0.88
SLC7A5	8140	3.1761	2.80E-21	0.862069	3.8467	7.59E-56	0.93269	3.504	2.57E-20	0.76
CENPM	79019	3.4973	2.45E-20	0.862069	3.83	1.47E-54	0.92308	3.7203	7.69E-19	0.84
PRC1	9055	3.0472	3.38E-38	0.827586	3.2974	2.23E-73	0.89423	2.7335	3.29E-30	0.76
GPRIN1	114787	2.9259	4.44E-32	0.827586	3.221	1.20E-66	0.85577	2.9027	6.18E-27	0.68
SPAG5	10615	2.8442	8.50E-32	0.827586	3.3239	1.46E-73	0.93269	2.7709	1.49E-27	0.76
RAD51	5888	2.9826	2.10E-31	0.827586	3.4526	1.14E-75	0.93269	3.1584	1.51E-29	0.88
CKS2	1164	2.7186	2.48E-28	0.827586	3.2743	3.10E-66	0.91346	3.0271	2.09E-28	0.8
CLEC5A	23601	3.7148	1.14E-27	0.827586	3.401	9.95E-44	0.75	2.4313	4.50E-13	0.48
PTTG3P	26255	2.4272	1.92E-27	0.827586	2.6532	1.55E-53	0.90385	2.4866	2.82E-25	0.84
TNFRSF9	3604	3.7602	8.73E-27	0.827586	3.2441	1.04E-34	0.71154	2.5909	4.25E-14	0.6
CNIH2	254263	3.9264	1.25E-26	0.827586	4.041	3.07E-46	0.78846	3.7624	2.64E-23	0.8
LOC339674	339674	2.7855	2.39E-25	0.827586	3.0474	1.70E-49	0.80769	2.8018	6.87E-22	0.8
CLSPN	63967	3.665	1.02E-23	0.827586	4.0335	6.38E-53	0.86538	3.3676	2.22E-19	0.8
ADAMDEC1	27299	4.8205	8.77E-20	0.827586	4.3561	1.08E-32	0.67308	3.7768	5.31E-12	0.52

FAM111B	374393	3.5203	1.15E-19	0.827586	3.6499	2.10E-46	0.78846	3.2813	5.55E-15	0.72
CCNE1	898	3.2257	4.02E-37	0.793103	4.186	2.80E-78	0.91346	3.5942	8.02E-37	0.92
FAM72D	728833	3.5015	1.45E-33	0.793103	3.5992	3.08E-56	0.84615	3.2325	5.66E-27	0.76
SCT	6343	3.2643	2.76E-32	0.793103	2.3953	8.91E-29	0.57692	2.5788	1.38E-18	0.6
MCM10	55388	3.9637	2.75E-30	0.793103	4.6242	4.59E-73	0.96154	3.6911	2.91E-25	0.88
EZH2	2146	3.0845	4.25E-29	0.793103	3.3082	8.29E-66	0.89423	2.7981	2.80E-24	0.76
E2F1	1869	2.8447	1.10E-28	0.793103	3.4874	1.29E-76	0.91346	3.0188	8.30E-28	0.8
TUBB3	10381	3.6367	2.79E-28	0.793103	3.8851	5.69E-54	0.82692	3.8485	8.25E-24	0.76
NMU	10874	5.0037	2.86E-28	0.793103	5.1731	8.85E-42	0.79808	4.175	1.03E-18	0.64
TK1	7083	3.3432	9.21E-27	0.793103	3.8687	8.15E-71	0.95192	3.4179	1.41E-24	0.84
BLM	641	2.6206	2.37E-26	0.793103	2.9901	6.00E-60	0.82692	2.2612	1.49E-19	0.6
LMNB1	4001	2.8816	2.48E-26	0.793103	3.1	2.21E-61	0.81731	2.6762	1.53E-20	0.72
AIM1L	55057	3.616	3.66E-26	0.793103	3.9049	6.06E-47	0.83654	4.2097	9.02E-25	0.84
DTL	51514	3.3125	4.55E-25	0.793103	3.5868	4.01E-61	0.92308	3.0125	3.06E-19	0.68
E2F8	79733	4.1244	5.02E-25	0.793103	4.5115	1.38E-61	0.86538	3.7423	1.07E-19	0.72
ACTL8	81569	4.663	1.19E-24	0.793103	5.613	1.14E-37	0.85577	4.5935	7.09E-22	0.8
FANCA	2175	3.1812	2.42E-24	0.793103	3.4903	3.75E-59	0.875	3.0212	1.62E-19	0.76
TCAM1P	146771	4.0196	2.90E-23	0.793103	3.8318	5.76E-33	0.68269	NA	NA	NA
RECQL4	9401	3.0024	5.40E-23	0.793103	3.693	2.13E-64	0.90385	3.6415	1.77E-25	0.84
E2F2	1870	3.2279	8.19E-23	0.793103	3.6879	1.19E-58	0.91346	3.2132	1.39E-20	0.8
KIAA1199	57214	3.7122	4.96E-22	0.793103	3.4908	1.12E-31	0.70192	3.3347	5.15E-16	0.56
CASC5	57082	3.2303	6.76E-22	0.793103	3.3683	4.15E-41	0.76923	2.9347	3.11E-15	0.72
ESCO2	157570	3.088	1.85E-21	0.793103	3.4331	5.50E-52	0.79808	2.8931	2.07E-16	0.64
CTHRC1	115908	2.821	6.46E-21	0.793103	2.2569	1.56E-30	0.45192	2.5913	4.00E-16	0.68
S100P	6286	5.1003	1.09E-19	0.793103	4.3259	2.02E-24	0.64423	4.6156	2.09E-14	0.72
C1orf135	79000	2.5966	1.20E-33	0.758621	3.1878	8.55E-78	0.90385	2.5062	4.21E-28	0.76
MFAP2	4237	2.8828	4.25E-32	0.758621	2.6821	9.56E-41	0.70192	2.8125	4.78E-25	0.72
RAD51AP1	10635	2.8564	9.38E-29	0.758621	3.2941	5.53E-62	0.86538	2.4127	6.87E-20	0.68
C15orf42	90381	2.7773	7.94E-28	0.758621	3.3142	1.33E-66	0.89423	2.5889	1.43E-22	0.72

KIF11	3832	2.8833	1.57E-27	0.758621	3.2926	2.03E-65	0.92308	2.7296	2.30E-22	0.76
HSD17B6	8630	2.9287	7.56E-27	0.758621	2.6689	1.80E-42	0.70192	2.4771	5.88E-18	0.6
CST1	1469	6.1509	5.36E-26	0.758621	4.9291	9.00E-30	0.66346	3.7862	1.21E-11	0.36
KIF18A	81930	3.0161	1.61E-24	0.758621	3.5031	4.07E-62	0.90385	2.9171	1.50E-20	0.64
LHX2	9355	3.2786	5.88E-24	0.758621	3.6347	1.41E-32	0.73077	3.4112	1.03E-22	0.72
CDC6	990	2.7998	6.06E-24	0.758621	3.3768	1.05E-66	0.92308	2.8856	1.53E-22	0.84
HIST2H3C	126961	2.4904	3.03E-23	0.758621	2.3956	7.04E-38	0.70192	2.7483	4.15E-26	0.8
ESPL1	9700	3.3405	3.54E-22	0.758621	3.8476	2.54E-63	0.94231	3.198	2.78E-18	0.72
E2F7	144455	3.5166	1.10E-21	0.758621	3.6817	9.70E-48	0.80769	3.0554	4.54E-15	0.52
ENTHD1	150350	2.88	3.14E-21	0.758621	2.311	1.29E-30	0.61538	NA	NA	NA
ADAMTS14	140766	3.5369	3.24E-21	0.758621	3.2517	1.30E-41	0.60577	3.0757	7.28E-15	0.56
CST4	1472	2.5384	4.66E-21	0.758621	NA	NA	NA	NA	NA	NA
WBSCR28	135886	2.6484	2.74E-20	0.758621	2.3214	2.06E-25	0.67308	NA	NA	NA
HIST1H2AM	8336	2.983	3.43E-20	0.758621	3.0827	6.64E-43	0.75962	3.514	8.63E-25	0.88
CDT1	81620	3.3231	1.66E-19	0.758621	3.9228	4.35E-57	0.93269	3.5204	9.69E-19	0.8
CLEC6A	93978	2.0693	7.65E-18	0.758621	2.0957	1.18E-23	0.65385	NA	NA	NA
CBS	875	2.7366	4.52E-17	0.758621	3.3062	2.67E-43	0.78846	NA	NA	NA
ZIC1	7545	4.0664	1.33E-15	0.758621	4.1791	1.61E-28	0.78846	2.1718	4.77E-05	0.52
CCNB1	891	2.5727	1.24E-33	0.724138	3.0603	7.78E-77	0.875	2.9043	1.39E-33	0.84
UBE2S	27338	2.6835	3.28E-31	0.724138	3.3311	1.91E-76	0.89423	3.1006	3.07E-31	0.8
TRIP13	9319	2.7118	1.86E-28	0.724138	3.5018	1.77E-73	0.92308	2.8363	4.36E-28	0.76
CBX2	84733	2.6854	4.38E-28	0.724138	3.7014	1.26E-77	0.90385	3.2018	1.66E-35	0.8
CXCL10	3627	4.6167	1.75E-27	0.724138	4.222	4.52E-42	0.81731	3.2932	1.65E-17	0.68
CA9	768	5.5562	5.02E-27	0.724138	6.1719	1.35E-45	0.82692	5.8261	1.53E-25	0.76
SH2D2A	9047	2.858	9.84E-26	0.724138	2.8777	4.27E-51	0.74038	2.6264	5.45E-21	0.76
TYMS	7298	2.7367	2.61E-25	0.724138	3.1345	3.46E-56	0.80769	2.5466	5.69E-20	0.68
POLQ	10721	3.2282	4.76E-25	0.724138	3.6646	2.73E-63	0.95192	2.9287	3.85E-19	0.8
HIST1H2BO	8348	2.8169	1.99E-23	0.724138	3.0169	2.83E-44	0.85577	2.9576	6.20E-26	0.84
CCL7	6354	3.5255	5.98E-22	0.724138	3.935	4.34E-38	0.75	2.2056	1.32E-10	0.52

CCR8	1237	3.2082	5.49E-21	0.724138	2.7915	3.96E-30	0.66346	2.5008	3.27E-13	0.6
ORC1L	4998	2.9902	1.12E-20	0.724138	3.8125	3.27E-64	0.94231	3.1133	1.22E-19	0.76
PRAME	23532	5.7185	3.92E-20	0.724138	6.5928	5.04E-45	0.82692	4.6327	4.60E-12	0.64
ETV7	51513	2.8083	9.85E-20	0.724138	2.7618	1.44E-35	0.61538	NA	NA	NA
LRRC15	131578	3.3665	5.53E-19	0.724138	2.4345	2.14E-20	0.50962	2.2353	1.82E-08	0.4
HAPLN1	1404	4.4536	7.75E-19	0.724138	4.1224	2.42E-32	0.71154	3.6656	1.53E-14	0.6
KIF26B	55083	3.3308	7.94E-19	0.724138	2.5992	7.30E-25	0.58654	2.9402	6.23E-15	0.68
LOC645323	645323	3.1471	8.20E-19	0.724138	3.372	9.37E-30	0.71154	2.4996	3.77E-12	0.52
CHST6	4166	2.8951	2.84E-18	0.724138	3.133	1.68E-31	0.65385	2.1324	1.31E-09	0.44
RAET1K	646024	2.3682	2.85E-18	0.724138	2.4689	2.04E-32	0.66346	2.2803	3.18E-15	0.72
COL9A3	1299	3.9741	3.07E-16	0.724138	3.2217	4.25E-20	0.56731	2.5079	1.62E-07	0.44
MKRN3	7681	3.9641	5.36E-16	0.724138	4.1122	4.38E-31	0.76923	3.0401	1.36E-10	0.6
COMP	1311	4.1757	1.17E-15	0.724138	3.4435	1.97E-19	0.63462	3.5684	1.39E-10	0.64
RLTPR	146206	3.0851	1.42E-15	0.724138	3.104	1.59E-32	0.67308	2.3997	2.79E-11	0.52
PAQR4	124222	2.5002	2.61E-32	0.689655	2.7456	2.04E-66	0.75962	2.6136	2.92E-26	0.76
LRP8	7804	2.4823	1.58E-31	0.689655	2.8698	2.09E-62	0.80769	2.2571	2.63E-20	0.56
ARHGAP11A	9824	2.4588	3.93E-29	0.689655	2.9124	6.85E-64	0.84615	2.431	1.62E-25	0.64
CCNE2	9134	3.1269	8.33E-28	0.689655	3.1245	7.00E-50	0.75	2.9945	4.39E-24	0.8
GINS1	9837	2.7084	4.09E-26	0.689655	3.0549	2.16E-62	0.84615	2.7884	1.21E-25	0.8
ULBP1	80329	3.1868	2.21E-25	0.689655	3.8879	1.55E-55	0.86538	3.0304	1.63E-19	0.6
CXCL11	6373	4.5712	5.58E-25	0.689655	4.0685	1.62E-40	0.78846	3.2669	2.79E-15	0.64
TFR2	7036	3.3922	1.31E-22	0.689655	3.6623	4.15E-43	0.77885	2.9314	8.05E-17	0.6
A2ML1	144568	4.4862	3.18E-21	0.689655	4.283	1.71E-26	0.70192	3.7598	1.24E-13	0.72
KIF4B	285643	2.1742	3.16E-18	0.689655	2.7541	2.35E-55	0.83654	2.2715	9.36E-19	0.68
GAL	51083	3.2804	4.20E-16	0.689655	3.9515	3.04E-35	0.75	3.0476	2.63E-12	0.6
PITX1	5307	3.6808	5.89E-14	0.689655	4.535	8.43E-45	0.875	4.7661	2.21E-19	0.8
SOX11	6664	3.9797	6.87E-14	0.689655	4.4043	2.76E-32	0.75	3.4747	5.63E-10	0.56
MEX3A	92312	2.887	2.90E-12	0.689655	3.3617	3.09E-36	0.79808	2.3166	5.07E-08	0.52
C1orf106	55765	2.965	6.47E-09	0.689655	3.1932	1.90E-25	0.71154	2.9775	2.63E-08	0.56

HMGB3	3149	2.4395	1.33E-32	0.655172	2.8378	3.36E-74	0.85577	2.7745	6.61E-36	0.92
STMN1	3925	2.384	3.44E-32	0.655172	2.6682	6.50E-67	0.75	2.2452	4.01E-29	0.6
MAD2L1	4085	2.3594	7.61E-28	0.655172	2.8858	5.79E-64	0.80769	2.3473	6.20E-26	0.64
DIAPH3	81624	3.0639	2.52E-27	0.655172	3.4708	8.62E-59	0.88462	2.7081	2.66E-21	0.68
CENPI	2491	2.8511	1.17E-25	0.655172	3.3056	9.84E-55	0.875	2.9807	4.38E-24	0.8
CDC25A	993	2.833	6.71E-25	0.655172	3.5098	1.54E-66	0.89423	2.9885	1.94E-24	0.84
ZWINT	11130	2.5818	6.19E-23	0.655172	2.9755	2.37E-60	0.83654	2.7043	3.65E-22	0.72
GSG2	83903	2.3852	1.26E-22	0.655172	2.9446	1.38E-59	0.79808	2.2381	6.23E-19	0.64
DMRT1	1761	2.3187	5.86E-18	0.655172	3.1056	2.96E-29	0.73077	NA	NA	NA
C2orf48	348738	2.7337	1.50E-17	0.655172	3.4369	4.03E-52	0.83654	2.3074	8.37E-15	0.6
STRA8	346673	2.0481	3.81E-17	0.655172	2.1528	1.24E-21	0.70192	NA	NA	NA
TLX1NB	1E+08	2.3983	4.39E-16	0.655172	2.9331	3.76E-27	0.76923	NA	NA	NA
OTX1	5013	3.4273	1.59E-15	0.655172	3.7295	8.35E-39	0.75	3.019	7.27E-12	0.52
ZIC4	84107	2.7993	1.19E-12	0.655172	2.945	3.09E-24	0.70192	NA	NA	NA
KPNA2	3838	2.1905	6.39E-34	0.62069	2.6487	1.70E-70	0.78846	2.1087	8.54E-29	0.6
WDR62	284403	3.7009	1.85E-28	0.62069	4.0506	3.53E-71	0.81731	3.5741	2.47E-24	0.72
KIAA1524	57650	2.2542	2.92E-25	0.62069	2.6464	1.39E-56	0.71154	NA	NA	NA
CIT	11113	2.3232	3.14E-24	0.62069	2.645	7.00E-59	0.75962	2.0644	2.67E-18	0.4
C16orf75	116028	2.7406	5.38E-24	0.62069	3.0609	4.34E-59	0.83654	2.6027	4.69E-19	0.56
CDC7	8317	2.3433	1.35E-21	0.62069	2.75	5.55E-54	0.72115	2.1424	4.36E-17	0.48
TLX1	3195	4.276	3.90E-21	0.62069	5.4103	8.17E-37	0.78846	3.2221	2.28E-12	0.52
C16orf59	80178	2.7817	1.25E-20	0.62069	3.5597	5.13E-65	0.94231	3.1454	6.53E-23	0.76
CCDC150	284992	2.2251	9.77E-20	0.62069	2.7755	7.39E-51	0.73077	2.0213	2.59E-15	0.56
PRR11	55771	3.0226	8.99E-19	0.62069	3.3769	5.49E-37	0.80769	2.3978	6.98E-11	0.56
KCNK12	56660	2.6177	1.49E-18	0.62069	2.4962	3.79E-37	0.70192	2.7898	3.64E-17	0.72
CDKN2A	1029	2.999	2.64E-16	0.62069	3.597	2.70E-29	0.71154	2.7474	8.09E-13	0.56
HORMAD1	84072	4.4563	2.61E-15	0.62069	5.5917	2.88E-32	0.73077	3.4521	1.56E-09	0.48
TLX3	30012	2.2706	2.57E-13	0.62069	3.1764	8.43E-26	0.75962	NA	NA	NA
CDCA7	83879	2.3656	3.35E-13	0.62069	3.1264	6.35E-48	0.81731	2.4869	1.16E-13	0.56

STIL	6491	2.6033	2.86E-26	0.586207	2.9991	3.88E-59	0.81731	2.3565	4.20E-21	0.72
ECE2	9718	2.3059	2.54E-25	0.586207	2.8963	7.80E-67	0.81731	2.6512	1.06E-28	0.72
EME1	146956	2.2939	6.45E-22	0.586207	3.0229	9.61E-68	0.85577	2.1737	8.06E-20	0.56
KREMEN2	79412	3.3451	5.17E-21	0.586207	3.7957	2.01E-42	0.74038	3.9127	1.32E-22	0.76
RDM1	201299	2.5923	8.09E-15	0.586207	3.4356	1.18E-46	0.84615	2.1863	2.57E-11	0.56
C18orf56	494514	2.2182	1.63E-14	0.586207	2.7897	1.06E-40	0.73077	2.3988	6.25E-16	0.6
HMGA1	3159	2.2129	6.94E-24	0.551724	2.5938	4.91E-57	0.71154	2.1747	2.55E-20	0.4
MFSD2B	388931	2.3537	2.99E-21	0.551724	2.794	3.32E-53	0.75	2.3744	4.20E-18	0.52
PSRC1	84722	2.3077	8.78E-20	0.551724	2.7786	8.90E-56	0.77885	2.4787	5.78E-20	0.68
RCOR2	283248	2.4176	3.28E-17	0.551724	3.4824	4.05E-50	0.76923	2.5678	5.11E-15	0.6
C17orf93	360205	2.1441	2.27E-13	0.551724	2.809	9.95E-25	0.71154	2.3393	1.66E-16	0.6
HN1	51155	2.1996	5.47E-28	0.517241	2.73	5.50E-68	0.76923	2.2561	2.08E-25	0.6
NFKBIL2	4796	2.2644	1.74E-18	0.517241	2.8499	2.29E-53	0.75	2.7379	1.34E-21	0.72
B3GNT4	79369	2.387	1.18E-14	0.482759	2.764	1.07E-38	0.70192	3.0138	1.07E-19	0.76
HPDL	84842	2.4721	1.31E-11	0.482759	3.3782	1.17E-38	0.74038	2.5104	3.73E-10	0.64
POC1A	25886	2.07	5.79E-26	0.448276	2.4424	9.88E-65	0.71154	2.3003	2.09E-28	0.64
HIST1H3B	8358	NA	NA	NA	2.1927	6.65E-37	0.85577	2.0583	2.73E-22	0.84
CENPW	387103	NA	NA	NA	2.8611	3.08E-61	0.75962	2.3757	7.74E-26	0.6
SMC1B	27127	3.5499	7.32E-19	0.689655	3.5222	1.29E-32	0.68269	3.2393	1.66E-16	0.76
SIX2	10736	2.3182	1.48E-12	0.586207	2.122	9.95E-18	0.47115	2.7205	3.20E-13	0.72
C9orf140	89958	2.5601	2.22E-11	0.517241	3.0824	8.92E-37	0.67308	2.9088	6.63E-12	0.72
HIST2H2AA3	8337	2.3231	5.99E-17	0.482759	2.0284	3.81E-27	0.45192	2.9133	4.51E-24	0.72
H2AFX	3014	2.1307	1.48E-28	0.413793	2.3559	2.77E-60	0.58654	2.6416	5.47E-33	0.72
GBX2	2637	NA	NA	NA	2.1465	3.47E-22	0.67308	2.0881	5.75E-19	0.76
SLC39A4	55630	NA	NA	NA	2.0328	7.18E-38	0.39423	2.3764	3.15E-17	0.72

Supplementary Table 2b. Down regulated genes in 70% of TNBC patients

Down regulated genes		Stage I			Stage II			Stage III		
GeneName	GeneID	FC	FDR	frequency	FC	FDR	frequency	FC	FDR	frequency
SCN4B	6330	-3.8108	1.91E-56	1	-4.1333	3.25E-71	0.932692308	-3.455	6.41E-36	0.8
FIGF	2277	-6.5493	1.95E-53	1	-6.6462	1.13E-86	1	-5.905	3.49E-40	0.96
TGFBR3	7049	-3.8102	2.18E-52	1	-3.8561	5.79E-56	0.855769231	-3.346	1.54E-31	0.8
NOVA1	4857	-4.7897	1.25E-51	1	-5.1405	9.83E-70	0.971153846	-4.857	2.06E-38	0.88
SDPR	8436	-5.3104	1.50E-51	1	-5.7932	2.15E-84	0.980769231	-4.547	6.88E-36	0.88
CA4	762	-7.41	1.53E-46	1	-8.0526	1.03E-97	1	-7.035	2.68E-38	0.96
ADAMTS5	11096	-3.5081	1.37E-45	1	-3.3096	4.59E-60	0.913461538	-3.258	6.32E-31	0.8
CD300LG	146894	-6.7673	1.72E-45	1	-7.6642	3.12E-79	1	-6.532	1.50E-38	0.96
FXYD1	5348	-5.105	1.18E-43	1	-5.8756	5.91E-69	0.961538462	-4.703	1.77E-35	0.88
SCN2B	6327	-5.0377	9.99E-42	1	-5.519	5.78E-74	0.980769231	-5.038	3.10E-32	0.88
PDE2A	5138	-3.8844	3.34E-41	1	-4.2627	1.73E-72	0.971153846	-3.465	1.54E-29	0.88
MAB21L1	4081	-3.8366	3.34E-40	1	-4.5394	2.86E-65	0.923076923	-4.043	1.35E-26	0.84
MYH11	4629	-5.1969	3.73E-40	1	-5.599	1.38E-61	0.971153846	-4.395	1.75E-26	0.84
LOC572558	572558	-4.1552	6.86E-40	1	-4.0255	8.81E-74	0.980769231	-3.637	1.88E-27	0.88
SCARA5	286133	-5.9383	3.21E-39	1	-6.3186	5.48E-65	0.990384615	-5.601	1.94E-30	0.92
ABCA8	10351	-5.8626	8.06E-38	1	-6.6605	3.33E-61	1	-5.643	7.48E-30	0.96
FGF10	2255	-4.6468	3.60E-37	1	-4.8801	2.73E-63	0.923076923	-4.868	1.77E-35	0.96
BTNL9	153579	-4.9242	1.12E-36	1	-5.3742	9.61E-68	0.971153846	-4.509	5.01E-30	0.92
MEOX2	4223	-3.4534	2.37E-36	1	-4.2199	6.80E-46	0.875	-2.96	5.01E-22	0.76
TNXB	7148	-5.0447	6.09E-36	1	-5.4724	5.46E-70	0.980769231	-4.565	7.16E-28	0.92
ABCA9	10350	-4.8609	7.43E-36	1	-5.7108	3.15E-70	1	-4.738	2.88E-29	0.96
OGN	4969	-5.0294	2.53E-35	1	-6.0827	3.68E-55	0.932692308	-5.358	2.65E-28	0.88
CCL14	6358	-4.6713	1.05E-34	1	-5.5794	6.03E-59	0.951923077	-3.962	3.67E-25	0.84
LRRN4CL	221091	-4.344	2.08E-34	1	-4.9705	1.05E-68	0.971153846	-3.985	3.31E-26	0.84
CPA1	1357	-4.806	2.89E-34	1	-4.7328	1.06E-69	0.980769231	-4.332	8.50E-27	1
BMX	660	-4.193	5.61E-34	1	-4.5601	2.72E-53	0.894230769	-3.635	3.09E-21	0.76

GDF10	2662	-4.8643	5.62E-34	1	-4.9746	1.95E-52	0.951923077	-4.778	2.29E-27	0.92
INMT	11185	-3.8249	5.88E-34	1	-4.0761	3.55E-56	0.903846154	-2.968	1.18E-20	0.8
ANGPTL1	9068	-4.3274	7.03E-34	1	-4.7371	2.98E-61	0.980769231	-3.947	3.33E-25	0.84
AVPR2	554	-3.8072	1.19E-33	1	-4.172	8.91E-62	0.932692308	-3.386	4.34E-26	0.8
ITIH5	80760	-4.4513	5.03E-33	1	-4.7628	4.37E-59	0.971153846	-4.132	1.46E-27	0.92
TNMD	64102	-5.6287	1.69E-31	1	-5.6124	1.60E-46	0.951923077	-5.007	3.66E-24	0.96
PDK4	5166	-4.4624	1.15E-30	1	-4.9479	5.11E-60	0.990384615	-4.116	4.20E-22	0.92
IGFBP6	3489	-3.6049	2.43E-30	1	-3.8353	1.00E-54	0.942307692	-3.011	3.28E-19	0.84
GRIA1	2890	-5.0845	3.18E-30	1	-4.9695	2.32E-60	0.971153846	-4.177	1.31E-19	0.88
TMEM178	130733	-3.8421	3.67E-30	1	-3.5816	3.14E-46	0.884615385	-3.127	8.80E-18	0.84
FHL5	9457	-3.626	3.14E-29	1	-4.1404	4.44E-55	0.942307692	-2.895	7.95E-18	0.92
CHRDL1	91851	-4.8525	5.81E-29	1	-4.8388	1.62E-50	0.980769231	-5.024	1.23E-23	0.96
ABCB5	340273	-4.78	9.38E-29	1	-5.5278	1.73E-63	0.971153846	-4.828	1.05E-24	0.88
LRRC2	79442	-3.1687	1.26E-28	1	-3.5468	1.47E-52	0.951923077	-3.412	5.41E-25	0.92
CD36	948	-5.0768	7.10E-28	1	-5.8328	8.50E-64	1	-4.505	6.65E-23	0.96
CLEC3B	7123	-4.2017	8.69E-28	1	-4.6535	5.00E-57	0.961538462	-3.622	1.96E-19	0.92
MYOC	4653	-7.1815	1.81E-27	1	-6.9666	4.00E-60	1	-6.468	2.22E-20	1
NPR1	4881	-3.7097	3.47E-27	1	-3.7884	2.28E-53	0.961538462	-3.072	7.59E-19	0.92
GPR146	115330	-2.907	6.47E-27	1	-3.1415	1.99E-62	0.980769231	-2.694	3.48E-20	0.84
CASQ2	845	-4.1983	7.19E-27	1	-4.7294	1.11E-50	0.971153846	-3.411	1.53E-16	0.88
AQP7	364	-6.1928	9.57E-27	1	-7.5977	1.85E-66	1	-5.621	9.12E-21	0.96
HRC	3270	-3.1397	1.72E-26	1	-3.3029	4.42E-50	0.951923077	-2.319	7.40E-15	0.88
KIAA0408	9729	-6.6013	1.80E-26	1	-6.8282	2.24E-58	1	-6.33	4.47E-22	0.96
ADH1B	125	-7.1795	2.30E-26	1	-9.0403	3.61E-52	1	-7.433	2.51E-24	1
HEPACAM	220296	-5.4683	2.50E-26	1	-6.2266	2.17E-60	0.990384615	-5.191	8.15E-22	1
FABP4	2167	-6.3587	2.87E-26	1	-7.4882	2.74E-48	0.971153846	-5.831	1.09E-20	0.96
ACACB	32	-4.029	5.80E-26	1	-4.4734	8.06E-66	1	-4.059	9.35E-24	1
AQP7P1	375719	-5.5551	6.01E-26	1	-6.1415	3.77E-61	1	-5.648	9.43E-24	1
EBF1	1879	-3.1335	8.48E-26	1	-3.7027	3.22E-54	0.951923077	-2.995	7.97E-20	0.84

TIMP4	7079	-5.8424	1.95E-25	1	-6.401	1.72E-56	1	-5.328	1.69E-19	0.96
CES1	1066	-5.1909	3.35E-25	1	-5.4852	1.66E-48	0.971153846	-4.518	2.03E-17	0.96
MMRN1	22915	-4.9549	5.72E-25	1	-5.5729	4.93E-51	0.971153846	-4.252	9.00E-18	0.88
LRRN3	54674	-3.344	1.06E-24	1	-3.6596	6.35E-44	0.903846154	-3.661	9.25E-22	0.88
HSD17B13	345275	-5.0593	1.10E-24	1	-5.5354	2.88E-59	0.990384615	-5.413	3.57E-23	1
GLYAT	10249	-6.5217	4.29E-24	1	-7.27	6.65E-64	1	-6.728	1.77E-22	1
PDE8B	8622	-3.4241	5.51E-24	1	-3.577	1.61E-41	0.932692308	-3.445	6.07E-19	0.8
KCNE1	3753	-4.4069	6.11E-24	1	-4.2467	3.16E-44	0.961538462	-3.824	4.72E-16	0.96
LYVE1	10894	-4.6943	6.11E-24	1	-5.0627	5.49E-54	0.971153846	-4.415	2.25E-18	0.96
PLIN4	729359	-6.3833	9.29E-24	1	-7.1417	9.93E-54	0.980769231	-5.894	1.02E-19	0.96
PLIN1	5346	-6.7001	1.21E-23	1	-8.2119	2.67E-57	1	-6.482	3.58E-20	1
ACSM5	54988	-4.4308	1.54E-23	1	-4.9084	7.37E-54	1	-4.138	8.70E-20	0.96
TUSC5	286753	-6.9683	3.82E-23	1	-8.3242	1.86E-56	1	-6.889	1.56E-20	1
GPD1	2819	-6.5449	5.10E-23	1	-7.4312	2.26E-58	1	-6.159	3.71E-20	1
AOC3	8639	-4.0826	5.63E-23	1	-4.5034	4.40E-56	1	-3.726	5.08E-18	0.92
ITGA7	3679	-3.8256	6.23E-23	1	-3.6951	1.29E-44	0.932692308	-3.039	7.80E-15	0.8
LOC283392	283392	-5.8579	9.31E-23	1	-6.6172	1.69E-61	1	-5.6	4.10E-18	0.96
PI16	221476	-5.0366	9.76E-23	1	-5.8023	4.16E-46	0.951923077	-4.898	5.70E-17	0.84
FHL1	2273	-4.3886	1.11E-22	1	-4.8762	1.47E-54	1	-4.211	1.95E-18	0.96
DEFB132	400830	-6.2968	2.23E-22	1	-6.889	1.94E-62	1	-6.123	1.08E-18	0.96
LEP	3952	-7.2176	5.13E-22	1	-8.2675	5.29E-56	1	-7.378	3.81E-20	1
HBB	3043	-5.3729	6.00E-22	1	-5.1295	2.39E-42	0.980769231	-3.76	6.50E-11	0.96
TNNT3	7140	-4.7536	6.52E-22	1	-4.6903	1.04E-34	0.990384615	-4.126	2.21E-16	1
CIDEC	63924	-6.341	1.62E-21	1	-7.7346	7.15E-57	0.990384615	-6.727	2.05E-20	1
C14orf180	400258	-6.0508	2.03E-21	1	-6.8936	1.26E-55	0.980769231	-6.079	8.66E-20	1
ADRA1A	148	-4.1217	2.35E-21	1	-4.3117	5.34E-46	0.971153846	-4.452	2.52E-21	1
CRHBP	1393	-4.0722	5.24E-21	1	-4.2681	7.81E-48	0.990384615	-3.578	6.05E-15	1
ADIPOQ	9370	-6.9671	6.25E-21	1	-8.8911	2.72E-48	1	-7.023	1.92E-18	1
LIPE	3991	-4.952	7.20E-21	1	-5.4958	7.13E-52	0.980769231	-4.793	4.83E-18	1

SRL	6345	-2.7089	1.21E-20	1	-2.789	2.25E-30	0.951923077	-2.389	3.20E-14	0.96
APOB	338	-5.2006	2.70E-20	1	-5.7864	3.71E-52	1	-5.461	1.15E-18	0.96
DPT	1805	-3.6856	2.89E-20	1	-4.1365	1.32E-44	0.990384615	-3.756	1.13E-16	0.96
HBA2	3040	-4.9569	4.63E-20	1	-4.4606	3.84E-38	0.980769231	-3.119	1.84E-08	0.84
GPAM	57678	-3.938	6.31E-20	1	-4.0358	4.93E-51	1	-3.965	1.55E-17	1
KCNIP2	30819	-5.0072	1.24E-19	1	-5.4724	7.01E-53	1	-5.002	5.06E-18	1
RDH5	5959	-4.1018	1.39E-19	1	-4.5383	4.81E-50	0.990384615	-4.176	1.08E-17	1
LCN6	158062	-2.9899	2.37E-19	1	-3.0467	3.45E-45	0.971153846	-2.418	6.74E-11	0.76
CLDN19	149461	-5.2289	3.73E-19	1	-5.4749	1.97E-44	0.961538462	-4.854	1.50E-14	0.92
RBP4	5950	-5.2939	5.45E-19	1	-5.8323	1.24E-41	0.971153846	-5.493	5.16E-17	0.96
LOC90586	90586	-2.8591	5.46E-19	1	-2.8789	9.73E-45	0.961538462	-2.683	1.00E-14	0.92
AQPEP	206338	-4.8668	6.43E-19	1	-5.2668	7.32E-50	1	-5.014	2.45E-16	0.96
SGCG	6445	-4.7605	7.12E-19	1	-4.9102	3.78E-46	0.980769231	-5.211	5.14E-19	1
PYGM	5837	-3.7239	1.05E-18	1	-3.9472	7.42E-48	1	-3.212	1.55E-13	1
FAM180B	399888	-4.0821	1.46E-18	1	-3.6825	9.28E-34	0.903846154	-3.578	1.75E-13	0.92
GLRA4	441509	-3.6159	1.65E-18	1	-3.5623	5.41E-46	0.980769231	-3.489	2.06E-15	1
MRAP	56246	-6.2038	2.44E-18	1	-6.9524	4.45E-49	0.980769231	-5.881	4.23E-15	1
PCK1	5105	-6.2822	3.71E-18	1	-7.1138	1.88E-47	0.980769231	-6.02	2.95E-15	1
ACVR1C	130399	-4.5472	6.70E-18	1	-5.2993	1.30E-48	0.990384615	-4.8	4.29E-17	1
GABRA2	2555	-3.4076	1.19E-17	1	-3.5934	1.14E-41	0.932692308	-3.608	1.59E-16	0.92
CPB1	1360	-5.9033	3.17E-17	1	-6.4946	4.58E-41	0.990384615	-5.159	9.25E-12	0.88
HSPB7	27129	-4.671	3.21E-17	1	-5.4136	7.36E-47	1	-4.667	4.55E-15	0.96
FRMD1	79981	-4.2005	8.92E-17	1	-4.59	7.41E-43	0.980769231	-4.421	2.71E-16	0.96
SLC19A3	80704	-4.6494	1.72E-16	1	-5.1104	2.26E-42	1	-4.61	1.88E-14	0.96
LOC55908	55908	-4.4672	2.26E-16	1	-4.7384	8.07E-46	0.990384615	-4.451	2.00E-14	0.96
SLC14A2	8170	-4.0078	2.63E-16	1	-4.217	8.37E-42	0.990384615	-3.571	1.12E-11	0.92
TRDN	10345	-4.8923	2.71E-16	1	-4.3835	1.05E-30	0.990384615	-3.823	1.28E-09	1
LGALS12	85329	-4.9533	5.47E-16	1	-6.0588	2.18E-45	0.971153846	-4.802	8.11E-15	1
SLC7A3	84889	-4.7559	1.18E-15	1	-4.4813	2.93E-31	0.932692308	-4.294	5.17E-11	0.84

KLB	152831	-4.0578	1.19E-15	1	-4.6703	6.29E-45	1	-4.472	1.18E-15	1
HBA1	3039	-5.8625	2.62E-15	1	-6.0248	1.11E-33	0.980769231	-4.149	2.08E-07	0.88
PKHD1L1	93035	-3.6322	5.77E-15	1	-3.5905	1.51E-28	0.913461538	-4.205	1.40E-15	0.92
FAM196B	100131897	-4.0393	8.28E-15	1	-4.2942	3.20E-33	0.942307692	-4.239	3.39E-13	0.92
AQP7P3	441432	-3.6603	5.16E-14	1	-3.9877	2.26E-40	0.990384615	-3.345	9.44E-11	1
KCNC2	3747	-4.6892	1.02E-13	1	-4.8142	8.98E-37	0.990384615	-4.448	5.60E-11	0.96
NTS	4922	-4.4272	2.33E-13	1	-3.4231	6.73E-19	0.913461538	-2.653	7.95E-05	0.88
LHCGR	3973	-3.5234	4.54E-13	1	-3.7289	2.57E-38	0.990384615	-3.516	2.24E-11	0.96
OXTR	5021	-4.7748	4.55E-13	1	-4.8471	4.03E-33	0.971153846	-4.695	5.52E-11	0.96
GYG2	8908	-3.351	5.45E-13	1	-3.3622	1.35E-30	0.990384615	-3.302	2.71E-11	1
MYH1	4619	-4.0379	7.09E-13	1	-4.3674	9.36E-39	1	-3.847	1.66E-10	1
GPR144	347088	-4.4207	1.36E-12	1	-4.5025	1.26E-34	0.980769231	-3.996	1.80E-09	0.96
TP53AIP1	63970	-3.6599	1.38E-12	1	-3.3982	3.80E-26	0.884615385	-2.757	5.34E-07	0.84
SMYD1	150572	-4.8108	2.95E-12	1	-4.5385	9.19E-26	0.951923077	-5.493	1.34E-12	0.96
HMGCS2	3158	-6.1116	4.39E-12	1	-7.098	5.98E-33	0.961538462	-4.978	1.37E-07	0.84
EPB42	2038	-3.908	4.47E-12	1	-4.1503	9.67E-33	0.961538462	-3.858	1.14E-10	1
UGT2B4	7363	-2.8436	2.14E-11	1	-2.843	7.70E-29	0.971153846	-2.361	5.33E-07	0.8
ITIH2	3698	-4.2524	7.58E-11	1	-4.4025	2.33E-30	0.980769231	-4.325	8.49E-10	0.96
MYOZ2	51778	-2.1536	7.78E-11	1	-2.4448	3.69E-30	0.990384615	NA	NA	NA
CSF3	1440	-3.6863	1.00E-10	1	-3.1602	3.50E-20	0.951923077	-2.209	0.000590777	0.84
CAV3	859	-2.0384	1.30E-10	1	NA	NA	NA	NA	NA	NA
CST9	128822	-3.8442	2.52E-10	1	-3.6015	4.22E-24	0.971153846	-2.97	1.18E-05	0.92
SLC22A12	116085	-2.9215	4.29E-10	1	-2.9589	1.22E-28	0.990384615	-2.947	4.24E-09	1
LOC729467	729467	-2.2848	5.39E-09	1	NA	NA	NA	-2.155	2.65E-07	1
GYS2	2998	-3.1287	7.65E-09	1	-3.086	7.27E-23	0.980769231	-3.157	5.89E-08	1
DES	1674	-3.032	1.97E-08	1	-3.455	2.41E-20	0.980769231	-2.598	1.54E-05	0.96
AMPD1	270	-2.938	2.68E-08	1	-2.4211	3.82E-13	0.961538462	-2.696	3.56E-06	0.96
HBG1	3047	-2.4553	9.21E-08	1	-2.2201	9.36E-18	0.980769231	NA	NA	NA
PDE3B	5140	-2.4937	2.18E-07	1	-2.6911	4.17E-19	0.961538462	-3.122	3.89E-09	0.96

ARHGAP36	158763	-3.9528	4.05E-07	1	-4.3261	4.05E-20	0.971153846	-3.769	9.20E-06	0.92
ALAS2	212	-2.2372	1.11E-06	1	-2.21	2.80E-17	0.980769231	NA	NA	NA
MYH2	4620	-2.048	8.30E-06	1	-2.1016	8.96E-17	1	NA	NA	NA
PAMR1	25891	-4.5922	3.18E-47	0.9655172	-4.8376	3.33E-76	0.971153846	-4.218	7.46E-33	0.88
SSTR1	6751	-4.5347	1.37E-46	0.9655172	-4.5204	1.06E-56	0.913461538	-3.548	1.19E-23	0.8
PGM5	5239	-4.2498	6.64E-46	0.9655172	-4.4939	1.35E-61	0.913461538	-3.63	1.33E-28	0.84
ABCA10	10349	-4.8242	2.57E-41	0.9655172	-5.3367	9.96E-79	0.980769231	-4.912	3.48E-35	0.92
RERGL	79785	-4.8153	7.48E-40	0.9655172	-5.2222	2.95E-50	0.875	-4	7.11E-21	0.8
ALDH1A2	8854	-4.0688	7.48E-40	0.9655172	-4.4592	2.50E-62	0.932692308	-3.922	3.89E-27	0.84
GPIHBP1	338328	-5.0231	7.97E-40	0.9655172	-5.5069	5.73E-70	0.961538462	-3.953	7.51E-27	0.88
ARHGAP20	57569	-3.8031	1.71E-38	0.9655172	-4.3218	2.26E-66	0.971153846	-3.791	1.58E-32	0.88
AGTR1	185	-4.4639	1.34E-37	0.9655172	-4.9327	1.75E-70	0.971153846	-4.238	1.47E-27	0.84
CTTNBP2	83992	-3.5613	4.44E-37	0.9655172	-3.9209	8.27E-50	0.875	-3.896	2.99E-29	0.76
CAPN11	11131	-3.9465	5.42E-37	0.9655172	-4.1285	5.96E-63	0.942307692	-3.393	5.27E-25	0.88
LRRC3B	116135	-4.4968	1.83E-36	0.9655172	-4.3839	3.64E-59	0.923076923	-4.171	2.02E-27	0.88
SLC17A7	57030	-3.7361	8.05E-36	0.9655172	-3.5367	2.96E-51	0.903846154	-3.364	6.82E-27	0.88
CAV1	857	-3.568	6.78E-33	0.9655172	-3.7413	1.31E-61	0.932692308	-2.87	1.86E-21	0.8
ATP1A2	477	-6.1342	1.51E-32	0.9655172	-7.9079	1.04E-82	1	-6.337	1.49E-30	1
SLC16A12	387700	-4.4035	1.86E-32	0.9655172	-4.4456	7.37E-61	0.961538462	-3.972	1.29E-25	0.88
GHR	2690	-4.1547	4.95E-31	0.9655172	-4.3422	2.60E-53	0.942307692	-3.917	5.58E-25	0.96
SEMA3G	56920	-3.567	6.12E-31	0.9655172	-4.0102	1.79E-62	0.961538462	-3.359	1.06E-23	0.92
ESR1	2099	-4.5273	6.86E-31	0.9655172	-5.075	3.00E-53	0.932692308	-3.649	2.37E-18	0.8
IGSF10	285313	-3.8107	1.15E-29	0.9655172	-3.7435	2.17E-41	0.826923077	-4.192	3.03E-32	0.92
PGR	5241	-5.198	1.67E-29	0.9655172	-5.721	2.65E-55	0.951923077	-4.751	5.94E-19	0.88
CDO1	1036	-4.2436	6.38E-29	0.9655172	-4.5918	4.54E-47	0.903846154	-4.32	1.82E-22	0.92
C18orf34	374864	-3.7272	1.06E-28	0.9655172	-4.4263	1.10E-65	0.971153846	-3.341	3.90E-24	0.8
FREM1	158326	-3.9525	1.38E-28	0.9655172	-4.3897	7.43E-53	0.913461538	-4.147	3.54E-26	0.88
SVEP1	79987	-4.0355	1.88E-28	0.9655172	-4.7349	1.60E-54	0.980769231	-4.273	3.67E-23	0.92
LEPR	3953	-3.1295	2.99E-28	0.9655172	-3.8022	1.60E-63	0.961538462	-3.119	8.60E-23	0.84

ASPA	443	-4.0176	3.43E-28	0.9655172	-4.7399	4.31E-62	0.990384615	-4.49	1.97E-26	0.96
DNAH9	1770	-3.413	6.20E-28	0.9655172	-3.918	2.43E-49	0.923076923	-3.54	3.15E-20	0.76
TMEM132C	92293	-5.8393	1.43E-27	0.9655172	-7.1923	4.23E-65	0.980769231	-6.268	2.07E-28	1
SLC2A4	6517	-4.6851	1.81E-27	0.9655172	-4.941	1.33E-56	0.990384615	-4.15	1.31E-19	1
SCN4A	6329	-3.8789	4.81E-27	0.9655172	-4.5696	1.54E-56	0.961538462	-3.701	4.50E-21	0.88
TNS1	7145	-3.1385	1.71E-26	0.9655172	-3.3723	2.38E-59	0.990384615	-3.166	1.88E-23	0.92
C10orf116	10974	-3.7601	1.71E-25	0.9655172	-4.2743	1.74E-48	0.923076923	-3.159	1.06E-17	0.76
AKAP12	9590	-3.4144	2.90E-25	0.9655172	-3.6223	2.21E-46	0.894230769	-3.176	9.06E-20	0.92
GALNTL2	117248	-3.6947	6.11E-25	0.9655172	-4.299	2.67E-57	0.980769231	-4.131	1.10E-23	0.88
CKMT2	1160	-3.7721	9.99E-25	0.9655172	-3.5693	5.51E-35	0.942307692	-3.602	4.40E-21	1
RGS22	26166	-4.3395	1.82E-24	0.9655172	-4.7493	1.74E-55	0.961538462	-4	3.16E-19	0.96
ADH1A	124	-5.5549	3.28E-24	0.9655172	-6.5143	8.79E-64	1	-6.085	7.08E-25	1
CFD	1675	-4.443	5.16E-24	0.9655172	-4.9691	4.41E-55	0.961538462	-3.667	6.29E-18	0.92
PLAC9	219348	-3.9539	7.12E-24	0.9655172	-4.4699	3.87E-50	0.942307692	-3.342	1.47E-16	0.84
ANGPT4	51378	-3.6765	1.29E-23	0.9655172	-4.0393	3.23E-61	0.980769231	-3.457	6.55E-19	0.96
HEPN1	641654	-5.2524	2.26E-23	0.9655172	-5.9061	6.66E-56	0.990384615	-5.211	5.77E-21	1
MYOM1	8736	-3.5984	2.93E-23	0.9655172	-4.2111	1.85E-56	1	-3.672	1.39E-20	0.96
MAP1LC3C	440738	-3.5789	8.25E-23	0.9655172	-4.144	3.21E-53	0.980769231	-3.646	4.37E-21	1
PPARG	5468	-3.4642	2.40E-22	0.9655172	-3.6443	9.80E-49	0.971153846	-2.908	3.13E-15	0.88
MLXIPL	51085	-4.6441	4.96E-22	0.9655172	-4.6961	2.00E-44	0.951923077	-4.492	5.22E-20	1
NAALAD2	10003	-3.0839	5.22E-22	0.9655172	-3.1938	7.25E-44	0.913461538	-3.454	2.93E-23	0.96
C1QTNF7	114905	-3.8319	6.27E-22	0.9655172	-4.7401	4.54E-54	0.961538462	-3.773	3.66E-18	0.88
B3GALT1	8708	-3.4676	7.07E-22	0.9655172	-3.5148	1.89E-45	0.942307692	-3.497	3.52E-18	0.84
CNN1	1264	-3.7413	8.43E-22	0.9655172	-4.2299	2.58E-45	0.923076923	-3.518	1.49E-16	0.8
NNAT	4826	-4.1119	1.73E-21	0.9655172	-4.7605	1.12E-51	0.961538462	-4.302	7.03E-20	0.96
PDZRN4	29951	-3.155	7.09E-21	0.9655172	-3.2233	1.05E-34	0.846153846	-3.215	4.20E-16	0.8
C7orf58	79974	-3.3109	1.01E-20	0.9655172	-3.8435	1.03E-47	0.980769231	-3.393	3.35E-19	0.92
GSTM5	2949	-4.4006	1.69E-20	0.9655172	-5.2762	5.38E-58	1	-4.434	3.95E-19	0.92
NPY2R	4887	-6.0554	2.25E-20	0.9655172	-6.5778	2.86E-52	0.961538462	-6.111	4.65E-18	0.96

PLIN5	440503	-3.6189	2.33E-20	0.9655172	-3.8313	2.51E-34	0.865384615	-3.179	5.90E-15	0.8
ELANE	1991	-2.8544	2.59E-20	0.9655172	-2.8022	7.61E-44	0.932692308	-2.193	2.89E-11	0.88
SORBS1	10580	-3.2426	1.02E-19	0.9655172	-3.645	1.88E-51	0.980769231	-3.932	7.04E-21	0.96
SGK2	10110	-4.0862	1.92E-19	0.9655172	-4.3887	9.55E-43	0.932692308	-3.649	2.55E-14	0.92
KLHL33	123103	-2.6015	1.44E-18	0.9655172	-2.8716	5.49E-49	0.990384615	-2.794	1.66E-18	1
FLJ37543	285668	-2.5666	1.72E-18	0.9655172	-2.5469	3.16E-42	0.913461538	-2.519	6.16E-16	0.88
RXRG	6258	-3.5875	1.74E-18	0.9655172	-3.6077	1.16E-36	0.903846154	-3.63	4.80E-18	0.96
CIDEA	1149	-5.7831	1.66E-17	0.9655172	-7.0581	7.03E-51	0.990384615	-5.976	4.92E-18	0.96
HEPACAM2	253012	-4.8468	7.50E-17	0.9655172	-4.8006	5.80E-35	0.932692308	-4.279	2.07E-11	0.84
ADH4	127	-3.6879	6.59E-16	0.9655172	-3.7256	1.09E-33	0.903846154	-3.344	1.40E-11	0.88
C11orf87	399947	-2.5665	3.18E-15	0.9655172	-2.2966	5.50E-28	0.903846154	NA	NA	NA
CA3	761	-3.731	5.88E-15	0.9655172	-3.5637	8.82E-27	0.961538462	-3.926	1.34E-13	0.96
PRRT4	401399	-3.8461	1.01E-14	0.9655172	-3.8501	2.78E-31	0.894230769	-3.284	4.18E-11	0.96
CITED1	4435	-4.1931	1.18E-14	0.9655172	-4.1758	1.19E-32	0.942307692	-3.369	3.63E-09	0.92
NXNL1	115861	-2.457	4.81E-14	0.9655172	-2.444	4.49E-36	0.961538462	-2.29	4.14E-11	0.92
CMYA5	202333	-2.9677	5.43E-14	0.9655172	-3.2337	9.62E-33	0.980769231	-2.626	2.63E-11	0.96
SLC13A2	9058	-5.27	1.66E-13	0.9655172	-4.6085	8.07E-22	0.903846154	-4.315	3.46E-08	0.92
NCRNA00110	642976	-3.5011	4.22E-13	0.9655172	-3.6179	7.39E-35	0.951923077	-3.273	3.12E-10	0.88
AGR3	155465	-5.301	1.26E-12	0.9655172	-5.4911	3.00E-29	0.942307692	-3.88	1.65E-06	0.8
MYOZ3	91977	-2.0244	1.50E-12	0.9655172	-2.0895	2.15E-23	0.913461538	NA	NA	NA
CYP3A4	1576	-2.304	1.78E-12	0.9655172	-2.1947	5.98E-28	0.875	-2.109	7.27E-09	0.92
OR2L13	284521	-2.8386	2.12E-12	0.9655172	-2.898	4.31E-31	0.932692308	-2.504	1.87E-08	0.84
ACSM2A	123876	-2.0827	9.92E-12	0.9655172	-2.0808	4.17E-29	0.932692308	-2.081	1.57E-10	0.96
SLC7A10	56301	-4.1054	2.37E-11	0.9655172	-4.5821	3.21E-30	0.971153846	-4.043	1.44E-09	0.92
PGA3	643834	-3.3127	3.65E-11	0.9655172	-3.2112	4.94E-26	0.942307692	-2.984	9.16E-09	0.88
RFX6	222546	-3.3754	4.79E-11	0.9655172	-3.5079	8.28E-31	0.971153846	-3.377	8.88E-10	0.96
SGCA	6442	-2.4547	2.89E-10	0.9655172	-2.2754	3.28E-20	0.971153846	NA	NA	NA
GABRA4	2557	-2.5227	2.97E-10	0.9655172	-2.3497	4.16E-19	0.942307692	-2.32	8.64E-08	0.88
PLN	5350	-2.044	3.92E-10	0.9655172	-2.4584	3.42E-25	0.875	NA	NA	NA

SEL1L2	80343	-2.5282	5.91E-10	0.9655172	-2.6795	5.89E-30	0.980769231	-2.464	1.40E-08	1
TAT	6898	-5.6192	1.03E-09	0.9655172	-6.8266	1.21E-33	0.971153846	-6.006	9.44E-10	0.96
HHATL	57467	-2.8834	1.82E-09	0.9655172	-3.0798	5.84E-29	0.990384615	-2.964	2.23E-09	1
FRMPD2	143162	-3.0032	2.87E-09	0.9655172	-3.2564	9.48E-28	0.990384615	-2.737	9.62E-07	0.92
ANGPTL5	253935	-2.9669	4.12E-09	0.9655172	-3.3325	1.05E-26	0.951923077	-3.29	1.47E-09	0.96
COL17A1	1308	-4.4047	6.84E-09	0.9655172	-4.9554	1.66E-24	0.942307692	-3.933	2.30E-06	0.8
CYP2A7	1549	-2.5004	7.45E-09	0.9655172	-2.4518	1.54E-20	0.971153846	-2.51	7.13E-08	0.96
SERPINA6	866	-4.3867	1.34E-08	0.9655172	-4.6258	7.33E-25	0.980769231	-4.51	5.67E-08	0.92
TFF1	7031	-4.42	1.96E-08	0.9655172	-5.4586	8.86E-27	0.971153846	-3.821	8.91E-06	0.88
C12orf39	80763	-3.4393	1.26E-07	0.9655172	-3.9078	3.32E-22	0.942307692	-3.939	1.83E-08	0.96
GLRA3	8001	-3.919	2.02E-07	0.9655172	-4.4058	3.49E-24	0.980769231	-3.854	2.02E-06	0.88
ALB	213	-3.9435	3.23E-07	0.9655172	-5.3207	1.94E-29	0.980769231	-5.19	1.62E-09	0.88
AADAC	13	-3.2685	3.43E-07	0.9655172	-3.7684	6.02E-23	0.990384615	-2.845	3.87E-05	0.96
CSN1S1	1446	-3.1003	4.47E-05	0.9655172	-3.5605	3.76E-18	0.990384615	-3.583	2.47E-06	1
CYP1A1	1543	-2.3813	0.000241958	0.9655172	-2.5588	1.42E-12	1	-2.304	0.001165662	0.92
ANKRD29	147463	-3.9865	1.31E-54	0.9310345	-3.9169	7.99E-73	0.903846154	-3.352	4.84E-34	0.76
C5orf4	10826	-3.2776	4.34E-46	0.9310345	-3.6866	4.21E-77	0.932692308	-2.94	2.82E-33	0.72
LMOD1	25802	-3.4699	3.93E-44	0.9310345	-4.0914	3.91E-68	0.913461538	-3.454	3.78E-33	0.84
MME	4311	-3.9582	3.11E-43	0.9310345	-4.2295	1.74E-51	0.884615385	-4.36	1.87E-29	0.88
MATN2	4147	-3.8837	3.96E-42	0.9310345	-3.7282	2.08E-57	0.826923077	-2.873	1.33E-32	0.68
TMTC1	83857	-3.7693	2.36E-40	0.9310345	-3.5228	5.49E-51	0.817307692	-3.36	3.87E-24	0.72
SYNPO2	171024	-3.6163	7.26E-40	0.9310345	-4.1074	1.14E-62	0.913461538	-3.621	4.38E-30	0.88
ADAM33	80332	-4.3096	3.21E-39	0.9310345	-4.9151	5.89E-63	0.903846154	-4.089	2.23E-30	0.8
DACH1	1602	-3.7655	2.29E-37	0.9310345	-4.1541	5.98E-54	0.875	-3.022	1.96E-21	0.68
GPC3	2719	-4.777	2.30E-37	0.9310345	-4.6822	3.89E-54	0.913461538	-4.182	1.79E-28	0.88
TPRG1	285386	-3.5214	2.16E-36	0.9310345	-3.4898	7.54E-51	0.875	-3.024	4.07E-27	0.76
C6orf97	80129	-3.4449	8.56E-36	0.9310345	-3.8164	1.44E-60	0.923076923	-3.354	1.60E-27	0.84
AGAP11	119385	-3.6705	3.90E-35	0.9310345	-3.8673	1.47E-58	0.961538462	-3.488	9.57E-28	0.88
C16orf89	146556	-4.4412	5.61E-34	0.9310345	-5.0848	1.68E-50	0.875	-3.787	4.45E-23	0.76

DHDPSL	112817	-4.3718	3.89E-33	0.9310345	-4.0617	3.22E-51	0.875	-4.201	9.39E-31	0.96
MAOA	4128	-5.0447	1.23E-32	0.9310345	-5.2931	4.23E-58	0.980769231	-4.006	1.00E-22	0.92
ATOH8	84913	-4.058	4.98E-31	0.9310345	-4.0975	6.81E-58	0.951923077	-3.468	3.78E-26	0.84
SCN7A	6332	-4.6974	2.14E-30	0.9310345	-5.5958	3.02E-64	0.951923077	-4.326	1.59E-21	0.76
SCN3A	6328	-3.6365	2.82E-29	0.9310345	-4.271	1.18E-48	0.884615385	-4.075	2.02E-26	0.84
GFRA1	2674	-4.2153	5.30E-29	0.9310345	-5.0604	9.53E-42	0.875	-4.363	2.95E-20	0.76
ZNF676	163223	-3.8019	6.05E-29	0.9310345	-3.9296	6.08E-47	0.855769231	-3.504	2.92E-25	0.84
LOC728264	728264	-4.0537	6.85E-29	0.9310345	-4.515	2.81E-57	0.961538462	-3.674	4.18E-20	0.8
EBF3	253738	-3.2746	5.56E-28	0.9310345	-3.5332	4.48E-50	0.865384615	-2.761	1.17E-18	0.72
CASP12	120329	-2.7496	1.82E-27	0.9310345	-2.9212	2.00E-59	0.923076923	-2.218	2.71E-16	0.76
C7orf41	222166	-2.8775	1.83E-27	0.9310345	-2.7706	7.48E-50	0.884615385	-2.633	2.40E-21	0.88
CCL16	6360	-2.8336	1.94E-27	0.9310345	-3.0064	5.10E-57	0.884615385	-2.211	1.12E-15	0.72
HRASLS5	117245	-4.4742	1.95E-26	0.9310345	-4.7576	6.50E-44	0.903846154	-4.418	1.13E-22	0.92
ANGPTL7	10218	-5.8738	8.53E-26	0.9310345	-5.7946	6.59E-49	0.903846154	-5.696	2.90E-23	0.92
MYOCD	93649	-3.6054	9.12E-26	0.9310345	-4.006	4.33E-50	0.913461538	-2.923	4.93E-16	0.88
COX7A1	1346	-2.9966	2.13E-25	0.9310345	-3.3886	6.76E-51	0.951923077	-2.913	1.43E-19	0.84
THRSP	7069	-5.1675	5.47E-25	0.9310345	-5.977	6.58E-44	0.913461538	-4.773	5.62E-18	0.84
C4orf49	84709	-2.9422	3.04E-24	0.9310345	-2.9645	1.13E-42	0.875	-2.575	4.77E-16	0.8
HSPB6	126393	-4.6104	1.63E-23	0.9310345	-5.6383	1.41E-61	0.990384615	-4.528	2.05E-21	0.96
EGR1	1958	-3.2465	2.93E-23	0.9310345	-3.4391	2.87E-51	0.894230769	-2.667	1.27E-14	0.76
TRPM3	80036	-3.0012	5.32E-23	0.9310345	-2.7697	3.64E-43	0.855769231	-2.242	1.35E-11	0.68
ADCYAP1R1	117	-4.0024	5.54E-23	0.9310345	-4.0737	1.17E-40	0.913461538	-3.613	7.65E-18	0.92
GLDN	342035	-3.6221	2.27E-22	0.9310345	-3.8568	2.49E-46	0.913461538	-2.675	1.14E-13	0.8
FOSB	2354	-4.6806	2.56E-22	0.9310345	-4.9661	1.16E-49	0.951923077	-3.889	1.51E-15	0.88
ALDH1L1	10840	-4.7279	5.11E-21	0.9310345	-5.3145	3.22E-46	0.961538462	-4.801	1.52E-20	1
CMA1	1215	-4.3406	8.82E-21	0.9310345	-4.4943	2.60E-44	0.942307692	-4.059	3.63E-16	0.92
FZD4	8322	-2.8706	2.82E-20	0.9310345	-3.2837	1.08E-50	0.942307692	-2.62	1.16E-15	0.88
FOS	2353	-3.3737	2.91E-20	0.9310345	-3.7046	2.54E-46	0.913461538	-2.711	8.20E-13	0.8
EGR3	1960	-3.5579	3.43E-20	0.9310345	-3.8637	4.23E-42	0.923076923	-2.827	2.88E-12	0.84

CES4	51716	-4.1524	5.75E-20	0.9310345	-4.0824	2.26E-40	0.951923077	-3.246	5.09E-12	0.84
KLF15	28999	-2.8388	8.66E-20	0.9310345	-3.1822	9.55E-43	0.903846154	-3.455	3.45E-20	0.84
CYP2A6	1548	-4.3276	1.07E-18	0.9310345	-5.1764	4.40E-56	0.971153846	-4.542	3.09E-21	1
LPL	4023	-4.2564	1.28E-18	0.9310345	-4.2948	3.24E-38	0.951923077	-4.311	1.30E-17	1
ECM2	1842	-2.847	2.19E-18	0.9310345	-3.5996	2.47E-47	0.990384615	-2.974	6.00E-17	0.88
PIWIL2	55124	-2.9186	7.06E-18	0.9310345	-2.8556	3.16E-26	0.903846154	-2.797	3.20E-14	0.88
P2RX6	9127	-3.1134	1.03E-17	0.9310345	-2.727	1.46E-29	0.884615385	-2.736	7.44E-13	0.88
PFKFB1	5207	-4.348	1.03E-17	0.9310345	-4.9884	1.34E-49	0.980769231	-4.778	3.30E-18	0.96
GRIA4	2893	-4.919	2.06E-17	0.9310345	-5.6305	3.78E-43	0.961538462	-4.822	4.66E-14	0.92
PAPPA2	60676	-3.4265	7.24E-17	0.9310345	-4.1525	3.89E-48	0.951923077	-3.575	1.82E-15	0.88
PPP1R1A	5502	-3.7728	7.95E-17	0.9310345	-4.2881	2.30E-35	0.903846154	-3.673	3.48E-14	0.8
GPX3	2878	-3.2512	1.70E-15	0.9310345	-3.4157	1.25E-37	0.942307692	-3.065	1.09E-12	0.88
MMP27	64066	-2.9755	3.23E-15	0.9310345	-3.4913	1.83E-45	0.971153846	-3.506	3.12E-17	0.96
CCDC3	83643	-2.5305	3.86E-15	0.9310345	-2.2772	6.33E-25	0.817307692	-2.094	8.48E-10	0.8
CDH12	1010	-2.907	1.42E-14	0.9310345	-2.3454	3.76E-18	0.836538462	-2.13	8.01E-07	0.68
BMP3	651	-3.5025	1.81E-14	0.9310345	-3.8517	7.03E-36	0.961538462	-3.099	1.25E-09	0.88
LGI1	9211	-2.3087	4.72E-14	0.9310345	-2.0998	3.22E-20	0.865384615	-2.04	1.48E-09	0.84
MPPED1	758	-3.0561	4.83E-14	0.9310345	-2.91	1.79E-28	0.884615385	-2.489	4.93E-09	0.84
PTPRQ	374462	-3.9717	5.31E-14	0.9310345	-4.6913	1.32E-41	0.980769231	-4.87	4.61E-18	1
BPIL1	80341	-4.129	5.59E-14	0.9310345	-4.919	4.62E-37	0.932692308	-4.404	9.87E-13	0.8
GRPR	2925	-3.8967	8.04E-14	0.9310345	-4.4482	4.10E-39	0.961538462	-2.963	1.65E-07	0.84
TRHDE	29953	-4.1682	9.26E-14	0.9310345	-4.6414	1.83E-37	0.980769231	-3.62	1.10E-09	0.84
PEBP4	157310	-3.112	3.71E-13	0.9310345	-3.2463	6.27E-32	0.971153846	-3.23	4.52E-13	0.96
G0S2	50486	-3.4904	3.81E-13	0.9310345	-3.9587	2.12E-35	0.951923077	-3.115	9.85E-10	0.88
AK5	26289	-3.857	7.93E-13	0.9310345	-4.3949	1.50E-30	0.894230769	-3.653	1.43E-09	0.8
PLXNA4	91584	-2.8295	8.33E-13	0.9310345	-3.5365	5.47E-33	0.884615385	-3.603	1.61E-15	0.92
C2orf84	653140	-2.4404	1.38E-12	0.9310345	-2.6336	1.89E-29	0.894230769	-2.402	1.19E-10	0.92
MUC6	4588	-4.5761	1.38E-12	0.9310345	-4.1566	1.09E-20	0.894230769	-4.225	1.84E-09	0.88
CLEC4G	339390	-3.0641	1.51E-12	0.9310345	-2.9778	2.16E-24	0.875	-2.315	1.00E-06	0.84

GRIK1	2897	-3.6171	1.86E-12	0.9310345	-3.8108	1.13E-33	0.971153846	-3.538	1.04E-10	0.88
TPSG1	25823	-2.5697	5.77E-12	0.9310345	-2.7381	2.45E-31	0.913461538	-2.693	6.85E-12	0.92
LOC389033	389033	-3.0595	7.32E-12	0.9310345	-3.0141	1.58E-29	0.932692308	-2.535	3.96E-08	0.88
KCNJ3	3760	-2.868	1.02E-11	0.9310345	-3.0511	2.24E-30	0.932692308	NA	NA	NA
AFP	174	-4.4099	1.35E-11	0.9310345	-4.6291	1.99E-30	0.942307692	-3.379	1.43E-06	0.84
MRGPRX2	117194	-2.3529	2.09E-11	0.9310345	-2.5812	1.62E-35	0.971153846	-2.174	1.21E-08	0.92
CYP4A11	1579	-2.1134	3.82E-11	0.9310345	-2.6898	1.37E-37	0.961538462	-2.26	8.97E-11	0.84
GABRG1	2565	-2.0952	9.13E-11	0.9310345	NA	NA	NA	NA	NA	NA
PENK	5179	-3.5588	1.09E-10	0.9310345	-4.2817	4.01E-32	0.971153846	-3.64	2.45E-11	0.96
CT62	196993	-3.2392	1.12E-10	0.9310345	-3.9239	1.86E-34	0.951923077	-2.899	1.19E-07	0.8
PAK7	57144	-3.4854	3.99E-10	0.9310345	-3.8541	6.87E-27	0.932692308	-3.621	7.45E-09	0.88
DLK1	8788	-4.2361	9.14E-10	0.9310345	-4.3843	2.43E-17	0.875	-4.803	3.11E-11	0.96
PDZK1	5174	-3.4181	1.08E-09	0.9310345	-3.8523	9.70E-28	0.951923077	-3.161	2.54E-07	0.84
KCNA4	3739	-2.4051	1.44E-09	0.9310345	-2.3992	9.39E-23	0.884615385	-2.643	2.50E-10	0.96
UCP1	7350	-2.2845	1.63E-09	0.9310345	-2.346	2.94E-26	0.971153846	-2.112	1.94E-07	0.88
PROKR1	10887	-2.4479	3.84E-09	0.9310345	-2.3603	7.48E-19	0.932692308	-2.691	5.04E-10	0.96
PHGR1	644844	-2.5457	4.30E-09	0.9310345	-2.623	1.31E-25	0.942307692	-2.23	2.32E-06	0.84
ABCD2	225	-2.704	4.92E-09	0.9310345	-3.4308	2.50E-31	0.980769231	-3.766	6.19E-13	1
MMD	23531	-2.0282	2.13E-08	0.9310345	-2.1961	2.24E-25	0.951923077	-2.252	9.53E-09	0.96
LOC10012897	100128977	-2.1185	2.77E-08	0.9310345	-2.2876	2.17E-24	0.913461538	NA	NA	NA
MYL7	58498	-2.9862	1.10E-07	0.9310345	-2.9929	1.08E-18	0.865384615	-2.991	4.20E-07	0.88
IGFBP1	3484	-2.8849	1.24E-07	0.9310345	-2.8063	6.69E-19	0.942307692	-2.905	4.15E-07	1
NXNL2	158046	-2.1683	3.66E-07	0.9310345	-2.5248	1.78E-20	0.884615385	-2.385	4.45E-07	0.84
PIGR	5284	-4.054	1.34E-06	0.9310345	-4.8853	2.40E-17	0.865384615	-4.748	3.50E-07	0.88
MYOM2	9172	-2.0304	2.73E-06	0.9310345	-2.6632	5.61E-20	0.980769231	-2.002	9.91E-06	0.92
SYNPO2L	79933	-2.0446	1.06E-05	0.9310345	-2.2678	1.22E-17	0.980769231	NA	NA	NA
PRG4	10216	-2.8412	2.86E-05	0.9310345	-3.0952	3.89E-12	0.913461538	-2.461	0.000921499	0.84
RSPO1	284654	-2.1278	9.73E-05	0.9310345	-2.6155	5.12E-14	0.894230769	-2.611	8.28E-06	0.84
HOXA5	3202	-3.4396	5.01E-41	0.8965517	-3.168	6.59E-39	0.711538462	-2.639	2.82E-28	0.72

KL	9365	-3.34	3.34E-40	0.8965517	-3.9768	2.17E-69	0.932692308	-3.197	5.04E-28	0.84
ACOX2	8309	-3.1685	1.82E-36	0.8965517	-3.5857	3.51E-50	0.826923077	-2.333	4.22E-20	0.64
TPO	7173	-4.1742	8.52E-34	0.8965517	-4.6037	6.98E-58	0.913461538	-3.711	3.31E-26	0.88
ABLIM3	22885	-3.5594	2.31E-32	0.8965517	-4.1031	1.65E-61	0.913461538	-3.355	8.54E-29	0.8
SEMA6D	80031	-2.9546	2.15E-31	0.8965517	-3.675	3.63E-57	0.903846154	-3.007	1.46E-26	0.76
ADH1C	126	-5.2958	5.93E-30	0.8965517	-6.4078	1.79E-62	0.951923077	-4.607	3.84E-25	0.92
LRRTM2	26045	-3.2365	2.18E-29	0.8965517	-3.7237	2.42E-60	0.923076923	-3.638	1.09E-31	0.88
MESTIT1	317751	-4.094	2.80E-29	0.8965517	-3.9147	7.83E-50	0.903846154	-3.869	4.74E-25	0.92
CLDN5	7122	-3.3805	3.39E-29	0.8965517	-3.7396	1.12E-52	0.913461538	-2.495	1.78E-15	0.72
GPR81	27198	-3.8418	1.08E-28	0.8965517	-3.8722	2.38E-45	0.865384615	-3.39	6.58E-19	0.72
ABCA6	23460	-3.3916	1.60E-27	0.8965517	-4.3152	2.24E-57	0.932692308	-3.557	1.80E-25	0.88
LOC284276	284276	-3.1369	1.74E-27	0.8965517	-3.2904	2.22E-48	0.855769231	-2.968	4.03E-20	0.8
F10	2159	-3.2885	4.34E-26	0.8965517	-3.8512	1.17E-52	0.942307692	-3.031	1.57E-19	0.72
CXCL2	2920	-3.6437	4.01E-25	0.8965517	-3.4598	9.85E-44	0.875	-2.977	1.76E-16	0.68
LOC145820	145820	-2.7837	1.08E-24	0.8965517	-3.1612	8.89E-47	0.875	-2.274	4.23E-15	0.64
SCUBE2	57758	-3.9489	1.91E-24	0.8965517	-4.9033	1.61E-54	0.913461538	-3.731	6.41E-20	0.84
PHYHIP	9796	-3.1634	3.04E-24	0.8965517	-3.4695	2.99E-52	0.865384615	-2.969	2.24E-24	0.76
HPSE2	60495	-4.0462	4.60E-24	0.8965517	-4.4961	2.00E-48	0.894230769	-4.083	1.09E-21	0.88
MASP1	5648	-4.1682	1.23E-23	0.8965517	-4.28	5.07E-44	0.875	-4.641	1.51E-27	0.92
KCNJ16	3773	-4.7358	2.86E-23	0.8965517	-4.2863	7.02E-31	0.846153846	-4.399	2.87E-18	0.84
STAB2	55576	-4.3844	9.37E-23	0.8965517	-4.9247	1.69E-52	0.971153846	-4.344	1.82E-19	0.92
LUZP2	338645	-4.2288	8.47E-22	0.8965517	-4.5974	5.14E-47	0.942307692	-3.972	6.82E-18	0.8
SNCG	6623	-3.4462	1.23E-21	0.8965517	-3.6642	7.28E-34	0.855769231	-2.542	7.42E-11	0.64
KLF4	9314	-2.788	4.29E-21	0.8965517	-3.0859	4.93E-48	0.942307692	-2.738	1.12E-16	0.8
EBF2	64641	-3.3566	6.14E-21	0.8965517	-3.7934	8.21E-43	0.894230769	-2.995	1.99E-14	0.76
COL6A6	131873	-3.5036	8.36E-21	0.8965517	-4.7493	4.55E-51	0.961538462	-4.251	1.56E-21	0.92
GCOM1	145781	-3.1689	2.20E-20	0.8965517	-3.4298	2.81E-47	0.971153846	-3.296	1.13E-19	0.88
ADAMTS18	170692	-3.4438	3.01E-20	0.8965517	-3.5338	5.29E-32	0.846153846	-3.171	2.96E-13	0.76
COL4A6	1288	-3.8657	9.09E-20	0.8965517	-4.3974	1.59E-41	0.932692308	-3.836	2.65E-16	0.72

MGLL	11343	-2.5866	9.85E-20	0.8965517	-3.0717	1.19E-51	0.932692308	-2.178	2.60E-14	0.76
MUSTN1	389125	-2.7237	8.79E-19	0.8965517	-2.8556	2.90E-41	0.942307692	-2.064	1.16E-10	0.84
CTSG	1511	-3.6363	6.85E-18	0.8965517	-4.0954	2.03E-37	0.903846154	-2.93	4.58E-11	0.68
DMGDH	29958	-3.1431	7.39E-18	0.8965517	-3.1865	9.45E-36	0.865384615	-2.746	1.33E-13	0.84
ODF3L1	161753	-2.5799	1.70E-17	0.8965517	-2.623	3.56E-34	0.807692308	-2.054	1.06E-09	0.72
ANKRD30A	91074	-5.6108	1.74E-17	0.8965517	-7.084	2.10E-48	0.942307692	-5.188	1.23E-12	0.8
MMP28	79148	-2.565	1.84E-17	0.8965517	-3.0587	4.69E-37	0.846153846	NA	NA	NA
CALCR	799	-2.9092	2.37E-17	0.8965517	-2.8256	2.72E-34	0.875	-2.619	1.35E-13	0.88
NIPSNAP3B	55335	-2.2503	8.28E-17	0.8965517	-2.3906	1.36E-38	0.875	-2.808	8.96E-19	0.88
TMEM195	392636	-2.7941	4.95E-16	0.8965517	-3.0256	1.22E-30	0.903846154	-2.293	1.14E-09	0.72
SAMD5	389432	-3.2524	5.68E-16	0.8965517	-3.4869	1.06E-32	0.855769231	-2.815	2.30E-10	0.84
FLNC	2318	-2.4055	7.24E-16	0.8965517	-2.5775	9.99E-26	0.884615385	-2.037	3.67E-11	0.84
GPR109A	338442	-3.012	8.44E-16	0.8965517	-3.2836	5.11E-38	0.903846154	-2.59	4.04E-11	0.76
C14orf139	79686	-2.4912	1.26E-15	0.8965517	-3.0278	9.72E-45	0.932692308	-2.608	7.07E-15	0.92
ALDH3A1	218	-3.181	5.77E-15	0.8965517	-2.4905	8.96E-16	0.788461538	-3.081	8.61E-13	0.96
ADRB1	153	-2.8489	6.43E-15	0.8965517	-2.8577	1.00E-25	0.855769231	-2.082	2.42E-07	0.72
RAB3C	115827	-2.7032	7.99E-15	0.8965517	-2.8009	1.94E-30	0.798076923	-2.292	4.53E-09	0.68
CYP3A5	1577	-2.3462	8.32E-15	0.8965517	-2.4613	8.41E-32	0.884615385	NA	NA	NA
BHMT2	23743	-3.17	8.42E-15	0.8965517	-3.3969	2.84E-28	0.884615385	-2.787	1.98E-10	0.76
TP63	8626	-4.3264	2.00E-14	0.8965517	-4.1623	2.05E-29	0.884615385	-3.202	5.72E-08	0.72
KY	339855	-3.6032	2.01E-14	0.8965517	-4.0461	2.97E-31	0.875	-4.32	5.57E-16	0.92
GPR88	54112	-2.7139	2.04E-14	0.8965517	-2.9764	9.93E-32	0.894230769	-2.665	6.19E-15	0.8
PCDH9	5101	-2.7733	2.78E-14	0.8965517	-3.2212	6.69E-31	0.846153846	-3.761	2.09E-20	0.96
C1orf64	149563	-5.1848	7.67E-14	0.8965517	-5.921	1.46E-39	0.951923077	-4.143	2.62E-08	0.84
RHOXF1	158800	-2.6303	8.35E-14	0.8965517	-2.7272	6.63E-32	0.923076923	-2.907	7.50E-14	0.88
TMEM37	140738	-2.8341	9.58E-14	0.8965517	-2.9323	2.20E-35	0.913461538	-2.499	2.40E-10	0.84
RNASE7	84659	-3.4895	9.58E-14	0.8965517	-3.2231	1.47E-24	0.875	-2.602	2.69E-07	0.68
XPNPEP2	7512	-2.8504	1.27E-13	0.8965517	-3.0901	1.59E-29	0.875	-2.647	7.13E-10	0.84
NEK10	152110	-4.3914	2.38E-13	0.8965517	-4.7939	5.67E-36	0.951923077	-4.146	1.50E-10	0.88

ANKRD30B	374860	-3.6571	3.14E-13	0.8965517	-4.1573	2.45E-36	0.923076923	-3.271	1.05E-09	0.84
CORO6	84940	-2.0412	1.08E-12	0.8965517	NA	NA	NA	NA	NA	NA
ANO3	63982	-3.065	1.48E-12	0.8965517	-3.5937	1.22E-30	0.865384615	-2.803	8.26E-09	0.68
MAS1L	116511	-2.0296	1.83E-12	0.8965517	-2.0683	5.08E-32	0.903846154	NA	NA	NA
HCN1	348980	-2.4973	3.61E-12	0.8965517	-2.3275	4.95E-22	0.875	-2.317	4.54E-10	0.84
SLC5A7	60482	-2.0016	2.11E-10	0.8965517	-2.1263	4.14E-27	0.903846154	NA	NA	NA
SLC26A3	1811	-4.6639	3.46E-10	0.8965517	-5.3518	6.68E-33	0.961538462	-4.521	7.16E-09	0.92
CBLN4	140689	-2.598	5.51E-10	0.8965517	-3.4663	5.47E-30	0.884615385	-2.518	1.52E-07	0.8
AGXT	189	-2.046	1.18E-09	0.8965517	-2.2112	8.75E-27	0.894230769	NA	NA	NA
CYP4F22	126410	-3.5767	1.61E-09	0.8965517	-4.5348	8.35E-29	0.923076923	-3.62	6.84E-08	0.8
CYP2B7P1	1556	-3.0833	1.65E-09	0.8965517	-3.8489	5.84E-29	0.923076923	-2.334	8.64E-06	0.84
EYA4	2070	-2.4172	1.80E-09	0.8965517	NA	NA	NA	-2.52	7.77E-09	0.84
EDN3	1908	-4.0448	2.00E-09	0.8965517	-3.5694	7.19E-16	0.788461538	-3.545	7.77E-07	0.8
DKK4	27121	-2.0075	2.15E-09	0.8965517	NA	NA	NA	NA	NA	NA
LOC10013014[illegible]	100130148	-2.0824	3.94E-09	0.8965517	-2.0578	5.80E-22	0.884615385	NA	NA	NA
SPOCK3	50859	-2.6578	1.16E-08	0.8965517	-2.8826	2.86E-22	0.903846154	-2.8	1.12E-08	0.88
C6	729	-3.4545	1.35E-08	0.8965517	-4.5229	3.48E-27	0.923076923	-3.41	4.56E-08	0.88
PROL1	58503	-4.7165	1.40E-08	0.8965517	-5.3433	4.85E-24	0.971153846	-3.834	2.72E-05	0.8
OVCH2	341277	-2.7397	2.29E-08	0.8965517	-3.3292	5.17E-29	0.942307692	-2.682	4.52E-07	0.88
SYCE1	93426	-2.715	4.98E-08	0.8965517	-3.3306	8.41E-23	0.894230769	-3.012	2.15E-08	0.88
STC2	8614	-2.5824	6.92E-08	0.8965517	-2.1815	3.43E-16	0.855769231	-2.514	9.42E-07	0.84
PROK1	84432	-2.2097	1.00E-06	0.8965517	-2.2294	4.35E-15	0.942307692	NA	NA	NA
LOC728606	728606	-2.6006	5.03E-06	0.8965517	-3.0353	3.41E-18	0.865384615	-2.245	0.000267089	0.76
HPD	3242	-2.5449	6.96E-06	0.8965517	-3.1238	1.81E-19	0.942307692	-2.806	7.80E-07	0.96
KLK3	354	-2.4867	6.29E-05	0.8965517	-3.0642	1.38E-16	0.961538462	-3.166	1.48E-06	0.96
UGT2B28	54490	-2.922	0.000913596	0.8965517	-3.5926	7.37E-13	0.932692308	NA	NA	NA
MAMDC2	256691	-4.3314	2.05E-42	0.862069	-4.8554	1.54E-54	0.903846154	-3.878	5.88E-31	0.8
HOXA7	3204	-3.3616	1.76E-40	0.862069	-2.9207	3.89E-31	0.644230769	-2.506	2.22E-27	0.68
TSLP	85480	-3.8699	4.42E-37	0.862069	-3.5847	3.17E-49	0.836538462	-3.742	4.86E-28	0.8

C1QTNF9	338872	-3.8765	4.10E-34	0.862069	-4.2664	2.49E-69	0.951923077	-3.559	1.43E-28	0.8
HLF	3131	-3.4405	4.39E-34	0.862069	-3.9372	3.94E-58	0.923076923	-3.816	1.89E-32	0.96
MAPT	4137	-3.883	2.10E-33	0.862069	-3.4198	3.99E-41	0.769230769	-2.854	1.66E-19	0.56
C9orf125	84302	-2.7032	6.16E-33	0.862069	-2.6381	2.45E-41	0.75	-2.527	1.93E-24	0.72
ADCY5	111	-4.4087	1.03E-32	0.862069	-5.2629	3.68E-58	0.884615385	-4.401	1.44E-33	0.8
NKAPL	222698	-3.0068	1.20E-32	0.862069	-3.2513	6.95E-68	0.923076923	-2.858	2.67E-31	0.88
CHL1	10752	-4.1689	3.45E-30	0.862069	-4.7583	8.38E-52	0.913461538	-4.469	8.33E-28	0.88
BAI3	577	-3.0001	1.40E-28	0.862069	-3.0026	9.64E-40	0.855769231	-2.914	5.83E-26	0.8
ADAMTS15	170689	-4.0493	1.56E-27	0.862069	-4.1983	1.38E-47	0.903846154	-3.642	4.53E-20	0.68
CCDC158	339965	-3.4611	8.44E-27	0.862069	-3.6215	1.63E-46	0.846153846	-4.014	4.78E-29	0.92
DNALI1	7802	-3.4038	2.17E-26	0.862069	-3.7967	2.61E-42	0.817307692	-3.1	8.52E-19	0.76
CCDC85A	114800	-2.9913	1.85E-25	0.862069	-3.3961	2.52E-47	0.875	-3.065	6.84E-22	0.84
BMPER	168667	-3.6631	2.13E-25	0.862069	-3.9253	7.47E-43	0.855769231	-3.514	2.78E-19	0.76
IGF1	3479	-3.2392	2.90E-25	0.862069	-3.8928	9.22E-44	0.846153846	-3.339	2.96E-20	0.76
MOCS1	4337	-2.4195	3.09E-25	0.862069	-2.4884	2.76E-50	0.807692308	-2.294	7.28E-18	0.72
DARC	2532	-3.6639	5.38E-24	0.862069	-4.9068	9.20E-42	0.855769231	-3.404	2.04E-17	0.8
CLIC5	53405	-2.5256	7.76E-24	0.862069	-2.7453	2.11E-47	0.846153846	-2.482	8.84E-19	0.68
ERBB4	2066	-4.1837	3.31E-23	0.862069	-5.5366	2.43E-46	0.903846154	-4.543	2.86E-19	0.8
HSPB8	26353	-2.7323	6.44E-23	0.862069	-2.9232	1.95E-31	0.788461538	-2.644	2.55E-18	0.76
MFAP4	4239	-3.1589	1.63E-22	0.862069	-4.2109	1.80E-49	0.932692308	-3.416	8.90E-20	0.8
PDE11A	50940	-3.5202	2.13E-22	0.862069	-4.5855	4.16E-57	0.932692308	-4.095	2.29E-25	0.84
RELN	5649	-3.8796	1.38E-20	0.862069	-4.8599	9.90E-41	0.903846154	-4.198	9.18E-23	0.84
ABCC6	368	-3.3009	1.59E-20	0.862069	-3.5455	3.68E-40	0.884615385	-2.71	8.93E-13	0.68
PTGER3	5733	-3.2854	1.65E-20	0.862069	-4.3915	3.01E-55	0.971153846	-3.659	1.51E-20	0.88
PREX2	80243	-3.7674	4.57E-20	0.862069	-4.3658	2.52E-41	0.923076923	-3.819	9.08E-18	0.88
PTH1R	5745	-2.8882	6.90E-19	0.862069	-3.4391	4.39E-40	0.884615385	-2.791	1.52E-15	0.84
CYP4B1	1580	-4.238	1.58E-18	0.862069	-5.2432	1.70E-46	0.942307692	-3.313	2.06E-12	0.84
NMNAT2	23057	-2.636	1.54E-16	0.862069	-2.6709	2.51E-27	0.740384615	-2.601	3.71E-16	0.76
ACADL	33	-3.8217	4.84E-16	0.862069	-4.1155	9.49E-30	0.855769231	-4.008	3.28E-15	0.8

GPR133	283383	-2.8976	7.39E-16	0.862069	-3.2739	5.82E-33	0.846153846	-3.132	6.29E-14	0.76
C8orf34	116328	-3.0185	8.59E-16	0.862069	-3.3891	6.67E-38	0.903846154	-3.169	3.52E-14	0.84
ABCC9	10060	-2.4948	1.79E-14	0.862069	-2.9266	7.20E-34	0.846153846	-2.006	6.06E-09	0.72
GRIK5	2901	-2.6412	2.56E-14	0.862069	-2.2327	3.14E-20	0.730769231	-2.1	2.44E-08	0.6
ART4	420	-2.5876	5.80E-14	0.862069	-3.0834	1.66E-32	0.875	-2.452	1.12E-11	0.68
SHISA3	152573	-2.4988	6.46E-14	0.862069	-2.1276	2.04E-17	0.711538462	NA	NA	NA
KLHL4	56062	-2.5625	7.69E-14	0.862069	-2.9126	1.06E-30	0.836538462	-2.218	2.10E-09	0.76
ALX4	60529	-3.2981	9.56E-14	0.862069	-3.1177	2.27E-24	0.826923077	-3.101	2.88E-11	0.84
PSD2	84249	-2.8495	1.10E-13	0.862069	-2.5592	4.28E-22	0.826923077	-2.101	2.80E-07	0.72
ERVFRDE1	405754	-2.3726	1.42E-13	0.862069	-2.6173	5.72E-34	0.913461538	-2.212	1.97E-10	0.8
TTC36	143941	-3.3269	1.59E-13	0.862069	-3.872	6.49E-34	0.923076923	-2.537	1.40E-07	0.72
ODZ2	57451	-3.4847	1.70E-13	0.862069	-3.7565	3.35E-29	0.855769231	-3.537	9.46E-11	0.8
RNF186	54546	-3.3137	3.08E-12	0.862069	-3.6044	2.03E-29	0.903846154	-3.696	2.54E-13	0.84
TCEAL5	340543	-2.2722	3.57E-12	0.862069	-2.4696	1.77E-27	0.836538462	-2.1	3.80E-09	0.64
CXCR7	57007	-2.2333	4.61E-12	0.862069	-2.3666	6.25E-31	0.855769231	NA	NA	NA
GRIK3	2899	-3.5213	5.90E-12	0.862069	-4.1301	3.42E-28	0.875	-2.819	1.24E-07	0.72
LMX1A	4009	-2.7864	7.75E-12	0.862069	-2.8347	1.25E-23	0.855769231	-2.623	2.98E-09	0.88
SLC26A5	375611	-2.3409	8.45E-12	0.862069	-2.3611	2.91E-26	0.884615385	-2.28	2.89E-10	0.84
SAA1	6288	-2.9844	9.56E-12	0.862069	-3.4934	1.45E-26	0.846153846	-3.593	1.34E-13	0.92
SLC4A4	8671	-2.7126	1.42E-11	0.862069	-3.358	3.42E-35	0.961538462	-3.081	2.34E-11	0.84
PCOLCE2	26577	-3.5888	5.98E-11	0.862069	-3.5328	4.99E-24	0.913461538	-3.5	2.61E-10	0.96
DCT	1638	-2.4892	1.04E-10	0.862069	-3.1485	2.29E-38	0.951923077	-2.249	7.64E-08	0.8
FAM3B	54097	-3.1183	1.21E-10	0.862069	-2.652	8.09E-15	0.730769231	-2.296	2.99E-05	0.64
GPX2	2877	-3.501	1.48E-10	0.862069	-3.5752	4.02E-20	0.865384615	-2.702	4.37E-06	0.72
CDH20	28316	-2.7132	1.54E-10	0.862069	-3.0989	3.50E-29	0.942307692	-3.272	7.73E-13	0.92
TAC1	6863	-3.1934	1.60E-10	0.862069	-3.7767	1.98E-30	0.932692308	-3.603	2.97E-11	0.88
GPBAR1	151306	-2.262	5.95E-10	0.862069	-2.2564	8.72E-24	0.826923077	NA	NA	NA
FSIP1	161835	-2.3483	6.44E-10	0.862069	-2.6516	7.72E-25	0.884615385	-2.16	1.09E-07	0.76
SCGB1D2	10647	-4.9694	6.53E-10	0.862069	-6.8934	2.21E-37	0.980769231	-4.56	3.85E-08	0.92

CAPZA3	93661	-2.3942	7.25E-10	0.862069	-2.4129	4.29E-21	0.769230769	NA	NA	NA
KIAA1239	57495	-3.18	1.07E-09	0.862069	-3.4296	5.67E-24	0.932692308	-3.099	1.70E-08	0.92
SERPINA11	256394	-3.7104	1.44E-09	0.862069	-4.0931	2.91E-25	0.913461538	-3.478	2.75E-07	0.84
DCDC2	51473	-3.4676	2.17E-09	0.862069	-3.3758	5.56E-19	0.846153846	-3.236	5.07E-07	0.8
LILRB5	10990	-2.4157	4.58E-09	0.862069	-2.0465	3.06E-15	0.769230769	NA	NA	NA
SCGB2A2	4250	-4.968	2.84E-08	0.862069	-7.4619	6.04E-35	0.980769231	-4.42	2.26E-06	0.84
FER1L5	90342	-2.4135	5.96E-08	0.862069	-2.7335	1.46E-23	0.923076923	-2.813	3.81E-09	0.88
PGLYRP2	114770	-2.8253	6.44E-08	0.862069	-3.1369	2.16E-21	0.884615385	-2.223	7.23E-05	0.8
PTN	5764	-2.784	1.08E-07	0.862069	-3.5059	1.35E-22	0.817307692	-3.094	9.81E-08	0.72
GRIA2	2891	-2.6822	1.66E-07	0.862069	-2.187	8.40E-10	0.711538462	-2.723	7.05E-08	0.68
CALML3	810	-3.3287	1.77E-07	0.862069	-3.0965	7.83E-12	0.807692308	-2.189	0.002295366	0.64
UGT2B15	7366	-3.3793	2.22E-07	0.862069	-4.1069	1.32E-24	0.961538462	-3.089	1.05E-05	0.92
SYTL5	94122	-2.9728	7.35E-07	0.862069	-3.9292	4.66E-24	0.884615385	NA	NA	NA
NLRP5	126206	-2.5996	1.19E-06	0.862069	-3.3264	2.70E-27	0.942307692	-2.869	6.37E-07	0.88
UGT2B11	10720	-4.2703	2.91E-06	0.862069	-5.7191	1.12E-25	0.961538462	-3.681	0.000158773	0.84
ABCC11	85320	-2.7116	0.000174313	0.862069	-4.2711	2.83E-18	0.913461538	NA	NA	NA
MUCL1	118430	-3.947	0.000189031	0.862069	-6.1504	8.85E-20	0.913461538	-3.158	0.005477845	0.72
LDB2	9079	-2.6045	4.28E-38	0.8275862	-2.9861	9.90E-65	0.826923077	-2.282	9.88E-23	0.6
LHFP	10186	-2.4765	2.83E-37	0.8275862	-2.8927	2.55E-63	0.855769231	-2.174	1.64E-24	0.72
PEAR1	375033	-2.7657	8.64E-37	0.8275862	-3.1147	1.51E-55	0.826923077	-2.508	4.06E-26	0.72
GPRASP1	9737	-2.7057	5.21E-36	0.8275862	-3.0169	4.09E-56	0.826923077	-2.691	7.32E-29	0.76
GIPC2	54810	-3.4347	7.93E-36	0.8275862	-3.6319	4.93E-52	0.836538462	-3.371	3.87E-32	0.84
NTRK2	4915	-4.2586	1.90E-34	0.8275862	-4.5558	6.61E-57	0.884615385	-4.747	4.09E-36	0.88
RAPGEF3	10411	-2.9132	4.10E-34	0.8275862	-3.3631	9.29E-69	0.875	-2.323	6.23E-23	0.6
TCEAL7	56849	-3.0276	5.07E-34	0.8275862	-3.453	1.27E-64	0.951923077	-3.04	1.60E-27	0.8
TSPAN7	7102	-2.9656	1.45E-33	0.8275862	-3.3865	5.34E-51	0.826923077	-2.912	8.24E-25	0.8
LOC134466	134466	-2.9274	6.30E-32	0.8275862	-3.3228	2.05E-50	0.875	-3.194	6.68E-27	0.84
MYRIP	25924	-3.6667	1.49E-31	0.8275862	-4.3575	1.18E-48	0.855769231	-4.235	1.30E-28	0.76
CD34	947	-2.4209	4.79E-30	0.8275862	-2.6352	4.91E-57	0.798076923	-2.05	1.24E-19	0.52

RBMS3	27303	-3.1863	6.93E-30	0.8275862	-3.2965	2.93E-50	0.846153846	-2.888	3.66E-25	0.8
PCDH19	57526	-3.0251	8.47E-30	0.8275862	-3.3784	1.26E-46	0.865384615	-3.146	1.53E-27	0.76
ZBTB16	7704	-5.4404	3.43E-29	0.8275862	-6.5004	1.61E-64	0.942307692	-5.654	2.45E-29	0.88
HOXA6	3203	-3.4585	5.39E-29	0.8275862	-2.9152	2.56E-31	0.682692308	-2.679	2.25E-20	0.72
LRRC70	100130733	-2.6059	9.15E-29	0.8275862	-2.9404	5.76E-57	0.817307692	-2.223	4.31E-18	0.6
ADRB2	154	-2.9763	3.17E-27	0.8275862	-3.8742	2.51E-52	0.875	-3.327	2.16E-26	0.76
DUSP1	1843	-2.8907	1.95E-26	0.8275862	-2.9735	8.27E-49	0.836538462	-2.356	1.36E-16	0.56
C4orf31	79625	-3.3038	6.02E-26	0.8275862	-3.9822	1.22E-42	0.875	-4.047	3.57E-26	0.8
ENPP2	5168	-3.054	6.22E-26	0.8275862	-3.1175	1.95E-41	0.788461538	-2.38	1.30E-18	0.68
ALDH1A1	216	-3.0819	7.19E-26	0.8275862	-3.3188	4.85E-48	0.875	-2.827	1.82E-22	0.68
DACT2	168002	-3.727	7.60E-26	0.8275862	-3.3871	6.62E-30	0.75	-3.019	1.22E-17	0.76
C2orf40	84417	-4.9617	7.18E-25	0.8275862	-5.4662	1.79E-51	0.923076923	-4.953	3.03E-28	0.84
FAM149A	25854	-2.6509	1.17E-24	0.8275862	-2.4898	1.39E-34	0.673076923	-2.583	3.79E-19	0.64
C1QTNF9B	387911	-2.8256	1.40E-24	0.8275862	-3.0782	2.50E-44	0.865384615	-3.014	2.58E-24	0.92
MEOX1	4222	-2.9641	6.84E-24	0.8275862	-3.7208	2.72E-42	0.826923077	-3.119	5.34E-24	0.76
LOC285419	285419	-2.3162	1.19E-23	0.8275862	-2.4079	1.55E-35	0.826923077	NA	NA	NA
NLGN1	22871	-3.3519	8.24E-23	0.8275862	-3.4755	1.61E-36	0.817307692	-3.788	2.80E-20	0.76
WSCD2	9671	-3.1003	4.65E-22	0.8275862	-3.6985	3.74E-36	0.826923077	-3.757	6.78E-28	0.88
GAS2L2	246176	-2.9868	4.99E-22	0.8275862	-3.2607	3.81E-33	0.817307692	-2.898	2.49E-19	0.76
CLSTN2	64084	-3.396	8.03E-22	0.8275862	-4.0753	7.04E-46	0.903846154	-3.324	3.62E-19	0.84
P2RY12	64805	-3.0164	1.34E-21	0.8275862	-4.0259	5.64E-50	0.875	-3.532	1.57E-22	0.88
SLC10A6	345274	-2.8176	1.35E-21	0.8275862	-2.7471	6.85E-36	0.826923077	NA	NA	NA
SLC7A4	6545	-3.5174	2.14E-21	0.8275862	-3.5446	1.16E-30	0.75	-3.921	5.19E-24	0.8
PLCXD3	345557	-3.8383	6.46E-21	0.8275862	-4.3771	8.65E-42	0.903846154	-3.596	1.28E-16	0.8
AMIGO2	347902	-2.7867	1.06E-20	0.8275862	-2.874	2.74E-38	0.817307692	-2.286	1.05E-11	0.64
DPP6	1804	-3.7834	1.13E-20	0.8275862	-4.3462	1.33E-40	0.875	-4.704	6.32E-31	0.96
LRFN5	145581	-2.4494	1.95E-20	0.8275862	-3.284	8.55E-40	0.875	-2.727	6.02E-19	0.72
AOX1	316	-3.2507	4.15E-20	0.8275862	-3.8683	2.36E-43	0.894230769	-3.678	1.98E-20	0.8
MYH7B	57644	-2.2528	1.96E-19	0.8275862	-2.2129	8.05E-33	0.865384615	-2.22	3.53E-16	0.8

SLC1A7	6512	-2.7765	4.38E-19	0.8275862	-3.0313	8.98E-37	0.817307692	-2.353	9.48E-13	0.68
NRXN1	9378	-3.3228	7.46E-19	0.8275862	-3.1892	5.52E-26	0.788461538	-2.72	2.10E-11	0.6
GFAP	2670	-3.4433	1.20E-17	0.8275862	-4.2249	5.36E-44	0.932692308	-4.311	1.02E-20	0.84
TCF21	6943	-2.331	1.41E-16	0.8275862	-2.4288	3.69E-32	0.865384615	NA	NA	NA
TMEM100	55273	-3.3644	1.65E-16	0.8275862	-3.6182	1.85E-28	0.788461538	-2.881	9.63E-14	0.8
EMX2OS	196047	-2.5471	3.61E-16	0.8275862	-2.9542	1.02E-32	0.817307692	-2.866	7.24E-15	0.84
TPPP3	51673	-2.2678	4.51E-16	0.8275862	-2.29	1.48E-26	0.788461538	NA	NA	NA
AKR1C1	1645	-3.4006	9.49E-16	0.8275862	-3.9124	5.13E-35	0.913461538	-3.066	1.60E-11	0.76
LRP1B	53353	-3.1494	1.11E-15	0.8275862	-3.7738	1.21E-34	0.826923077	-3.531	1.89E-17	0.76
DMRT2	10655	-2.9272	1.26E-15	0.8275862	-2.8507	3.01E-24	0.759615385	-3.275	3.45E-16	0.88
C10orf82	143379	-3.1106	3.03E-15	0.8275862	-3.3164	1.95E-29	0.875	-3.295	2.99E-15	0.88
ASTN1	460	-2.928	4.03E-15	0.8275862	-2.9455	4.01E-22	0.75	-3.014	1.81E-11	0.72
SLC14A1	6563	-2.4045	5.52E-15	0.8275862	-2.4747	1.19E-25	0.730769231	-2.411	4.74E-14	0.76
PFKFB3	5209	-2.106	9.58E-15	0.8275862	-2.0744	1.60E-32	0.721153846	-2.28	1.46E-14	0.76
ZFP36	7538	-2.2761	1.28E-14	0.8275862	-2.5906	9.11E-38	0.855769231	NA	NA	NA
CADM2	253559	-2.8181	3.20E-14	0.8275862	-3.4151	1.17E-32	0.836538462	-3.435	4.93E-18	0.8
IGSF1	3547	-3.7167	3.93E-14	0.8275862	-3.3776	2.11E-21	0.846153846	-4.261	1.15E-14	0.88
SLC25A18	83733	-2.4163	7.53E-14	0.8275862	-2.7827	8.55E-35	0.884615385	-2.21	3.48E-11	0.8
SCUBE1	80274	-2.795	3.39E-13	0.8275862	-3.0422	7.03E-25	0.798076923	-3.012	6.99E-13	0.8
CLEC4GP1	440508	-2.6244	4.84E-13	0.8275862	-2.7136	6.37E-32	0.894230769	-2.482	1.85E-10	0.84
AOC2	314	-2.2761	6.10E-13	0.8275862	-2.2105	7.60E-26	0.807692308	-2.509	1.91E-13	0.84
HSPA12A	259217	-2.2852	7.98E-13	0.8275862	-2.5463	5.32E-32	0.826923077	-2.516	1.18E-13	0.8
CLDND2	125875	-2.2981	1.79E-12	0.8275862	-2.3335	8.78E-28	0.759615385	-2.063	1.05E-10	0.76
CDH22	64405	-2.4106	2.75E-12	0.8275862	-2.1025	5.07E-15	0.769230769	-2.218	2.18E-09	0.68
SPINK5	11005	-3.0015	2.95E-12	0.8275862	-2.1293	5.43E-10	0.701923077	NA	NA	NA
CHAD	1101	-3.3189	3.98E-12	0.8275862	-3.6644	1.43E-31	0.903846154	-3.364	4.29E-10	0.76
KLHDC7A	127707	-3.5697	6.19E-12	0.8275862	-4.7746	3.01E-33	0.884615385	-3.339	7.47E-10	0.76
SLCO1C1	53919	-2.1027	1.15E-11	0.8275862	-2.7053	2.02E-34	0.875	NA	NA	NA
NR0B1	190	-2.2551	1.28E-11	0.8275862	NA	NA	NA	NA	NA	NA

PKDCC	91461	-2.0849	1.76E-11	0.8275862	NA	NA	NA	NA	NA	NA
C1orf173	127254	-3.3195	4.61E-11	0.8275862	-3.567	3.59E-26	0.894230769	-3.708	1.28E-10	0.88
CLCA4	22802	-3.6323	6.88E-11	0.8275862	-3.6695	2.06E-24	0.846153846	-3.344	2.52E-08	0.76
MOSC1	64757	-2.2402	8.30E-11	0.8275862	-2.4329	7.35E-28	0.855769231	-2.523	7.30E-11	0.88
SLC16A7	9194	-2.6221	2.30E-10	0.8275862	-3.1243	3.17E-31	0.932692308	-3.245	1.65E-13	0.92
MYEOV	26579	-2.8701	3.80E-10	0.8275862	-2.2204	2.51E-10	0.701923077	-2.643	1.72E-07	0.8
LYPD6	130574	-2.6927	9.04E-10	0.8275862	-2.8591	7.44E-23	0.807692308	-2.994	4.61E-10	0.8
IGDCC3	9543	-3.1297	1.76E-09	0.8275862	-3.6164	7.68E-29	0.932692308	-2.762	1.61E-07	0.76
IL1RAPL2	26280	-2.5702	1.98E-09	0.8275862	-2.6692	1.67E-20	0.826923077	NA	NA	NA
MYO16	23026	-2.4077	3.34E-09	0.8275862	-2.2064	8.82E-16	0.75	-2.889	3.54E-11	0.88
SYT9	143425	-2.7502	3.52E-09	0.8275862	-3.598	5.53E-29	0.894230769	-3.444	2.80E-12	0.84
SAA2	6289	-2.9465	3.61E-09	0.8275862	-3.4485	6.24E-21	0.836538462	-3.779	1.20E-12	0.88
KLHL31	401265	-2.2332	4.20E-09	0.8275862	-3.0086	1.75E-36	0.971153846	-3.086	1.07E-12	0.92
C13orf36	400120	-2.4094	6.02E-09	0.8275862	-2.6778	9.32E-24	0.846153846	-3.192	3.87E-13	0.96
DLK2	65989	-2.6691	1.07E-08	0.8275862	-2.3414	7.26E-16	0.75	-2.142	3.30E-05	0.6
PTHLH	5744	-2.1524	3.63E-08	0.8275862	-2.3709	3.63E-19	0.875	-2.023	1.78E-05	0.72
WISP2	8839	-2.2822	4.03E-08	0.8275862	-2.8125	8.03E-22	0.817307692	-2.412	6.13E-07	0.76
AREG	374	-2.3752	1.42E-07	0.8275862	-2.6948	1.24E-18	0.817307692	NA	NA	NA
IL6	3569	-2.4217	3.39E-07	0.8275862	-2.143	2.35E-13	0.817307692	NA	NA	NA
EGF	1950	-3.1757	4.06E-07	0.8275862	-2.6206	2.10E-11	0.75	NA	NA	NA
PIP	5304	-5.1326	5.42E-07	0.8275862	-6.7443	1.03E-23	0.913461538	-4.97	4.83E-06	0.84
NTF4	4909	-2.3883	4.88E-06	0.8275862	-2.6032	5.43E-15	0.778846154	-2.244	8.89E-05	0.72
SCGN	10590	-2.3822	5.40E-06	0.8275862	-3.4347	3.90E-23	0.923076923	-2.891	5.35E-08	0.84
GJB1	2705	-2.4215	1.50E-05	0.8275862	-2.5436	4.09E-11	0.807692308	NA	NA	NA
PDGFD	80310	-2.923	3.28E-40	0.7931034	-3.3699	1.08E-72	0.884615385	-2.797	1.13E-30	0.72
SH3BGRL2	83699	-2.7812	4.45E-39	0.7931034	-2.7221	2.76E-49	0.673076923	-2.798	1.41E-28	0.68
FAM47E	100129583	-3.1947	3.16E-37	0.7931034	-3.551	2.24E-59	0.855769231	-2.885	1.77E-30	0.68
LIFR	3977	-3.4438	3.70E-36	0.7931034	-3.437	1.07E-49	0.836538462	-3.334	3.33E-29	0.8
NOSTRIN	115677	-3.0621	8.18E-36	0.7931034	-3.7864	3.30E-61	0.855769231	-2.877	3.68E-23	0.72

CORO2B	10391	-2.8179	3.28E-35	0.7931034	-3.4343	3.82E-51	0.836538462	-2.703	8.46E-28	0.8
FAM13C	220965	-2.8701	5.88E-34	0.7931034	-3.1877	1.71E-51	0.836538462	-2.813	1.64E-26	0.8
SCN3B	55800	-2.9823	2.66E-32	0.7931034	-3.5539	3.45E-52	0.865384615	-2.663	2.18E-24	0.68
TPPP	11076	-3.1313	3.82E-31	0.7931034	-3.1103	9.82E-50	0.769230769	-2.34	1.07E-21	0.6
USHBP1	83878	-2.739	5.68E-31	0.7931034	-3.0509	6.10E-56	0.846153846	-2.063	6.53E-18	0.6
MMRN2	79812	-2.476	2.14E-30	0.7931034	-2.7344	6.66E-56	0.798076923	-2.01	4.15E-18	0.52
TDRD10	126668	-2.7642	1.37E-29	0.7931034	-2.9304	2.56E-43	0.75	NA	NA	NA
DLGAP2	9228	-2.7163	1.70E-29	0.7931034	-2.9055	3.63E-42	0.807692308	-2.871	1.27E-28	0.84
SRPX	8406	-2.6755	1.34E-28	0.7931034	-2.8735	8.33E-48	0.817307692	-2.386	1.55E-17	0.56
IL33	90865	-3.3084	1.48E-28	0.7931034	-4.4241	7.72E-53	0.884615385	-3.349	2.12E-18	0.72
TSHZ2	128553	-3.052	2.86E-27	0.7931034	-3.4852	2.48E-48	0.855769231	-3.148	9.65E-24	0.76
STK32B	55351	-2.7107	5.33E-27	0.7931034	-3.406	9.17E-50	0.884615385	-2.333	1.76E-20	0.72
HOXA9	3205	-2.862	1.84E-25	0.7931034	-2.6045	4.98E-24	0.711538462	NA	NA	NA
ABCG2	9429	-2.8431	2.42E-24	0.7931034	-3.2422	2.80E-52	0.855769231	-2.395	5.71E-20	0.68
DNASE1L3	1776	-3.3525	6.11E-24	0.7931034	-4.1082	1.91E-42	0.846153846	-3.282	2.48E-19	0.72
SLC7A2	6542	-3.3745	6.18E-24	0.7931034	-3.9625	1.47E-46	0.865384615	-2.444	1.82E-14	0.64
HIF3A	64344	-3.8517	6.19E-24	0.7931034	-3.8127	1.13E-37	0.807692308	-3.701	5.82E-21	0.76
THSD7B	80731	-3.1771	2.19E-23	0.7931034	-3.7785	9.82E-45	0.865384615	-3.495	2.17E-21	0.76
DOC2B	8447	-3.2226	6.71E-23	0.7931034	-2.9503	2.22E-30	0.682692308	-3.2	7.48E-20	0.68
ITPR1	3708	-2.8407	7.83E-23	0.7931034	-3.0817	3.25E-52	0.817307692	-2.779	4.47E-22	0.72
LAMA3	3909	-3.5538	1.01E-22	0.7931034	-3.639	8.52E-41	0.865384615	-3.006	2.96E-16	0.76
COL25A1	84570	-2.9681	1.29E-22	0.7931034	-2.838	1.97E-27	0.769230769	-3.12	4.23E-19	0.72
LOC286367	286367	-2.0843	1.44E-22	0.7931034	-2.2572	8.39E-47	0.730769231	-2.638	6.10E-26	0.84
NTN4	59277	-2.8184	1.46E-22	0.7931034	-3.5361	4.47E-53	0.894230769	-2.703	4.63E-16	0.72
EHD2	30846	-2.3667	1.57E-22	0.7931034	-2.5508	7.57E-44	0.740384615	NA	NA	NA
CAV2	858	-2.4675	2.18E-22	0.7931034	-2.7035	9.88E-45	0.75	-2.209	3.30E-17	0.68
LPHN3	23284	-3.1966	3.67E-22	0.7931034	-3.377	6.90E-27	0.759615385	-3.192	1.62E-15	0.76
GPER	2852	-2.4066	1.84E-21	0.7931034	-2.397	8.65E-36	0.721153846	NA	NA	NA
FMO2	2327	-2.9081	2.77E-21	0.7931034	-3.7323	7.71E-42	0.826923077	-3.254	2.04E-23	0.68

GSN	2934	-2.1463	4.29E-21	0.7931034	-2.2761	5.20E-48	0.788461538	-2.018	7.36E-17	0.64
CABP1	9478	-2.8406	6.19E-21	0.7931034	-2.5706	6.36E-35	0.788461538	-2.111	2.35E-13	0.8
GPR109B	8843	-2.7531	7.84E-21	0.7931034	-2.8603	9.45E-42	0.817307692	-2.44	6.65E-15	0.6
GRM7	2917	-2.6399	1.64E-20	0.7931034	-2.5621	8.33E-35	0.817307692	-2.18	6.22E-13	0.76
CCDC36	339834	-2.5525	3.40E-19	0.7931034	-2.7766	5.05E-44	0.894230769	-2.542	3.81E-17	0.84
SCN9A	6335	-2.5955	7.33E-17	0.7931034	-2.6531	4.93E-34	0.836538462	-2.207	4.43E-13	0.68
ANKRD53	79998	-2.4335	2.17E-16	0.7931034	-2.2006	4.25E-26	0.692307692	-2.569	1.49E-14	0.72
TBX15	6913	-2.2271	2.24E-16	0.7931034	-2.5962	1.86E-32	0.807692308	-2.418	9.02E-16	0.76
LDHD	197257	-2.8791	3.61E-16	0.7931034	-3.5364	5.55E-38	0.855769231	-2.277	6.83E-12	0.76
CCDC69	26112	-2.5687	4.97E-16	0.7931034	-2.9097	3.98E-38	0.855769231	-2.745	5.02E-16	0.8
AKR1C3	8644	-2.4131	9.03E-16	0.7931034	-2.7507	1.02E-25	0.692307692	NA	NA	NA
STBD1	8987	-2.0885	1.43E-15	0.7931034	-2.2597	4.30E-34	0.788461538	-2.038	3.98E-13	0.72
PDZD2	23037	-2.3767	1.49E-15	0.7931034	-2.7171	2.13E-36	0.778846154	-2.509	6.27E-15	0.72
ABCC6P1	653190	-2.6516	1.72E-15	0.7931034	-2.8944	3.79E-35	0.846153846	-2.66	1.94E-13	0.72
TFF3	7033	-4.2414	1.83E-15	0.7931034	-5.1042	1.48E-32	0.894230769	-2.996	2.86E-08	0.64
C21orf15	54094	-3.0413	2.15E-15	0.7931034	-3.0911	4.44E-30	0.836538462	-3.157	2.18E-15	0.8
CNTFR	1271	-3.0769	4.17E-15	0.7931034	-3.776	1.05E-27	0.778846154	-2.454	1.95E-08	0.64
HAS3	3038	-3.1251	4.17E-15	0.7931034	-3.2447	1.12E-32	0.865384615	-2.706	5.40E-10	0.76
GPLD1	2822	-2.9883	6.05E-15	0.7931034	-2.618	7.97E-26	0.788461538	-2.77	1.82E-11	0.8
TESC	54997	-2.6798	1.05E-14	0.7931034	-2.918	1.79E-32	0.836538462	-2.28	1.38E-11	0.68
BCHE	590	-2.7213	5.85E-14	0.7931034	-2.953	1.96E-24	0.75	-3.187	4.49E-16	0.8
LOC440173	440173	-2.3708	7.30E-14	0.7931034	-2.2446	1.06E-20	0.788461538	NA	NA	NA
C11orf53	341032	-2.5286	8.40E-14	0.7931034	-2.2588	6.72E-17	0.721153846	NA	NA	NA
RNF157	114804	-2.4434	1.14E-13	0.7931034	-2.797	5.45E-30	0.817307692	-2.717	2.48E-15	0.88
SOX5	6660	-2.4949	1.85E-13	0.7931034	-2.9358	6.45E-29	0.778846154	-3.434	1.08E-19	0.88
MT1A	4489	-2.7224	1.87E-13	0.7931034	-2.608	3.36E-21	0.759615385	-2.539	7.36E-12	0.8
HTR2A	3356	-2.9045	3.16E-13	0.7931034	-2.5809	2.59E-19	0.75	-3.191	5.72E-14	0.8
ABCC8	6833	-4.1327	8.70E-13	0.7931034	-4.7023	5.06E-34	0.913461538	-3.504	1.01E-08	0.8
CDKL2	8999	-2.9083	1.43E-12	0.7931034	-2.73	1.60E-21	0.759615385	-2.974	5.77E-12	0.76

CYP4X1	260293	-3.1273	2.38E-12	0.7931034	-3.4304	6.90E-24	0.730769231	-2.761	6.35E-09	0.72
PKD1L2	114780	-2.9529	2.62E-12	0.7931034	-3.2119	4.81E-28	0.865384615	-3.428	1.12E-14	0.92
DRD1	1812	-2.3087	6.19E-12	0.7931034	-2.5266	7.12E-28	0.798076923	-2.191	5.75E-10	0.64
EFCAB1	79645	-3.0358	1.02E-11	0.7931034	-3.363	1.31E-25	0.826923077	-3.167	1.03E-10	0.76
FAM166B	730112	-2.3486	1.35E-11	0.7931034	-2.4725	4.76E-23	0.865384615	-2.518	2.38E-11	0.84
EGR2	1959	-2.1694	1.59E-11	0.7931034	-2.4766	3.62E-28	0.807692308	-2.196	7.14E-10	0.76
TSPAN8	7103	-2.7487	1.76E-11	0.7931034	-2.5856	1.03E-12	0.644230769	NA	NA	NA
LPPR1	54886	-2.8602	2.29E-11	0.7931034	-3.3684	4.64E-27	0.836538462	-3.092	4.91E-11	0.76
TFPI2	7980	-2.684	9.47E-11	0.7931034	-3.0534	2.33E-22	0.865384615	-2.564	3.05E-08	0.8
IL17B	27190	-3.2206	1.22E-10	0.7931034	-3.5166	5.14E-26	0.875	-3.186	3.12E-10	0.8
THSD4	79875	-2.4118	1.29E-10	0.7931034	-3.2677	7.57E-33	0.865384615	-2.711	5.04E-11	0.76
C13orf33	84935	-2.2094	2.22E-10	0.7931034	-2.4928	1.30E-23	0.759615385	NA	NA	NA
CLIC6	54102	-2.0611	3.12E-10	0.7931034	-2.6724	2.20E-24	0.836538462	-2.423	2.90E-10	0.76
PHF21B	112885	-2.2811	6.09E-10	0.7931034	NA	NA	NA	NA	NA	NA
CNTN6	27255	-2.0614	7.67E-10	0.7931034	-2.339	3.31E-22	0.788461538	-2.167	8.67E-10	0.72
GREB1	9687	-2.3717	8.22E-10	0.7931034	-2.6245	3.49E-23	0.836538462	-2.208	2.15E-07	0.68
LOC145837	145837	-2.824	2.55E-09	0.7931034	-3.1993	3.44E-25	0.913461538	-2.257	3.32E-06	0.72
DPY19L2P4	442523	-2.3757	4.32E-09	0.7931034	-2.3477	4.17E-18	0.759615385	-2.761	1.06E-09	0.88
DRD2	1813	-2.5099	7.08E-09	0.7931034	-2.2981	1.96E-14	0.663461538	-2.003	2.89E-05	0.64
SPHKAP	80309	-3.2372	2.69E-08	0.7931034	-2.5653	8.76E-11	0.692307692	-4.134	5.69E-12	0.92
TMEM26	219623	-2.0982	3.77E-08	0.7931034	-2.409	1.08E-23	0.836538462	-2.082	2.60E-07	0.8
BCAS1	8537	-2.4179	8.53E-08	0.7931034	-2.9651	2.45E-20	0.769230769	NA	NA	NA
SPATA22	84690	-2.0616	1.32E-07	0.7931034	NA	NA	NA	-2.01	1.76E-06	0.8
PIK3C2G	5288	-3.0856	4.78E-07	0.7931034	-3.5001	1.45E-16	0.759615385	-2.24	0.000737178	0.52
C2CD4B	388125	-2.1679	1.01E-06	0.7931034	-2.3391	2.48E-14	0.836538462	NA	NA	NA
FLJ45983	399717	-2.3683	1.11E-06	0.7931034	-2.4001	6.22E-14	0.75	NA	NA	NA
DSC1	1823	-2.4774	1.23E-06	0.7931034	-2.9383	2.89E-20	0.875	-2.392	1.03E-05	0.76
FCN2	2220	-2.1313	1.35E-05	0.7931034	-3.2209	3.40E-25	0.875	-2.588	4.63E-08	0.84
NRG1	3084	-2.079	6.09E-05	0.7931034	-2.7717	8.85E-15	0.778846154	-2.659	7.68E-06	0.68

LOC642587	642587	-2.7497	7.34E-05	0.7931034	-2.2812	7.75E-08	0.721153846	NA	NA	NA
AGR2	10551	-2.8921	0.000144539	0.7931034	-3.9318	6.12E-15	0.865384615	NA	NA	NA
JAM2	58494	-2.6427	7.26E-43	0.7586207	-3.0302	2.98E-60	0.798076923	-2.07	5.82E-24	0.64
HOXA4	3201	-2.8389	2.90E-37	0.7586207	-2.6071	1.00E-30	0.605769231	NA	NA	NA
SPARCL1	8404	-2.4676	1.53E-35	0.7586207	-3.2156	4.35E-67	0.817307692	-2.47	1.10E-30	0.68
PPP1R12B	4660	-2.348	3.35E-35	0.7586207	-2.6302	8.80E-63	0.846153846	-2.245	1.62E-25	0.68
CXorf36	79742	-2.471	3.63E-33	0.7586207	-2.7456	2.32E-60	0.817307692	-2.023	1.06E-19	0.52
KIAA1377	57562	-2.7297	7.85E-31	0.7586207	-3.0612	1.58E-54	0.798076923	-2.931	4.98E-29	0.72
CXCL14	9547	-3.0891	1.01E-30	0.7586207	-3.828	6.37E-39	0.788461538	-3.261	1.18E-17	0.72
MYCT1	80177	-2.3949	1.46E-29	0.7586207	-2.8182	7.37E-55	0.798076923	-2.013	2.95E-17	0.52
GNG11	2791	-2.4805	6.19E-29	0.7586207	-2.7311	1.09E-54	0.817307692	NA	NA	NA
NECAB1	64168	-2.6178	1.38E-28	0.7586207	-2.7855	6.94E-41	0.788461538	-2.441	2.41E-22	0.72
ACADSB	36	-2.433	2.84E-28	0.7586207	-2.4131	8.75E-45	0.740384615	-2.503	1.95E-21	0.76
NAP1L2	4674	-2.8492	1.23E-27	0.7586207	-3.4167	9.54E-43	0.778846154	-2.899	3.34E-18	0.56
DMD	1756	-2.6535	1.23E-27	0.7586207	-2.5471	3.34E-39	0.711538462	-2.844	3.31E-26	0.64
TFPI	7035	-2.3047	2.81E-26	0.7586207	-2.5728	3.18E-47	0.721153846	NA	NA	NA
TNNI3K	51086	-2.7692	7.94E-26	0.7586207	-3.1073	2.46E-43	0.759615385	-3.681	1.42E-34	0.92
COL14A1	7373	-3.3012	1.58E-25	0.7586207	-3.5998	9.41E-42	0.788461538	-2.673	2.47E-18	0.72
FAM70A	55026	-2.7612	2.52E-24	0.7586207	-3.4721	7.45E-50	0.865384615	-2.891	1.30E-23	0.76
LAMA2	3908	-2.8008	3.73E-24	0.7586207	-3.4706	2.08E-47	0.836538462	-2.803	1.23E-19	0.72
PTPRB	5787	-2.5059	4.47E-24	0.7586207	-2.9991	1.16E-51	0.855769231	-2.464	3.08E-18	0.68
PER1	5187	-2.503	4.70E-24	0.7586207	-2.4835	7.14E-41	0.778846154	-2.145	6.53E-19	0.72
TGFBR2	7048	-2.2689	8.98E-24	0.7586207	-2.7862	1.47E-58	0.826923077	-2.342	5.67E-23	0.68
LONRF2	164832	-3.2529	9.57E-24	0.7586207	-3.0195	8.60E-26	0.653846154	-3.183	4.34E-17	0.6
IGF2	3481	-2.7933	1.14E-23	0.7586207	-2.9938	1.57E-25	0.759615385	-2.346	1.00E-11	0.64
TBC1D9	23158	-2.4059	1.37E-23	0.7586207	-2.7287	1.44E-48	0.807692308	-2.077	1.84E-17	0.72
SLIT3	6586	-2.7976	1.86E-23	0.7586207	-3.2511	7.66E-43	0.807692308	-2.681	1.76E-18	0.72
LOC10019093[illegible]	100190938	-2.8222	7.85E-23	0.7586207	-2.9116	1.50E-38	0.711538462	-2.463	2.31E-17	0.6
ADHFE1	137872	-2.3055	1.73E-22	0.7586207	-2.8186	8.87E-40	0.740384615	-2.744	1.12E-20	0.8

SLC47A2	146802	-2.3837	6.23E-22	0.7586207	-2.3274	1.90E-29	0.846153846	-2.12	1.07E-16	0.68
PRTG	283659	-2.3989	7.01E-22	0.7586207	-2.6566	4.63E-34	0.701923077	-2.177	9.30E-15	0.64
PGM5P2	595135	-2.1242	1.11E-20	0.7586207	NA	NA	NA	NA	NA	NA
ABCB1	5243	-2.667	1.75E-20	0.7586207	-3.5163	2.15E-44	0.817307692	-3.002	4.34E-19	0.8
TMEM22	80723	-2.2593	2.66E-20	0.7586207	-2.1451	4.22E-35	0.682692308	NA	NA	NA
FGF2	2247	-2.848	2.74E-20	0.7586207	-3.2465	2.67E-40	0.75	-3.423	8.00E-23	0.8
VWF	7450	-2.4514	7.94E-20	0.7586207	-2.531	2.37E-42	0.778846154	-2.177	2.75E-14	0.72
GPM6A	2823	-3.2352	4.03E-19	0.7586207	-3.8615	2.47E-39	0.836538462	-3.876	2.92E-18	0.84
RGS7BP	401190	-3.0704	6.29E-19	0.7586207	-2.8113	9.99E-24	0.740384615	-2.794	1.96E-13	0.72
VIPR2	7434	-2.8107	2.74E-18	0.7586207	-3.0455	1.03E-27	0.711538462	-3.157	2.77E-18	0.72
PLP1	5354	-3.4274	2.29E-17	0.7586207	-3.4153	2.24E-21	0.701923077	-3.687	1.82E-19	0.76
AR	367	-3.4642	1.36E-16	0.7586207	-4.8324	4.56E-39	0.865384615	-3.987	5.47E-17	0.72
PTPRT	11122	-3.7288	6.21E-16	0.7586207	-4.8949	8.06E-39	0.903846154	-3.921	2.68E-14	0.72
VIP	7432	-2.295	3.61E-15	0.7586207	-2.5097	4.47E-38	0.836538462	-2.07	1.23E-11	0.84
RASD1	51655	-2.3986	4.98E-15	0.7586207	-2.3119	8.55E-24	0.634615385	NA	NA	NA
WFIKKN2	124857	-2.0485	5.77E-15	0.7586207	NA	NA	NA	-2.09	1.98E-13	0.8
ARC	23237	-2.965	6.04E-15	0.7586207	-2.7971	3.25E-23	0.721153846	-2.022	5.63E-08	0.68
SLC5A4	6527	-2.2322	1.40E-14	0.7586207	-2.6203	6.60E-36	0.836538462	-2.109	4.96E-10	0.6
SLC7A14	57709	-2.4773	3.51E-14	0.7586207	-2.7486	1.06E-34	0.875	-2.571	3.83E-12	0.84
CACNA2D2	9254	-2.2521	5.37E-14	0.7586207	-2.3288	4.47E-28	0.759615385	NA	NA	NA
CRTAC1	55118	-2.7723	2.18E-12	0.7586207	-2.436	1.21E-15	0.644230769	-3.246	3.01E-16	0.76
CCBE1	147372	-2.2685	3.31E-12	0.7586207	-2.6504	2.74E-32	0.865384615	-2.292	3.18E-10	0.68
HRNR	388697	-2.0837	3.52E-12	0.7586207	-2.3869	1.69E-27	0.759615385	NA	NA	NA
AKR1C2	1646	-3.4696	3.71E-12	0.7586207	-4.389	1.54E-27	0.884615385	-3.478	9.98E-11	0.76
ALDH2	217	-2.1116	9.80E-12	0.7586207	-2.2718	4.29E-30	0.778846154	-2.038	1.63E-09	0.64
C4BPB	725	-2.4646	1.21E-11	0.7586207	NA	NA	NA	NA	NA	NA
LOC148696	148696	-2.1152	2.07E-11	0.7586207	-2.4648	2.04E-32	0.865384615	-2.358	1.39E-11	0.76
KCNA1	3736	-2.519	2.25E-11	0.7586207	-3.1001	1.24E-23	0.807692308	-3.57	1.25E-15	0.88
GPR182	11318	-2.1099	3.06E-11	0.7586207	-2.2685	1.73E-27	0.836538462	NA	NA	NA

NPC1L1	29881	-2.0984	4.33E-11	0.7586207	-2.1271	2.96E-24	0.75	NA	NA	NA
ANKRD43	134548	-2.2306	4.95E-11	0.7586207	-2.4184	3.21E-22	0.740384615	-2.109	2.15E-09	0.6
UBXN10	127733	-2.2057	6.05E-11	0.7586207	-2.6251	3.84E-25	0.711538462	NA	NA	NA
TMC2	117532	-2.2952	7.06E-11	0.7586207	-2.6916	1.27E-34	0.913461538	-2.257	3.73E-10	0.84
NMUR1	10316	-2.5121	1.11E-10	0.7586207	-2.5236	7.00E-24	0.788461538	-2.69	1.42E-10	0.84
IGFN1	91156	-2.253	1.16E-10	0.7586207	-2.5034	1.96E-24	0.798076923	NA	NA	NA
CA12	771	-2.4406	1.84E-10	0.7586207	-2.8684	1.28E-28	0.807692308	-2.26	1.44E-07	0.56
CD209	30835	-2.4784	1.91E-10	0.7586207	-2.0489	1.32E-16	0.692307692	NA	NA	NA
PTCHD3	374308	-2.6127	6.57E-10	0.7586207	-2.64	2.08E-17	0.778846154	-2.785	1.95E-09	0.76
TP53TG3B	729355	-2.0626	8.23E-10	0.7586207	-2.0541	3.69E-18	0.807692308	-2.255	4.52E-10	0.84
FAM5B	57795	-2.46	6.68E-09	0.7586207	NA	NA	NA	NA	NA	NA
CYP4Z1	199974	-4.0412	1.98E-08	0.7586207	-4.8221	8.84E-25	0.913461538	-2.63	0.000344915	0.64
MGC45800	90768	-2.0373	2.84E-08	0.7586207	-2.2096	3.98E-17	0.788461538	-2.06	2.19E-06	0.68
NEUROG2	63973	-2.8414	3.69E-08	0.7586207	-2.6121	1.68E-13	0.817307692	-3.27	1.16E-09	0.88
NR4A1	3164	-2.0271	1.31E-07	0.7586207	-2.0047	7.78E-18	0.788461538	NA	NA	NA
LPPR3	79948	-2.7233	1.12E-06	0.7586207	NA	NA	NA	NA	NA	NA
KLHL30	377007	-2.0646	1.19E-06	0.7586207	-2.1011	6.66E-13	0.75	NA	NA	NA
CYP4Z2P	163720	-3.1628	1.56E-06	0.7586207	-4.2767	1.11E-24	0.884615385	-2.44	0.000195172	0.72
HAP1	9001	-2.3084	5.61E-06	0.7586207	NA	NA	NA	NA	NA	NA
PI15	51050	-2.6286	7.94E-06	0.7586207	-2.4657	1.49E-10	0.769230769	-2.658	2.50E-05	0.8
GP2	2813	-2.95	1.82E-05	0.7586207	-4.1379	1.87E-20	0.903846154	-2.921	9.06E-05	0.84
TCN1	6947	-2.8894	0.000111918	0.7586207	-3.7432	1.67E-14	0.855769231	-2.493	0.001673266	0.68
GPR98	84059	-2.1448	0.000261167	0.7586207	-2.8331	4.29E-14	0.788461538	-2.127	0.000834499	0.76
SCGB3A1	92304	-2.397	0.000536928	0.7586207	-3.5141	1.38E-14	0.817307692	-2.434	0.0013232	0.72
SPRY1	10252	-2.4277	4.03E-36	0.7241379	-2.6361	1.09E-53	0.75	NA	NA	NA
EMCN	51705	-2.5199	1.17E-35	0.7241379	-3.0388	3.08E-57	0.826923077	-2.133	1.76E-20	0.52
CACHD1	57685	-2.6073	3.81E-35	0.7241379	-2.2886	1.14E-43	0.576923077	-2.308	3.82E-28	0.68
RAI2	10742	-2.5799	1.47E-33	0.7241379	-3.3681	3.36E-59	0.865384615	-2.931	4.53E-31	0.76
FAM162B	221303	-2.8862	1.13E-31	0.7241379	-3.2009	5.75E-56	0.875	-2.302	2.21E-18	0.56

PCDHGB7	56099	-2.8086	2.37E-31	0.7241379	-3.2933	1.54E-46	0.75	-3.142	5.33E-32	0.76
RNASE4	6038	-2.4104	2.99E-31	0.7241379	-2.9524	4.56E-63	0.865384615	-2.254	1.17E-21	0.68
NAV3	89795	-2.8402	5.10E-31	0.7241379	-3.4398	1.16E-51	0.836538462	-2.813	2.59E-25	0.68
WSCD1	23302	-2.8903	8.58E-31	0.7241379	-2.7567	9.03E-35	0.730769231	NA	NA	NA
AHNAK	79026	-2.335	5.92E-30	0.7241379	-2.9371	1.16E-60	0.807692308	-2.463	8.91E-28	0.6
HOXA2	3199	-2.975	9.51E-28	0.7241379	-2.6557	9.02E-31	0.644230769	-2.09	4.36E-18	0.6
SHE	126669	-2.4777	2.83E-27	0.7241379	-2.9769	1.45E-52	0.778846154	NA	NA	NA
TAL1	6886	-2.3519	1.78E-25	0.7241379	-2.7471	5.73E-45	0.75	-2.029	1.08E-16	0.56
TMEM88	92162	-2.378	1.96E-25	0.7241379	-2.5762	5.32E-47	0.759615385	NA	NA	NA
PCDHGA2	56113	-2.7768	2.98E-25	0.7241379	-3.3794	1.26E-35	0.778846154	-2.758	4.24E-22	0.72
S1PR1	1901	-2.3197	2.31E-24	0.7241379	-2.7047	2.24E-50	0.788461538	-2.051	1.52E-16	0.64
PHYHD1	254295	-2.7676	3.45E-24	0.7241379	-3.7891	1.57E-41	0.778846154	-2.76	5.63E-19	0.6
DCLK1	9201	-2.6701	4.89E-24	0.7241379	-2.9432	1.13E-39	0.769230769	-2.689	8.25E-21	0.56
DENND2A	27147	-2.4116	6.48E-24	0.7241379	-2.3527	4.40E-30	0.634615385	-2.16	3.64E-17	0.64
ARHGEF15	22899	-2.2807	1.37E-23	0.7241379	-2.5264	4.47E-48	0.788461538	NA	NA	NA
PALMD	54873	-2.6092	1.71E-23	0.7241379	-2.9911	9.57E-47	0.817307692	-2.223	1.35E-16	0.6
DCN	1634	-2.495	3.15E-23	0.7241379	-3.3192	1.98E-37	0.855769231	-2.912	1.41E-20	0.68
C14orf132	56967	-2.4122	6.29E-23	0.7241379	-2.6604	5.73E-32	0.634615385	-2.504	1.55E-21	0.6
C8orf79	57604	-2.7645	1.57E-22	0.7241379	-3.6807	6.28E-46	0.884615385	-3.36	8.05E-20	0.64
LTBP4	8425	-2.1144	1.95E-22	0.7241379	-2.33	1.11E-41	0.692307692	-2.065	4.50E-20	0.6
TEK	7010	-2.3469	2.55E-22	0.7241379	-2.8678	5.95E-45	0.798076923	-2.211	2.94E-15	0.64
ADRA2A	150	-2.8085	4.13E-22	0.7241379	-3.4249	8.26E-45	0.826923077	-3.307	1.31E-19	0.72
TMEM232	642987	-2.4807	1.22E-21	0.7241379	-2.7174	1.46E-40	0.769230769	-2.526	2.45E-21	0.72
FLJ42875	440556	-2.653	1.74E-21	0.7241379	-2.8004	1.39E-37	0.817307692	-2.314	3.82E-16	0.72
RYR3	6263	-2.5822	5.26E-21	0.7241379	-2.6915	3.44E-32	0.759615385	-2.644	3.61E-17	0.72
TACR1	6869	-2.8499	1.01E-20	0.7241379	-3.1165	2.59E-30	0.711538462	-3.121	2.43E-18	0.68
PALM	5064	-2.8538	1.06E-20	0.7241379	-2.9673	5.09E-35	0.759615385	-2.592	3.39E-15	0.68
VIT	5212	-3.3643	1.48E-20	0.7241379	-4.1118	7.44E-34	0.769230769	-4.027	1.26E-25	0.8
ADAMTS1	9510	-2.1461	1.81E-20	0.7241379	-2.0766	1.94E-33	0.663461538	NA	NA	NA

SOX17	64321	-2.4383	4.55E-20	0.7241379	-2.7062	3.17E-41	0.836538462	NA	NA	NA
LOC283174	283174	-3.0932	3.49E-19	0.7241379	-3.637	1.30E-36	0.788461538	-3.46	2.82E-21	0.68
CLDN11	5010	-2.6173	4.41E-19	0.7241379	-3.8094	9.30E-53	0.903846154	-3.413	2.09E-25	0.8
RBP7	116362	-2.5025	5.98E-19	0.7241379	-2.4042	7.53E-30	0.634615385	-2.219	1.05E-14	0.64
GPR44	11251	-2.0461	6.87E-19	0.7241379	-2.6288	7.50E-57	0.836538462	-2.11	5.06E-20	0.76
ANK2	287	-2.5277	1.24E-18	0.7241379	-2.9105	3.50E-43	0.846153846	-2.767	1.07E-16	0.8
CYP4F12	66002	-3.2689	1.61E-18	0.7241379	-3.9091	7.79E-38	0.817307692	-2.625	3.29E-14	0.72
SIK2	23235	-2.0281	1.23E-17	0.7241379	-2.0375	4.76E-39	0.759615385	-2.095	5.74E-16	0.68
ASAM	79827	-2.4407	3.81E-17	0.7241379	-2.6628	1.73E-30	0.788461538	-2.246	7.20E-14	0.76
PKNOX2	63876	-2.4068	8.86E-17	0.7241379	-2.3114	9.76E-22	0.634615385	NA	NA	NA
ST6GALNAC1	55808	-2.7015	1.33E-16	0.7241379	-3.4872	5.05E-34	0.788461538	-2.144	1.56E-09	0.64
KLHL13	90293	-2.8274	1.59E-16	0.7241379	-2.7858	2.29E-28	0.769230769	-2.831	2.13E-12	0.64
NGFR	4804	-2.8653	2.78E-16	0.7241379	-3.0222	9.43E-27	0.711538462	-2.507	3.15E-11	0.6
PPP1R3C	5507	-2.2507	3.57E-15	0.7241379	-3.0031	4.28E-35	0.846153846	-2.494	3.02E-16	0.72
KCNMA1	3778	-2.1174	6.78E-15	0.7241379	-2.5349	1.42E-29	0.730769231	-2.006	2.08E-11	0.6
FAM189A2	9413	-2.4046	8.59E-15	0.7241379	-2.8212	3.27E-27	0.701923077	-2.888	7.65E-15	0.72
CSRNP1	64651	-2.0341	1.15E-14	0.7241379	-2.2144	1.44E-37	0.798076923	NA	NA	NA
KCNK3	3777	-2.5246	1.51E-14	0.7241379	NA	NA	NA	NA	NA	NA
C10orf108	414235	-2.2615	2.87E-14	0.7241379	-2.2912	8.91E-21	0.644230769	-2.352	1.40E-15	0.76
TF	7018	-2.9902	2.90E-14	0.7241379	-2.8663	1.04E-17	0.701923077	-3.449	8.60E-16	0.72
IL6ST	3572	-2.679	3.69E-14	0.7241379	-3.5128	1.30E-30	0.769230769	-3.334	4.10E-17	0.72
LOC339524	339524	-2.2585	5.00E-14	0.7241379	-2.4071	3.28E-30	0.817307692	-2.149	1.09E-11	0.72
KCNB1	3745	-2.8577	8.68E-14	0.7241379	-3.7586	1.89E-35	0.846153846	-3.165	1.46E-14	0.76
CALN1	83698	-2.4094	3.80E-13	0.7241379	-2.9997	1.13E-25	0.865384615	-3.546	4.84E-25	0.92
SLC26A4	5172	-2.1459	7.08E-13	0.7241379	-2.1177	4.83E-21	0.653846154	-2.406	2.64E-13	0.68
CACNA2D1	781	-3.0047	8.75E-13	0.7241379	-3.6186	2.33E-27	0.807692308	-4.286	1.57E-19	0.96
CBLN1	869	-2.5955	1.75E-12	0.7241379	-2.8598	3.27E-26	0.759615385	-2.005	2.77E-07	0.6
PAR5	8123	-2.6391	2.22E-12	0.7241379	-3.2735	5.71E-29	0.826923077	-2.77	1.59E-11	0.76
GPR77	27202	-2.0596	2.93E-12	0.7241379	NA	NA	NA	NA	NA	NA

C10orf93	255352	-2.1134	4.78E-12	0.7241379	-2.0781	6.53E-20	0.740384615	-2.107	2.95E-11	0.72
GREM2	64388	-2.3168	7.35E-12	0.7241379	-4.1227	2.38E-46	0.894230769	-3.387	1.08E-17	0.8
ANKK1	255239	-2.3788	1.24E-11	0.7241379	-2.1236	4.24E-21	0.673076923	NA	NA	NA
PCDHA10	56139	-2.3762	5.45E-11	0.7241379	-2.379	8.93E-15	0.653846154	-2.326	1.67E-07	0.64
IL20RA	53832	-2.6235	6.50E-11	0.7241379	-3.1013	4.82E-27	0.788461538	-2.674	9.07E-09	0.64
NCRNA00093	100188954	-2.1177	1.76E-10	0.7241379	-2.6365	2.84E-29	0.807692308	-2.087	8.36E-09	0.6
FLRT3	23767	-2.474	3.11E-10	0.7241379	-2.9952	5.83E-22	0.701923077	NA	NA	NA
SCN2A	6326	-2.4291	6.03E-10	0.7241379	-2.5157	1.80E-15	0.692307692	-2.941	7.50E-11	0.72
NPY1R	4886	-2.6139	1.92E-09	0.7241379	-3.1855	3.07E-22	0.740384615	-3.229	3.90E-10	0.6
KIT	3815	-2.3969	3.71E-09	0.7241379	-2.7391	1.02E-21	0.75	-2.706	4.20E-09	0.76
CACNA1G	8913	-2.2258	5.85E-09	0.7241379	-2.6922	6.10E-22	0.826923077	-2.195	7.83E-08	0.88
NAT8L	339983	-2.2942	2.12E-08	0.7241379	-2.7087	2.51E-22	0.778846154	-2.771	6.66E-10	0.8
PLD5	200150	-2.0892	4.04E-08	0.7241379	-2.8871	2.18E-25	0.759615385	-2.771	5.24E-13	0.76
ACTG2	72	-2.6101	5.47E-08	0.7241379	-2.3896	5.46E-11	0.615384615	-3.035	1.29E-08	0.6
CAPN8	388743	-3.0346	8.13E-07	0.7241379	-4.3713	6.51E-27	0.913461538	-2.752	1.79E-05	0.72
TMEM84	283673	-2.1546	2.32E-06	0.7241379	-2.5247	1.71E-16	0.75	NA	NA	NA
C1orf168	199920	-2.3065	3.36E-06	0.7241379	-2.9218	9.11E-20	0.855769231	-2.727	6.65E-07	0.76
HS3ST4	9951	-2.6066	4.53E-06	0.7241379	-3.6925	9.55E-21	0.807692308	-3.638	6.45E-09	0.8
C20orf114	92747	-2.6392	2.66E-05	0.7241379	-2.6836	7.91E-10	0.759615385	NA	NA	NA
SLC44A4	80736	-2.0273	0.000124836	0.7241379	-2.7908	3.20E-13	0.692307692	NA	NA	NA
C4BPA	722	-2.195	0.000288559	0.7241379	-3.1387	1.86E-14	0.788461538	-2.877	4.31E-06	0.8
SLCO1A2	6579	-2.122	0.000843078	0.7241379	-2.5232	3.06E-10	0.807692308	-2.745	2.67E-05	0.8
FOXA1	3169	-2.1416	0.001560165	0.7241379	-2.8032	3.03E-11	0.759615385	NA	NA	NA
KRT14	3861	-2.0697	0.004378714	0.7241379	-2.6248	7.10E-08	0.75	-2.34	0.006155861	0.6
PARK2	5071	-2.3347	3.88E-40	0.6896552	-2.8609	2.15E-59	0.778846154	-2.42	2.57E-29	0.64
FLRT2	23768	-2.4778	7.85E-31	0.6896552	-2.8757	1.02E-38	0.721153846	-2.083	7.20E-15	0.56
TXNIP	10628	-2.2651	4.25E-29	0.6896552	-2.7222	5.82E-60	0.817307692	-2.249	4.25E-24	0.68
ITM2A	9452	-2.6123	4.61E-29	0.6896552	-3.2817	1.52E-47	0.807692308	-2.873	3.20E-28	0.76
EFEMP1	2202	-2.7908	5.98E-29	0.6896552	-2.8421	5.33E-35	0.730769231	-2.328	1.25E-19	0.6

CYBRD1	79901	-2.3265	4.35E-27	0.6896552	-3.148	1.14E-67	0.875	-2.605	4.36E-28	0.76
SOCS2	8835	-2.4001	1.25E-25	0.6896552	-2.7925	3.54E-49	0.769230769	-2.073	9.13E-20	0.64
DBX2	440097	-2.9933	2.35E-25	0.6896552	-3.4377	3.58E-50	0.865384615	-3.224	2.32E-23	0.84
FGF1	2246	-2.4513	5.80E-24	0.6896552	-3.2335	3.67E-54	0.846153846	-2.804	1.06E-24	0.76
GPR116	221395	-2.2184	2.40E-22	0.6896552	-2.3746	2.98E-43	0.740384615	NA	NA	NA
GNAL	2774	-2.3206	7.20E-22	0.6896552	-2.9063	1.47E-37	0.721153846	-2.652	9.71E-20	0.68
MEG3	55384	-2.5091	8.43E-22	0.6896552	-3.0437	2.72E-32	0.769230769	-2.482	1.44E-15	0.68
GCNT4	51301	-2.5866	1.18E-21	0.6896552	-3.2157	6.69E-44	0.836538462	-2.575	5.27E-19	0.64
FMOD	2331	-2.2487	2.82E-21	0.6896552	-2.6232	1.77E-35	0.740384615	-2.503	1.27E-21	0.8
TLL1	7092	-2.5322	1.08E-20	0.6896552	-2.9929	2.82E-37	0.75	-2.002	1.46E-09	0.48
ASXL3	80816	-2.6583	3.15E-20	0.6896552	-3.0031	2.90E-31	0.759615385	-3.155	5.54E-19	0.76
RIMBP2	23504	-2.5084	2.33E-19	0.6896552	-3.076	1.92E-38	0.826923077	-2.967	3.02E-24	0.72
BTG2	7832	-2.0117	5.46E-19	0.6896552	-2.3135	3.29E-41	0.711538462	NA	NA	NA
PID1	55022	-2.3854	1.41E-18	0.6896552	-2.4547	6.67E-34	0.75	-2.502	1.20E-17	0.72
PLA2G16	11145	-2.2796	3.14E-17	0.6896552	-2.5713	2.90E-31	0.730769231	-2.388	1.79E-15	0.72
SLC22A3	6581	-2.6147	1.95E-16	0.6896552	-3.1847	3.42E-35	0.817307692	-2.861	1.15E-15	0.64
ODZ1	10178	-2.152	2.24E-15	0.6896552	-2.8241	9.90E-26	0.721153846	-2.263	3.41E-10	0.6
SEMA5A	9037	-2.2159	5.04E-15	0.6896552	-2.8862	3.12E-35	0.740384615	-2.476	5.38E-14	0.76
LIN7A	8825	-2.3559	1.23E-14	0.6896552	-3.3826	3.77E-41	0.846153846	-2.876	2.44E-18	0.76
SERPINA5	5104	-3.1332	1.32E-14	0.6896552	-3.6958	1.96E-28	0.769230769	-3.408	7.06E-18	0.68
KCNA6	3742	-2.1072	1.74E-12	0.6896552	-2.6537	7.44E-23	0.711538462	-3.314	3.88E-21	0.76
PACRG	135138	-2.127	2.17E-12	0.6896552	-2.3297	2.46E-24	0.711538462	-2.495	3.73E-13	0.56
RBM24	221662	-2.3076	3.47E-12	0.6896552	-2.3913	1.95E-21	0.778846154	-2.603	5.16E-14	0.84
APOD	347	-2.7226	3.88E-12	0.6896552	-4.6362	8.10E-37	0.875	-3.635	1.49E-15	0.64
SORCS1	114815	-2.6235	4.24E-11	0.6896552	-3.2743	1.39E-20	0.759615385	-3.689	3.28E-18	0.84
C14orf49	161176	-2.1422	4.63E-11	0.6896552	-2.4916	5.44E-29	0.846153846	-2.2	7.05E-11	0.76
D4S234E	27065	-2.4654	1.07E-10	0.6896552	-3.0372	5.72E-22	0.75	-2.905	4.29E-12	0.68
FMO5	2330	-2.0645	1.30E-10	0.6896552	-3.1989	8.54E-39	0.875	-2.202	7.38E-10	0.72
RANBP3L	202151	-2.549	2.53E-10	0.6896552	-3.6303	2.30E-31	0.798076923	-3.38	1.48E-13	0.8

IGSF9B	22997	-2.2443	4.89E-10	0.6896552	-2.7702	3.92E-24	0.75	-3.119	2.10E-14	0.68
CSRNP3	80034	-2.5054	6.48E-10	0.6896552	-2.6277	3.70E-20	0.788461538	-3.099	1.10E-12	0.88
CEBPA	1050	-2.0506	6.76E-09	0.6896552	-2.0364	1.19E-20	0.740384615	NA	NA	NA
ELOVL2	54898	-2.0442	1.12E-08	0.6896552	-2.4353	2.60E-18	0.721153846	NA	NA	NA
LOC10018894	100188947	-2.1696	4.45E-08	0.6896552	-2.8192	2.56E-27	0.836538462	-2.04	2.57E-06	0.76
CLCA2	9635	-2.5832	5.94E-06	0.6896552	-3.8299	3.05E-17	0.798076923	NA	NA	NA
AQP1	358	-2.6043	5.93E-36	0.6551724	-2.9136	5.89E-48	0.721153846	-2.312	4.03E-24	0.68
THRB	7068	-2.3971	3.19E-33	0.6551724	-3.2029	4.35E-57	0.836538462	-2.642	1.88E-27	0.6
C4orf32	132720	-2.3905	7.10E-32	0.6551724	-2.5441	1.68E-49	0.740384615	NA	NA	NA
SEPP1	6414	-2.6985	1.52E-31	0.6551724	-3.1789	2.33E-56	0.788461538	-2.532	1.29E-22	0.56
C21orf34	388815	-2.6309	3.78E-31	0.6551724	-3.2879	3.69E-56	0.807692308	-2.639	1.48E-23	0.68
EDNRB	1910	-2.7732	4.63E-30	0.6551724	-3.0423	1.94E-45	0.798076923	-2.706	1.57E-22	0.72
ARHGAP6	395	-2.253	6.49E-30	0.6551724	-2.5464	3.63E-48	0.721153846	NA	NA	NA
SEMA3C	10512	-2.9017	2.01E-28	0.6551724	-3.108	2.67E-44	0.759615385	-2.131	3.27E-18	0.56
TENC1	23371	-2.0769	1.82E-27	0.6551724	-2.6313	1.25E-66	0.817307692	-2.317	3.43E-25	0.72
DST	667	-2.8554	2.16E-27	0.6551724	-2.9804	4.41E-55	0.884615385	-2.671	6.88E-22	0.68
SH3D19	152503	-2.2841	4.88E-27	0.6551724	-2.5981	8.46E-55	0.798076923	-2.465	7.77E-26	0.76
PNPLA7	375775	-2.245	8.63E-27	0.6551724	-2.8771	3.53E-44	0.759615385	-2.206	1.69E-24	0.6
FAM13A	10144	-2.1503	1.31E-24	0.6551724	-2.2964	3.80E-60	0.826923077	-2.285	3.91E-24	0.64
MRGPRF	116535	-2.4087	1.92E-24	0.6551724	-2.8671	5.94E-47	0.778846154	-2.296	5.81E-20	0.72
RUNX1T1	862	-2.6911	9.23E-24	0.6551724	-3.6288	1.80E-45	0.855769231	-3.171	2.60E-23	0.8
SLC40A1	30061	-2.2628	9.57E-24	0.6551724	-2.8686	1.17E-41	0.740384615	-2.054	1.46E-14	0.48
CPEB1	64506	-2.4604	1.86E-23	0.6551724	-3.1707	1.40E-40	0.778846154	-2.956	1.28E-22	0.72
CCDC48	79825	-2.1677	4.16E-23	0.6551724	-2.6066	7.86E-37	0.730769231	NA	NA	NA
LGI4	163175	-2.3728	3.55E-22	0.6551724	-2.7364	2.43E-42	0.778846154	-2.319	1.65E-18	0.72
RSPO3	84870	-2.2311	7.75E-20	0.6551724	-2.755	6.96E-36	0.769230769	-2.362	5.70E-15	0.68
GRRP1	79927	-2.1661	2.05E-19	0.6551724	-2.6637	4.59E-46	0.826923077	NA	NA	NA
PCDHGA3	56112	-2.3114	2.78E-19	0.6551724	-2.9852	1.98E-42	0.817307692	-2.367	8.41E-19	0.68
CDH23	64072	-2.4747	5.45E-19	0.6551724	-2.8044	1.17E-34	0.778846154	-2.174	1.15E-13	0.68

KCND3	3752	-3.1009	6.57E-17	0.6551724	-4.0067	2.62E-45	0.865384615	-2.929	1.06E-13	0.72
KLF2	10365	-2.0061	1.65E-16	0.6551724	-2.2806	2.13E-37	0.721153846	NA	NA	NA
STEAP4	79689	-2.3713	3.63E-16	0.6551724	-3.4789	2.11E-43	0.817307692	-2.307	2.02E-16	0.72
AFF3	3899	-2.6859	1.14E-15	0.6551724	-3.8972	5.35E-39	0.817307692	-3.632	5.47E-18	0.76
C7	730	-3.6137	1.37E-14	0.6551724	-4.8417	7.93E-31	0.826923077	-3.163	1.86E-11	0.76
DNAH6	1768	-2.3277	5.59E-14	0.6551724	-2.4748	1.07E-30	0.701923077	-3.09	1.63E-18	0.72
PRRG3	79057	-2.2578	1.00E-13	0.6551724	-2.7461	2.44E-29	0.75	-3.21	1.84E-23	0.84
ZNF385B	151126	-2.4001	1.88E-13	0.6551724	-2.6725	4.71E-27	0.759615385	-2.433	1.47E-09	0.68
SIGLECP3	284367	-2.1594	2.25E-13	0.6551724	-2.6915	1.86E-34	0.826923077	-2.268	2.81E-12	0.68
FCER1A	2205	-2.143	6.17E-13	0.6551724	-3.2044	1.44E-29	0.730769231	-2.08	2.90E-11	0.56
WDR86	349136	-2.2714	2.45E-12	0.6551724	-2.4569	5.92E-25	0.730769231	NA	NA	NA
MAPK10	5602	-2.4176	2.13E-11	0.6551724	-2.989	1.54E-24	0.730769231	-2.261	1.17E-09	0.68
MS4A2	2206	-2.4875	5.74E-11	0.6551724	-3.2257	1.59E-30	0.778846154	-2.136	8.86E-08	0.72
SNORD116-4	100033416	-2.6944	2.17E-10	0.6551724	-3.281	1.44E-23	0.740384615	-3.06	9.50E-11	0.64
C1orf150	148823	-2.2045	5.94E-10	0.6551724	-2.588	2.87E-29	0.846153846	-2.15	2.45E-08	0.72
DDR2	4921	-2.2047	7.49E-10	0.6551724	-2.5005	1.32E-21	0.778846154	-2.642	2.52E-12	0.84
PDE1C	5137	-2.6389	9.82E-10	0.6551724	-2.9007	5.83E-23	0.721153846	-3.269	2.17E-13	0.72
VSIG2	23584	-2.9812	1.44E-09	0.6551724	-3.896	6.62E-27	0.788461538	-2.648	1.02E-06	0.68
CADM3	57863	-2.3395	1.95E-09	0.6551724	-3.8479	2.76E-32	0.807692308	-3.141	1.24E-16	0.72
MLPH	79083	-2.2286	3.52E-09	0.6551724	-3.1079	4.66E-25	0.817307692	NA	NA	NA
LOC643763	643763	-2.0559	3.55E-09	0.6551724	-2.6819	1.11E-24	0.817307692	-2.626	3.34E-14	0.76
TMC5	79838	-2.6418	2.73E-07	0.6551724	-3.3662	1.10E-17	0.759615385	-2.144	0.000129701	0.56
CRB2	286204	-2.0074	1.02E-06	0.6551724	-2.5719	5.01E-22	0.807692308	-2.14	1.69E-06	0.64
TFAP2B	7021	-2.2077	0.003074672	0.6551724	-3.8231	1.02E-14	0.836538462	NA	NA	NA
LRP2	4036	-2.26	0.004783356	0.6551724	-3.0387	8.99E-09	0.701923077	-3.855	1.15E-05	0.8
CXCL12	6387	-2.3335	1.02E-28	0.6206897	-2.862	1.44E-52	0.788461538	-2.625	3.90E-25	0.68
NDN	4692	-2.2374	1.43E-28	0.6206897	-2.7541	1.74E-44	0.75	-2.139	1.14E-19	0.6
RUNDC3B	154661	-2.403	7.24E-27	0.6206897	-2.9816	1.63E-50	0.788461538	-2.594	6.63E-23	0.76
PCDHGB6	56100	-2.5481	1.44E-26	0.6206897	-2.6837	6.67E-38	0.730769231	-2.834	3.26E-28	0.84

RGS5	8490	-2.1938	2.58E-26	0.6206897	-2.5362	7.20E-40	0.75	NA	NA	NA
NR5A2	2494	-2.1066	2.67E-25	0.6206897	-2.4026	4.76E-45	0.701923077	NA	NA	NA
C7orf63	79846	-2.237	3.73E-25	0.6206897	-2.7408	1.46E-53	0.769230769	-2.722	1.15E-25	0.72
PLSCR4	57088	-2.1391	1.40E-24	0.6206897	-2.9219	3.21E-60	0.846153846	-2.89	2.42E-28	0.8
EPAS1	2034	-2.134	6.67E-23	0.6206897	-2.3493	2.50E-48	0.721153846	NA	NA	NA
MAST4	375449	-2.4305	8.54E-23	0.6206897	-2.8272	1.39E-44	0.701923077	-2.169	3.01E-20	0.6
ATP1B2	482	-2.0404	3.89E-22	0.6206897	-2.561	1.52E-35	0.701923077	-2.335	2.46E-17	0.64
ROBO4	54538	-2.0006	1.11E-21	0.6206897	-2.2613	4.62E-47	0.701923077	NA	NA	NA
PLEKHH2	130271	-2.1661	1.94E-21	0.6206897	-2.9926	5.32E-43	0.75	-2.834	9.67E-24	0.68
DLG2	1740	-2.0918	6.14E-21	0.6206897	-2.6154	8.18E-34	0.701923077	-2.059	3.42E-13	0.52
TTC18	118491	-2.1753	3.74E-19	0.6206897	-2.5371	1.49E-39	0.711538462	-2.54	3.12E-19	0.64
PRDM16	63976	-2.1531	3.78E-18	0.6206897	-2.6617	1.04E-32	0.75	-2.306	3.57E-17	0.6
F3	2152	-2.1939	1.06E-17	0.6206897	-2.6969	2.82E-45	0.711538462	NA	NA	NA
ACER2	340485	-2.2019	1.38E-17	0.6206897	-2.7678	2.38E-44	0.769230769	-2.306	2.68E-17	0.6
KIAA1683	80726	-2.3242	2.23E-17	0.6206897	-2.9566	1.36E-39	0.721153846	-2.502	3.24E-17	0.68
C9orf68	55064	-2.204	5.23E-17	0.6206897	-2.5984	5.57E-33	0.75	-2.244	2.02E-14	0.64
MYLK	4638	-2.2046	1.01E-16	0.6206897	-2.541	2.45E-32	0.701923077	-2.327	1.50E-16	0.68
SCN11A	11280	-2.1881	2.36E-16	0.6206897	-2.784	9.15E-43	0.865384615	-2.354	4.08E-18	0.64
ABI3BP	25890	-2.7258	3.24E-16	0.6206897	-3.5395	3.98E-35	0.826923077	-2.785	3.07E-14	0.72
GPR34	2857	-2.1165	1.54E-15	0.6206897	-2.5317	5.25E-39	0.759615385	NA	NA	NA
OMD	4958	-2.2957	3.41E-15	0.6206897	-3.3895	7.42E-38	0.817307692	-3.165	1.64E-17	0.8
SNORD116-20	100033431	-2.0779	3.46E-14	0.6206897	-2.5582	2.27E-33	0.75	-2.13	1.20E-13	0.52
NCAM2	4685	-2.0909	4.76E-13	0.6206897	-2.6498	1.06E-28	0.730769231	-2.53	1.17E-11	0.64
HDC	3067	-2.2395	1.90E-10	0.6206897	-2.8799	3.02E-28	0.788461538	-2.035	1.15E-08	0.68
NPY5R	4889	-2.6245	1.44E-09	0.6206897	-3.1402	1.45E-22	0.721153846	-3.004	5.34E-10	0.6
THBS4	7060	-2.3151	2.26E-09	0.6206897	-2.6002	1.94E-16	0.730769231	NA	NA	NA
LMO3	55885	-2.1858	4.83E-08	0.6206897	-2.1667	7.95E-13	0.701923077	-2.618	2.96E-09	0.76
WNK4	65266	-2.8082	3.78E-06	0.6206897	-3.0287	1.27E-14	0.788461538	-2.419	0.000113672	0.64
WIF1	11197	-2.6487	0.000421613	0.6206897	-3.6589	1.15E-13	0.817307692	-3.898	2.15E-07	0.88

SPRY2	10253	-2.118	8.50E-35	0.5862069	-2.4388	4.05E-53	0.701923077	-2.191	3.84E-28	0.64
C16orf45	89927	-2.1123	2.93E-30	0.5862069	-2.6078	2.23E-55	0.75	NA	NA	NA
BCL2	596	-2.3262	1.08E-29	0.5862069	-2.6409	1.18E-48	0.75	-2.598	5.79E-29	0.68
NR3C2	4306	-2.4232	3.19E-29	0.5862069	-2.938	2.21E-56	0.769230769	-2.67	2.56E-24	0.68
KLHL29	114818	-2.384	1.09E-27	0.5862069	-2.6603	1.48E-45	0.730769231	-2.487	1.96E-26	0.68
C4orf12	404201	-2.1682	5.08E-27	0.5862069	-2.8951	5.55E-56	0.817307692	-2.472	5.65E-29	0.68
NXPH3	11248	-2.2969	5.24E-26	0.5862069	-2.6876	2.87E-40	0.711538462	-2.468	8.28E-27	0.72
FLJ36777	730971	-2.0861	8.96E-26	0.5862069	-2.3202	1.49E-44	0.711538462	-2.064	3.36E-19	0.6
ECSCR	641700	-2.1224	2.10E-25	0.5862069	-2.5007	2.12E-47	0.759615385	NA	NA	NA
EFHA2	286097	-2.3844	3.32E-25	0.5862069	-2.9311	6.08E-43	0.759615385	-2.874	4.99E-27	0.84
PRICKLE2	166336	-2.1183	5.25E-25	0.5862069	-2.7535	6.42E-51	0.759615385	-2.11	3.37E-23	0.64
GALNTL1	57452	-2.4231	1.56E-21	0.5862069	-3.1297	5.39E-37	0.701923077	-2.232	5.33E-18	0.6
FAT4	79633	-2.3048	3.31E-21	0.5862069	-2.8214	1.58E-35	0.730769231	-2.591	1.88E-21	0.72
ANGPTL2	23452	-2.0335	3.40E-18	0.5862069	-2.4072	2.66E-41	0.721153846	-2.082	1.72E-17	0.68
SELP	6403	-2.7252	4.74E-16	0.5862069	-3.7728	5.06E-34	0.778846154	-2.472	2.65E-12	0.52
CYP21A2	1589	-2.2343	2.56E-15	0.5862069	-2.4406	8.44E-23	0.701923077	NA	NA	NA
SEMA3D	223117	-2.485	4.83E-15	0.5862069	-3.4671	8.63E-35	0.759615385	-3.413	1.64E-23	0.72
DSCAML1	57453	-2.2274	2.50E-14	0.5862069	-2.4447	7.50E-29	0.75	-2.609	5.45E-17	0.76
INPP4B	8821	-2.1708	8.43E-14	0.5862069	-2.8719	2.58E-34	0.769230769	-2.068	2.87E-10	0.56
ZNF366	167465	-2.0759	2.34E-13	0.5862069	-2.6974	8.86E-34	0.711538462	-2.473	1.53E-15	0.64
GPR17	2840	-2.2653	1.17E-12	0.5862069	-2.7892	6.75E-30	0.778846154	-2.666	6.21E-16	0.76
FOXP2	93986	-2.2447	1.58E-11	0.5862069	-2.9883	1.93E-26	0.778846154	-3.276	3.80E-17	0.84
SHISA6	388336	-2.1559	1.92E-11	0.5862069	-2.8963	2.14E-33	0.798076923	-2.603	5.68E-13	0.72
ST8SIA6	338596	-2.0626	1.34E-06	0.5862069	-2.73	5.67E-20	0.721153846	-3.156	9.27E-13	0.72
CCL28	56477	-2.1991	7.12E-06	0.5862069	-3.3703	8.05E-21	0.807692308	-3.094	9.55E-09	0.76
THSD1	55901	-2.1393	1.10E-40	0.5517241	-2.4503	5.89E-63	0.701923077	NA	NA	NA
CBX7	23492	-2.0195	5.09E-35	0.5517241	-2.5624	2.86E-65	0.759615385	-2.295	6.70E-32	0.72
KLHDC1	122773	-2.257	6.98E-34	0.5517241	-2.7782	3.30E-67	0.75	-2.25	2.92E-26	0.56
PELI2	57161	-2.1885	1.12E-29	0.5517241	-2.5931	2.98E-38	0.740384615	-2.282	8.53E-24	0.6

ADCY4	196883	-2.2154	2.23E-27	0.5517241	-2.5462	4.96E-45	0.701923077	NA	NA	NA
ZCCHC24	219654	-2.2369	2.23E-26	0.5517241	-2.6627	1.39E-53	0.778846154	-2.019	1.45E-19	0.64
LRRC17	10234	-2.0087	2.31E-14	0.5517241	-2.7868	4.39E-33	0.75	NA	NA	NA
DYNLRB2	83657	-2.1913	3.16E-12	0.5517241	-2.8806	1.26E-31	0.798076923	-2.326	1.22E-10	0.6
CPA3	1359	-2.4981	6.64E-09	0.5517241	-3.1295	2.84E-23	0.730769231	-2.125	3.26E-06	0.56
CCL21	6366	-2.4263	3.31E-08	0.5517241	-4.4958	4.20E-28	0.817307692	-3.163	4.17E-11	0.64
MSRB3	253827	-2.1388	9.03E-31	0.5172414	-2.8266	2.88E-55	0.769230769	-2.47	6.30E-31	0.8
CCDC152	100129792	-2.0151	1.45E-24	0.5172414	-2.6038	3.63E-48	0.711538462	-2.018	1.27E-19	0.52
PDE1A	5136	-2.0683	1.24E-22	0.5172414	-2.6075	1.44E-41	0.721153846	NA	NA	NA
PLA2R1	22925	-2.068	1.44E-18	0.4827586	-2.4154	3.89E-36	0.701923077	-2.189	4.26E-21	0.6
NR2F1	7025	-2.1008	5.55E-17	0.4827586	-2.9107	2.59E-33	0.701923077	NA	NA	NA
ISM1	140862	-2.1647	1.22E-14	0.4827586	-3.347	9.80E-42	0.769230769	-2.851	7.55E-20	0.72
VIPR1	7433	-2.2079	1.74E-20	0.4482759	-2.7169	1.38E-39	0.721153846	NA	NA	NA
LDB3	11155	NA	NA	NA	-2.3242	3.41E-29	1	-2.076	1.66E-09	1
MYL3	4634	NA	NA	NA	-2.3152	3.02E-21	1	-2.069	4.83E-07	1
SULT1C3	442038	NA	NA	NA	-2.0031	6.02E-07	0.990384615	NA	NA	NA
NLRP13	126204	NA	NA	NA	-2.0354	1.59E-21	0.971153846	NA	NA	NA
MYOM3	127294	NA	NA	NA	-2.2698	2.73E-21	0.961538462	NA	NA	NA
NLRP8	126205	NA	NA	NA	-2.0535	2.68E-24	0.951923077	NA	NA	NA
HSD3B2	3284	NA	NA	NA	-2.491	9.17E-11	0.951923077	NA	NA	NA
GLP2R	9340	NA	NA	NA	-2.5571	6.10E-20	0.942307692	-2.607	1.71E-07	1
CHRNA2	1135	NA	NA	NA	-2.111	3.85E-13	0.942307692	NA	NA	NA
ACSM1	116285	NA	NA	NA	-2.4223	7.25E-10	0.942307692	NA	NA	NA
MYBPC1	4604	NA	NA	NA	-3.1243	9.66E-09	0.932692308	-3.566	0.000112029	0.92
CLEC3A	10143	NA	NA	NA	-2.3205	5.49E-08	0.932692308	NA	NA	NA
SERHL2	253190	NA	NA	NA	-2.2768	8.07E-12	0.923076923	NA	NA	NA
MUC7	4589	NA	NA	NA	-2.1356	2.72E-07	0.923076923	NA	NA	NA
PRKAR2B	5577	NA	NA	NA	-2.5932	2.74E-28	0.913461538	-2.614	1.56E-10	0.88
LRRC31	79782	NA	NA	NA	-2.6341	2.38E-15	0.913461538	NA	NA	NA

ALOX15B	247	NA	NA	NA	-2.0918	1.11E-06	0.913461538	NA	NA	NA
SMR3B	10879	NA	NA	NA	-2.4325	1.06E-07	0.903846154	NA	NA	NA
UNC13C	440279	NA	NA	NA	-2.4791	3.01E-32	0.894230769	-2.394	1.65E-13	0.8
APOF	319	NA	NA	NA	-2.1184	1.50E-21	0.894230769	NA	NA	NA
AMBP	259	NA	NA	NA	-2.1182	7.59E-15	0.894230769	NA	NA	NA
CYP4F8	11283	NA	NA	NA	-2.8982	1.49E-11	0.894230769	NA	NA	NA
DHRS2	10202	NA	NA	NA	-2.7482	9.29E-09	0.884615385	NA	NA	NA
DCD	117159	NA	NA	NA	-2.148	1.51E-07	0.875	NA	NA	NA
HPGD	3248	NA	NA	NA	-2.5622	4.15E-16	0.865384615	-2.032	4.54E-05	0.8
SPINK8	646424	NA	NA	NA	-2.2624	1.67E-09	0.865384615	NA	NA	NA
KCTD4	386618	NA	NA	NA	-2.0948	8.81E-26	0.855769231	-2.455	2.87E-14	0.8
SCGB2A1	4246	NA	NA	NA	-3.1227	9.79E-13	0.855769231	NA	NA	NA
ACSL1	2180	NA	NA	NA	-2.2431	1.05E-25	0.846153846	NA	NA	NA
KIAA0087	9808	NA	NA	NA	-2.4885	1.61E-44	0.836538462	-2.397	1.87E-16	0.8
IQUB	154865	NA	NA	NA	-2.514	6.83E-34	0.836538462	-2.425	6.21E-13	0.76
ITIH3	3699	NA	NA	NA	-2.6267	2.35E-30	0.836538462	NA	NA	NA
CCDC141	285025	NA	NA	NA	-2.2293	2.64E-25	0.836538462	-2.069	1.32E-09	0.8
HPX	3263	NA	NA	NA	-2.2492	1.37E-15	0.836538462	NA	NA	NA
SPAG6	9576	NA	NA	NA	-2.1621	1.08E-12	0.836538462	-2.587	9.34E-07	0.84
ATP1A4	480	NA	NA	NA	-2.3344	1.52E-19	0.826923077	NA	NA	NA
AQP4	361	NA	NA	NA	-2.1965	1.82E-12	0.826923077	-2.036	1.33E-05	0.76
RETSAT	54884	NA	NA	NA	-2.0275	3.20E-34	0.817307692	NA	NA	NA
CUX2	23316	NA	NA	NA	-2.3221	4.15E-12	0.817307692	NA	NA	NA
NEGR1	257194	NA	NA	NA	-2.8155	7.85E-32	0.807692308	-2.691	3.70E-14	0.72
APOLD1	81575	NA	NA	NA	-2.1415	2.46E-27	0.807692308	NA	NA	NA
TEPP	374739	NA	NA	NA	-2.2399	1.79E-19	0.807692308	NA	NA	NA
FGFBP2	83888	NA	NA	NA	-2.5261	5.28E-15	0.807692308	-2.66	1.77E-07	0.72
CX3CR1	1524	NA	NA	NA	-3.1143	3.86E-37	0.798076923	-2.734	3.13E-16	0.76
SPATA4	132851	NA	NA	NA	-2.1587	4.27E-29	0.798076923	-2.107	4.44E-11	0.8

EYS	346007	NA	NA	NA	-2.1364	5.75E-25	0.798076923	-2.176	1.41E-09	0.88
SPAG17	200162	NA	NA	NA	-2.5084	2.74E-22	0.798076923	-2.115	2.73E-08	0.64
KERA	11081	NA	NA	NA	-2.366	3.20E-17	0.798076923	-2.013	1.06E-06	0.64
CDR1	1038	NA	NA	NA	-2.2525	5.41E-14	0.798076923	-2.964	2.83E-09	0.92
PLA2G2A	5320	NA	NA	NA	-2.7333	2.74E-09	0.798076923	NA	NA	NA
TNN	63923	NA	NA	NA	-3.5724	2.97E-35	0.788461538	-3.141	2.84E-17	0.72
PNPLA2	57104	NA	NA	NA	-2.1209	1.81E-34	0.788461538	NA	NA	NA
CAB39L	81617	NA	NA	NA	-2.6652	3.72E-59	0.778846154	NA	NA	NA
LOC10012678	100126784	NA	NA	NA	-2.5585	5.32E-30	0.778846154	-2.321	7.40E-12	0.64
STK32A	202374	NA	NA	NA	-2.4441	8.82E-24	0.778846154	-2.706	1.28E-15	0.88
MFAP5	8076	NA	NA	NA	-2.4652	6.08E-18	0.778846154	-2.304	2.45E-07	0.76
RASL10A	10633	NA	NA	NA	-2.1127	9.52E-18	0.778846154	NA	NA	NA
HSD11B1	3290	NA	NA	NA	-2.0405	7.33E-16	0.778846154	NA	NA	NA
PODN	127435	NA	NA	NA	-2.7166	7.63E-31	0.769230769	NA	NA	NA
MSTN	2660	NA	NA	NA	-2.3794	4.94E-29	0.769230769	-2.736	2.04E-25	0.84
LOC10019237	100192378	NA	NA	NA	-2.3119	2.66E-23	0.769230769	-2.083	2.04E-10	0.72
DNAH5	1767	NA	NA	NA	-2.3841	6.64E-17	0.769230769	-2.373	5.88E-06	0.64
HSPA12B	116835	NA	NA	NA	-2.3105	5.16E-43	0.759615385	NA	NA	NA
PCDHGA5	56110	NA	NA	NA	-2.7175	6.89E-36	0.759615385	-2.176	3.44E-15	0.68
CYTL1	54360	NA	NA	NA	-2.4906	1.07E-27	0.759615385	NA	NA	NA
TWIST2	117581	NA	NA	NA	-2.2409	1.93E-24	0.759615385	-2.097	9.82E-10	0.64
KCNMB2	10242	NA	NA	NA	-2.1699	2.04E-20	0.759615385	NA	NA	NA
FAT3	120114	NA	NA	NA	-2.2434	7.90E-18	0.759615385	-2.137	2.18E-09	0.72
PM20D1	148811	NA	NA	NA	-2.0391	1.39E-17	0.759615385	NA	NA	NA
DBC1	1620	NA	NA	NA	-3.2455	9.20E-17	0.759615385	-2.132	0.000370422	0.56
MRGPRX3	117195	NA	NA	NA	-2.4559	1.25E-13	0.759615385	-2.495	6.10E-06	0.8
TMEM45B	120224	NA	NA	NA	-2.3367	3.83E-11	0.759615385	NA	NA	NA
ATP13A4	84239	NA	NA	NA	-2.3341	1.16E-09	0.759615385	NA	NA	NA
HGD	3081	NA	NA	NA	-2.2471	8.59E-09	0.759615385	NA	NA	NA

CLDN8	9073	NA	NA	NA	-2.456	1.29E-08	0.759615385	NA	NA	NA
LOC257358	257358	NA	NA	NA	-2.0329	5.78E-36	0.75	NA	NA	NA
ACSS3	79611	NA	NA	NA	-2.6737	1.46E-28	0.75	-2.871	2.19E-15	0.72
CNTN1	1272	NA	NA	NA	-3.3182	1.41E-26	0.75	-3.012	1.14E-11	0.64
SNTG2	54221	NA	NA	NA	-2.873	3.29E-25	0.75	-2.8	2.12E-12	0.68
CDK15	65061	NA	NA	NA	-2.1858	4.34E-24	0.75	NA	NA	NA
FMN2	56776	NA	NA	NA	-2.4585	1.90E-22	0.75	-2.065	2.45E-10	0.68
GLB1L3	112937	NA	NA	NA	-2.0028	2.40E-15	0.75	NA	NA	NA
KRT32	3882	NA	NA	NA	-2.0393	1.43E-13	0.75	NA	NA	NA
CNRIP1	25927	NA	NA	NA	-2.3232	8.44E-42	0.740384615	NA	NA	NA
DTX1	1840	NA	NA	NA	-2.1705	6.94E-32	0.740384615	-2.082	5.18E-13	0.68
CXCR2	3579	NA	NA	NA	-2.0364	1.35E-22	0.740384615	NA	NA	NA
ADAMTS8	11095	NA	NA	NA	-2.438	2.78E-19	0.740384615	NA	NA	NA
C9orf152	401546	NA	NA	NA	-2.5918	3.48E-11	0.740384615	NA	NA	NA
HP	3240	NA	NA	NA	-2.0808	4.05E-08	0.740384615	NA	NA	NA
FLJ13197	79667	NA	NA	NA	-2.5141	2.61E-39	0.730769231	-2.534	8.18E-19	0.68
HYAL1	3373	NA	NA	NA	-2.2907	2.73E-34	0.730769231	NA	NA	NA
RIC3	79608	NA	NA	NA	-2.8401	1.25E-27	0.730769231	-3.118	3.20E-16	0.6
DNAH7	56171	NA	NA	NA	-2.5768	9.07E-26	0.730769231	-2.61	1.36E-11	0.72
WNT9B	7484	NA	NA	NA	-2.0633	1.21E-25	0.730769231	NA	NA	NA
NRK	203447	NA	NA	NA	-2.6463	2.55E-25	0.730769231	NA	NA	NA
SLC38A4	55089	NA	NA	NA	-2.2529	5.69E-20	0.730769231	NA	NA	NA
STON1-GTF2A	286749	NA	NA	NA	-2.3152	5.29E-11	0.730769231	-2.709	7.14E-06	0.8
DSG1	1828	NA	NA	NA	-2.2122	1.34E-07	0.730769231	NA	NA	NA
BCL8	606	NA	NA	NA	-2.2743	2.74E-35	0.721153846	NA	NA	NA
ANO5	203859	NA	NA	NA	-2.6656	6.54E-25	0.721153846	-3.37	2.74E-23	0.8
INPP5J	27124	NA	NA	NA	-2.0815	2.39E-16	0.721153846	NA	NA	NA
VWCE	220001	NA	NA	NA	-2.1964	1.23E-29	0.711538462	NA	NA	NA
PDLIM3	27295	NA	NA	NA	-2.1717	5.48E-27	0.711538462	NA	NA	NA

C14orf50	145376	NA	NA	NA	-2.2953	1.88E-26	0.711538462	-2.255	1.50E-14	0.68
ANGPT1	284	NA	NA	NA	-2.2213	1.06E-20	0.711538462	-2.229	4.13E-10	0.72
CDH8	1006	NA	NA	NA	-2.012	3.05E-14	0.711538462	-2.293	3.39E-08	0.84
KLF9	687	NA	NA	NA	-2.3346	2.13E-52	0.701923077	NA	NA	NA
IRS1	3667	NA	NA	NA	-2.6571	1.36E-46	0.701923077	-2.235	6.36E-20	0.6
ELTD1	64123	NA	NA	NA	-2.2859	2.84E-42	0.701923077	NA	NA	NA
CYP46A1	10858	NA	NA	NA	-2.2501	2.88E-39	0.701923077	NA	NA	NA
DLC1	10395	NA	NA	NA	-2.3911	1.60E-38	0.701923077	-2.036	2.96E-15	0.6
BAALC	79870	NA	NA	NA	-2.3177	1.14E-28	0.701923077	NA	NA	NA
DOCK11	139818	NA	NA	NA	-2.0027	1.99E-22	0.701923077	NA	NA	NA
F13A1	2162	NA	NA	NA	-2.4005	7.70E-19	0.701923077	NA	NA	NA
TCEAL2	140597	NA	NA	NA	-2.4325	1.08E-14	0.701923077	-2.908	1.67E-12	0.6
RNF39	80352	NA	NA	NA	-2.0302	3.61E-13	0.701923077	NA	NA	NA
RASSF6	166824	NA	NA	NA	-2.1731	3.15E-12	0.701923077	-2.156	0.000113232	0.72
ENPP3	5169	NA	NA	NA	-2.2315	8.58E-11	0.701923077	NA	NA	NA
TMEM35	59353	-2.2761	1.88E-14	0.6896552	-2.4609	9.74E-17	0.625	-2.745	1.53E-16	0.72
LOC10012469:	100124692	-2.0042	0.001744032	0.6896552	NA	NA	NA	-3.285	1.92E-06	0.88
USP44	84101	-2.3294	3.38E-20	0.6551724	-2.8544	9.96E-34	0.682692308	-2.537	2.58E-18	0.72
STXBP6	29091	-2.3773	1.07E-17	0.6551724	NA	NA	NA	-2.738	7.59E-16	0.72
ZNF483	158399	-2.3162	2.40E-16	0.6551724	-2.359	4.20E-32	0.653846154	-2.624	4.97E-17	0.72
GSTM2	2946	-2.0753	3.97E-14	0.6551724	-2.2005	2.14E-24	0.576923077	-2.515	1.75E-21	0.72
ZSCAN23	222696	-2.3246	1.49E-10	0.6551724	-2.5912	2.07E-22	0.682692308	-2.84	2.27E-15	0.72
C1orf190	541468	-2.2871	1.85E-30	0.6206897	-2.4171	2.61E-46	0.663461538	-2.181	2.46E-24	0.72
BMP4	652	-2.39	1.66E-28	0.6206897	-2.9599	1.21E-46	0.682692308	-2.422	5.60E-21	0.76
NBLA00301	79804	-2.1925	1.24E-22	0.6206897	-2.3842	5.41E-29	0.644230769	-3.275	2.17E-26	0.84
ZDBF2	57683	-2.0703	1.40E-16	0.6206897	NA	NA	NA	-2.411	2.76E-22	0.72
EDA2R	60401	-2.1733	4.26E-21	0.5862069	-2.6336	3.05E-41	0.673076923	-2.404	4.97E-19	0.72
DYNC2H1	79659	-2.0429	2.19E-19	0.5172414	-2.1688	1.10E-35	0.576923077	-2.631	5.96E-27	0.76
RNF150	57484	-2.1233	4.84E-10	0.5172414	-2.1199	2.50E-19	0.605769231	-3.096	1.73E-20	0.8

CSMD1	64478	NA	NA	NA	-2.1841	3.18E-11	0.682692308	-2.683	9.27E-10	0.84
PROX1	5629	NA	NA	NA	-2.3912	2.57E-17	0.673076923	-2.473	2.24E-11	0.8
MPPED2	744	NA	NA	NA	-2.4855	1.11E-21	0.653846154	-2.761	9.78E-20	0.72
CASC1	55259	NA	NA	NA	-2.1379	4.00E-20	0.653846154	-2.127	6.98E-10	0.72
NTF3	4908	NA	NA	NA	-2.1646	5.43E-17	0.653846154	-2.753	9.69E-16	0.76
DMRTC1B	728656	NA	NA	NA	-2.1366	4.82E-15	0.644230769	-2.344	5.23E-08	0.8
PAK3	5063	NA	NA	NA	-2.1648	1.38E-16	0.634615385	-3.169	1.07E-14	0.8
NTRK3	4916	NA	NA	NA	-2.6325	5.17E-18	0.625	-3.197	1.65E-17	0.72
ATRNL1	26033	NA	NA	NA	-2.0432	3.00E-12	0.625	-2.723	7.38E-11	0.8
GRIA3	2892	NA	NA	NA	-2.3349	4.60E-24	0.615384615	-2.731	2.46E-17	0.76
MUM1L1	139221	NA	NA	NA	-2.5586	8.73E-22	0.615384615	-2.291	1.03E-12	0.72
CCDC8	83987	NA	NA	NA	-2.1453	6.23E-17	0.596153846	-2.474	1.94E-11	0.76
S100B	6285	NA	NA	NA	-2.2377	2.95E-15	0.557692308	-2.835	4.36E-16	0.84
MCCD1	401250	NA	NA	NA	NA	NA	NA	-2.115	1.27E-06	0.96
GRIN2B	2904	NA	NA	NA	NA	NA	NA	-2.575	1.08E-13	0.92
ABCB11	8647	NA	NA	NA	NA	NA	NA	-2.095	3.29E-07	0.92
CLCN1	1180	NA	NA	NA	NA	NA	NA	-2.047	1.10E-09	0.88
LOC93432	93432	NA	NA	NA	NA	NA	NA	-2.461	3.25E-05	0.88
DDX25	29118	NA	NA	NA	NA	NA	NA	-2.101	2.70E-11	0.84
RORB	6096	NA	NA	NA	NA	NA	NA	-2.371	6.60E-08	0.84
SIM1	6492	NA	NA	NA	NA	NA	NA	-2.592	2.52E-06	0.84
EPHA6	285220	NA	NA	NA	NA	NA	NA	-2.093	3.67E-12	0.8
PMP2	5375	NA	NA	NA	NA	NA	NA	-2.086	6.57E-08	0.8
PAX3	5077	NA	NA	NA	NA	NA	NA	-2.046	3.24E-07	0.8
CLDN2	9075	NA	NA	NA	NA	NA	NA	-2.147	1.11E-08	0.76
CDH19	28513	NA	NA	NA	NA	NA	NA	-2.262	1.98E-07	0.76
KIF5A	3798	NA	NA	NA	NA	NA	NA	-2.546	6.39E-07	0.76
LOC643719	643719	NA	NA	NA	NA	NA	NA	-2.063	1.03E-06	0.76
CLDN10	9071	NA	NA	NA	NA	NA	NA	-2.086	7.86E-05	0.76

?	652919	NA	NA	NA	NA	NA	NA	-2.473	0.00074098	0.76
DAAM2	23500	NA	NA	NA	NA	NA	NA	-2.215	1.49E-18	0.72
OSR1	130497	NA	NA	NA	NA	NA	NA	-2.632	1.39E-13	0.72
PCDH11X	27328	NA	NA	NA	NA	NA	NA	-2.699	1.87E-13	0.72
WBSCR17	64409	NA	NA	NA	NA	NA	NA	-2.091	1.66E-11	0.72
RGS11	8786	NA	NA	NA	NA	NA	NA	-2.257	4.27E-11	0.72
MKX	283078	NA	NA	NA	NA	NA	NA	-2.3	3.19E-09	0.72
KIF5C	3800	NA	NA	NA	NA	NA	NA	-2.045	3.18E-08	0.72
SLC6A15	55117	NA	NA	NA	NA	NA	NA	-2.341	5.62E-08	0.72
KCNH8	131096	NA	NA	NA	NA	NA	NA	-2.622	9.43E-08	0.72
DCX	1641	NA	NA	NA	NA	NA	NA	-2.37	1.18E-05	0.72

Supplementary Table 3a. Up regulated genes in 70% of overall patients

Up regulated genes		StageI			StageII			StageIII			StageIV		
GeneName	GeneID	FC	FDR	frequency	FC	FDR	frequency	FC	FDR	frequency	FC	FDR	frequency
COL10A1	1300	7.0108	7.5E-104	0.9617486	6.7293	1.7E-133	0.9550562	6.9749	1.2E-115	0.9642857	7.22	7.28E-40	1
MMP11	4320	5.9094	3.6E-101	0.9562842	5.7306	4.2E-144	0.9518459	5.9607	1.7E-110	0.9484127	6.4081	5.40E-46	1
IBSP	3381	5.188	8.36E-72	0.9398907	4.9775	1.01E-86	0.929374	4.532	2.25E-60	0.8968254	4.5361	6.86E-33	0.95
COL11A1	1301	6.0578	1.37E-66	0.8852459	6.0138	1.45E-94	0.8876404	5.6999	1.86E-61	0.8452381	6.2155	2.06E-24	0.9
PPAPDC1A	196051	5.3893	1.33E-72	0.8797814	5.2062	8.2E-110	0.8539326	5.395	4.58E-84	0.8769841	5.755	4.58E-27	0.85
NEK2	4751	4.2875	2.62E-71	0.8688525	4.7871	5.6E-164	0.9341894	4.6036	7.82E-90	0.8888889	4.7642	7.12E-22	0.9
KIF4A	24137	3.9983	6.1E-68	0.8469945	4.4924	4.3E-144	0.9069021	4.2867	2.19E-84	0.8531746	4.5004	4.05E-22	0.9
PKMYT1	9088	4.3479	2.91E-66	0.8415301	4.7549	4E-145	0.9101124	4.6512	1.07E-84	0.8809524	4.8181	9.87E-19	0.9
CCL11	6356	3.8973	1.76E-56	0.8415301	3.5689	8.05E-77	0.8282504	3.6646	3.34E-66	0.8452381	3.0266	6.97E-20	0.85
CST4	1472	4.4024	9.95E-38	0.8360656	4.0355	5.24E-40	0.776886	4.1458	6.65E-41	0.7896825	3.9748	5.24E-23	0.9
LOC283867	283867	3.5183	3.27E-55	0.8306011	3.1431	8.15E-59	0.7624398	3.5041	8.6E-63	0.8888889	3.0088	3.48E-20	0.75
MMP13	4322	6.3392	1.01E-62	0.8196721	6.1997	3.74E-94	0.8330658	5.9266	2.04E-59	0.7777778	5.5327	1.73E-18	0.85
GJB2	2706	3.9487	1.14E-53	0.8196721	3.7813	2.45E-71	0.7849117	3.5903	9.22E-51	0.7579365	4.0812	3.96E-24	0.85
CST1	1469	6.7906	7.4E-58	0.7978142	6.1852	1.44E-63	0.741573	6.097	2.35E-50	0.7142857	6.4704	1.98E-24	0.85
CHRNA6	8973	3.3536	1.22E-52	0.7978142	2.8616	5.1E-49	0.6966292	2.9648	4.83E-44	0.7103175	2.2157	1.84E-10	0.55
KIAA0101	9768	3.4215	1.71E-63	0.7923497	3.6645	3.7E-134	0.847512	3.6065	2.48E-77	0.8253968	3.343	1.08E-15	0.8
CDC25C	995	3.681	1.26E-61	0.7923497	4.0954	1.1E-137	0.8860353	3.9458	1.97E-74	0.8333333	3.836	1.13E-17	0.85
COMP	1311	4.9131	9.94E-57	0.7923497	4.6839	7.93E-67	0.7897271	5.018	1.2E-70	0.8412698	5.3806	7.73E-18	0.9
UBE2C	11065	3.8471	1.98E-60	0.7868852	4.3677	1.7E-123	0.8651685	4.2296	2.85E-76	0.8293651	4.4257	1.81E-21	0.85
SLC24A2	25769	3.7599	3.34E-47	0.7868852	3.3417	4.82E-48	0.7399679	3.7166	6.3E-52	0.7936508	3.2911	5.87E-19	0.7
HSD17B6	8630	2.9981	1.46E-69	0.7759563	2.9923	1.9E-109	0.7752809	3.0441	2.91E-79	0.7738095	3.1367	1.46E-22	0.7
GABRD	2563	3.3314	8.86E-68	0.7759563	3.3708	8.6E-102	0.7784912	3.5661	3.33E-89	0.8452381	3.7349	2.91E-25	0.9
UHRF1	29128	3.517	1.15E-60	0.7759563	3.8534	1.3E-128	0.8218299	3.677	7.15E-72	0.8055556	3.525	8.89E-15	0.75
PLAC1	10761	3.4124	1.55E-45	0.7759563	3.6356	5.03E-63	0.7817014	3.6258	9.25E-61	0.8253968	3.8489	1.13E-23	0.9
IQGAP3	128239	3.6539	1.06E-61	0.7704918	4.1742	1.3E-135	0.8892456	3.9635	2.07E-79	0.8373016	3.8176	1.06E-18	0.75
HAGHL	84264	3.705	7.52E-56	0.7704918	3.6691	2.28E-88	0.7977528	3.8547	5.2E-70	0.8253968	4.0194	6.56E-20	0.85
PROC	5624	2.8988	3.67E-56	0.7650273	2.616	4.65E-65	0.6548957	2.6238	2.09E-52	0.6944444	2.5637	1.72E-15	0.7
EPYC	1833	4.3669	1.31E-42	0.7650273	4.2798	1.58E-51	0.7447833	3.8781	2.85E-35	0.6746032	3.9705	1.05E-20	0.75
LRRC15	131578	3.5897	2.82E-44	0.7595628	3.4235	1.25E-57	0.7094703	3.4974	1.65E-48	0.734127	3.6855	1.89E-15	0.75
WISP1	8840	3.4254	4.99E-58	0.7540984	3.2086	1.06E-79	0.7191011	3.3937	1.02E-66	0.781746	3.1736	2.12E-19	0.7

MELK	9833	3.5404	2.23E-55	0.7486339	4.0269	5.9E-113	0.8266453	3.7694	1.01E-69	0.7777778	3.8765	1.69E-18	0.85
CST2	1470	4.1664	5.99E-47	0.7486339	3.557	5.9E-40	0.6597111	4.1957	2.77E-51	0.781746	3.4976	5.76E-15	0.65
NUF2	83540	3.6044	7.42E-63	0.7431694	4.105	7.8E-126	0.8699839	3.8403	4.03E-78	0.8293651	3.9091	6.39E-22	0.8
SCT	6343	2.9578	6.03E-44	0.7377049	2.8547	1.01E-52	0.7239165	2.9813	8.51E-45	0.7103175	3.727	4.40E-33	0.95
PITX1	5307	4.0009	1.44E-34	0.726776	4.1574	2.29E-59	0.7431782	4.5536	7.66E-64	0.8055556	4.5456	1.38E-16	0.8
CILP2	148113	3.0655	9.01E-56	0.7213115	2.9239	1.74E-64	0.6773676	3.1375	7.47E-57	0.7261905	2.9634	2.09E-20	0.7
SPC24	147841	2.9659	2.58E-50	0.7213115	3.4491	6.41E-94	0.7961477	3.2569	2.49E-62	0.7460317	3.528	4.57E-22	0.85
MMP1	4312	5.0812	5.87E-43	0.7213115	5.412	7.97E-65	0.7287319	5.0486	7.17E-43	0.6666667	5.8872	1.14E-20	0.7
TROAP	10024	3.4835	1.22E-56	0.715847	3.9976	2.7E-119	0.8346709	3.8712	6.27E-75	0.8253968	4.0159	7.51E-21	0.85
ADAMTS14	140766	3.5688	2.11E-54	0.715847	3.4343	2.49E-96	0.6757624	3.4081	4.38E-60	0.6825397	3.7818	6.70E-18	0.7
TDO2	6999	3.8047	1.53E-52	0.715847	3.8951	6.81E-74	0.7094703	3.6845	4.63E-50	0.6428571	4.4552	2.09E-21	0.85
PLK1	5347	3.1171	2.36E-59	0.7103825	3.6349	6.9E-115	0.8154093	3.4465	1.29E-72	0.797619	3.7036	4.40E-24	0.8
KIF20A	10112	3.515	1.14E-55	0.7103825	3.9111	5.1E-122	0.8170144	3.7218	1.09E-66	0.7777778	3.793	5.28E-17	0.75
WBSCR28	135886	2.4543	1.17E-32	0.7103825	2.4124	1.57E-41	0.7303371	2.3185	1.04E-36	0.7460317	3.0337	4.13E-24	0.8
TPX2	22974	3.0468	1.01E-60	0.704918	3.5743	2.7E-120	0.8218299	3.3431	1.57E-74	0.7936508	3.3581	3.63E-24	0.85
SPC25	57405	3.0119	2.6E-60	0.704918	3.4023	8.5E-130	0.8346709	3.2262	9.68E-77	0.797619	3.2002	1.43E-17	0.85
OR2B6	26212	2.1441	3.58E-30	0.704918	2.426	1.64E-46	0.7865169	2.2888	7.01E-36	0.7222222	2.457	1.08E-18	0.8
HJURP	55355	3.315	6.95E-60	0.6994536	3.747	1.6E-121	0.8314607	3.5342	8.8E-73	0.7936508	3.4138	5.57E-19	0.8
PBK	55872	3.2549	6.11E-53	0.6721311	3.8287	5.3E-118	0.8282504	3.6466	1.36E-67	0.7698413	3.8066	6.79E-17	0.85
DLGAP5	9787	3.4285	3.54E-53	0.6721311	3.9114	4.9E-120	0.8202247	3.7007	1.48E-65	0.765873	3.6621	1.70E-15	0.8
CEP55	55165	3.0932	1.69E-56	0.6830601	3.5839	1.4E-113	0.8105939	3.2671	2.77E-68	0.7380952	3.3504	3.24E-19	0.8
BUB1	699	3.165	5.88E-53	0.6612022	3.704	1.6E-115	0.8073836	3.436	1.79E-68	0.7777778	3.5556	3.59E-19	0.8
ASPM	259266	3.449	1.1E-50	0.6557377	3.9325	6.6E-111	0.8073836	3.6557	4.72E-62	0.7857143	3.3676	1.10E-13	0.65
EXO1	9156	3.2152	6.37E-51	0.6229508	3.8052	3.3E-107	0.8073836	3.5553	7.3E-66	0.781746	3.5052	3.78E-16	0.75
UBE2T	29089	2.7813	7.19E-63	0.6721311	3.2724	1.4E-124	0.8057785	3.0928	7.29E-78	0.7738095	3.2962	3.82E-26	0.75
NEIL3	55247	3.2468	4.7E-51	0.6775956	3.7542	6.1E-107	0.8057785	3.5783	4.8E-65	0.7738095	3.3768	9.68E-16	0.8
KIF18B	146909	3.4873	8.28E-54	0.6994536	3.9651	1.5E-112	0.8025682	3.6843	6.15E-66	0.7619048	3.6931	4.57E-16	0.75
ASF1B	55723	2.8066	1.74E-60	0.6612022	3.1451	1.6E-126	0.8009631	2.9425	1.82E-74	0.7380952	2.9792	3.99E-19	0.8
HMMR	3161	3.1319	6.35E-56	0.6666667	3.5401	8.1E-122	0.7961477	3.3303	7.3E-66	0.7539683	3.223	5.85E-16	0.7
KIF2C	11004	3.1211	3.16E-54	0.6721311	3.6176	5.7E-107	0.7945425	3.3701	4.5E-67	0.7579365	3.4924	5.43E-18	0.75
DTL	51514	2.7969	3.42E-51	0.6065574	3.2013	1.8E-115	0.7913323	3.0443	2.48E-67	0.7301587	3.0002	1.82E-16	0.7
NDC80	10403	3.0938	3.19E-56	0.6721311	3.5195	2E-108	0.7913323	3.2336	2.58E-65	0.7380952	3.3569	1.72E-18	0.75
CDK1	983	2.7341	1.26E-62	0.6284153	3.1779	2E-119	0.7849117	2.9497	2.45E-67	0.734127	3.0024	2.57E-23	0.75

KIF14	9928	3.2537	3.56E-48	0.6174863	3.8661	9E-102	0.7849117	3.6106	1.65E-61	0.7460317	3.5222	1.35E-16	0.75
FOXM1	2305	3.0491	2.59E-53	0.6174863	3.617	2.6E-101	0.7849117	3.3321	3.36E-68	0.7460317	3.5012	1.79E-20	0.7
RRM2	6241	3.164	4.08E-53	0.6721311	3.6824	1.3E-113	0.7817014	3.4929	1.62E-63	0.7380952	3.5346	3.24E-16	0.75
ANLN	54443	2.9976	1.74E-51	0.6393443	3.4649	3.7E-102	0.776886	3.2817	6.21E-60	0.734127	3.3226	1.58E-18	0.8
EPR1	8475	3.4603	6.1E-50	0.6393443	3.9666	3.43E-95	0.776886	3.7377	4.89E-56	0.7261905	3.8335	2.17E-13	0.65
NUSAP1	51203	2.8646	2.75E-58	0.6666667	3.2254	7.2E-118	0.7752809	3.1022	1.89E-70	0.765873	2.9698	3.05E-17	0.75
KIFC1	3833	2.8388	3.33E-52	0.6120219	3.2502	4.1E-105	0.7752809	3.0692	7.04E-68	0.7261905	3.1799	2.58E-17	0.75
CCNB2	9133	2.9737	3.77E-52	0.6393443	3.4687	1.9E-109	0.7720706	3.2807	2.05E-68	0.734127	3.3612	3.42E-18	0.75
CENPF	1063	2.812	2.48E-49	0.5846995	3.2889	9.9E-109	0.7720706	3.0315	1.18E-64	0.718254	2.9809	1.20E-16	0.65
CKAP2L	150468	3.0056	8.91E-52	0.6120219	3.4586	2.8E-102	0.7720706	3.2276	5.74E-60	0.718254	3.1361	5.16E-17	0.75
SGOL1	151648	2.8652	1.85E-44	0.5901639	3.45	8.03E-96	0.7688604	3.2172	3.21E-58	0.7261905	3.1172	8.31E-17	0.65
SKA1	220134	2.8141	1.78E-52	0.6393443	3.2921	2.15E-94	0.7672552	2.9972	3.85E-60	0.6746032	3.4037	4.98E-24	0.85
PTTG1	9232	2.7849	1.81E-54	0.6612022	3.2063	2.7E-109	0.7640449	3.0479	8.72E-70	0.7380952	3.2278	1.58E-22	0.8
TOP2A	7153	3.3714	5.61E-52	0.6284153	3.8355	4.1E-107	0.7640449	3.5734	3.69E-60	0.7380952	3.2335	3.94E-13	0.7
CDC20	991	3.1299	1.27E-50	0.6721311	3.6207	9.3E-99	0.7640449	3.3644	7.73E-63	0.718254	3.6006	1.15E-17	0.8
TK1	7083	2.7575	2.7E-59	0.6612022	3.1455	1.6E-113	0.752809	3.0293	2.88E-71	0.6944444	3.1353	7.00E-18	0.7
BUB1B	701	2.9415	2.13E-53	0.6229508	3.3454	8E-108	0.7495987	3.1743	8.09E-62	0.718254	3.1452	4.32E-15	0.8
BIRC5	332	3.4123	5.38E-49	0.6284153	4.0377	4E-104	0.7495987	3.7599	7.07E-61	0.7261905	3.9089	2.45E-15	0.65
CNIH2	254263	2.9947	9.46E-46	0.6174863	3.4205	4.37E-84	0.7479936	3.2204	2.2E-60	0.702381	3.563	6.41E-22	0.8
HIST1H2BO	8348	2.4438	2.44E-35	0.6775956	2.6414	5.3E-56	0.7479936	2.5467	1.33E-43	0.7380952	2.6762	1.70E-18	0.8
C16orf59	80178	2.6554	2.12E-53	0.6393443	3.0106	1.4E-113	0.7463884	2.9584	1.29E-72	0.7380952	3.1164	3.59E-19	0.8
DEPDC1B	55789	2.8133	2.47E-47	0.6284153	3.2458	1.46E-96	0.7447833	2.9819	1.88E-60	0.6984127	2.9516	1.48E-15	0.75
RDM1	201299	2.6414	8.72E-38	0.6229508	3.0869	7.1E-75	0.7447833	2.7224	1.79E-43	0.6071429	3.0073	1.93E-16	0.75
CDKN3	1033	2.7119	2.73E-57	0.6120219	3.0648	9.2E-111	0.7431782	2.9907	1.71E-70	0.734127	2.8955	6.45E-19	0.7
GTSE1	51512	2.7384	1.27E-51	0.5901639	3.1784	1.1E-100	0.741573	2.9579	5.79E-64	0.6904762	3.0943	1.11E-19	0.7
SKA3	221150	2.7756	1.45E-52	0.5846995	3.2198	2.3E-101	0.7399679	3.0285	8.54E-63	0.718254	3.0428	1.43E-17	0.8
CDC45	8318	3.1854	2.44E-45	0.6010929	3.6631	5.45E-95	0.7399679	3.5007	6.58E-60	0.7103175	3.4685	4.33E-13	0.7
AURKB	9212	3.1465	3.88E-48	0.6120219	3.6629	1.08E-91	0.7399679	3.4385	3.99E-60	0.6984127	3.8108	4.97E-17	0.75
MYBL2	4605	3.3266	4.91E-43	0.6448087	3.8879	2.02E-85	0.7383628	3.6964	1.03E-56	0.7063492	3.8921	6.58E-18	0.8
NCAPG	64151	2.7428	6.4E-54	0.579235	3.136	4.1E-106	0.7303371	2.9046	4.61E-60	0.6706349	2.8501	6.20E-17	0.7
CENPA	1058	2.8008	2.76E-46	0.6065574	3.2923	9.36E-86	0.7287319	2.9673	2.71E-54	0.6825397	3.1872	4.12E-18	0.75
CDCA5	113130	2.5393	6.42E-51	0.5519126	3.0121	2.82E-98	0.717496	2.8603	9.39E-67	0.6865079	3.1345	1.33E-22	0.7
FAM83D	81610	2.7954	1.18E-47	0.6065574	3.3828	3.95E-89	0.7158909	3.1769	1.27E-57	0.7063492	3.2443	6.61E-20	0.8

CDCA3	83461	2.6158	2.06E-50	0.5846995	3.082	2.01E-94	0.7142857	2.8285	2.06E-64	0.6706349	2.8099	5.30E-19	0.65
MKI67	4288	2.8789	8.24E-47	0.5300546	3.3362	5.8E-100	0.7110754	3.1078	1.12E-57	0.6706349	3.2507	2.34E-15	0.75
CDCA8	55143	2.5378	2.85E-54	0.5846995	2.9209	1.2E-99	0.7094703	2.6944	1.65E-64	0.6785714	2.7752	1.77E-21	0.75
AURKA	6790	2.4687	5.22E-56	0.557377	2.949	2E-104	0.7078652	2.8296	3.05E-63	0.6547619	2.9257	1.09E-27	0.7
DEPDC1	55635	2.7771	3.78E-44	0.5464481	3.2796	8.19E-88	0.7078652	2.9935	1.7E-53	0.6626984	2.4005	2.51E-10	0.65
RAD54L	8438	2.7968	6.05E-50	0.6120219	3.2033	1.65E-91	0.70626	2.9903	2.83E-61	0.6904762	3.2312	6.94E-18	0.7
HIST1H2AM	8336	2.9095	9.61E-44	0.6830601	2.9597	1.12E-65	0.7030498	3.031	2.88E-52	0.7301587	3.4674	1.38E-21	0.85
GRM4	2914	4.2165	1.51E-39	0.6721311	4.1112	5.77E-50	0.6725522	4.3081	3.99E-50	0.7420635	4.1218	4.48E-20	0.75
C19orf21	126353	3.3743	8.72E-28	0.6666667	3.1826	8.8E-33	0.6227929	3.7824	5.03E-43	0.7261905	3.8966	5.61E-10	0.65
CCDC78	124093	3.2543	2.02E-37	0.6502732	3.2664	1.57E-54	0.6452648	3.4233	5.36E-48	0.7103175	3.5246	3.09E-15	0.75
HIST2H3C	126961	2.1824	7.43E-34	0.5901639	2.3125	9.01E-49	0.6677368	2.477	2.18E-43	0.702381	2.9632	3.31E-24	0.85
GAGE12D	1E+08	NA	NA	NA	NA	NA	NA	NA	NA	NA	2.0207	3.64E-06	1
HIST1H3J	8356	NA	NA	NA	NA	NA	NA	NA	NA	NA	2.0698	6.08E-29	0.9
HCN2	610	2.4382	1.61E-38	0.5355191	2.7893	3.4E-47	0.5987159	2.8441	3.93E-44	0.6587302	3.6688	4.97E-26	0.9
HIST1H3H	8357	2.9906	1.45E-37	0.6229508	3.1407	3.88E-50	0.64687	3.3008	6.02E-42	0.6904762	3.9221	3.51E-24	0.9
SAMD14	201191	2.4031	2.2E-50	0.5136612	2.3833	5.97E-79	0.5521669	2.4629	2.49E-58	0.5714286	3.0411	2.01E-21	0.85
HOXC13	3229	2.4635	4.68E-16	0.5737705	2.5726	7.8E-25	0.600321	2.8148	5.13E-23	0.6587302	3.4855	9.09E-10	0.85
FAM72D	728833	2.0872	3.35E-35	0.3879781	2.6222	9.12E-69	0.5955056	2.3525	7.32E-45	0.5238095	2.7808	1.88E-21	0.8
AIM1L	55057	2.9167	8.38E-38	0.6338798	3.2084	7.76E-55	0.6789727	3.1516	1.34E-46	0.6785714	3.7047	4.56E-21	0.8
LHX2	9355	2.0242	3.29E-21	0.4808743	2.5009	6.32E-32	0.5810594	2.2432	9.16E-26	0.5634921	2.8529	4.67E-19	0.8
FABP6	2172	NA	NA	NA	NA	NA	NA	NA	NA	NA	3.1259	1.44E-18	0.8
CTHRC1	115908	2.5609	5.11E-47	0.5737705	2.3789	3.27E-63	0.5329053	2.5263	4.7E-53	0.5952381	2.8051	9.30E-16	0.8
CSAG1	158511	NA	NA	NA	NA	NA	NA	NA	NA	NA	2.9588	1.09E-15	0.8
CCDC64	92558	2.9789	5.68E-52	0.6612022	3.0277	5.12E-93	0.6661316	3.0417	1.08E-64	0.6428571	3.3373	1.63E-14	0.8
SRMS	6725	2.6082	5.45E-27	0.5956284	2.3292	3.4E-27	0.5120385	2.6575	1.06E-31	0.5396825	3.4978	2.25E-12	0.8
FAM72B	653820	NA	NA	NA	2.4843	4.23E-67	0.5826645	2.1858	1.87E-46	0.5	2.5311	6.45E-22	0.75
CKS2	1164	2.2929	1.73E-61	0.5464481	2.5695	1.2E-105	0.6227929	2.4005	1.48E-68	0.5555556	2.5883	1.17E-20	0.75
ZWINT	11130	2.28	3.72E-55	0.5136612	2.6285	1.4E-108	0.6548957	2.4843	2.03E-66	0.6031746	2.7751	1.19E-20	0.75
GNG13	51764	2.6125	1.25E-26	0.6010929	2.8434	1.26E-30	0.6211878	3.1012	1.85E-33	0.6825397	3.186	2.44E-19	0.75
VGF	7425	2.1141	5.95E-20	0.4754098	2.791	4.2E-35	0.6179775	2.7653	6.63E-29	0.5952381	3.4599	1.06E-18	0.75
HIST1H3D	8351	3.139	1.75E-32	0.5136612	3.3643	8.19E-60	0.5730337	3.3831	6.79E-39	0.5952381	4.1246	8.57E-18	0.75
S100P	6286	4.3542	3.35E-34	0.6174863	4.5286	4.17E-46	0.600321	4.6444	8.84E-41	0.6388889	5.1686	1.98E-17	0.75
CCDC19	25790	2.632	8.85E-40	0.6174863	2.6779	2.68E-52	0.5858748	2.8064	1.46E-50	0.6190476	3.197	9.52E-17	0.75

MCM10	55388	2.5569	5.28E-38	0.5081967	3.0918	1.05E-71	0.670947	2.8551	7.39E-47	0.6507937	3.0143	9.74E-17	0.75
PAFAH1B3	5050	2.4978	2.08E-59	0.5519126	2.5348	3.5E-113	0.5810594	2.5794	4.3E-73	0.5992063	2.763	2.32E-16	0.75
HIST3H2BB	128312	NA	NA	NA	NA	NA	NA	NA	NA	NA	2.4233	1.01E-15	0.75
FAM64A	54478	2.7525	7.38E-41	0.5245902	3.2575	2.53E-86	0.6757624	2.9822	6.92E-55	0.6031746	3.4113	1.04E-15	0.75
HOXC11	3227	2.7653	4.34E-16	0.6174863	2.9701	1.43E-23	0.64687	3.1533	7.19E-24	0.6349206	3.9606	1.97E-12	0.75
HOXC10	3226	NA	NA	NA	NA	NA	NA	NA	NA	NA	2.5853	4.43E-10	0.75
UBE2S	27338	2.0475	1.66E-50	0.4699454	2.4021	3.47E-82	0.5698234	2.2812	1.34E-61	0.4722222	2.5758	5.68E-25	0.7
CCNB1	891	2.1049	3.87E-57	0.5081967	2.4467	9.7E-106	0.6195827	2.3318	5.63E-68	0.5952381	2.4044	2.36E-23	0.7
FAM54A	113115	2.3483	2.41E-47	0.5355191	2.8513	6.73E-82	0.6773676	2.624	9.37E-62	0.6230159	2.7497	4.80E-22	0.7
RAD51	5888	2.3124	3.53E-52	0.5464481	2.6612	8.33E-95	0.6565008	2.593	3.66E-64	0.6230159	2.6737	2.20E-20	0.7
TUBB3	10381	2.4755	3.67E-37	0.4754098	2.7221	4.23E-55	0.529695	2.5575	1.53E-37	0.4722222	3.2367	6.94E-20	0.7
OIP5	11339	2.4877	1.33E-54	0.5956284	2.8351	1.7E-101	0.6934189	2.7303	2.14E-67	0.6428571	2.7387	6.13E-19	0.7
ERCC6L	54821	2.4158	3.09E-49	0.5355191	2.7792	3.78E-91	0.6902087	2.6092	2.34E-57	0.6507937	2.7122	7.51E-18	0.7
CCNA2	890	2.3676	6.15E-47	0.4972678	2.8207	1.14E-87	0.6532905	2.6315	8.2E-56	0.6428571	2.6541	1.26E-17	0.7
SYNGR3	9143	2.2565	8.49E-31	0.5191257	2.5648	2.2E-50	0.5794543	2.3874	2.36E-39	0.5238095	3.1194	3.70E-17	0.7
NCAPH	23397	2.5147	7.92E-48	0.5409836	2.9786	8.01E-95	0.6757624	2.754	7.47E-59	0.6587302	2.8065	1.55E-16	0.7
MAPK8IP2	23542	2.1071	9.52E-23	0.4644809	2.2485	2.51E-30	0.5168539	2.3306	4.65E-31	0.5119048	3.1589	1.17E-15	0.7
KIF23	9493	2.3894	4.86E-49	0.5628415	2.7665	7.03E-95	0.6725522	2.5881	5.16E-58	0.6150794	2.5537	1.48E-15	0.7
ESPL1	9700	2.6827	6.8E-44	0.5191257	3.088	4.52E-94	0.6918138	2.9615	1.3E-55	0.6706349	3.1017	6.95E-15	0.7
HAPLN1	1404	3.4572	1.46E-28	0.6065574	3.4128	2.58E-39	0.6227929	3.0151	5.57E-26	0.5436508	3.6427	1.82E-14	0.7
CENPM	79019	2.846	7.73E-49	0.6065574	3.1824	1.4E-105	0.6837881	3.1288	1.52E-62	0.6666667	3.3542	6.05E-14	0.7
CTXN1	404217	2.8336	8.43E-47	0.6065574	2.8507	7.48E-83	0.611557	2.85	2.38E-53	0.5793651	2.897	1.37E-13	0.7
RTBDN	83546	NA	NA	NA	2.6286	2.81E-23	0.6292135	2.4448	1.78E-19	0.6190476	2.4418	6.41E-12	0.7
C20orf103	24141	3.2015	1.03E-41	0.6502732	2.8238	3.81E-41	0.5778491	3.2807	1.03E-48	0.6547619	3.138	1.16E-10	0.7
CNTNAP2	26047	3.6292	4.47E-25	0.5519126	3.1036	8.23E-21	0.4783307	3.8264	4.08E-29	0.5873016	3.7026	5.29E-09	0.7

Note: NA denotes the gene is not up regulated in that cancer stage.

Supplementary Table 3b. Down regulated genes in 70% of overall patients

Down-regulated genes		StageI			StageII			StageIII			StageIV		
GeneName	GeneID	FC	FDR	frequency	FC	FDR	frequency	FC	FDR	frequency	FC	FDR	frequency
FHL1	2273	-4.145	1.18E-62	1	-4.4389	5.48E-119	0.993579	-4.169	4.52E-75	0.988095	-4.615	5.64E-19	1
GLYAT	10249	-5.924	6.39E-62	1	-6.5306	1.86E-121	0.993579	-6.388	1.08E-75	0.992063	-7.015	6.22E-21	1
PYGM	5837	-3.613	1.46E-57	1	-3.693	4.55E-107	1	-3.457	8.54E-63	1	-3.539	8.79E-14	1
TRDN	10345	-4.473	2.84E-52	1	-4.2383	1.82E-74	0.991974	-4.056	1.83E-49	1	-4.307	5.14E-10	1
MYH1	4619	-3.852	1.18E-47	1	-3.9602	2.82E-98	0.99679	-3.684	6.92E-50	0.996032	-3.957	1.92E-09	1
TNNT3	7140	-3.956	1.04E-40	1	-4.3767	1.57E-62	0.995185	-3.809	5.24E-38	0.992063	-4.789	2.26E-17	1
FIGF	2277	-6.145	1.94E-87	0.994536	-6.4651	1.52E-142	0.982343	-6.094	1.59E-90	0.968254	-6.652	4.49E-44	1
MYOM1	8736	-3.776	5.04E-72	0.994536	-3.9926	2.77E-123	1	-3.889	2.82E-79	0.996032	-3.979	1.17E-20	1
MYOC	4653	-6.046	1.53E-63	0.994536	-6.559	3.58E-140	0.993579	-6.376	5.54E-85	1	-7.181	6.07E-21	1
LEP	3952	-6.83	5.10E-52	0.994536	-7.6419	8.81E-96	0.988764	-7.368	1.22E-62	0.992063	-8.241	1.18E-20	1
HSPB7	27129	-4.319	7.46E-52	0.994536	-4.5585	1.14E-97	0.988764	-4.208	7.45E-60	0.984127	-4.436	1.22E-12	1
DEFB132	400830	-3.968	1.23E-35	0.994536	-4.559	2.05E-64	0.974318	-4.486	2.82E-48	0.984127	-4.924	1.17E-11	1
DES	1674	-3.744	1.47E-33	0.994536	-3.9831	1.27E-47	0.979133	-3.398	1.23E-27	0.980159	-4.005	5.93E-10	1
AMPD1	270	-2.857	3.45E-25	0.994536	-2.7874	6.24E-35	0.974318	-2.592	1.09E-22	0.972222	-2.981	2.60E-06	0.95
LOC283392	283392	-5.514	8.53E-60	0.989071	-5.997	1.07E-113	0.99679	-5.622	7.31E-66	0.988095	-6.506	1.16E-20	1
ACVR1C	130399	-4.692	3.49E-56	0.989071	-5.0639	3.98E-106	0.995185	-4.967	2.05E-68	0.992063	-5.155	2.98E-16	1
KIAA0408	9729	-5.956	5.69E-56	0.989071	-6.1506	7.12E-93	0.985554	-5.665	7.17E-59	0.97619	-6.701	3.35E-22	1
CA3	761	-4.294	1.36E-52	0.989071	-4.7378	3.04E-78	0.991974	-4.855	2.23E-59	0.996032	-5.116	1.33E-17	1
OXTR	5021	-4.182	6.49E-40	0.989071	-4.3595	1.96E-79	0.956661	-4.133	7.73E-44	0.960317	-4.455	8.58E-09	1
GYS2	2998	-3.354	3.43E-38	0.989071	-3.4116	5.67E-83	0.988764	-3.531	2.05E-51	0.988095	-3.765	7.15E-09	1
GPAM	57678	-3.569	4.42E-62	0.983607	-3.6716	3.13E-133	0.985554	-3.644	3.86E-78	0.984127	-3.845	1.39E-14	1
APOB	338	-5.136	2.67E-59	0.983607	-5.544	1.66E-112	0.990369	-5.443	5.22E-75	0.996032	-5.614	3.42E-17	1
GLRA4	441509	-3.297	8.97E-57	0.983607	-3.2605	2.15E-104	0.94703	-3.184	3.65E-59	0.944444	-3.296	7.59E-12	0.95
LHCGR	3973	-3.404	2.00E-46	0.983607	-3.5341	4.60E-111	0.977528	-3.482	4.06E-58	0.960317	-3.804	3.66E-11	1
ADH1B	125	-5.896	8.85E-43	0.983607	-7.4792	8.66E-66	0.987159	-6.53	3.36E-47	0.97619	-7.524	4.60E-22	1
C12orf39	80763	-4.447	7.12E-41	0.983607	-4.6661	4.30E-89	0.979133	-4.763	4.60E-56	0.984127	-4.538	6.59E-09	0.95
SMYD1	150572	-5.144	8.14E-40	0.983607	-5.6981	4.50E-79	0.977528	-5.64	1.25E-53	0.97619	-5.867	1.29E-11	0.9
LOC729467	729467	-2.228	3.56E-37	0.983607	-2.2097	1.24E-90	0.982343	-2.24	1.76E-49	0.996032	-2.335	5.52E-07	1
MYOM2	9172	-2.135	5.05E-24	0.983607	-2.555	1.17E-47	0.991974	-2.239	1.02E-28	0.984127	-2.579	1.38E-08	1
LYVE1	10894	-4.762	6.10E-68	0.978142	-5.1902	3.65E-141	0.988764	-4.897	1.63E-80	0.992063	-4.904	1.01E-19	0.95
ADRA1A	148	-4.236	1.54E-67	0.978142	-4.2498	1.71E-110	0.974318	-4.264	6.60E-78	0.988095	-4.196	3.40E-17	1

SLC2A4	6517	-4.324	6.09E-67	0.978142	-4.5512	3.43E-109	0.982343	-4.186	1.41E-73	0.992063	-4.451	3.17E-19	1
HEPACAM	220296	-5.184	7.38E-62	0.978142	-5.7882	9.86E-111	0.987159	-5.434	1.99E-68	0.984127	-5.708	9.22E-22	1
AQP7P1	375719	-5.174	7.91E-62	0.978142	-5.7938	6.67E-121	0.991974	-5.548	1.11E-73	0.984127	-5.941	4.93E-22	1
CKMT2	1160	-3.409	1.46E-57	0.978142	-3.5543	7.91E-72	0.969502	-3.332	1.45E-59	0.968254	-3.418	1.70E-16	0.95
KCNIP2	30819	-4.666	1.74E-54	0.978142	-5.2241	2.31E-111	0.995185	-4.951	2.35E-66	0.980159	-5.159	5.28E-16	1
ADH1A	124	-4.951	1.76E-54	0.978142	-5.7898	1.92E-105	0.987159	-5.327	1.97E-64	0.980159	-5.518	4.87E-19	1
AQPEP	206338	-4.566	3.58E-54	0.978142	-4.839	1.18E-109	0.998395	-4.715	6.44E-64	0.980159	-5.088	1.40E-15	1
AQP7P3	441432	-3.67	4.60E-50	0.978142	-3.9221	6.86E-119	0.982343	-3.781	6.59E-63	0.988095	-3.94	9.49E-12	1
GPD1	2819	-5.488	1.05E-48	0.978142	-6.4797	1.28E-84	0.985554	-6.056	1.13E-57	0.97619	-6.367	7.11E-19	1
SLC19A3	80704	-4.859	1.55E-46	0.978142	-5.6884	2.30E-81	0.988764	-5.435	4.63E-53	0.968254	-5.889	9.40E-17	1
HBB	3043	-4.049	3.26E-44	0.978142	-4.5872	3.48E-90	0.990369	-4.191	3.37E-48	0.97619	-4.52	1.05E-12	0.95
HBA2	3040	-3.629	1.12E-39	0.978142	-4.0269	6.26E-78	0.977528	-3.549	2.08E-40	0.936508	-3.665	1.37E-09	0.9
ADIPOQ	9370	-5.735	2.21E-38	0.978142	-7.2661	6.89E-61	0.985554	-6.586	2.43E-44	0.972222	-8.315	1.32E-19	1
CSN1S1	1446	-3.399	7.21E-22	0.978142	-3.6912	8.71E-61	0.987159	-3.778	1.82E-40	0.992063	-4.156	7.27E-07	1
LALBA	3906	-3.059	6.59E-17	0.978142	-2.9488	3.70E-29	0.985554	-3.177	1.40E-25	0.988095	-2.707	0.004343	1
CPA1	1357	-4.356	3.73E-81	0.972678	-4.5694	3.92E-151	0.980738	-4.297	4.22E-86	0.968254	-4.222	1.36E-22	1
CA4	762	-6.629	7.19E-78	0.972678	-7.3688	1.26E-156	0.985554	-6.939	1.49E-92	0.97619	-7.117	8.85E-38	1
CD300LG	146894	-5.939	3.74E-69	0.972678	-6.9294	9.86E-112	0.988764	-6.368	4.04E-77	0.988095	-6.941	2.27E-39	1
SCARA5	286133	-6.008	3.15E-63	0.972678	-6.7556	3.52E-105	0.987159	-6.447	3.33E-75	0.972222	-6.801	5.34E-34	1
LPL	4023	-4.486	1.20E-58	0.972678	-4.7867	4.53E-103	0.979133	-4.69	1.87E-73	0.984127	-4.938	2.33E-19	1
AQP7	364	-5.417	3.83E-55	0.972678	-6.3776	2.14E-94	0.993579	-5.685	1.03E-59	0.972222	-6.604	7.79E-23	1
ADH4	127	-3.708	1.47E-53	0.972678	-4.0228	9.74E-109	0.958266	-3.897	3.56E-65	0.956349	-4.303	1.44E-15	1
KLB	152831	-3.874	1.41E-49	0.972678	-4.2424	5.03E-98	0.983949	-4.24	4.02E-64	0.980159	-4.342	3.15E-13	1
PLIN1	5346	-5.378	1.29E-48	0.972678	-6.4845	5.91E-83	0.990369	-5.906	1.45E-56	0.97619	-6.97	1.54E-19	1
PLIN4	729359	-5.19	4.66E-48	0.972678	-6.0449	3.22E-81	0.975923	-5.605	9.07E-59	0.968254	-5.986	1.84E-18	1
FRMD1	79981	-3.852	7.40E-47	0.972678	-4.0517	1.77E-85	0.972713	-3.794	3.33E-52	0.960317	-4.493	2.30E-14	1
GYG2	8908	-3.38	2.27E-45	0.972678	-3.4548	3.31E-75	0.987159	-3.578	2.84E-53	0.984127	-3.511	2.75E-10	0.95
SLC7A10	56301	-4.757	5.32E-43	0.972678	-5.0817	1.71E-78	0.979133	-4.924	1.20E-48	0.956349	-5.257	1.70E-12	1
TUSC5	286753	-5.726	7.03E-43	0.972678	-7.0119	2.44E-71	0.980738	-6.393	9.87E-49	0.956349	-7.535	5.23E-20	1
PCK1	5105	-5.246	1.57E-40	0.972678	-6.1456	6.58E-75	0.987159	-5.575	1.97E-47	0.980159	-6.546	4.77E-15	1
ANGPTL5	253935	-3.006	1.16E-34	0.972678	-3.2123	5.73E-74	0.974318	-3.14	3.89E-46	0.972222	-3.503	3.77E-09	1
HBA1	3039	-5.089	5.14E-34	0.972678	-5.4079	1.18E-58	0.979133	-4.529	3.86E-32	0.956349	-5.145	3.20E-09	0.9
GLP2R	9340	-2.614	3.65E-32	0.972678	-2.7311	5.37E-81	0.975923	-2.812	1.54E-50	0.992063	-2.94	1.01E-07	1
HHATL	57467	-2.761	1.02E-29	0.972678	-2.9556	6.87E-78	0.991974	-2.939	3.21E-51	1	-3.153	9.02E-09	1

SDPR	8436	-4.43	2.30E-82	0.967213	-4.9075	3.26E-120	0.956661	-4.328	2.82E-79	0.920635	-5.086	1.43E-45	1
LRRC2	79442	-3.443	2.06E-63	0.967213	-3.576	5.06E-96	0.969502	-3.366	1.35E-70	0.956349	-3.754	4.61E-28	1
ATP1A2	477	-4.883	4.88E-61	0.967213	-5.691	1.23E-88	0.977528	-5.009	2.97E-65	0.988095	-6.363	2.58E-29	1
HEPN1	641654	-4.994	2.75E-58	0.967213	-5.5186	1.23E-106	0.977528	-5.255	7.88E-68	0.972222	-5.613	8.86E-20	1
AOC3	8639	-3.487	6.83E-57	0.967213	-3.7736	1.12E-104	0.967897	-3.397	2.82E-64	0.960317	-3.702	4.46E-16	1
ALDH1L1	10840	-5.129	9.01E-57	0.967213	-5.7279	2.48E-92	0.980738	-5.522	3.54E-65	0.992063	-5.446	1.16E-20	1
RDH5	5959	-3.933	3.85E-56	0.967213	-4.2588	3.66E-106	0.974318	-4.11	3.06E-62	0.952381	-4.346	2.61E-16	1
LIPE	3991	-3.87	3.88E-48	0.967213	-4.381	3.56E-87	0.975923	-4.064	5.60E-56	0.952381	-4.172	2.46E-13	1
C14orf180	400258	-5.232	9.87E-47	0.967213	-6.0396	9.58E-90	0.982343	-5.556	1.20E-55	0.952381	-5.603	7.68E-15	1
RBP4	5950	-5.15	2.85E-46	0.967213	-5.71	1.56E-80	0.977528	-5.534	2.27E-58	0.980159	-5.591	1.61E-15	1
CIDEC	63924	-5.648	6.12E-45	0.967213	-6.9027	9.83E-80	0.985554	-6.419	2.13E-52	0.97619	-7.349	1.35E-19	1
MRAP	56246	-5.481	8.08E-45	0.967213	-6.1325	3.12E-88	0.977528	-5.791	1.22E-54	0.968254	-6.319	1.95E-14	1
LOC55908	55908	-3.783	2.75E-43	0.967213	-3.9856	2.98E-90	0.966292	-3.853	2.74E-54	0.968254	-3.835	1.23E-09	0.95
LGALS12	85329	-4.552	8.88E-42	0.967213	-5.3928	1.91E-74	0.967897	-4.862	8.28E-48	0.956349	-5.24	4.03E-13	0.95
EPB42	2038	-3.67	5.68E-40	0.967213	-3.9887	1.43E-94	0.974318	-3.872	8.93E-54	0.980159	-4.065	9.14E-10	1
SLC22A12	116085	-2.738	3.54E-37	0.967213	-2.8312	4.63E-96	0.977528	-2.794	1.22E-47	0.97619	-3.048	4.56E-08	1
HSD17B13	345275	-4.755	1.13E-64	0.961749	-5.1744	5.70E-138	0.991974	-5.067	4.03E-81	0.992063	-5.041	1.64E-18	1
TNMD	64102	-5.048	8.38E-61	0.961749	-5.4655	2.37E-96	0.958266	-5.276	1.57E-71	0.968254	-5.606	9.21E-26	1
HSPB6	126393	-4.353	3.25E-60	0.961749	-4.9215	3.42E-111	0.983949	-4.408	3.14E-70	0.964286	-4.459	1.00E-18	0.95
SORBS1	10580	-3.155	5.59E-58	0.961749	-3.4112	1.39E-103	0.966292	-3.435	3.46E-69	0.964286	-3.844	3.51E-19	1
FAM180B	399888	-3.774	2.50E-50	0.961749	-3.8352	6.50E-80	0.945425	-3.662	8.54E-54	0.952381	-4.212	4.75E-15	1
CRHBP	1393	-3.369	2.93E-50	0.961749	-3.6223	3.63E-95	0.967897	-3.322	1.37E-57	0.988095	-3.949	3.21E-15	1
CES1	1066	-4.291	8.94E-47	0.961749	-4.3916	8.27E-73	0.948636	-4.142	6.82E-53	0.960317	-4.314	2.10E-17	1
GSTM5	2949	-3.932	3.08E-44	0.961749	-4.399	3.02E-81	0.967897	-3.882	5.30E-50	0.956349	-3.637	1.74E-15	0.95
BMP3	651	-3.704	1.63E-42	0.961749	-3.8706	4.83E-69	0.961477	-3.825	5.78E-49	0.956349	-3.615	2.18E-11	0.9
PRRT4	401399	-3.678	9.67E-42	0.961749	-3.6741	7.48E-64	0.934189	-3.331	6.51E-40	0.912698	-3.473	3.21E-10	1
TRHDE	29953	-3.883	7.99E-40	0.961749	-4.1265	2.62E-72	0.966292	-3.793	3.83E-45	0.952381	-4.333	1.26E-11	1
HPD	3242	-3.242	2.68E-31	0.961749	-3.2074	2.33E-58	0.969502	-3.174	7.78E-41	0.980159	-3.585	7.99E-09	0.95
HSD3B2	3284	-2.34	1.33E-13	0.961749	-2.4287	7.94E-28	0.967897	-2.497	1.21E-17	0.956349	-2.238	0.003077	0.95
LOC572558	572558	-3.661	3.92E-80	0.956284	-3.7853	1.46E-137	0.94382	-3.555	6.66E-83	0.912698	-3.881	1.41E-27	0.95
ANGPT4	51378	-3.377	7.42E-63	0.956284	-3.5026	1.81E-115	0.958266	-3.19	3.52E-58	0.90873	-3.843	4.83E-19	0.9
KCNJ16	3773	-4.851	7.40E-57	0.956284	-5.2393	1.64E-90	0.934189	-5.091	5.13E-68	0.928571	-6.086	7.44E-29	0.95
ACACB	32	-3.265	2.19E-56	0.956284	-3.6018	1.56E-96	0.955056	-3.251	3.06E-62	0.936508	-3.937	1.56E-19	1
CHRDL1	91851	-4.961	1.01E-54	0.956284	-5.5504	8.19E-81	0.975923	-5.232	2.54E-58	0.972222	-6.337	7.25E-29	1

TMEM132C	92293	-5.105	5.81E-53	0.956284	-6.1296	1.02E-89	0.980738	-5.459	1.20E-56	0.952381	-6.229	5.89E-25	0.95
C13orf36	400120	-3.151	4.25E-48	0.956284	-3.2053	1.97E-88	0.945425	-3.271	1.34E-62	0.960317	-3.232	4.54E-11	0.95
RXRG	6258	-3.222	8.84E-48	0.956284	-3.0712	4.42E-60	0.906902	-2.873	7.85E-46	0.920635	-2.292	4.06E-07	0.75
KLHL31	401265	-2.9	2.55E-46	0.956284	-3.2704	1.34E-94	0.977528	-3.417	2.18E-62	0.968254	-3.668	1.47E-15	1
PFKFB1	5207	-3.539	7.28E-43	0.956284	-3.7535	5.79E-72	0.951846	-3.46	2.33E-45	0.924603	-4.087	9.45E-13	0.95
ABCD2	225	-3.704	2.86E-42	0.956284	-3.8855	5.85E-76	0.967897	-3.982	7.02E-55	0.980159	-4.523	6.46E-15	1
CIDEA	1149	-5.292	1.76E-39	0.956284	-6.3158	5.58E-75	0.977528	-5.783	2.29E-47	0.944444	-6.618	2.69E-17	1
GPR144	347088	-4.016	1.76E-38	0.956284	-4.2483	1.37E-90	0.974318	-3.838	6.36E-38	0.928571	-4.117	5.39E-08	0.9
PDE3B	5140	-2.988	3.42E-32	0.956284	-3.2604	3.79E-56	0.959872	-3.252	9.70E-42	0.940476	-3.794	2.74E-10	1
LRRC3B	116135	-4.103	1.00E-72	0.95082	-4.3694	1.80E-133	0.948636	-4.191	3.38E-84	0.952381	-4.36	4.25E-28	0.95
BTNL9	153579	-4.09	3.26E-70	0.95082	-4.5373	6.58E-112	0.971108	-4.086	6.63E-72	0.940476	-4.646	2.87E-29	1
ITIH5	80760	-3.992	6.46E-70	0.95082	-4.3841	3.19E-115	0.966292	-4.053	1.80E-80	0.956349	-4.469	1.01E-29	1
MESTIT1	317751	-3.616	2.48E-68	0.95082	-3.8025	1.86E-121	0.961477	-3.652	8.21E-83	0.964286	-4.065	9.62E-26	1
NAALAD2	10003	-3.106	1.06E-61	0.95082	-3.2402	9.72E-116	0.958266	-3.157	8.66E-77	0.948413	-3.067	4.15E-16	0.85
ACSM5	54988	-3.436	1.95E-49	0.95082	-3.7916	8.53E-87	0.969502	-3.412	4.04E-59	0.952381	-4.178	1.04E-16	1
PCOLCE2	26577	-4.834	4.67E-44	0.95082	-5.2427	1.50E-70	0.969502	-5.303	2.41E-56	0.984127	-5.463	8.36E-17	0.95
CASQ2	845	-3.171	2.86E-42	0.95082	-3.5986	9.21E-65	0.924559	-3.005	4.64E-42	0.884921	-4.161	9.39E-21	0.9
G0S2	50486	-3.801	8.13E-41	0.95082	-4.214	7.55E-78	0.961477	-4.109	3.36E-50	0.936508	-4.092	5.25E-13	1
ALB	213	-3.88	2.53E-21	0.95082	-4.1372	8.12E-33	0.9374	-3.932	6.06E-23	0.93254	-3.187	0.000842	0.9
MME	4311	-4.099	3.12E-69	0.945355	-4.3227	4.77E-95	0.939005	-4.094	7.03E-74	0.940476	-4.429	1.61E-37	0.95
NPR1	4881	-3.376	6.09E-67	0.945355	-3.6208	2.45E-117	0.964687	-3.326	1.33E-70	0.936508	-3.259	1.19E-18	1
TRPM3	80036	-3.035	1.21E-62	0.945355	-3.0463	4.91E-118	0.918138	-2.897	1.65E-63	0.880952	-2.811	6.04E-16	0.95
ANGPTL7	10218	-5.414	5.27E-60	0.945355	-5.8529	3.13E-115	0.953451	-5.575	1.22E-73	0.952381	-6.305	1.94E-26	1
SCN4A	6329	-3.403	5.02E-57	0.945355	-3.8356	2.88E-87	0.966292	-3.419	1.11E-55	0.920635	-3.889	4.39E-21	0.95
PCDH9	5101	-3.551	1.07E-55	0.945355	-3.633	6.43E-68	0.924559	-3.801	1.13E-57	0.944444	-3.803	1.07E-17	0.95
ITGA7	3679	-3.34	6.23E-55	0.945355	-3.6193	6.23E-92	0.942215	-3.339	3.48E-56	0.900794	-3.68	3.67E-17	1
PAPPA2	60676	-3.675	4.75E-53	0.945355	-3.812	1.36E-93	0.950241	-3.682	3.64E-62	0.944444	-3.944	9.10E-17	0.95
KLHL33	123103	-2.502	2.22E-51	0.945355	-2.6146	1.34E-99	0.963082	-2.433	3.48E-54	0.916667	-2.695	6.97E-15	0.95
FABP4	2167	-5.453	1.92E-45	0.945355	-6.5052	1.08E-74	0.985554	-5.917	1.35E-51	0.968254	-6.423	9.96E-23	1
KCNA4	3739	-2.554	3.08E-40	0.945355	-2.5453	8.55E-70	0.94703	-2.57	2.59E-51	0.964286	-2.44	1.24E-07	0.95
PGA3	643834	-3.129	1.20E-36	0.945355	-3.2533	5.42E-71	0.959872	-3.219	7.22E-44	0.948413	-3.54	3.20E-10	0.95
SEL1L2	80343	-2.469	3.51E-36	0.945355	-2.5107	3.44E-80	0.963082	-2.479	9.01E-46	0.972222	-2.717	1.94E-08	1
GABRA4	2557	-2.336	7.11E-34	0.945355	-2.3605	5.80E-59	0.934189	-2.437	2.98E-45	0.936508	-2.562	7.48E-08	0.95
PENK	5179	-3.794	1.49E-33	0.945355	-4.2141	3.43E-69	0.975923	-3.795	1.55E-43	0.964286	-4.538	2.30E-13	1

CYP1A1	1543	-2.164	8.14E-12	0.945355	-2.3529	5.98E-31	0.971108	-2.168	8.46E-15	0.952381	-2.513	0.001196	1
CSN3	1448	-2.56	1.46E-11	0.945355	-2.8231	1.64E-25	0.967897	-2.841	2.73E-16	0.968254	-2.742	0.002854	0.95
ADAMTS5	11096	-3.541	2.29E-80	0.939891	-3.5519	2.30E-118	0.919743	-3.527	6.01E-81	0.892857	-3.647	6.34E-37	0.9
CLEC3B	7123	-3.428	6.29E-59	0.939891	-3.8036	5.47E-103	0.956661	-3.451	8.39E-67	0.944444	-3.5	5.55E-17	1
SVEP1	79987	-3.408	3.17E-58	0.939891	-3.6778	2.76E-82	0.914928	-3.131	3.25E-54	0.876984	-3.674	1.27E-19	0.95
MAP1LC3C	440738	-3.664	6.78E-55	0.939891	-4.1603	1.17E-100	0.958266	-3.867	3.73E-65	0.93254	-4.472	1.74E-20	0.85
PKHD1L1	93035	-4.009	2.65E-46	0.939891	-4.1699	3.55E-81	0.950241	-4.066	6.90E-54	0.93254	-3.912	1.37E-11	0.85
NPY2R	4887	-5.436	1.39E-44	0.939891	-6.1143	1.10E-95	0.951846	-5.832	4.99E-59	0.952381	-5.819	2.28E-14	0.9
FAM196B	1E+08	-4.296	5.29E-44	0.939891	-4.552	6.13E-79	0.953451	-4.346	9.72E-52	0.964286	-4.691	2.44E-13	0.95
CDH20	28316	-2.876	1.75E-40	0.939891	-3.0373	1.05E-70	0.930979	-3.241	2.10E-57	0.968254	-3.18	1.81E-10	0.95
MYBPC1	4604	-3.361	6.64E-12	0.939891	-3.9914	1.55E-21	0.934189	-4.687	3.34E-24	0.944444	-5.396	5.46E-07	0.9
TNS1	7145	-2.736	3.93E-67	0.934426	-2.9229	6.03E-124	0.94061	-2.73	4.49E-77	0.93254	-2.993	6.42E-20	0.9
B3GALT1	8708	-3.444	3.18E-61	0.934426	-3.5457	1.29E-99	0.9374	-3.487	1.41E-64	0.912698	-3.443	6.46E-16	0.9
MASP1	5648	-4.432	7.71E-60	0.934426	-4.6159	1.73E-99	0.94382	-4.456	4.37E-70	0.936508	-5.54	3.97E-30	1
ABCA9	10350	-4.169	1.71E-58	0.934426	-4.6705	5.22E-99	0.955056	-4.015	2.37E-61	0.916667	-5.076	3.06E-28	0.95
PDK4	5166	-3.684	2.98E-58	0.934426	-4.0935	5.78E-86	0.94061	-3.721	6.13E-57	0.904762	-4.347	3.17E-25	1
GDF10	2662	-4.131	2.05E-56	0.934426	-4.6728	4.20E-85	0.935795	-4.277	5.04E-60	0.90873	-4.548	1.85E-22	0.9
CDO1	1036	-3.623	3.02E-55	0.934426	-4.1813	2.36E-83	0.927769	-3.838	4.25E-60	0.928571	-4.314	1.91E-21	0.95
CD36	948	-4.573	3.04E-53	0.934426	-4.9711	1.23E-89	0.948636	-4.552	3.25E-64	0.960317	-4.806	1.98E-22	1
GPX3	2878	-3.356	1.39E-48	0.934426	-3.5685	4.25E-90	0.950241	-3.328	4.54E-53	0.904762	-3.501	1.29E-13	1
SPHKAP	80309	-4.534	2.46E-43	0.934426	-4.6017	7.08E-65	0.911717	-4.942	4.23E-63	0.972222	-5.236	6.93E-14	0.95
SAA2	6289	-4.464	1.43E-41	0.934426	-4.9282	5.79E-57	0.930979	-5.138	1.47E-49	0.928571	-4.968	3.18E-17	1
ALDH3A1	218	-3.557	1.67E-38	0.934426	-3.5437	5.43E-47	0.910112	-3.659	2.82E-44	0.924603	-4.12	3.47E-16	0.95
CLDN19	149461	-4.138	2.66E-37	0.934426	-4.8161	2.34E-69	0.922953	-4.387	8.47E-43	0.924603	-5.032	5.80E-13	0.8
SGCG	6445	-3.824	1.43E-36	0.934426	-4.1586	6.27E-66	0.94061	-3.744	1.19E-38	0.90873	-4.66	3.71E-14	1
PROKR1	10887	-2.364	5.02E-30	0.934426	-2.4193	1.04E-55	0.930979	-2.441	4.70E-39	0.936508	-2.646	3.16E-08	1
CSF3	1440	-3.176	7.76E-28	0.934426	-3.2231	1.24E-56	0.951846	-3.209	7.85E-34	0.956349	-3.728	3.54E-08	1
DMD	1756	-3.672	1.02E-73	0.928962	-3.7947	2.83E-98	0.890851	-3.89	8.12E-85	0.916667	-4.147	5.54E-38	0.9
FXYD1	5348	-4.712	2.96E-65	0.928962	-5.3153	8.48E-98	0.942215	-4.706	2.28E-63	0.904762	-5.268	1.39E-37	1
LOC728264	728264	-3.712	1.57E-64	0.928962	-3.934	1.17E-105	0.926164	-3.597	4.59E-64	0.892857	-3.92	7.02E-22	0.9
TNXB	7148	-4.124	8.86E-64	0.928962	-4.6746	1.46E-115	0.969502	-4.244	8.17E-70	0.948413	-4.888	4.29E-30	0.95
KLF15	28999	-3.5	3.75E-61	0.928962	-3.6691	9.12E-83	0.930979	-3.638	1.75E-57	0.900794	-3.811	1.07E-22	1
ASPA	443	-3.504	1.99E-58	0.928962	-3.8764	8.96E-95	0.94703	-3.727	3.07E-68	0.952381	-3.687	2.59E-20	0.95
ABCB5	340273	-4.304	3.17E-57	0.928962	-4.8194	1.51E-109	0.964687	-4.147	1.13E-57	0.880952	-4.516	4.07E-18	0.85

ACADL	33	-4.582	2.02E-52	0.928962	-4.9956	1.65E-77	0.934189	-4.948	1.30E-62	0.924603	-5.346	2.13E-23	0.95
ABCA8	10351	-4.634	2.93E-52	0.928962	-5.4435	2.35E-76	0.950241	-4.742	4.99E-57	0.940476	-6.157	6.25E-31	0.95
GPR109A	338442	-3.038	8.58E-50	0.928962	-3.2457	3.27E-83	0.921348	-3.178	3.23E-56	0.90873	-3.845	4.61E-19	1
KY	339855	-3.954	3.69E-47	0.928962	-4.3319	8.68E-78	0.926164	-4.211	1.08E-53	0.916667	-4.543	6.43E-15	0.85
PLXNA4	91584	-2.999	3.04E-44	0.928962	-3.2428	3.84E-66	0.900482	-3.292	1.94E-48	0.928571	-3.538	8.81E-14	0.95
SGK2	10110	-3.298	6.93E-41	0.928962	-3.7538	5.46E-73	0.94382	-3.694	3.53E-54	0.948413	-3.876	2.30E-14	1
SLC16A7	9194	-3.097	5.82E-39	0.928962	-3.2961	2.09E-73	0.9374	-3.204	1.11E-52	0.948413	-3.668	5.21E-14	0.95
OVCH2	341277	-3.089	2.60E-35	0.928962	-3.3623	4.41E-78	0.922953	-3.054	1.80E-38	0.904762	-3.417	4.23E-09	0.95
DLK1	8788	-4.565	4.89E-30	0.928962	-4.9063	4.82E-47	0.935795	-4.792	5.42E-35	0.93254	-4.722	4.23E-09	0.9
LOC93432	93432	-3	1.87E-27	0.928962	-2.8318	2.71E-38	0.897271	-3.231	7.01E-45	0.952381	-2.896	5.03E-06	0.9
KIAA1239	57495	-2.994	3.12E-27	0.928962	-3.4017	1.63E-56	0.94061	-3.269	1.47E-38	0.936508	-3.848	2.03E-10	1
UGT2B28	54490	-2.048	2.43E-06	0.928962	-2.6089	5.94E-17	0.948636	-2.347	1.17E-09	0.93254	NA	NA	NA
HIF3A	64344	-5.007	3.12E-69	0.923497	-5.2192	1.12E-105	0.94382	-5.167	7.10E-75	0.936508	-5.175	5.27E-33	0.95
EBF1	1879	-2.904	2.29E-63	0.923497	-3.1337	2.25E-108	0.919743	-2.894	6.40E-69	0.900794	-3.048	5.73E-19	0.9
GPIHBP1	338328	-3.789	4.36E-62	0.923497	-4.3009	7.84E-96	0.934189	-3.731	2.54E-62	0.892857	-3.644	4.40E-24	0.95
FMO2	2327	-4.127	6.24E-60	0.923497	-4.5431	3.48E-81	0.908507	-4.203	3.02E-62	0.884921	-4.826	2.57E-32	0.95
IGSF10	285313	-3.803	5.13E-56	0.923497	-4.0989	3.76E-84	0.913323	-3.944	5.88E-68	0.90873	-4.526	5.14E-27	0.85
STAB2	55576	-4.107	2.50E-52	0.923497	-4.4759	1.08E-95	0.935795	-4.006	1.25E-53	0.896825	-4.76	2.83E-19	0.85
GCOM1	145781	-3.257	2.74E-51	0.923497	-3.3518	2.63E-77	0.916533	-3.12	1.24E-58	0.90873	-3.156	6.92E-17	0.8
NMUR1	10316	-3.241	5.86E-49	0.923497	-3.266	2.31E-77	0.914928	-3.355	1.12E-57	0.940476	-3.468	3.39E-14	1
NNAT	4826	-3.374	3.82E-45	0.923497	-3.9508	2.00E-75	0.935795	-3.779	1.05E-50	0.924603	-4.091	6.17E-16	1
TIMP4	7079	-4.516	3.26E-44	0.923497	-5.173	1.66E-77	0.969502	-4.725	2.35E-52	0.968254	-4.842	2.41E-15	1
CLCA4	22802	-4.166	1.06E-42	0.923497	-4.3392	1.44E-70	0.916533	-4.406	3.90E-52	0.912698	-4.849	3.69E-13	0.95
FGFBP2	83888	-3.804	2.20E-39	0.923497	-3.9386	9.18E-62	0.930979	-3.981	2.09E-51	0.944444	-4.049	6.60E-13	0.9
KLHL30	377007	-2.702	2.47E-34	0.923497	-2.4689	9.53E-43	0.88764	-2.339	1.61E-32	0.884921	NA	NA	NA
AADAC	13	-3.668	3.69E-28	0.923497	-3.9608	4.04E-65	0.971108	-3.953	4.58E-42	0.980159	-3.875	5.78E-07	0.95
ITIH2	3698	-3.356	2.51E-23	0.923497	-3.6636	2.27E-46	0.9374	-3.615	5.81E-31	0.936508	-4.026	1.85E-07	0.95
CAV1	857	-3.247	4.15E-76	0.918033	-3.4577	6.13E-133	0.94382	-3.218	1.18E-84	0.944444	-3.612	1.36E-27	1
RYR3	6263	-3.593	6.59E-75	0.918033	-3.7182	2.46E-103	0.905297	-3.692	1.13E-79	0.912698	-4.264	3.36E-34	0.95
ABCA10	10349	-4.369	5.56E-69	0.918033	-4.788	3.53E-112	0.945425	-4.478	6.71E-70	0.912698	-5.652	2.98E-37	0.95
HPSE2	60495	-4.482	6.53E-65	0.918033	-4.7232	2.02E-110	0.939005	-4.559	2.65E-70	0.920635	-5.16	3.68E-28	0.95
DPP6	1804	-4.232	2.86E-59	0.918033	-4.3911	1.70E-104	0.945425	-4.409	6.36E-81	0.936508	-3.922	1.43E-23	0.9
AKR1C1	1645	-4.219	5.39E-49	0.918033	-4.5705	1.21E-72	0.932584	-4.256	1.11E-55	0.920635	-4.805	1.74E-20	1
PPP1R1A	5502	-4.259	5.61E-45	0.918033	-4.8481	9.05E-64	0.935795	-4.832	1.66E-50	0.936508	-5.095	1.57E-19	0.95

DCT	1638	-3.127	2.27E-44	0.918033	-3.3361	1.93E-90	0.959872	-3.334	4.01E-59	0.944444	-3.318	1.78E-13	0.85
SAA1	6288	-4.254	1.24E-43	0.918033	-4.8639	6.68E-60	0.910112	-4.934	3.79E-48	0.920635	-4.831	1.46E-20	1
CLEC4G	339390	-3.222	8.88E-42	0.918033	-3.4256	1.33E-79	0.930979	-3.295	2.16E-50	0.944444	-3.141	5.20E-10	0.9
DPT	1805	-3.388	1.04E-41	0.918033	-3.5924	1.26E-66	0.9374	-3.275	1.21E-47	0.920635	-3.085	1.36E-11	0.85
MMD	23531	-2.175	7.19E-35	0.918033	-2.1847	1.22E-67	0.894061	-2.191	4.93E-40	0.888889	-2.129	1.27E-06	0.95
SLCO1A2	6579	-3.626	1.45E-27	0.918033	-3.8781	2.23E-56	0.9374	-3.985	3.61E-42	0.940476	-3.699	3.28E-07	0.85
IGFBP1	3484	-2.614	2.53E-21	0.918033	-2.814	3.07E-46	0.932584	-2.937	1.12E-35	0.956349	-3.248	6.79E-07	0.95
PROL1	58503	-4.137	1.56E-20	0.918033	-4.9772	2.59E-45	0.934189	-4.502	1.63E-26	0.912698	-6.419	3.09E-11	1
NTS	4922	-3.015	3.10E-17	0.918033	-3.0653	1.00E-23	0.906902	-3.093	1.08E-24	0.90873	-3.626	4.57E-07	0.95
SLC17A7	57030	-3.306	6.09E-67	0.912568	-3.447	2.56E-97	0.913323	-3.142	1.89E-67	0.876984	-3.315	9.18E-23	0.9
MYH11	4629	-3.93	9.14E-61	0.912568	-4.4093	8.09E-83	0.913323	-3.846	3.86E-59	0.873016	-4.64	1.75E-30	0.9
ANGPTL1	9068	-3.688	2.46E-56	0.912568	-3.9571	5.15E-91	0.922953	-3.429	2.49E-61	0.865079	-4.416	4.76E-26	0.9
TMEM100	55273	-3.655	3.43E-49	0.912568	-4.1688	5.86E-77	0.930979	-3.853	6.40E-57	0.924603	-4.486	3.42E-24	0.95
GRIA4	2893	-4.518	4.00E-41	0.912568	-5.0126	7.85E-71	0.930979	-4.597	3.85E-43	0.90873	-5.189	1.42E-13	0.9
PKD1L2	114780	-3.047	3.14E-39	0.912568	-3.0958	5.82E-55	0.890851	-3.286	2.18E-51	0.948413	-2.899	4.99E-10	0.9
BHMT2	23743	-2.837	1.10E-37	0.912568	-3.1244	5.05E-63	0.922953	-2.837	1.71E-42	0.880952	-3.176	1.57E-11	0.9
MRGPRX2	117194	-2.174	2.85E-37	0.912568	-2.3002	7.96E-81	0.924559	-2.179	7.98E-42	0.892857	-2.259	7.46E-08	0.9
DGAT2	84649	-3.068	6.32E-36	0.912568	-2.9766	1.41E-52	0.911717	-3.12	1.47E-41	0.93254	-3.186	2.81E-09	1
PROK1	84432	-2.151	9.03E-21	0.912568	-2.1466	5.08E-28	0.910112	NA	NA	NA	NA	NA	NA
PRG4	10216	-2.126	1.41E-10	0.912568	-2.8166	1.87E-23	0.924559	-2.47	1.93E-14	0.924603	-3.47	2.45E-05	1
PAMR1	25891	-3.856	8.00E-76	0.907104	-4.2634	2.94E-125	0.935795	-3.899	1.55E-84	0.928571	-4.055	5.68E-32	0.85
ATOH8	84913	-3.691	7.47E-64	0.907104	-3.9883	2.26E-107	0.926164	-3.65	7.85E-64	0.865079	-3.68	2.56E-26	0.9
KCNB1	3745	-3.986	1.88E-54	0.907104	-4.1104	9.00E-78	0.916533	-4.015	3.06E-57	0.892857	-4.254	1.23E-24	0.95
PPARG	5468	-3.07	6.73E-51	0.907104	-3.3113	1.94E-98	0.942215	-3.127	3.71E-64	0.924603	-3.436	2.14E-17	1
GHR	2690	-3.06	1.50E-49	0.907104	-3.3069	1.26E-74	0.900482	-2.979	2.36E-57	0.892857	-3.536	5.37E-20	1
CNN1	1264	-3.279	1.46E-48	0.907104	-3.6571	1.72E-81	0.911717	-3.376	1.37E-56	0.900794	-3.743	8.03E-17	0.9
CLEC4GP1	440508	-2.977	1.20E-47	0.907104	-2.9843	2.61E-86	0.929374	-2.831	2.65E-50	0.888889	-2.82	9.28E-12	0.85
ANGPT1	284	-3.074	1.34E-46	0.907104	-2.9863	2.23E-74	0.88443	-3.083	1.73E-58	0.912698	-3.045	3.86E-14	0.9
CYP3A4	1576	-2.191	3.46E-41	0.907104	-2.1778	1.06E-76	0.892456	-2.231	1.01E-49	0.912698	-2.286	4.03E-10	0.9
P2RX6	9127	-2.659	3.51E-41	0.907104	-2.4864	1.44E-50	0.869984	-2.584	1.05E-42	0.869048	-2.41	4.10E-09	0.95
RNF186	54546	-3.498	2.66E-40	0.907104	-3.7907	2.10E-74	0.914928	-3.604	1.31E-47	0.904762	-3.629	1.37E-10	0.9
SLC7A3	84889	-3.725	7.53E-32	0.907104	-4.1809	1.47E-60	0.906902	-3.704	8.28E-32	0.853175	-4.686	3.80E-11	0.9
DSC1	1823	-3.084	9.80E-31	0.907104	-3.1761	1.32E-49	0.892456	-3.042	7.47E-35	0.876984	-3.032	1.14E-06	0.9
RPE65	6121	-2.059	1.06E-27	0.907104	-2.1043	1.06E-50	0.916533	-2.129	2.53E-36	0.928571	-2.292	9.54E-07	0.9

AQP4	361	-2.407	1.21E-22	0.907104	-2.8144	1.20E-42	0.927769	-3.147	2.73E-38	0.944444	-3.203	4.08E-11	0.9
TSLP	85480	-3.929	1.06E-70	0.901639	-4.2692	9.77E-99	0.905297	-4.151	1.32E-71	0.888889	-4.516	1.54E-40	0.85
IGFBP6	3489	-3.064	1.40E-60	0.901639	-3.4227	3.84E-110	0.945425	-3.118	7.47E-65	0.896825	-3.039	1.55E-18	0.95
NIPSNAP3B	55335	-2.42	1.13E-55	0.901639	-2.7377	1.51E-97	0.918138	-2.763	1.68E-65	0.924603	-3.029	2.43E-18	0.95
CXCL2	2920	-4.228	5.83E-55	0.901639	-4.6335	3.79E-89	0.927769	-4.799	1.49E-65	0.880952	-5.496	8.67E-37	1
LOC90586	90586	-2.531	2.20E-50	0.901639	-2.5761	1.08E-89	0.898876	-2.362	5.90E-48	0.853175	-2.638	9.55E-13	0.9
CCDC3	83643	-2.553	1.48E-49	0.901639	-2.6133	8.68E-75	0.886035	-2.537	1.74E-52	0.873016	-2.703	1.79E-12	0.85
TF	7018	-4.153	5.25E-45	0.901639	-4.6536	1.88E-51	0.863563	-4.891	4.21E-55	0.90873	-4.461	4.20E-20	0.8
KCNE1	3753	-3.698	9.58E-45	0.901639	-4.1235	1.66E-75	0.913323	-3.864	1.60E-49	0.869048	-4.479	5.80E-17	0.85
AKR1C2	1646	-4.367	1.29E-43	0.901639	-4.8268	1.36E-60	0.924559	-4.432	8.87E-50	0.928571	-4.894	3.86E-18	1
DMGDH	29958	-2.738	1.47E-42	0.901639	-2.9799	1.36E-75	0.898876	-2.858	2.96E-53	0.896825	-3.124	1.32E-13	0.85
PI16	221476	-4.67	4.46E-40	0.901639	-5.6245	3.72E-65	0.927769	-5.082	8.18E-43	0.904762	-6.232	7.98E-23	1
C2orf84	653140	-2.253	3.92E-38	0.901639	-2.4194	4.97E-68	0.900482	-2.441	1.40E-46	0.896825	-2.111	1.51E-07	0.9
C4BPA	722	-3.769	1.77E-29	0.901639	-3.9881	7.94E-45	0.879615	-3.999	4.09E-38	0.916667	-4.605	8.12E-11	0.85
LOC10012469	1E+08	-3.629	4.43E-28	0.901639	-3.5529	1.68E-37	0.882825	-4.09	6.02E-42	0.936508	-3.812	1.94E-07	0.85
PDE2A	5138	-3.199	8.00E-76	0.896175	-3.5541	3.86E-117	0.918138	-3.171	7.71E-75	0.849206	-3.435	1.21E-29	0.9
ARHGAP20	57569	-3.29	2.71E-68	0.896175	-3.628	5.91E-108	0.924559	-3.309	3.81E-76	0.896825	-3.849	4.69E-30	0.95
RERGL	79785	-4.248	6.91E-54	0.896175	-4.9676	8.25E-79	0.902087	-4.353	8.18E-50	0.825397	-5.62	6.82E-36	0.9
VIT	5212	-4.059	5.19E-53	0.896175	-4.6273	1.56E-78	0.918138	-4.378	3.57E-64	0.896825	-5.084	1.70E-31	0.85
ADH1C	126	-4.586	2.00E-52	0.896175	-5.4064	1.97E-83	0.924559	-4.636	8.74E-59	0.900794	-5.467	1.59E-29	0.9
ADCYAP1R1	117	-3.55	9.93E-52	0.896175	-3.7608	1.02E-82	0.906902	-3.618	4.89E-59	0.904762	-3.851	1.88E-16	0.85
C7orf58	79974	-2.835	4.02E-44	0.896175	-3.092	9.38E-75	0.911717	-2.805	3.10E-49	0.892857	-3.38	2.26E-16	0.95
TMC2	117532	-2.493	5.07E-42	0.896175	-2.5564	9.34E-74	0.88443	-2.522	1.16E-48	0.892857	-2.813	4.75E-12	0.95
HS3ST4	9951	-4.183	9.58E-38	0.896175	-4.6485	5.71E-69	0.916533	-4.587	2.04E-52	0.944444	-4.963	3.23E-13	0.95
KBTBD12	166348	-2.163	1.73E-19	0.896175	-2.0711	2.27E-21	0.873194	-2.507	4.66E-34	0.936508	-2.499	6.46E-12	0.9
CA6	765	-2.836	7.09E-18	0.896175	-2.8076	4.52E-29	0.905297	-3.195	4.61E-34	0.928571	-2.907	3.81E-05	0.9
COL25A1	84570	-3.181	5.03E-63	0.89071	-3.3481	1.24E-83	0.897271	-3.409	1.15E-63	0.884921	-3.926	1.32E-26	0.9
C10orf90	118611	-4.315	2.02E-55	0.89071	-4.4047	2.28E-71	0.87801	-4.628	1.30E-70	0.924603	-4.904	1.98E-25	0.9
C2orf40	84417	-4.695	1.43E-49	0.89071	-5.4507	3.01E-79	0.905297	-4.888	8.31E-57	0.876984	-5.958	3.32E-34	0.9
SAMD5	389432	-3.322	2.19E-48	0.89071	-3.645	1.01E-76	0.900482	-3.431	5.73E-51	0.869048	-3.771	4.83E-14	0.9
NMNAT2	23057	-3.132	9.62E-47	0.89071	-2.9148	1.96E-50	0.826645	-2.96	8.47E-44	0.813492	-2.846	3.13E-14	0.8
GPLD1	2822	-3.132	1.22E-44	0.89071	-3.0858	1.50E-63	0.88122	-3.199	1.87E-51	0.900794	-3.84	3.70E-18	1
CFD	1675	-3.529	4.96E-44	0.89071	-3.7455	8.14E-63	0.879615	-3.21	2.07E-41	0.845238	-3.55	5.52E-15	0.95
FOSB	2354	-3.802	1.86E-40	0.89071	-4.3154	2.75E-70	0.922953	-4.265	6.27E-52	0.900794	-5.65	1.18E-23	1

MLXIPL	51085	-3.561	3.80E-38	0.89071	-3.9294	9.46E-68	0.939005	-3.687	5.63E-47	0.916667	-3.416	1.02E-11	0.9
THRSP	7069	-3.766	9.36E-36	0.89071	-4.2368	9.48E-43	0.866774	-3.821	3.02E-37	0.857143	-4.46	2.01E-17	0.95
FAM150B	285016	-3.598	1.32E-35	0.89071	-3.8862	2.55E-56	0.894061	-4.019	2.66E-54	0.904762	-4.133	9.59E-15	0.8
PAK7	57144	-3.508	6.00E-32	0.89071	-3.9512	2.12E-59	0.905297	-3.686	1.02E-34	0.884921	-4.869	6.42E-12	0.9
PTPRQ	374462	-3.63	8.14E-31	0.89071	-3.6833	2.47E-44	0.894061	-3.558	1.62E-34	0.892857	-3.488	2.20E-08	0.9
WIF1	11197	-4.982	8.59E-31	0.89071	-5.3769	4.89E-52	0.913323	-5.249	1.42E-39	0.920635	-4.751	3.20E-08	0.75
CALB2	794	-3.054	1.16E-29	0.89071	-2.8676	1.73E-29	0.820225	-3.041	4.36E-29	0.84127	-2.483	9.38E-06	0.75
LOC284578	284578	-3.68	1.38E-54	0.885246	-3.6331	9.18E-57	0.845907	-4.018	4.37E-68	0.896825	-4.04	4.11E-26	0.9
CHL1	10752	-3.856	4.30E-53	0.885246	-4.2207	7.29E-75	0.898876	-4.034	2.82E-64	0.916667	-5.05	1.56E-28	0.9
S100B	6285	-4.103	3.80E-47	0.885246	-4.5345	2.02E-64	0.87801	-4.774	3.57E-68	0.944444	-4.646	9.21E-26	0.95
CES4	51716	-3.437	6.43E-40	0.885246	-3.5507	3.44E-63	0.905297	-3.309	2.58E-44	0.888889	-3.55	1.88E-12	0.95
KIT	3815	-3.294	2.64E-39	0.885246	-3.6218	3.39E-63	0.879615	-3.624	2.54E-48	0.90873	-4.502	1.48E-17	0.95
GPBAR1	151306	-2.391	3.53E-36	0.885246	-2.4799	1.07E-72	0.910112	-2.278	2.21E-37	0.845238	-2.341	5.32E-08	0.95
EDN3	1908	-4.218	2.44E-29	0.885246	-4.7107	5.02E-49	0.88122	-4.477	3.00E-32	0.869048	-4.757	5.12E-09	0.85
FAM3D	131177	-3.475	7.52E-27	0.885246	-3.4762	1.05E-33	0.858748	-3.539	1.41E-29	0.884921	-3.268	5.24E-06	0.75
GSTA1	2938	-3.788	5.05E-24	0.885246	-3.5656	9.26E-26	0.815409	-3.797	3.20E-28	0.861111	-3.364	9.42E-06	0.8
PIGR	5284	-3.873	6.46E-19	0.885246	-4.5084	4.09E-32	0.895666	-4.001	1.88E-20	0.888889	-3.463	0.000577	0.9
ARHGAP36	158763	-3.416	5.00E-15	0.885246	-3.7603	8.15E-28	0.902087	-3.669	3.82E-22	0.920635	-3.759	9.84E-05	0.85
MAMDC2	256691	-3.668	1.60E-65	0.879781	-4.1221	9.58E-87	0.900482	-3.733	3.45E-70	0.873016	-4.56	2.36E-40	0.9
GPR146	115330	-2.632	4.39E-65	0.879781	-2.813	1.03E-121	0.900482	-2.585	4.59E-66	0.813492	-2.685	2.90E-18	0.9
CAPN11	11131	-3.311	1.15E-63	0.879781	-3.471	1.76E-91	0.869984	-3.053	7.16E-53	0.809524	-3.283	2.42E-22	0.9
SYNM	23336	-4.064	1.82E-63	0.879781	-4.2264	3.32E-90	0.88443	-4.308	3.45E-72	0.900794	-4.798	1.99E-32	0.95
C2orf88	84281	-3.081	9.26E-60	0.879781	-3.1235	1.51E-76	0.834671	-3.217	3.05E-72	0.865079	-3.574	4.30E-26	0.75
CRYAB	1410	-3.899	9.59E-58	0.879781	-4.0406	1.87E-66	0.865169	-4.108	5.03E-65	0.896825	-4.126	1.43E-26	0.9
C18orf34	374864	-3.343	4.67E-57	0.879781	-3.8991	8.19E-97	0.905297	-3.478	9.30E-58	0.845238	-4.059	9.64E-30	0.9
SCN3A	6328	-3.604	6.92E-57	0.879781	-4.1215	4.41E-86	0.906902	-3.826	2.52E-61	0.884921	-4.603	1.09E-27	0.85
LRRN4CL	221091	-3.438	1.24E-54	0.879781	-3.9971	1.11E-92	0.927769	-3.451	1.66E-60	0.857143	-4.285	4.52E-26	0.9
SLC6A2	6530	-3.555	6.33E-49	0.879781	-3.5254	1.23E-49	0.82504	-3.774	4.23E-57	0.865079	-3.834	3.00E-22	0.85
COL6A6	131873	-3.845	3.50E-46	0.879781	-4.4438	6.66E-85	0.935795	-4.182	1.22E-55	0.912698	-4.814	1.00E-21	0.85
PDE1C	5137	-3.633	1.51E-45	0.879781	-3.9219	3.49E-76	0.889246	-3.963	5.28E-58	0.900794	-4.324	1.79E-18	0.9
MAOA	4128	-3.496	6.18E-44	0.879781	-4.0685	2.89E-71	0.918138	-3.832	1.12E-58	0.888889	-4.582	1.98E-24	1
PAK3	5063	-3.461	1.58E-41	0.879781	-3.4398	3.34E-63	0.849117	-3.655	3.83E-61	0.912698	-4.078	2.45E-17	0.85
MMRN1	22915	-3.65	1.88E-40	0.879781	-4.4078	3.55E-73	0.921348	-3.59	2.51E-42	0.880952	-4.213	7.85E-18	0.9
PIK3C2G	5288	-3.861	7.15E-28	0.879781	-4.0829	1.86E-42	0.858748	-3.757	1.35E-28	0.809524	-4.105	2.74E-08	0.85

SLC14A2	8170	-2.62	6.59E-23	0.879781	-3.0366	8.96E-44	0.894061	-2.946	8.72E-35	0.90873	-3.75	1.87E-11	1
SMR3B	10879	-2.075	3.27E-09	0.879781	-2.5462	1.76E-21	0.919743	-2.189	1.27E-11	0.892857	-3.437	3.30E-06	1
LEPR	3953	-2.996	2.79E-61	0.874317	-3.3113	6.95E-107	0.911717	-3.048	2.18E-65	0.853175	-3.364	1.90E-23	0.9
DACT2	168002	-4.037	1.74E-58	0.874317	-4.3019	5.45E-80	0.890851	-4.137	3.86E-59	0.876984	-5.203	5.10E-31	0.95
ANKRD53	79998	-2.801	4.43E-54	0.874317	-3.0042	1.61E-81	0.874799	-2.893	5.15E-56	0.833333	-2.856	8.57E-16	0.9
SLC7A14	57709	-2.737	6.51E-52	0.874317	-2.851	1.51E-95	0.900482	-2.782	8.86E-57	0.900794	-2.765	7.91E-13	0.85
HAS3	3038	-3.403	1.67E-48	0.874317	-3.4117	3.32E-77	0.88443	-3.54	7.43E-64	0.928571	-4.124	5.04E-19	0.95
PLAC9	219348	-3.112	1.51E-44	0.874317	-3.6205	1.96E-74	0.905297	-3.131	1.08E-45	0.845238	-3.37	5.74E-17	0.95
NRXN1	9378	-3.426	2.70E-43	0.874317	-3.5284	1.08E-44	0.839486	-3.453	2.43E-39	0.809524	-2.79	2.63E-10	0.7
GRIN2B	2904	-2.555	1.07E-42	0.874317	-2.5457	2.17E-60	0.836276	-2.743	4.62E-58	0.900794	-2.567	2.21E-11	0.85
MMP27	64066	-2.695	1.41E-40	0.874317	-3.0247	1.37E-78	0.916533	-2.785	1.28E-45	0.869048	-2.728	1.62E-09	0.75
CAPZA3	93661	-2.718	1.56E-40	0.874317	-2.7375	2.77E-67	0.866774	-2.743	1.47E-44	0.876984	-2.696	3.10E-09	0.85
IRX6	79190	-3.369	1.29E-39	0.874317	-3.2114	3.30E-46	0.842697	-3.34	3.15E-40	0.861111	-3.042	3.41E-10	0.75
ANO3	63982	-3.315	8.08E-31	0.874317	-3.2257	1.40E-32	0.823435	-2.962	3.95E-24	0.789683	-2.468	6.13E-06	0.7
SPINK5	11005	-2.831	8.86E-27	0.874317	-2.7425	1.28E-27	0.823435	-2.755	2.14E-25	0.825397	-2.259	6.26E-06	0.7
CPA2	1358	-2.007	1.21E-26	0.874317	-2.0062	2.79E-42	0.861958	-2.221	2.58E-43	0.924603	-2.286	5.49E-08	1
DSG1	1828	-3.817	2.68E-26	0.874317	-4.1151	1.96E-38	0.868379	-4.284	1.32E-34	0.849206	-4.753	6.89E-13	0.85
FABP7	2173	-3.721	7.42E-18	0.874317	-3.4119	1.68E-16	0.784912	-4.146	1.46E-25	0.857143	-4.593	2.63E-08	0.85
C6	729	-2.606	5.40E-16	0.874317	-3.7168	1.11E-36	0.914928	-3.24	8.95E-23	0.869048	-3.424	5.16E-07	0.95
CAV2	858	-2.869	5.45E-70	0.868852	-3.0085	9.41E-113	0.874799	-2.95	1.99E-83	0.892857	-3.234	1.70E-26	0.9
HOXA5	3202	-3.137	3.39E-62	0.868852	-3.2823	5.73E-85	0.826645	-2.995	4.41E-66	0.805556	-2.91	5.00E-30	0.8
EBF3	253738	-2.787	1.69E-56	0.868852	-3.0488	7.45E-92	0.861958	-2.832	2.69E-60	0.833333	-3.135	4.83E-21	0.85
FZD4	8322	-2.276	8.21E-50	0.868852	-2.4792	2.45E-83	0.855538	-2.271	3.65E-56	0.84127	-2.315	8.69E-12	0.95
BMP5	653	-3.958	5.41E-49	0.868852	-3.7954	9.99E-47	0.847512	-4.075	4.70E-53	0.865079	-4.628	3.33E-25	0.9
RHOXF1	158800	-2.46	6.80E-40	0.868852	-2.5521	1.27E-66	0.87801	-2.495	1.06E-40	0.833333	-2.217	2.12E-08	0.9
MOSC1	64757	-2.318	1.17E-37	0.868852	-2.2503	4.24E-59	0.833066	-2.174	3.83E-39	0.829365	-2.352	1.34E-08	0.8
TMEM37	140738	-2.489	1.21E-36	0.868852	-2.6259	2.25E-76	0.890851	-2.481	2.09E-46	0.873016	-2.403	1.74E-08	1
CMTM5	116173	-3.047	8.22E-36	0.868852	-3.263	3.40E-57	0.871589	-3.221	1.27E-48	0.869048	-3.215	1.37E-13	0.85
DRD2	1813	-3.102	2.82E-32	0.868852	-3.0509	2.46E-38	0.817014	-3.082	3.31E-35	0.821429	-3.574	1.37E-12	0.9
APCDD1L	164284	-2.661	5.68E-28	0.868852	-2.6198	3.12E-35	0.820225	-2.787	6.15E-36	0.892857	-2.565	4.82E-08	0.85
SPOCK3	50859	-2.313	2.42E-21	0.868852	-2.5017	5.26E-37	0.860353	-2.512	6.72E-28	0.833333	-2.96	4.19E-08	0.95
CDR1	1038	-2.406	2.22E-20	0.868852	-2.448	3.14E-34	0.871589	-2.739	3.59E-33	0.93254	-2.876	9.03E-08	0.95
TMEM213	155006	-3.16	2.69E-20	0.868852	-2.895	1.52E-21	0.799358	-3.221	3.66E-24	0.853175	-2.481	0.000799	0.8
AFP	174	-3.045	3.00E-18	0.868852	-3.1213	1.82E-25	0.834671	-2.655	4.18E-14	0.797619	-2.389	0.002259	0.8

LMOD1	25802	-2.991	1.13E-72	0.863388	-3.1787	1.00E-95	0.845907	-2.81	1.60E-70	0.781746	-3.137	9.45E-34	0.9
NKAPL	222698	-2.761	7.22E-70	0.863388	-2.9019	1.34E-114	0.873194	-2.698	3.88E-80	0.845238	-2.836	8.41E-31	0.9
ALDH1A2	8854	-3.638	1.06E-59	0.863388	-4.1224	5.40E-90	0.900482	-3.717	7.52E-60	0.849206	-4.483	1.07E-33	0.9
C1QTNF9	338872	-3.201	1.07E-57	0.863388	-3.5273	1.95E-91	0.892456	-3.323	7.61E-64	0.857143	-3.143	8.76E-23	0.85
FREM1	158326	-3.713	8.48E-49	0.863388	-4.1443	1.97E-76	0.902087	-3.726	1.36E-52	0.873016	-5.115	2.58E-29	0.9
RELN	5649	-4.383	4.00E-48	0.863388	-5.0516	7.48E-83	0.929374	-4.601	6.69E-56	0.900794	-5.276	1.42E-26	0.95
C14orf139	79686	-2.517	1.71E-47	0.863388	-2.9776	3.97E-96	0.906902	-2.887	6.02E-60	0.888889	-2.818	2.63E-14	0.9
C3orf55	152078	-3.227	1.25E-44	0.863388	-3.282	9.20E-65	0.879615	-3.369	4.96E-54	0.900794	-3.67	5.38E-17	0.95
GFAP	2670	-3.7	2.27E-44	0.863388	-4.1215	1.34E-68	0.892456	-3.849	1.21E-48	0.869048	-4.068	1.65E-17	0.9
GALNTL2	117248	-3.098	3.26E-44	0.863388	-3.527	7.26E-82	0.902087	-3.281	1.68E-57	0.884921	-3.84	3.04E-20	0.95
CLDND2	125875	-2.557	7.04E-44	0.863388	-2.5572	8.50E-74	0.861958	-2.524	3.34E-50	0.833333	-2.235	1.22E-09	0.7
PLD5	200150	-2.959	3.76E-39	0.863388	-3.199	1.23E-70	0.87801	-3.313	9.28E-61	0.892857	-3.077	8.01E-17	0.85
SCN2A	6326	-3.165	2.08E-35	0.863388	-3.4083	3.07E-50	0.853933	-3.254	3.10E-40	0.837302	-3.783	4.26E-13	0.85
MYEOV	26579	-3.459	2.47E-32	0.863388	-3.2557	3.81E-35	0.807384	-3.332	8.55E-36	0.869048	-2.537	5.52E-06	0.7
SYN2	6854	-3.752	5.47E-58	0.857923	-4.1205	6.16E-68	0.836276	-3.949	3.04E-49	0.81746	-4.078	2.50E-25	0.8
NTRK2	4915	-3.756	7.81E-58	0.857923	-4.2714	5.81E-81	0.889246	-4.196	2.33E-65	0.888889	-4.946	2.81E-39	0.85
BMX	660	-3.363	1.67E-51	0.857923	-3.9382	4.00E-82	0.87801	-3.324	4.86E-49	0.789683	-4.101	3.20E-28	0.9
RNF150	57484	-3.225	7.27E-48	0.857923	-3.1966	3.70E-70	0.857143	-3.276	5.60E-67	0.884921	-3.708	3.74E-20	0.85
NLGN1	22871	-3.324	1.36E-43	0.857923	-3.5845	2.66E-61	0.836276	-3.484	8.06E-50	0.845238	-4.074	1.91E-25	0.9
D4S234E	27065	-3.136	1.13E-41	0.857923	-3.5258	9.70E-59	0.841091	-3.308	8.71E-45	0.809524	-3.81	3.95E-17	0.9
CNTFR	1271	-3.934	1.84E-41	0.857923	-4.1856	3.95E-49	0.834671	-3.854	5.90E-35	0.789683	-4.549	4.02E-22	1
C11orf87	399947	-2.184	8.27E-39	0.857923	-2.1327	5.64E-61	0.847512	-2.143	3.27E-39	0.880952	NA	NA	NA
ACTG2	72	-3.377	2.38E-34	0.857923	-3.7112	1.37E-45	0.847512	-3.893	5.84E-44	0.888889	-3.914	8.11E-14	0.85
XPNPEP2	7512	-3.009	4.89E-34	0.857923	-3.3793	1.36E-62	0.897271	-3.066	1.01E-40	0.880952	-3.404	7.26E-14	0.95
NEUROG2	63973	-3.153	2.42E-28	0.857923	-3.2208	5.08E-41	0.863563	-3.297	5.31E-36	0.900794	-3.425	2.35E-09	0.9
GRIA1	2890	-3.248	6.29E-27	0.857923	-3.4875	2.62E-34	0.857143	-3.382	3.14E-36	0.876984	-3.895	1.12E-16	0.95
PTN	5764	-2.82	3.77E-26	0.857923	-3.2334	1.27E-47	0.866774	-2.975	7.24E-29	0.849206	-3.853	5.15E-10	0.9
GRIK1	2897	-2.831	7.92E-21	0.857923	-3.2876	2.98E-49	0.911717	-3.14	1.04E-30	0.896825	-3.025	6.15E-07	0.8
GLRA3	8001	-2.788	7.54E-11	0.857923	-2.8547	3.45E-17	0.858748	-2.348	1.29E-08	0.793651	-2.978	0.001111	0.85
SLC26A3	1811	-2.279	6.23E-09	0.857923	-2.9937	9.48E-21	0.882825	-2.714	7.28E-14	0.876984	-3.554	2.33E-05	0.9
MEOX2	4223	-3.159	1.78E-56	0.852459	-3.6425	3.06E-73	0.853933	-3.065	1.89E-56	0.785714	-3.609	1.39E-29	0.95
PRRG3	79057	-2.933	5.68E-52	0.852459	-3.1779	1.18E-92	0.87801	-3.158	4.58E-73	0.90873	-3.39	3.92E-25	0.95
CCL14	6358	-3.546	3.77E-51	0.852459	-4.3878	1.79E-72	0.871589	-3.627	8.46E-50	0.821429	-3.824	7.74E-28	0.85
KLHL13	90293	-3.347	6.10E-49	0.852459	-3.3688	6.63E-65	0.853933	-3.21	4.53E-44	0.801587	-4.047	1.15E-18	0.85

SAA4	6291	-3.459	2.15E-43	0.852459	-3.438	2.30E-50	0.817014	-3.703	2.27E-50	0.84127	-3.802	1.09E-19	0.85
TMEM195	392636	-2.984	8.62E-42	0.852459	-3.1858	4.21E-72	0.900482	-2.978	1.96E-45	0.853175	-3.542	1.88E-17	0.9
GPR133	283383	-2.786	4.68E-39	0.852459	-3.078	3.87E-58	0.836276	-2.691	5.20E-38	0.765873	-3.591	4.82E-16	0.95
RNASE7	84659	-3.134	4.06E-35	0.852459	-3.4387	7.23E-57	0.853933	-3.206	2.03E-38	0.829365	-3.903	7.31E-13	0.85
DMRT2	10655	-2.754	8.21E-35	0.852459	-2.9965	3.73E-57	0.850722	-2.952	5.25E-46	0.876984	-3.259	1.10E-13	0.85
KCNA2	3737	-2.395	7.85E-33	0.852459	-2.4614	9.79E-57	0.87801	-2.508	2.01E-44	0.884921	-2.705	5.84E-10	0.85
CCDC141	285025	-2.275	1.32E-32	0.852459	-2.4768	2.03E-61	0.910112	-2.451	6.24E-43	0.884921	-2.859	1.16E-13	0.9
DKK4	27121	-2.049	1.20E-30	0.852459	NA	NA	NA	-2.018	4.26E-36	0.813492	-2.447	9.06E-12	0.95
CLDN10	9071	-2.83	1.74E-26	0.852459	-2.8158	1.64E-27	0.817014	-3.16	1.30E-34	0.873016	-3.476	5.25E-12	0.85
RASGEF1C	255426	-2.801	1.47E-25	0.852459	-2.6821	3.50E-31	0.805778	-3.143	1.11E-43	0.892857	-2.949	4.76E-09	0.8
LIFR	3977	-2.862	3.06E-68	0.846995	-3.0362	4.76E-90	0.834671	-2.96	7.17E-67	0.801587	-3.379	2.65E-32	0.95
PALMD	54873	-2.753	4.11E-67	0.846995	-3.0125	4.76E-104	0.879615	-2.867	3.36E-72	0.865079	-2.996	1.32E-23	0.9
FAM107A	11170	-3.573	1.22E-58	0.846995	-3.9552	4.56E-77	0.873194	-3.766	8.14E-62	0.861111	-4.289	1.44E-31	0.9
FGF2	2247	-3.194	5.05E-52	0.846995	-3.6519	2.66E-77	0.857143	-3.563	1.04E-63	0.869048	-4.385	9.45E-34	0.95
AVPR2	554	-2.998	1.78E-50	0.846995	-3.4475	4.24E-79	0.857143	-3.017	8.74E-45	0.738095	-3.181	4.25E-22	0.8
CCDC69	26112	-2.619	1.00E-49	0.846995	-2.8758	4.56E-86	0.88764	-2.703	4.53E-58	0.869048	-3.073	1.41E-16	0.9
SFRP1	6422	-4.455	2.68E-45	0.846995	-4.8283	3.59E-53	0.82183	-4.946	2.37E-49	0.857143	-5.85	1.59E-27	0.8
EPHB1	2047	-3.01	9.21E-42	0.846995	-3.0125	6.77E-58	0.81862	-3.194	4.63E-56	0.888889	-3.463	4.21E-15	0.85
HTR2A	3356	-2.961	2.16E-38	0.846995	-3.1552	3.10E-60	0.874799	-3.121	1.75E-47	0.884921	-3.527	7.41E-14	0.9
TEPP	374739	-2.376	1.34E-35	0.846995	-2.5319	1.10E-62	0.866774	-2.35	4.43E-36	0.813492	-2.408	4.62E-08	0.8
CMA1	1215	-3.416	2.35E-35	0.846995	-3.8423	7.83E-63	0.876404	-3.61	5.81E-42	0.853175	-4.373	1.14E-15	0.9
CD209	30835	-2.802	4.41E-35	0.846995	-2.7397	1.35E-53	0.844302	-2.616	1.58E-38	0.821429	-2.684	1.36E-09	0.95
FGFBP1	9982	-4.173	6.32E-34	0.846995	-4.1666	2.01E-40	0.823435	-4.647	3.39E-49	0.892857	-5.307	7.45E-19	0.9
ALX4	60529	-3.052	1.22E-33	0.846995	-3.1857	9.02E-50	0.845907	-3.125	3.28E-38	0.849206	-3.576	4.12E-12	0.85
GABRA2	2555	-2.66	1.25E-30	0.846995	-2.8632	3.45E-41	0.836276	-2.866	1.16E-36	0.84127	-2.87	7.66E-10	0.85
LMX1A	4009	-2.376	7.34E-28	0.846995	-2.5562	6.51E-49	0.869984	-2.543	4.53E-36	0.857143	-2.704	9.28E-09	0.85
TTYH1	57348	-3.421	3.02E-27	0.846995	-3.1753	5.96E-27	0.810594	-3.549	2.32E-38	0.873016	-3.773	8.37E-12	0.85
IL6	3569	-2.844	5.64E-23	0.846995	-3.1711	2.35E-39	0.855538	-3.109	2.22E-31	0.876984	-3.827	2.69E-11	0.95
OLFM4	10562	-3.801	3.61E-20	0.846995	-4.0418	6.81E-26	0.834671	-4.112	5.65E-27	0.837302	-5.036	8.38E-12	0.85
CITED1	4435	-2.67	4.03E-19	0.846995	-2.8808	6.01E-29	0.812199	-2.778	6.73E-22	0.805556	-2.876	3.76E-06	0.8
COL17A1	1308	-3.438	1.82E-18	0.846995	-4.2492	1.41E-37	0.869984	-3.635	6.14E-21	0.809524	-5.015	3.07E-08	0.85
MUCL1	118430	-3.45	2.41E-10	0.846995	-3.9755	3.46E-17	0.823435	-3.464	1.61E-11	0.809524	-3.734	0.002063	0.8
PLA2G2A	5320	-2.225	4.55E-10	0.846995	-2.703	5.72E-16	0.82504	-2.469	8.33E-12	0.789683	-3.042	6.75E-05	0.9
LRRTM2	26045	-2.94	6.39E-62	0.84153	-3.1289	3.36E-95	0.874799	-2.907	8.06E-68	0.833333	-3.427	5.49E-29	0.9

PGM5	5239	-3.009	6.00E-61	0.84153	-3.4957	2.90E-74	0.817014	-2.945	9.65E-54	0.742063	-3.107	1.28E-26	0.75
SIK2	23235	-2.194	6.58E-61	0.84153	-2.2308	4.05E-106	0.810594	-2.245	7.44E-71	0.813492	-2.422	1.87E-17	0.9
HLF	3131	-3.407	1.15E-56	0.84153	-3.8769	6.79E-83	0.866774	-3.822	6.56E-64	0.865079	-3.665	6.84E-30	0.9
NRG2	9542	-3.776	1.37E-51	0.84153	-3.8553	3.04E-66	0.820225	-3.932	1.49E-55	0.865079	-4.047	4.12E-23	0.7
LRRN3	54674	-2.741	2.48E-42	0.84153	-2.9832	4.28E-58	0.852327	-2.912	1.26E-49	0.821429	-2.905	3.46E-13	0.8
ART4	420	-2.753	1.98E-37	0.84153	-2.9357	2.47E-51	0.850722	-2.839	6.04E-48	0.84127	-3.151	5.33E-14	0.9
LPPR1	54886	-3.356	3.48E-36	0.84153	-3.4054	2.06E-50	0.829856	-3.348	2.31E-38	0.809524	-3.759	6.28E-13	0.85
FLNC	2318	-2.008	3.77E-26	0.84153	-2.2612	1.12E-41	0.88764	NA	NA	NA	-2.191	2.56E-11	0.85
PI3	5266	-3.55	3.89E-24	0.84153	-3.2395	3.65E-22	0.76886	-3.783	2.33E-29	0.873016	-4.385	2.73E-12	0.9
C8orf34	116328	-2.31	8.10E-22	0.84153	-2.646	3.72E-39	0.842697	-2.532	4.88E-33	0.853175	-2.951	4.79E-12	0.9
NTF4	4909	-2.536	3.26E-21	0.84153	-2.8384	9.59E-39	0.807384	-2.681	1.62E-22	0.769841	-2.928	5.24E-06	0.85
NR0B1	190	-2.007	4.30E-20	0.84153	NA	NA	NA	NA	NA	NA	-2.473	1.60E-10	0.95
C5orf23	79614	-2.098	2.14E-19	0.84153	-2.1365	3.56E-26	0.791332	-2.05	1.02E-20	0.813492	-2.434	5.00E-06	0.8
ROPN1B	152015	-3.242	2.30E-19	0.84153	-3.2114	7.07E-23	0.797753	-3.726	5.14E-31	0.880952	-3.906	2.47E-08	0.8
RSPO3	84870	-2.929	3.05E-51	0.836066	-3.0618	4.72E-73	0.837881	-2.943	2.86E-57	0.821429	-3.033	1.98E-22	0.9
PDZD2	23037	-2.576	3.89E-46	0.836066	-2.7407	1.51E-72	0.815409	-2.643	1.39E-50	0.793651	-3.051	3.84E-17	0.8
SHISA3	152573	-2.741	7.77E-40	0.836066	-2.9446	9.52E-58	0.842697	-2.714	1.49E-32	0.769841	-2.961	2.54E-12	0.9
MUSTN1	389125	-2.099	4.52E-34	0.836066	-2.3257	2.32E-51	0.874799	NA	NA	NA	-2.615	7.27E-15	0.9
LILRB5	10990	-2.672	8.95E-30	0.836066	-2.5534	9.63E-44	0.839486	-2.38	2.94E-29	0.81746	-2.387	1.09E-06	0.85
LOC286002	286002	-2.49	1.52E-23	0.836066	-2.3689	1.39E-28	0.783307	-2.665	1.79E-30	0.84127	-2.232	1.80E-05	0.65
CFTR	1080	-2.049	2.44E-16	0.836066	NA	NA	NA	NA	NA	NA	NA	NA	NA
KLK5	25818	-3.301	4.52E-15	0.836066	-3.52	1.47E-19	0.788122	-3.568	1.47E-17	0.825397	-5.525	2.76E-09	0.9
HOXA4	3201	-2.862	8.97E-73	0.830601	-2.9796	1.56E-85	0.789727	-2.714	4.03E-69	0.761905	-2.831	1.34E-35	0.85
ENPP2	5168	-2.956	8.82E-57	0.830601	-3.2162	4.91E-87	0.844302	-2.997	1.01E-61	0.81746	-3.121	1.85E-22	0.9
SEMA3G	56920	-2.653	3.99E-52	0.830601	-3.0678	2.84E-83	0.866774	-2.789	5.16E-59	0.837302	-3.301	4.12E-22	0.9
SLC22A3	6581	-3.352	1.30E-45	0.830601	-3.5775	1.54E-67	0.871589	-3.386	9.58E-55	0.865079	-3.655	5.61E-21	0.95
ZBTB16	7704	-4.059	5.57E-42	0.830601	-4.7923	4.36E-68	0.903692	-4.384	4.56E-49	0.876984	-5.084	1.22E-28	1
CNKSR2	22866	-3.033	2.63E-40	0.830601	-3.0425	2.43E-53	0.826645	-3.227	5.00E-53	0.845238	-3.645	1.71E-17	0.8
SLC16A12	387700	-3.185	9.16E-40	0.830601	-3.4709	3.94E-65	0.866774	-3.112	9.69E-46	0.813492	-3.51	9.14E-20	0.95
ELANE	1991	-2.26	2.77E-38	0.830601	-2.3508	9.10E-62	0.826645	-2.132	2.02E-36	0.793651	-2.521	2.19E-12	0.8
ACSL1	2180	-2.362	2.48E-36	0.830601	-2.4077	2.45E-63	0.868379	-2.336	9.63E-45	0.857143	-2.441	1.10E-09	0.95
DLK2	65989	-2.787	2.71E-28	0.830601	-3.0619	1.57E-45	0.826645	-2.928	8.37E-31	0.777778	-3.597	8.74E-10	0.85
PTCHD3	374308	-2.783	1.23E-26	0.830601	-2.8015	2.17E-36	0.82183	-2.826	1.31E-30	0.837302	-3.507	1.62E-13	0.95
BBOX1	8424	-3.578	9.70E-26	0.830601	-3.8517	4.17E-34	0.781701	-3.873	6.71E-30	0.813492	-4.267	1.94E-10	0.75

MYO16	23026	-2.261	3.06E-25	0.830601	-2.2455	4.03E-30	0.781701	-2.399	9.81E-28	0.809524	-2.727	1.44E-08	0.85
SLC4A4	8671	-2.776	7.90E-24	0.830601	-3.2063	1.19E-45	0.897271	-3.11	9.67E-35	0.888889	-3.619	2.96E-13	0.95
CDH4	1002	-2.252	9.01E-24	0.830601	-2.3387	1.43E-38	0.836276	-2.418	3.75E-36	0.880952	-2.415	5.64E-08	0.85
CHST9	83539	-3.206	2.19E-23	0.830601	-3.537	2.49E-36	0.82183	-3.397	3.97E-29	0.857143	-3.816	8.45E-08	0.8
ATP13A5	344905	-3.239	2.74E-18	0.830601	-3.4113	2.55E-25	0.799358	-3.347	1.41E-17	0.769841	-3.277	4.87E-05	0.75
B3GAT1	27087	-2.283	2.55E-17	0.830601	-2.3318	4.08E-23	0.791332	-2.414	3.43E-21	0.81746	-2.669	3.29E-07	0.9
SIM1	6492	-2.322	1.57E-16	0.830601	-2.473	3.10E-25	0.82183	-2.586	4.34E-23	0.849206	NA	NA	NA
RFX6	222546	-2.292	6.27E-16	0.830601	-2.6342	5.14E-32	0.858748	-2.189	1.64E-14	0.81746	-2.359	0.000131	0.8
MAB21L1	4081	-3.204	3.02E-63	0.825137	-3.4732	3.78E-83	0.860353	-2.979	4.31E-51	0.753968	-3	2.50E-25	0.85
GIPC2	54810	-3.054	2.47E-62	0.825137	-3.4372	1.64E-90	0.829856	-3.209	3.37E-68	0.813492	-3.551	6.41E-36	0.9
SSTR1	6751	-3.107	6.50E-56	0.825137	-3.4571	6.35E-74	0.823435	-3.095	3.04E-50	0.777778	-2.969	3.38E-15	0.8
F10	2159	-2.888	1.78E-52	0.825137	-3.1631	4.45E-80	0.866774	-2.765	7.82E-49	0.781746	-2.845	1.79E-16	0.75
FHL5	9457	-2.775	8.80E-50	0.825137	-3.0898	1.52E-68	0.857143	-2.6	9.40E-44	0.77381	-3.242	3.42E-20	0.9
RGS6	9628	-3.291	1.96E-49	0.825137	-3.6055	1.14E-63	0.808989	-3.589	1.87E-51	0.813492	-3.471	2.15E-28	0.75
SCN2B	6327	-3.547	1.79E-46	0.825137	-4.1734	2.48E-73	0.863563	-3.715	2.84E-52	0.837302	-4.12	1.36E-22	0.8
DNASE1L3	1776	-3.38	5.43E-45	0.825137	-3.938	1.08E-65	0.841091	-3.724	1.62E-51	0.821429	-3.984	4.09E-24	0.85
BMP2	650	-2.558	2.80E-42	0.825137	-2.682	1.16E-58	0.810594	-2.768	5.73E-52	0.853175	-2.834	3.47E-14	0.8
PLP1	5354	-3.433	9.55E-42	0.825137	-3.8633	7.58E-50	0.820225	-3.497	1.05E-38	0.785714	-4.195	3.43E-23	0.75
LOXL4	84171	-2.785	4.30E-40	0.825137	-2.8426	9.66E-52	0.778491	-2.954	1.03E-62	0.829365	-3.325	8.68E-28	0.85
AOC2	314	-2.249	4.96E-40	0.825137	-2.2835	3.67E-58	0.81862	-2.422	1.93E-48	0.845238	-2.741	6.80E-14	0.9
PTPRZ1	5803	-4.099	3.24E-31	0.825137	-4.2034	1.85E-38	0.82183	-4.299	1.21E-32	0.813492	-5.658	1.27E-17	0.95
CSRNP3	80034	-2.578	6.70E-31	0.825137	-2.775	1.26E-49	0.839486	-2.734	2.06E-39	0.861111	-3.082	1.84E-10	0.85
PTGS2	5743	-2.712	1.65E-27	0.825137	-3.0865	8.82E-41	0.82504	-3.365	7.79E-46	0.880952	-4.528	4.41E-21	0.95
SOX10	6663	-4.041	4.49E-22	0.825137	-4.5303	4.98E-28	0.784912	-4.588	9.38E-28	0.821429	-5.791	3.19E-12	0.75
MARCO	8685	-3.15	3.07E-21	0.825137	-2.7378	2.68E-19	0.75923	-3.095	3.12E-23	0.801587	-2.615	2.10E-06	0.8
ROPN1	54763	-3.489	7.17E-21	0.825137	-3.4246	2.23E-23	0.778491	-3.996	2.48E-32	0.865079	-4.379	1.39E-09	0.8
OCA2	4948	-2.37	8.76E-19	0.825137	-2.2491	3.83E-19	0.760835	-2.78	1.18E-35	0.849206	-2.266	1.88E-06	0.75
SYCE1	93426	-2.581	1.08E-18	0.825137	-2.9057	3.28E-34	0.861958	-2.724	4.21E-24	0.849206	-2.684	4.50E-06	0.8
CDH19	28513	-2.603	1.53E-18	0.825137	-2.5492	4.09E-19	0.792937	-3.2	2.07E-40	0.857143	-2.609	2.54E-08	0.8
KLK7	5650	-3.364	4.16E-18	0.825137	-3.4312	5.04E-21	0.776886	-3.609	1.06E-20	0.81746	-5.209	3.52E-10	0.85
C4orf49	84709	-2.523	3.55E-53	0.819672	-2.634	2.22E-79	0.81862	-2.472	1.23E-52	0.769841	-2.404	1.02E-13	0.7
LPHN3	23284	-3.237	1.41E-47	0.819672	-3.451	5.83E-61	0.844302	-3.218	1.46E-47	0.829365	-3.426	8.39E-21	0.9
JPH2	57158	-2.901	1.65E-46	0.819672	-2.9283	3.31E-55	0.812199	-2.941	2.74E-47	0.845238	-2.606	5.48E-14	0.85
MYOCD	93649	-2.762	1.11E-43	0.819672	-3.0911	1.42E-60	0.857143	-2.719	5.24E-43	0.801587	-3.702	9.08E-22	0.9

SCN9A	6335	-2.593	1.48E-42	0.819672	-2.6204	5.50E-68	0.841091	-2.417	2.78E-47	0.805556	-2.869	3.72E-16	0.85
LOC201651	201651	-2.082	1.72E-42	0.819672	-2.265	2.50E-73	0.844302	-2.168	4.78E-48	0.81746	-2.396	8.74E-15	0.9
LOC339524	339524	-2.52	2.74E-41	0.819672	-2.8218	3.04E-68	0.842697	-2.62	1.63E-49	0.805556	-3.083	3.36E-16	0.9
KCTD4	386618	-2.178	1.09E-40	0.819672	-2.3017	5.70E-78	0.906902	-2.262	2.12E-47	0.880952	-2.418	8.38E-12	0.9
GABRE	2564	-3.597	1.39E-40	0.819672	-3.553	4.57E-52	0.831461	-3.722	2.98E-47	0.861111	-3.521	8.40E-15	0.8
PHKG1	5260	-2.431	2.95E-39	0.819672	-2.5898	6.00E-50	0.842697	-2.586	2.12E-40	0.829365	-2.481	6.94E-12	0.8
PTCHD1	139411	-3.655	8.70E-33	0.819672	-3.6349	1.61E-37	0.778491	-4.017	5.11E-43	0.857143	-3.418	1.15E-09	0.75
FLJ35024	401491	-2.424	2.51E-32	0.819672	-2.1156	5.20E-35	0.744783	-2.473	1.93E-40	0.809524	NA	NA	NA
EFCAB1	79645	-3.066	3.96E-31	0.819672	-3.4059	9.66E-54	0.831461	-3.227	1.58E-36	0.829365	-2.755	1.62E-07	0.75
IL17B	27190	-2.978	3.46E-27	0.819672	-3.5183	1.64E-54	0.874799	-3.191	4.70E-34	0.861111	-3.607	1.12E-10	0.85
SLC29A4	222962	-2.281	2.46E-26	0.819672	-2.0928	1.87E-34	0.778491	-2.084	8.05E-26	0.789683	NA	NA	NA
ADRB1	153	-2.592	1.21E-25	0.819672	-2.7862	1.81E-31	0.810594	-2.831	1.62E-30	0.813492	-2.619	6.19E-09	0.8
SOSTDC1	25928	-3.426	5.48E-24	0.819672	-3.5752	7.36E-29	0.776886	-4.05	2.60E-34	0.873016	-4.341	6.62E-10	0.8
EDAR	10913	-2.523	1.54E-21	0.819672	-2.7103	1.00E-36	0.823435	-2.82	2.97E-30	0.84127	-3.192	2.79E-08	0.8
STAC2	342667	-3.861	5.89E-19	0.819672	-4.3613	8.13E-29	0.808989	-4.395	2.87E-22	0.829365	-5.453	1.25E-08	0.75
KRT5	3852	-3.263	9.80E-17	0.819672	-3.8548	4.57E-22	0.767255	-3.959	4.00E-20	0.821429	-4.948	2.84E-08	0.8
KRT14	3861	-3.341	7.42E-16	0.819672	-4.2104	3.63E-25	0.849117	-4.141	6.81E-20	0.833333	-5.293	1.99E-08	0.9
C2orf82	389084	-2.351	2.63E-11	0.819672	-2.6903	2.10E-21	0.837881	-2.686	1.02E-18	0.84127	-2.84	4.73E-05	0.8
GSN	2934	-2.269	2.34E-64	0.814208	-2.4202	3.19E-115	0.82825	-2.331	4.32E-67	0.753968	-2.381	8.64E-19	0.9
ADRB2	154	-2.829	3.18E-61	0.814208	-3.2967	1.04E-90	0.857143	-3.03	1.79E-68	0.853175	-3.506	1.31E-27	0.9
HOXA6	3203	-3.04	1.13E-51	0.814208	-3.1828	3.06E-68	0.791332	-2.924	1.70E-54	0.805556	-2.712	2.20E-19	0.8
GPC3	2719	-3.449	1.07E-46	0.814208	-3.8842	1.48E-66	0.871589	-3.631	2.86E-57	0.869048	-3.855	2.01E-25	0.8
AKAP12	9590	-2.587	1.40E-42	0.814208	-2.8708	1.21E-76	0.836276	-2.584	1.86E-52	0.821429	-2.962	1.22E-16	0.85
RGS7BP	401190	-2.807	3.82E-42	0.814208	-2.8246	7.10E-48	0.778491	-2.659	2.57E-38	0.742063	-3.209	4.02E-15	0.75
AOX1	316	-2.933	6.94E-41	0.814208	-3.1799	3.15E-56	0.833066	-2.908	7.17E-42	0.809524	-3.764	4.66E-19	0.85
FAM89A	375061	-2.326	1.61E-38	0.814208	-2.4504	1.96E-63	0.836276	-2.393	4.39E-44	0.821429	-2.53	4.66E-11	0.8
NCRNA00092	1E+08	-2.947	2.60E-35	0.814208	-2.8729	1.20E-39	0.810594	-3.199	5.43E-51	0.849206	-3.302	3.16E-17	0.85
UNC13C	440279	-2.002	1.52E-31	0.814208	-2.1285	3.80E-45	0.823435	-2.043	2.70E-34	0.769841	NA	NA	NA
NAT8L	339983	-2.757	6.83E-30	0.814208	-2.9348	1.07E-45	0.839486	-2.733	1.91E-34	0.821429	-2.735	6.34E-09	0.85
BCHE	590	-2.537	6.17E-28	0.814208	-2.7456	1.12E-40	0.815409	-2.607	3.03E-30	0.821429	NA	NA	NA
EMILIN3	90187	-2.93	1.93E-26	0.814208	-2.9175	1.65E-35	0.807384	-2.945	1.40E-31	0.821429	-3.034	1.26E-10	0.85
MPZ	4359	-2.02	3.04E-24	0.814208	NA	NA	NA	-2.158	1.91E-34	0.837302	-2.13	8.12E-09	0.75
IL22RA2	116379	-2.32	2.88E-14	0.814208	-2.1174	1.92E-17	0.733547	-2.559	1.85E-22	0.833333	-2.086	0.00092	0.75
TAT	6898	-3.987	7.41E-14	0.814208	-4.7331	3.69E-29	0.869984	-4.317	4.59E-19	0.857143	-6.48	4.00E-09	0.9

KLHL29	114818	-2.936	1.56E-68	0.808743	-3.1928	2.04E-101	0.841091	-3.165	4.54E-76	0.845238	-3.569	3.98E-36	0.85
LOC286367	286367	-2.475	3.24E-63	0.808743	-2.7398	6.50E-106	0.831461	-2.8	4.67E-70	0.81746	-2.72	8.94E-25	0.8
DENND2A	27147	-2.542	1.12E-60	0.808743	-2.5047	1.03E-81	0.760835	-2.439	3.07E-68	0.769841	-2.221	1.11E-17	0.7
MATN2	4147	-3.214	1.92E-57	0.808743	-3.5789	4.33E-82	0.81862	-3.252	7.71E-64	0.777778	-3.743	1.50E-34	0.85
SYNPO2	171024	-2.755	5.45E-56	0.808743	-3.0177	8.90E-70	0.813804	-2.657	4.15E-55	0.781746	-3.246	8.17E-29	0.85
ADAM33	80332	-3.347	3.40E-50	0.808743	-3.9042	4.17E-78	0.850722	-3.327	2.36E-47	0.761905	-4.022	4.25E-28	0.9
GPR17	2840	-2.609	3.07E-42	0.808743	-2.9676	6.14E-71	0.836276	-2.705	1.93E-44	0.793651	-2.883	3.71E-18	0.85
BMPER	168667	-3.019	3.34E-38	0.808743	-3.5034	3.90E-65	0.855538	-3.276	3.21E-51	0.821429	-3.373	1.03E-15	0.85
SNTG2	54221	-2.755	7.24E-36	0.808743	-3.042	9.98E-58	0.850722	-2.986	1.85E-43	0.825397	-3.35	3.64E-16	0.9
GPR12	2835	-3.39	4.96E-30	0.808743	-3.4346	3.81E-35	0.778491	-3.714	2.83E-45	0.833333	-3.673	2.21E-16	0.75
ACE2	59272	-2.549	1.81E-22	0.808743	-2.1798	5.00E-16	0.70305	-2.613	7.37E-28	0.75	-2.521	1.48E-12	0.65
TSPAN8	7103	-3.134	2.05E-22	0.808743	-3.1251	3.51E-22	0.739968	-3.064	1.16E-21	0.694444	-3.516	6.82E-11	0.75
KLK8	11202	-3.199	9.22E-20	0.808743	-2.9563	3.28E-19	0.749599	-3.173	1.11E-19	0.781746	-4.45	3.23E-10	0.85
CLDN8	9073	-2.286	9.21E-12	0.808743	-2.787	1.65E-23	0.81862	-2.713	7.94E-17	0.805556	-2.875	0.000892	0.8
HSPB2	3316	-2.789	3.39E-63	0.803279	-2.9041	1.18E-83	0.829856	-2.67	1.98E-62	0.781746	-2.609	6.18E-24	0.85
C1QTNF9B	387911	-2.536	1.78E-50	0.803279	-2.7387	1.14E-74	0.81862	-2.618	4.54E-53	0.813492	-2.35	9.54E-16	0.65
TPO	7173	-3.049	4.16E-49	0.803279	-3.5648	8.09E-79	0.876404	-3.088	1.24E-54	0.809524	-3.611	8.19E-24	0.85
ALDH1A1	216	-2.832	2.55E-48	0.803279	-2.9683	3.66E-75	0.812199	-2.655	1.47E-60	0.753968	-3.258	5.49E-24	0.9
CLDN5	7122	-2.719	4.00E-48	0.803279	-3.088	1.34E-76	0.829856	-2.601	7.13E-40	0.730159	-2.557	2.24E-17	0.7
TCF21	6943	-2.149	1.32E-42	0.803279	-2.2149	1.08E-67	0.820225	-2.033	9.42E-42	0.797619	NA	NA	NA
HOXA9	3205	-2.614	2.29E-42	0.803279	-2.7524	7.56E-54	0.807384	-2.58	4.16E-48	0.805556	-2.812	1.05E-25	0.85
KCNMB1	3779	-2.598	4.85E-38	0.803279	-2.8122	1.40E-65	0.82504	-2.749	5.11E-46	0.833333	-2.768	5.59E-11	0.8
LOC158376	158376	-2.246	1.64E-37	0.803279	-2.4003	9.49E-59	0.802568	-2.439	6.33E-54	0.793651	-2.622	4.64E-19	0.85
GLDN	342035	-2.575	9.55E-36	0.803279	-2.7719	7.11E-48	0.81862	-2.344	1.11E-33	0.765873	-2.894	3.81E-13	0.85
ASTN1	460	-2.889	3.37E-35	0.803279	-3.1604	8.65E-46	0.807384	-2.98	1.11E-31	0.785714	-3.606	8.74E-14	0.8
DDR2	4921	-2.587	1.95E-33	0.803279	-2.6354	2.35E-50	0.815409	-2.593	2.07E-39	0.837302	-2.802	1.46E-11	0.9
PSD2	84249	-2.397	4.14E-33	0.803279	-2.408	9.53E-45	0.786517	-2.273	2.25E-31	0.777778	-2.364	1.49E-07	0.75
C1QTNF7	114905	-2.614	4.24E-31	0.803279	-3.0386	1.13E-45	0.817014	-2.543	2.25E-35	0.809524	-3.179	9.40E-13	0.9
CCL28	56477	-2.967	7.22E-26	0.803279	-3.2819	1.13E-39	0.82504	-3.213	3.63E-35	0.849206	-4.081	2.02E-12	0.95
TP53AIP1	63970	-2.368	1.48E-21	0.803279	-2.6162	2.45E-40	0.817014	-2.54	4.62E-26	0.84127	-2.571	1.99E-05	0.75
PI15	51050	-2.781	1.13E-15	0.803279	-3.3593	9.69E-32	0.853933	-3.284	4.69E-24	0.861111	-3.571	1.27E-06	0.8
FAM13A	10144	-2.499	2.72E-69	0.797814	-2.5634	4.59E-119	0.841091	-2.639	1.13E-84	0.849206	-2.771	2.05E-25	0.85
GNAI1	2770	-2.487	7.58E-57	0.797814	-2.5368	2.66E-81	0.776886	-2.503	1.06E-59	0.789683	-2.632	7.52E-18	0.8
PID1	55022	-2.732	5.81E-54	0.797814	-2.8095	2.93E-71	0.788122	-2.714	2.63E-56	0.777778	-2.868	1.14E-17	0.75

INMT	11185	-2.937	1.27E-51	0.797814	-3.2408	4.65E-71	0.799358	-2.621	5.91E-47	0.714286	-3.11	2.16E-22	0.75
IQSEC3	440073	-2.586	8.61E-42	0.797814	-2.7404	2.71E-59	0.789727	-2.641	1.19E-45	0.797619	-3.19	8.27E-20	0.95
AKAP6	9472	-2.218	2.02E-41	0.797814	-2.2955	7.96E-66	0.773676	-2.227	4.41E-52	0.813492	-2.627	6.14E-18	0.9
EGR1	1958	-2.72	6.21E-41	0.797814	-3.1661	7.58E-73	0.831461	-3.075	2.91E-48	0.801587	-3.98	5.76E-24	0.9
C11orf92	399948	-2.849	5.11E-37	0.797814	-2.7632	1.07E-35	0.760835	-2.55	3.45E-29	0.72619	-3.137	2.43E-13	0.8
NCAM1	4684	-2.666	5.14E-34	0.797814	-2.7915	1.08E-47	0.776886	-2.937	1.64E-52	0.797619	-3.095	9.76E-24	0.8
PIWIL2	55124	-2.223	2.74E-27	0.797814	-2.44	9.80E-39	0.805778	-2.503	5.58E-37	0.821429	-2.721	2.30E-11	0.85
HSD11B1	3290	-2.338	1.17E-23	0.797814	-2.5272	1.38E-40	0.841091	-2.447	5.81E-31	0.833333	-3	1.37E-09	0.9
FAM166B	730112	-2.09	2.71E-22	0.797814	-2.3349	2.09E-39	0.849117	-2.193	1.07E-27	0.81746	NA	NA	NA
C1QL2	165257	-2.391	1.72E-19	0.797814	-2.1775	9.79E-18	0.743178	-2.548	4.29E-24	0.805556	-2.833	4.11E-07	0.8
LOC121838	121838	-2.371	1.97E-19	0.797814	-2.4963	3.59E-28	0.780096	-2.517	1.27E-26	0.801587	-2.242	4.17E-06	0.7
DCX	1641	-2.612	5.60E-17	0.797814	-2.5841	3.42E-17	0.752809	-2.998	2.30E-22	0.845238	-3.404	7.22E-09	0.75
GABRP	2568	-3.249	8.12E-14	0.797814	-3.4951	2.76E-18	0.760835	-3.962	1.76E-21	0.857143	-5.207	1.87E-08	0.75
HOXA7	3204	-2.752	1.78E-52	0.79235	-2.937	2.90E-65	0.752809	-2.709	4.74E-57	0.746032	-2.68	6.98E-35	0.8
PHYHIP	9796	-2.753	6.03E-51	0.79235	-2.9994	1.38E-74	0.802568	-2.636	7.17E-53	0.714286	-3.031	1.05E-24	0.75
CNTNAP3	79937	-3.37	1.83E-50	0.79235	-3.8546	2.18E-61	0.799358	-3.866	6.37E-59	0.837302	-4.76	6.10E-34	0.85
ABCA6	23460	-3.148	5.10E-48	0.79235	-3.5169	1.39E-73	0.855538	-3.039	7.87E-53	0.805556	-3.777	2.81E-24	0.95
IL33	90865	-3.378	9.96E-47	0.79235	-4.1278	7.67E-68	0.839486	-3.714	2.94E-47	0.789683	-4.771	1.23E-36	0.9
TACR1	6869	-3.276	4.65E-46	0.79235	-3.9085	1.72E-64	0.82825	-3.822	3.49E-54	0.801587	-4.103	2.39E-25	0.8
AKR1C3	8644	-2.548	2.38E-41	0.79235	-2.7788	1.23E-59	0.788122	-2.572	9.10E-48	0.821429	-2.816	2.18E-15	0.8
CXCL3	2921	-2.872	1.07E-38	0.79235	-2.8589	6.66E-50	0.76886	-3.141	5.75E-50	0.813492	-3.413	4.49E-21	0.9
FLJ37543	285668	-2.039	7.06E-32	0.79235	-2.1117	1.39E-46	0.805778	-2.143	2.51E-38	0.809524	-2.674	1.18E-13	0.9
LMO3	55885	-2.638	7.47E-27	0.79235	-2.4755	1.48E-24	0.741573	-2.415	3.65E-25	0.777778	-2.095	6.73E-06	0.6
CXCL5	6374	-2.467	3.54E-18	0.79235	-2.1464	2.11E-15	0.772071	-2.864	1.32E-27	0.892857	-2.821	3.87E-07	0.95
SCGB3A1	92304	-2.494	1.04E-10	0.79235	-3.4644	1.79E-24	0.831461	-3.409	1.52E-19	0.861111	-3.244	3.37E-05	0.8
UGT2B11	10720	-2.276	3.19E-05	0.79235	-3.2451	2.10E-14	0.873194	-2.844	3.03E-09	0.845238	NA	NA	NA
NRN1	51299	-2.514	9.27E-57	0.786885	-2.5092	3.30E-71	0.757624	-2.401	1.40E-58	0.746032	-2.495	2.28E-19	0.8
LOC145820	145820	-2.542	7.24E-50	0.786885	-2.8486	6.69E-72	0.820225	-2.501	2.97E-48	0.742063	-2.992	4.77E-21	0.85
KLF4	9314	-2.388	7.29E-47	0.786885	-2.6472	5.85E-76	0.820225	-2.556	1.17E-52	0.785714	-3.041	6.00E-18	0.9
MEOX1	4222	-3.06	2.25E-41	0.786885	-3.786	1.02E-60	0.812199	-3.355	7.91E-43	0.761905	-3.966	5.17E-26	0.85
OSR1	130497	-3.129	1.01E-37	0.786885	-3.6254	9.75E-47	0.754414	-3.532	3.03E-40	0.753968	-4.244	4.90E-24	0.8
WSCD2	9671	-2.864	9.98E-37	0.786885	-3.2716	1.91E-58	0.82825	-3.043	2.00E-52	0.825397	-3.302	1.39E-24	0.85
C14orf49	161176	-2.385	1.27E-36	0.786885	-2.6699	1.87E-68	0.857143	-2.536	9.30E-47	0.825397	-2.685	5.15E-12	0.85
PTGFR	5737	-2.962	2.35E-36	0.786885	-3.1083	6.01E-48	0.799358	-3.204	1.89E-49	0.84127	-3.893	2.46E-21	0.9

LOC440173	440173	-2.233	3.16E-34	0.786885	-2.214	6.42E-35	0.76244	-2.124	6.99E-27	0.761905	-2.003	1.70E-08	0.8
MYO1H	283446	-2.339	3.50E-34	0.786885	-2.2593	3.08E-40	0.756019	-2.236	5.09E-31	0.738095	NA	NA	NA
PROX1	5629	-2.835	5.72E-33	0.786885	-3.1218	1.95E-51	0.820225	-3.163	3.79E-46	0.84127	-3.536	3.62E-16	0.9
OGN	4969	-3.351	1.03E-32	0.786885	-4.1261	8.99E-51	0.820225	-3.568	2.48E-41	0.813492	-4.58	1.52E-24	0.9
PCDH11X	27328	-2.619	1.08E-22	0.786885	-2.8165	1.06E-35	0.796148	-3.111	1.36E-41	0.853175	-3.604	3.86E-19	0.85
KCNS1	3787	-2.362	8.95E-18	0.786885	-2.4987	2.21E-25	0.775281	-2.857	2.92E-29	0.861111	-2.524	1.39E-05	0.75
SLC6A14	11254	-3.237	4.26E-16	0.786885	-3.2871	1.11E-19	0.76886	-3.947	1.35E-26	0.849206	-4.038	3.02E-06	0.75
KLK6	5653	-2.742	2.70E-13	0.786885	-2.6828	3.68E-13	0.720706	-2.912	1.47E-14	0.761905	-4.113	2.04E-07	0.85
USP44	84101	-2.842	7.80E-54	0.781421	-3.1636	7.61E-79	0.820225	-3.081	2.26E-61	0.84127	-3.524	2.50E-29	0.9
TSHZ2	128553	-3.084	8.28E-52	0.781421	-3.316	5.60E-76	0.826645	-2.955	8.01E-57	0.793651	-3.774	5.40E-29	0.9
CCL16	6360	-2.305	3.31E-46	0.781421	-2.584	6.35E-84	0.815409	-2.186	6.77E-42	0.714286	-2.573	2.83E-18	0.8
C10orf108	414235	-2.44	2.20E-44	0.781421	-2.6364	2.56E-61	0.81862	-2.6	2.47E-58	0.813492	-2.568	5.34E-17	0.75
TMEM178	130733	-2.331	3.68E-39	0.781421	-2.648	5.64E-61	0.810594	-2.432	1.89E-40	0.757937	-2.599	7.83E-13	0.85
SOD3	6649	-2.578	6.59E-39	0.781421	-2.9242	4.11E-63	0.796148	-2.748	4.71E-43	0.753968	-3.026	6.27E-19	0.85
ECM2	1842	-2.372	1.09E-38	0.781421	-2.6105	1.34E-63	0.807384	-2.36	3.96E-43	0.781746	-2.714	1.31E-12	0.9
STK32A	202374	-2.496	3.46E-36	0.781421	-2.6999	2.55E-61	0.831461	-2.733	1.89E-55	0.869048	-3.082	3.48E-18	1
CRTAC1	55118	-2.818	4.02E-30	0.781421	-3.1435	1.08E-36	0.749599	-3.267	1.61E-39	0.777778	-2.627	1.35E-11	0.75
TP63	8626	-3.114	4.24E-25	0.781421	-3.539	4.42E-41	0.810594	-3.207	1.48E-26	0.777778	-4.578	1.16E-11	0.85
MUC15	143662	-3.339	2.19E-16	0.781421	-3.0981	5.13E-19	0.717496	-3.149	1.79E-16	0.753968	-2.636	0.0025	0.55
FOLR1	2348	-2.933	2.80E-15	0.781421	-3.045	5.73E-19	0.727127	-2.832	2.04E-14	0.718254	-3.098	6.17E-05	0.6
LY6D	8581	-2.611	4.11E-15	0.781421	-2.1106	1.31E-09	0.741573	-2.47	4.98E-15	0.785714	-2.934	3.13E-06	0.8
TAC1	6863	-2.18	4.70E-15	0.781421	-2.6808	1.10E-30	0.789727	-2.101	1.24E-15	0.714286	-3.089	6.65E-08	0.85
C7orf41	222166	-2.262	1.03E-54	0.775956	-2.473	2.18E-93	0.823435	-2.506	2.36E-67	0.829365	-2.695	1.69E-19	0.9
MRAS	22808	-2.392	1.12E-53	0.775956	-2.3237	1.49E-76	0.756019	-2.485	9.12E-80	0.825397	-2.331	6.82E-21	0.75
NECAB1	64168	-2.68	6.39E-50	0.775956	-2.7171	1.54E-64	0.788122	-2.566	6.03E-51	0.730159	-3.139	1.56E-28	0.9
EGFR	1956	-3.279	1.09E-46	0.775956	-3.2922	5.30E-51	0.75923	-3.479	2.23E-53	0.825397	-3.986	1.39E-28	0.85
CX3CL1	6376	-2.806	2.92E-44	0.775956	-2.9159	3.37E-65	0.781701	-3.009	9.39E-53	0.805556	-3.304	1.87E-19	0.9
PPP1R14A	94274	-2.781	3.42E-43	0.775956	-2.8587	3.16E-52	0.756019	-2.628	3.67E-38	0.690476	-2.383	2.83E-17	0.75
ODZ1	10178	-2.881	1.42E-42	0.775956	-2.9637	1.86E-54	0.788122	-2.876	3.33E-42	0.765873	-2.968	1.62E-17	0.75
SCN5A	6331	-2.658	4.13E-39	0.775956	-2.8878	3.23E-60	0.813804	-2.965	8.39E-48	0.789683	-3.271	9.10E-17	0.8
GFRA2	2675	-2.437	1.55E-33	0.775956	-2.8002	2.62E-55	0.81862	-2.698	1.33E-40	0.813492	-2.806	1.31E-12	0.9
PPARGC1A	10891	-2.549	1.84E-33	0.775956	-2.5503	3.40E-34	0.731942	-2.751	3.85E-43	0.765873	-2.78	8.89E-15	0.7
LCN6	158062	-2.194	1.02E-29	0.775956	-2.5373	3.00E-62	0.82825	-2.266	1.85E-31	0.753968	-2.588	1.65E-11	0.9
CLDN2	9075	-2.217	2.48E-26	0.775956	-2.0889	5.18E-27	0.736758	-2.225	9.70E-26	0.757937	-2.04	5.84E-07	0.85

OR2L13	284521	-2.037	3.32E-22	0.775956	-2.3434	2.15E-47	0.808989	-2.141	6.23E-26	0.769841	-2.509	1.14E-07	0.85
ELOVL3	83401	-2.047	3.27E-21	0.775956	NA	NA	NA	-2.115	4.18E-23	0.753968	NA	NA	NA
CCDC129	223075	-2.568	8.60E-18	0.775956	-2.4062	2.29E-20	0.752809	-2.73	2.55E-23	0.789683	-2.751	3.87E-06	0.8
VGLL1	51442	-2.859	7.86E-13	0.775956	-2.6402	1.89E-12	0.715891	-2.972	7.86E-17	0.789683	-3.253	1.33E-05	0.8
SCGN	10590	-2.134	1.88E-11	0.775956	-2.2672	1.21E-14	0.783307	-2.344	2.44E-15	0.781746	NA	NA	NA
LGI4	163175	-2.541	5.78E-55	0.770492	-2.678	2.48E-82	0.789727	-2.405	9.75E-53	0.714286	-2.451	2.96E-19	0.75
TGFBR3	7049	-2.953	6.31E-52	0.770492	-3.4991	4.98E-71	0.799358	-3.214	1.10E-59	0.77381	-3.602	6.08E-31	0.8
RGN	9104	-3.124	7.90E-46	0.770492	-3.3784	2.30E-56	0.760835	-3.49	4.17E-56	0.801587	-3.492	3.11E-31	0.8
COX7A1	1346	-2.288	1.21E-40	0.770492	-2.5928	2.59E-67	0.82504	-2.277	7.72E-43	0.761905	-2.007	1.34E-10	0.8
ODF3L1	161753	-2.363	5.72E-40	0.770492	-2.5166	1.71E-62	0.791332	-2.44	1.19E-45	0.789683	-3.046	6.21E-17	0.85
EBF2	64641	-2.563	2.31E-35	0.770492	-2.8022	1.47E-52	0.789727	-2.541	1.77E-36	0.765873	-2.941	1.13E-12	0.9
ABCC9	10060	-2.166	3.15E-35	0.770492	-2.3136	1.36E-54	0.770465	NA	NA	NA	-2.215	2.08E-09	0.9
KCNA1	3736	-2.616	1.32E-28	0.770492	-3.0313	3.51E-46	0.81862	-2.835	4.98E-33	0.757937	-3.962	1.41E-16	0.9
BNC1	646	-2.602	1.44E-27	0.770492	-2.9135	1.71E-36	0.807384	-3.007	1.06E-33	0.853175	-3.467	2.32E-14	0.9
SLC27A6	28965	-3.204	3.17E-26	0.770492	-3.5615	8.64E-39	0.786517	-3.667	1.22E-32	0.801587	-3.981	2.06E-11	0.8
CCL23	6368	-2.038	4.54E-25	0.770492	-2.1892	1.70E-40	0.764045	NA	NA	NA	-2.459	3.24E-09	0.8
DEFB1	1672	-2.849	1.21E-23	0.770492	-2.8721	5.75E-25	0.712681	-2.937	1.70E-26	0.722222	-3.405	6.21E-11	0.75
GPR64	10149	-2.129	1.03E-22	0.770492	-2.3066	7.23E-33	0.783307	-2.525	1.73E-35	0.876984	-2.344	5.18E-10	0.75
AK5	26289	-2.911	8.85E-22	0.770492	-3.4771	1.39E-40	0.82504	-3.234	8.41E-30	0.829365	-3.093	7.54E-07	0.8
NRG1	3084	-2.561	1.66E-20	0.770492	-2.8473	1.72E-33	0.804173	-2.909	9.26E-27	0.81746	-3.506	7.28E-08	0.9
SLC13A2	9058	-2.966	6.33E-15	0.770492	-3.35	1.81E-25	0.820225	-2.874	4.04E-15	0.765873	-3.246	6.21E-05	0.85
KL	9365	-2.778	1.48E-61	0.765027	-3.1778	1.24E-87	0.836276	-2.838	5.94E-65	0.801587	-3.454	1.52E-34	0.95
NDRG2	57447	-2.511	2.85E-60	0.765027	-2.6649	3.30E-77	0.76565	-2.744	2.60E-68	0.781746	-3.011	1.60E-27	0.75
GNAL	2774	-2.693	3.99E-58	0.765027	-2.9957	1.34E-76	0.788122	-2.904	2.59E-59	0.777778	-3.302	5.68E-27	0.8
LOC134466	134466	-2.581	1.34E-51	0.765027	-2.6904	8.25E-71	0.747994	-2.425	1.15E-51	0.698413	-2.267	1.57E-17	0.55
HOXA2	3199	-2.783	1.11E-48	0.765027	-3.0388	4.57E-72	0.792937	-2.76	2.86E-56	0.77381	-2.585	2.25E-20	0.7
STXBP6	29091	-2.615	1.45E-39	0.765027	-2.5767	1.63E-36	0.719101	-2.896	5.90E-51	0.789683	-2.358	2.07E-12	0.7
FOXP2	93986	-2.796	2.96E-37	0.765027	-2.8898	9.44E-50	0.800963	-2.734	2.09E-39	0.77381	-3.104	3.98E-14	0.8
TESC	54997	-2.461	2.31E-36	0.765027	-2.6247	1.09E-51	0.796148	-2.471	2.73E-35	0.793651	-2.394	1.25E-11	0.7
C2orf71	388939	-2.338	1.10E-35	0.765027	-2.3153	3.76E-42	0.75923	-2.59	6.40E-57	0.837302	-2.053	6.27E-11	0.7
PLCXD3	345557	-2.923	1.55E-32	0.765027	-3.7114	2.22E-53	0.823435	-3.126	8.72E-34	0.75	-3.746	1.35E-16	0.85
CAPN6	827	-3.435	3.04E-30	0.765027	-3.828	8.39E-34	0.781701	-3.821	1.26E-34	0.801587	-4.323	7.51E-18	0.8
CNTN6	27255	-2.155	4.30E-29	0.765027	-2.3395	1.85E-43	0.802568	-2.365	3.17E-37	0.833333	NA	NA	NA
PTX3	5806	-2.822	7.99E-27	0.765027	-2.992	7.44E-34	0.752809	-3.159	8.95E-37	0.777778	-3.673	9.28E-18	0.85

IRX1	79192	-2.855	1.21E-25	0.765027	-3.1917	6.97E-33	0.744783	-3.072	1.37E-26	0.746032	-3.061	4.49E-09	0.75
TUBB2B	347733	-2.567	1.54E-24	0.765027	-2.6979	2.53E-27	0.696629	-3.091	3.60E-34	0.742063	-2.789	3.59E-12	0.75
NR4A1	3164	-2.023	3.53E-22	0.765027	-2.0625	1.78E-33	0.741573	-2.063	4.86E-26	0.734127	-2.491	5.72E-08	0.8
DBC1	1620	-2.494	1.57E-15	0.765027	-3.2256	1.75E-32	0.800963	-2.968	5.68E-23	0.781746	-4.003	9.41E-10	0.8
SCN4B	6330	-2.94	1.56E-68	0.759563	-3.2715	4.38E-93	0.812199	-2.915	4.59E-63	0.75	-3.358	1.55E-37	0.8
ITPRIPL1	150771	-2.781	1.40E-53	0.759563	-2.9807	1.42E-82	0.792937	-2.893	1.33E-67	0.793651	-3.158	1.77E-25	0.75
ASAM	79827	-2.574	7.53E-42	0.759563	-2.6312	1.66E-61	0.776886	-2.508	2.52E-46	0.769841	-3.038	1.36E-17	0.9
ANK2	287	-2.369	1.66E-40	0.759563	-2.6277	3.44E-66	0.764045	-2.41	9.62E-46	0.746032	-2.666	1.36E-14	0.75
OXGR1	27199	-3.079	1.99E-31	0.759563	-2.8189	2.43E-27	0.714286	-3.075	2.66E-32	0.769841	-2.764	5.41E-09	0.7
LUZP2	338645	-2.717	3.04E-27	0.759563	-3.1731	6.56E-44	0.792937	-2.795	4.65E-26	0.738095	-3.309	2.68E-12	0.75
GLB1L3	112937	-2.025	2.77E-21	0.759563	-2.185	6.99E-38	0.786517	-2.152	4.52E-27	0.813492	-2.268	1.60E-07	0.75
TFPI2	7980	-2.436	3.07E-16	0.759563	-2.7772	3.73E-22	0.794543	-2.565	8.86E-19	0.742063	-3.91	1.75E-14	0.9
ELF5	2001	-2.877	5.03E-12	0.759563	-2.9326	1.66E-14	0.736758	-3.012	3.89E-16	0.833333	-2.477	0.006033	0.75
SRPX	8406	-2.816	7.21E-55	0.754098	-2.9528	1.37E-85	0.813804	-2.68	9.91E-55	0.761905	-2.876	1.26E-22	0.9
TMTC1	83857	-2.995	8.43E-48	0.754098	-3.1249	9.22E-68	0.778491	-2.845	9.17E-46	0.72619	-4.063	6.23E-38	0.9
MT1M	4499	-2.688	1.56E-39	0.754098	-2.9543	6.59E-57	0.773676	-2.693	2.65E-44	0.746032	-3.246	4.19E-25	0.9
KLF14	136259	-2.126	2.65E-34	0.754098	-2.098	5.06E-43	0.773676	-2.008	1.82E-30	0.757937	-2.37	2.19E-11	0.9
EMX2OS	196047	-2.461	7.92E-34	0.754098	-2.6109	3.85E-52	0.760835	-2.368	2.22E-38	0.730159	-2.342	3.09E-11	0.8
RGS2	5997	-2.273	6.12E-31	0.754098	-2.3498	1.95E-46	0.736758	-2.463	6.39E-44	0.801587	-2.89	8.99E-15	0.85
APOD	347	-2.986	2.56E-30	0.754098	-3.4521	1.02E-38	0.789727	-3.051	1.62E-32	0.757937	-4.187	1.95E-23	0.85
MRGPRX3	117195	-2.555	1.96E-19	0.754098	-3.0352	2.66E-41	0.834671	-2.916	6.44E-29	0.853175	-3.193	3.03E-07	0.8
DNER	92737	-2.529	1.24E-18	0.754098	-2.4678	5.83E-21	0.720706	-2.717	2.36E-26	0.765873	-2.239	1.46E-07	0.7
UGT8	7368	-2.366	5.41E-15	0.754098	-2.174	4.34E-14	0.701445	-2.805	4.07E-23	0.813492	-2.832	5.54E-06	0.75
SCRG1	11341	-2.259	1.68E-14	0.754098	-2.2364	4.50E-15	0.733547	-2.76	1.26E-25	0.793651	-2.85	1.84E-10	0.8
SLC6A15	55117	-2.053	5.36E-13	0.754098	NA	NA	NA	-2.594	1.58E-26	0.793651	-2.773	1.36E-09	0.8
CYP2A6	1548	-2.132	1.14E-07	0.754098	-2.7577	3.32E-13	0.789727	-2.667	2.21E-12	0.789683	-2.795	2.01E-06	0.7
C4orf7	260436	-2.265	9.74E-07	0.754098	-2.7379	3.95E-10	0.778491	-3.018	8.50E-12	0.797619	-3.801	7.39E-05	0.9
DST	667	-2.671	8.37E-59	0.748634	-2.7567	1.78E-92	0.807384	-2.699	1.30E-70	0.801587	-3.09	8.99E-24	0.8
AGAP11	119385	-2.53	8.20E-49	0.748634	-2.8016	3.86E-70	0.786517	-2.515	8.21E-49	0.730159	-3.117	2.94E-22	0.85
ADAMTS1	9510	-2.502	6.66E-47	0.748634	-2.6258	4.25E-67	0.739968	-2.605	1.86E-53	0.765873	-2.991	3.30E-23	0.85
CLIC5	53405	-2.227	1.64E-43	0.748634	-2.5316	7.60E-72	0.826645	-2.227	2.33E-43	0.706349	-2.964	8.76E-23	0.85
PDE11A	50940	-2.595	1.13E-37	0.748634	-3.0964	2.96E-57	0.826645	-3.007	2.39E-50	0.825397	-3.102	5.22E-18	0.75
VIP	7432	-2.072	5.03E-35	0.748634	-2.153	3.32E-64	0.76565	NA	NA	NA	NA	NA	NA
SLITRK2	84631	-2.02	3.50E-27	0.748634	NA	NA	NA	NA	NA	NA	-2.249	8.47E-08	0.85

C13orf33	84935	-2.108	3.08E-26	0.748634	-2.6063	7.65E-59	0.813804	-2.317	2.41E-40	0.781746	-2.816	7.28E-12	0.85
TCEAL2	140597	-2.321	1.00E-25	0.748634	-2.8113	1.03E-43	0.773676	-2.652	3.52E-30	0.710317	-3.095	5.70E-15	0.8
NPY5R	4889	-3	8.39E-22	0.748634	-3.3291	1.83E-28	0.767255	-3.626	8.37E-32	0.821429	-3.849	4.93E-14	0.9
HAP1	9001	-2.172	3.14E-18	0.748634	-2.1528	1.39E-25	0.744783	NA	NA	NA	NA	NA	NA
CCL13	6357	-2.397	5.78E-17	0.748634	-2.3781	2.01E-23	0.747994	-2.408	3.12E-22	0.81746	-2.738	3.45E-07	0.95
IGSF1	3547	-2.739	1.95E-15	0.748634	-3.056	6.31E-21	0.767255	-3.525	7.36E-26	0.797619	-3.242	5.28E-08	0.7
GPX2	2877	-2.352	2.19E-15	0.748634	-2.4628	2.17E-19	0.741573	NA	NA	NA	-2.343	0.000365	0.65
SPRY2	10253	-2.692	8.24E-77	0.743169	-2.9063	1.31E-109	0.802568	-2.912	1.38E-80	0.801587	-3.253	3.94E-46	0.85
FAM13C	220965	-2.649	1.37E-60	0.743169	-2.7881	1.91E-75	0.757624	-2.665	9.18E-51	0.714286	-2.85	7.44E-24	0.75
PRELP	5549	-2.408	2.36E-34	0.743169	-2.5669	1.87E-46	0.730337	-2.378	2.05E-37	0.65873	-2.428	1.06E-12	0.6
CADM3	57863	-3.035	6.19E-32	0.743169	-3.8182	7.17E-52	0.815409	-3.375	4.12E-37	0.777778	-3.948	1.69E-20	0.8
ATF3	467	-2.293	7.58E-31	0.743169	-2.398	1.27E-46	0.76886	-2.368	6.01E-38	0.75	-3.025	1.60E-13	0.9
FOS	2353	-2.438	1.19E-28	0.743169	-3.0628	5.45E-58	0.820225	-3.058	1.06E-43	0.801587	-4.173	2.52E-22	1
FGF9	2254	-2.306	1.98E-28	0.743169	-2.3477	2.72E-29	0.690209	-2.397	1.15E-32	0.686508	-3.028	7.83E-18	0.75
PPYR1	5540	-2.05	1.26E-27	0.743169	-2.1468	3.64E-38	0.76244	-2.275	3.94E-37	0.813492	-2.203	4.08E-11	0.8
CACNA2D1	781	-2.699	3.70E-25	0.743169	-2.8194	4.45E-32	0.715891	-2.674	4.99E-27	0.734127	-3.225	5.37E-11	0.8
TRIM29	23650	-2.468	1.19E-15	0.743169	-2.692	8.79E-19	0.685393	-2.936	1.58E-18	0.753968	-3.278	8.63E-06	0.7
CLCA2	9635	-2.7	4.01E-13	0.743169	-2.9341	2.66E-15	0.728732	-2.961	8.14E-16	0.75	-4.298	8.55E-12	0.8
HSD17B2	3294	-2.452	7.00E-13	0.743169	-2.6883	7.34E-20	0.725522	-2.837	2.55E-18	0.746032	-2.094	0.002353	0.7
MIA	8190	-2.44	7.30E-12	0.743169	-2.7067	8.63E-16	0.70947	-3.03	4.68E-19	0.769841	-3.705	1.08E-07	0.8
MUC6	4588	-2.517	3.22E-08	0.743169	-2.6663	1.79E-10	0.736758	-2.236	1.69E-06	0.678571	-2.269	0.002498	0.7
ANXA1	301	-2.501	2.46E-63	0.737705	-2.5939	1.70E-86	0.746388	-2.639	5.93E-73	0.789683	-2.883	5.00E-30	0.8
DLGAP2	9228	-2.5	3.70E-52	0.737705	-2.6941	8.04E-65	0.75923	-2.684	8.20E-59	0.75	-3.194	1.63E-29	0.85
BAI3	577	-2.522	1.28E-49	0.737705	-2.696	2.62E-60	0.780096	-2.63	4.37E-53	0.75	-3.327	1.27E-26	0.8
RBP7	116362	-2.434	2.58E-44	0.737705	-2.5769	1.08E-58	0.701445	-2.542	7.53E-48	0.710317	-2.652	2.68E-18	0.7
C10orf10	11067	-2.267	5.89E-42	0.737705	-2.376	4.23E-64	0.717496	-2.174	9.09E-41	0.68254	-2.242	1.32E-13	0.7
MGLL	11343	-2.176	1.53E-40	0.737705	-2.2528	9.32E-62	0.701445	NA	NA	NA	NA	NA	NA
P2RY12	64805	-2.633	1.14E-39	0.737705	-3.2573	3.37E-65	0.82183	-3.088	3.91E-49	0.813492	-3.699	7.17E-23	0.9
SOX5	6660	-2.375	1.04E-27	0.737705	-2.4883	1.32E-38	0.751204	-2.369	9.55E-36	0.777778	-3.29	6.20E-17	0.9
C5orf27	202299	-2.201	5.21E-21	0.737705	-2.221	3.22E-28	0.723917	-2.516	9.43E-37	0.781746	-2.299	2.13E-09	0.8
FGF10	2255	-2.601	1.94E-18	0.737705	-3.1883	7.24E-30	0.744783	-3.088	4.47E-28	0.765873	-3.737	4.30E-21	0.75
C1orf130	400746	-2.017	3.92E-12	0.737705	-2.1646	7.86E-18	0.699839	-2.438	2.29E-20	0.765873	-2.672	2.44E-05	0.75
SCN11A	11280	-2.396	4.46E-46	0.73224	-2.6044	5.56E-68	0.783307	-2.391	5.33E-47	0.718254	-3.037	1.44E-24	0.95
PLA2G4A	5321	-2.541	7.26E-45	0.73224	-2.558	6.53E-50	0.728732	-2.717	3.11E-65	0.793651	-3.372	8.43E-33	0.8

SLC10A6	345274	-2.339	6.28E-37	0.73224	-2.519	3.02E-53	0.76886	-2.524	4.83E-37	0.718254	-2.955	1.39E-17	0.75
FAT2	2196	-3.431	5.60E-33	0.73224	-3.7888	1.79E-44	0.784912	-3.651	1.36E-36	0.781746	-4.566	7.93E-18	0.8
DNAH9	1770	-2.271	1.23E-26	0.73224	-2.7032	2.05E-39	0.770465	-2.615	1.40E-36	0.730159	-2.896	1.87E-15	0.75
KCNH8	131096	-2.67	1.22E-22	0.73224	-3.0566	6.68E-39	0.789727	-3.289	1.41E-37	0.833333	-3.492	1.01E-09	0.8
C8orf47	203111	-2.099	1.61E-18	0.73224	NA	NA	NA	-2.297	2.95E-22	0.753968	-2.669	1.29E-09	0.8
ANKRD29	147463	-2.878	4.68E-57	0.726776	-3.0903	2.05E-69	0.757624	-2.985	1.92E-62	0.738095	-3.393	2.20E-30	0.75
TDRD10	126668	-2.553	6.31E-52	0.726776	-2.8058	1.73E-72	0.757624	-2.412	3.35E-45	0.68254	-2.633	3.96E-24	0.75
MYLK	4638	-2.458	2.51E-50	0.726776	-2.595	1.76E-71	0.756019	-2.511	2.11E-56	0.734127	-2.733	1.22E-18	0.85
CASP12	120329	-2.245	1.43E-47	0.726776	-2.3609	7.67E-79	0.794543	-2.007	2.49E-41	0.702381	-2.224	3.03E-14	0.7
CABP1	9478	-2.287	1.70E-41	0.726776	-2.3886	2.26E-67	0.76886	-2.155	1.80E-41	0.730159	NA	NA	NA
ID4	3400	-2.802	2.34E-39	0.726776	-3.1842	7.07E-54	0.754414	-3.14	6.48E-49	0.769841	-3.239	3.02E-20	0.7
CCDC85A	114800	-2.292	7.76E-39	0.726776	-2.6569	1.38E-57	0.764045	-2.538	5.90E-48	0.753968	-3.171	2.03E-23	0.9
HFM1	164045	-2	9.93E-31	0.726776	-2.2125	6.59E-49	0.756019	-2.206	1.28E-38	0.753968	-2.996	9.53E-19	0.9
KCNMB2	10242	-2.181	1.51E-30	0.726776	-2.2945	1.08E-46	0.780096	-2.166	9.72E-31	0.738095	-2.255	2.11E-09	0.75
SNCG	6623	-2.478	2.42E-30	0.726776	-2.8181	9.40E-38	0.739968	-2.159	1.38E-20	0.642857	-2.138	2.60E-08	0.6
PREX2	80243	-2.694	5.46E-30	0.726776	-3.2459	5.39E-50	0.810594	-2.774	9.00E-34	0.77381	-3.808	1.53E-17	0.9
RAB3C	115827	-2.191	4.52E-28	0.726776	-2.3101	3.81E-32	0.743178	-2.17	8.55E-24	0.686508	-2.769	9.17E-12	0.95
PDE8B	8622	-2.341	4.45E-27	0.726776	-2.5438	6.24E-35	0.696629	-2.418	1.35E-28	0.698413	-2.628	3.58E-11	0.7
MRC1	4360	-2.3	1.72E-25	0.726776	-2.3571	2.17E-35	0.725522	-2.209	3.15E-27	0.694444	-2.449	1.46E-08	0.85
EPHA7	2045	-2.243	1.34E-21	0.726776	NA	NA	NA	-2.023	4.99E-19	0.654762	NA	NA	NA
STON1-GTF2A	286749	-2.025	3.35E-11	0.726776	-2.1009	4.09E-18	0.720706	-2.139	1.11E-14	0.734127	NA	NA	NA
KRT17	3872	-2.338	1.13E-08	0.726776	-2.7519	3.37E-14	0.720706	-2.896	7.08E-12	0.769841	-3.511	0.000387	0.8
HMGCS2	3158	-2.611	2.52E-07	0.726776	-3.4769	1.78E-14	0.760835	-2.63	1.63E-08	0.75	-3.93	8.36E-05	0.9
APCDD1	147495	-2.448	1.82E-63	0.721311	-2.537	5.46E-76	0.714286	-2.479	1.92E-57	0.702381	-2.453	2.97E-24	0.65
ALDOC	230	-2.554	1.83E-40	0.721311	-2.4135	2.32E-45	0.686998	-2.345	1.17E-39	0.670635	NA	NA	NA
FLJ42875	440556	-2.319	5.26E-40	0.721311	-2.4934	5.40E-61	0.76244	-2.174	1.05E-36	0.670635	-2.984	9.10E-23	0.85
TBX15	6913	-2.005	7.33E-39	0.721311	-2.1946	1.36E-61	0.733547	-2.034	2.98E-44	0.710317	NA	NA	NA
TRPM6	140803	-2.717	9.24E-36	0.721311	-3.1145	9.07E-53	0.751204	-2.959	3.74E-42	0.72619	-3.89	3.27E-26	0.8
PTH1R	5745	-2.546	8.23E-35	0.721311	-2.8167	5.42E-55	0.781701	-2.497	1.13E-39	0.734127	-2.094	8.89E-10	0.65
DOCK11	139818	-2.126	7.68E-33	0.721311	-2.1337	4.01E-54	0.751204	-2.05	2.12E-39	0.761905	-2.28	1.01E-09	0.75
ANGPTL4	51129	-2.491	9.33E-30	0.721311	-2.5692	8.16E-38	0.715891	-2.581	2.67E-31	0.690476	-2.157	7.08E-09	0.55
MAPK4	5596	-2.717	1.97E-21	0.721311	-2.9447	4.92E-23	0.720706	-3.365	8.88E-35	0.801587	-3.549	3.97E-15	0.7
KRT15	3866	-2.083	3.05E-07	0.721311	-2.4301	6.07E-14	0.741573	-2.643	2.11E-12	0.769841	-3.984	0.000117	0.85
NR3C2	4306	-2.77	2.68E-63	0.715847	-2.793	1.67E-73	0.720706	-2.597	7.85E-64	0.714286	-3.011	2.19E-31	0.75

TSPAN7	7102	-2.563	7.57E-56	0.715847	-3.0803	1.25E-80	0.804173	-2.891	1.21E-57	0.746032	-3.505	8.17E-33	0.85
SCN3B	55800	-2.749	2.64E-50	0.715847	-3.2695	1.76E-68	0.815409	-2.764	2.68E-46	0.702381	-3.366	5.14E-27	0.75
FAM149A	25854	-2.313	1.23E-48	0.715847	-2.5175	3.41E-62	0.707865	-2.479	6.29E-51	0.710317	-2.937	3.27E-22	0.8
CLDN11	5010	-2.807	1.76E-44	0.715847	-3.4048	1.16E-76	0.817014	-2.919	4.42E-46	0.702381	-3.72	9.61E-25	0.8
SLC25A27	9481	-2.746	3.26E-44	0.715847	-2.7894	3.41E-59	0.704655	-2.745	8.05E-46	0.694444	-2.829	2.15E-22	0.7
DHDPSL	112817	-2.602	1.46E-30	0.715847	-2.558	1.45E-34	0.685393	-2.531	8.09E-34	0.714286	-2.46	4.88E-13	0.65
PKNOX2	63876	-2.247	5.30E-30	0.715847	-2.3972	9.37E-36	0.698234	-2.246	1.28E-29	0.722222	-2.776	5.69E-18	0.8
EGR3	1960	-2.372	9.24E-26	0.715847	-2.7197	2.57E-41	0.757624	-2.542	7.32E-32	0.730159	-4.044	2.45E-18	0.95
SMOC1	64093	-2.615	1.11E-24	0.715847	-2.551	3.85E-21	0.674157	-2.924	1.04E-32	0.710317	-2.747	3.48E-14	0.7
GPR88	54112	-2.059	1.06E-21	0.715847	-2.3017	1.28E-30	0.772071	-2.095	6.73E-27	0.746032	-2.536	4.91E-11	0.85
GPM6A	2823	-2.523	7.22E-21	0.715847	-3.0622	2.33E-31	0.794543	-3.062	1.03E-31	0.753968	-3.547	6.78E-14	0.8
LGR6	59352	-2.526	6.52E-17	0.715847	-2.8345	3.84E-24	0.739968	-2.685	2.20E-19	0.706349	-3.622	2.57E-09	0.8
PPP1R12B	4660	-2.192	1.06E-78	0.710383	-2.2857	3.19E-115	0.722311	-2.166	2.31E-77	0.670635	-2.301	2.29E-27	0.65
CORO2B	10391	-2.507	1.01E-60	0.710383	-2.837	3.26E-87	0.776886	-2.422	3.75E-57	0.698413	-2.91	1.04E-28	0.8
PER1	5187	-2.183	2.68E-46	0.710383	-2.2971	1.05E-75	0.725522	-2.181	8.21E-49	0.674603	-2.261	3.64E-18	0.8
NCRNA00086	399668	-2.561	4.05E-43	0.710383	-2.7946	1.01E-58	0.76565	-2.825	1.35E-43	0.718254	-2.557	6.59E-14	0.7
ASXL3	80816	-2.634	5.16E-42	0.710383	-2.7243	1.21E-46	0.711075	-2.635	3.02E-44	0.738095	-2.851	1.67E-16	0.8
EGR2	1959	-2.239	1.08E-30	0.710383	-2.4625	4.98E-55	0.770465	-2.39	2.43E-39	0.781746	-3.043	3.68E-14	0.9
ADAMTS18	170692	-2.462	2.96E-24	0.710383	-2.97	1.73E-36	0.75923	-2.684	5.08E-28	0.72619	-3.575	9.67E-17	0.85
CCL21	6366	-3.131	3.24E-24	0.710383	-4.0901	4.07E-38	0.808989	-3.375	3.83E-28	0.742063	-4.999	2.90E-17	0.85
NPM2	10361	-2.069	1.63E-20	0.710383	NA	NA	NA	NA	NA	NA	NA	NA	NA
LRP1B	53353	-2.351	1.24E-12	0.710383	-2.5863	1.57E-16	0.711075	-2.477	6.69E-14	0.706349	-2.75	1.20E-08	0.7
SLC34A2	10568	-2.532	1.12E-09	0.710383	-2.7007	1.11E-12	0.704655	-3.046	1.30E-13	0.753968	-3.102	0.001307	0.65
GPM6B	2824	-2.23	3.95E-36	0.704918	-2.2555	4.29E-44	0.707865	-2.41	1.45E-40	0.706349	-2.368	1.92E-10	0.7
FAM189A2	9413	-2.508	1.91E-34	0.704918	-2.9388	3.99E-41	0.699839	-2.833	2.98E-33	0.694444	-3.349	1.90E-14	0.7
PACRG	135138	-2.187	7.67E-32	0.704918	-2.4718	8.34E-47	0.711075	-2.281	2.56E-32	0.650794	-2.773	2.30E-14	0.85
WNT11	7481	-2.355	5.19E-26	0.704918	-2.4318	1.76E-28	0.70626	-2.434	4.49E-28	0.710317	NA	NA	NA
HRASLS5	117245	-2.751	1.08E-24	0.704918	-3.5466	4.68E-37	0.770465	-3.065	2.07E-29	0.746032	-3.778	1.55E-16	0.8
HRCT1	646962	-2.379	1.51E-19	0.704918	-2.4626	6.45E-27	0.739968	-2.707	5.94E-30	0.742063	-2.776	2.39E-12	0.85
SLC28A3	64078	-2.599	1.96E-13	0.704918	-2.8232	8.18E-20	0.728732	-3.393	9.79E-25	0.809524	-4.342	1.44E-09	0.95
SULT1C3	442038	NA	NA	NA	-2.1162	3.60E-28	0.991974	NA	NA	NA	NA	NA	NA
MYOZ2	51778	NA	NA	NA	-2.1139	8.99E-47	0.987159	-2.047	2.63E-33	0.97619	NA	NA	NA
HBG1	3047	NA	NA	NA	-2.1468	1.10E-58	0.983949	NA	NA	NA	NA	NA	NA
ALAS2	212	NA	NA	NA	-2.1328	6.87E-58	0.982343	NA	NA	NA	NA	NA	NA

SRL	6345	NA	NA	NA	-2.2124	2.82E-53	0.982343	NA	NA	NA	-2.36	2.18E-13	1
KLK3	354	NA	NA	NA	-2.5396	8.75E-25	0.932584	-2.335	4.52E-16	0.93254	-2.084	0.007105	0.8
MUC7	4589	NA	NA	NA	-2.096	8.03E-15	0.906902	-2.03	5.36E-10	0.873016	-2.511	0.002153	0.95
PMP2	5375	NA	NA	NA	-2.0986	5.31E-39	0.889246	-2.152	2.26E-33	0.869048	-2.713	1.87E-11	1
NXNL1	115861	NA	NA	NA	-2.0242	1.17E-68	0.879615	NA	NA	NA	NA	NA	NA
HRC	3270	NA	NA	NA	-2.0333	9.98E-36	0.861958	NA	NA	NA	NA	NA	NA
SPATA22	84690	NA	NA	NA	-2.0945	3.07E-36	0.857143	-2.131	5.44E-30	0.84127	NA	NA	NA
FCER2	2208	NA	NA	NA	-2.4782	4.12E-23	0.820225	-2.385	4.64E-19	0.809524	-3.097	3.78E-08	0.9
RSPO1	284654	NA	NA	NA	-2.3313	1.27E-22	0.820225	-2.12	3.89E-16	0.813492	-2.801	1.43E-05	0.95
C2CD4B	388125	NA	NA	NA	-2.1152	9.24E-25	0.808989	NA	NA	NA	NA	NA	NA
CUX2	23316	NA	NA	NA	-2.1338	4.51E-18	0.808989	-2.134	7.69E-15	0.821429	-2.512	2.60E-05	0.85
KIF25	3834	NA	NA	NA	-2.0589	1.43E-31	0.799358	-2.099	2.16E-26	0.797619	-2.619	9.28E-09	0.8
MYL7	58498	NA	NA	NA	-2.404	1.66E-22	0.799358	-2.376	7.99E-17	0.809524	-2.76	3.25E-05	0.9
MFAP4	4239	-2.587	3.99E-32	0.644809	-3.2331	8.54E-54	0.797753	-2.667	1.01E-35	0.702381	-3.376	3.94E-18	0.7
DTX1	1840	-2.333	4.33E-38	0.699454	-2.5011	1.73E-67	0.792937	-2.414	1.23E-47	0.75	-2.36	2.58E-12	0.75
NR4A3	8013	NA	NA	NA	-2.0181	3.10E-29	0.792937	-2.1	1.16E-23	0.825397	-2.568	4.45E-07	0.8
NCRNA00110	642976	NA	NA	NA	-2.4357	6.50E-28	0.791332	-2.419	4.84E-22	0.781746	-2.707	1.84E-06	0.8
LRFN5	145581	-2.53	5.43E-40	0.688525	-3.0006	2.65E-58	0.789727	-2.697	7.40E-43	0.718254	-3.003	3.42E-18	0.7
GPR182	11318	NA	NA	NA	-2.1124	5.50E-58	0.789727	NA	NA	NA	-2.352	1.04E-09	0.85
C21orf34	388815	-2.827	1.22E-55	0.699454	-3.1199	5.66E-81	0.781701	-2.819	7.62E-57	0.730159	-3.27	3.20E-31	0.85
SLCO1C1	53919	NA	NA	NA	-2.1405	8.60E-52	0.781701	NA	NA	NA	-2.291	9.21E-10	0.8
EFHA2	286097	-2.544	2.49E-54	0.677596	-2.8446	2.09E-76	0.780096	-2.703	3.13E-59	0.730159	-3.136	7.74E-24	0.7
DARC	2532	-2.936	3.34E-31	0.68306	-4.1438	7.69E-45	0.780096	-3.283	1.56E-29	0.734127	-4.168	4.82E-24	0.85
ITM2A	9452	-2.584	1.59E-52	0.68306	-3.0084	1.45E-79	0.773676	-2.749	8.60E-56	0.68254	-3.059	9.56E-36	0.8
KCNC2	3747	NA	NA	NA	-2.4996	8.80E-11	0.772071	-2.459	8.30E-10	0.769841	-2.097	0.009739	0.7
RASL10A	10633	NA	NA	NA	-2.0033	1.45E-37	0.770465	NA	NA	NA	NA	NA	NA
CCBE1	147372	-2.329	6.08E-30	0.699454	-2.3781	5.67E-44	0.76886	-2.357	1.19E-32	0.714286	-2.085	1.72E-07	0.55
GREM2	64388	-2.426	1.47E-29	0.699454	-3.0322	3.02E-48	0.767255	-2.444	1.97E-30	0.623016	-2.992	9.30E-14	0.85
TCEAL7	56849	-2.454	2.27E-53	0.677596	-2.7203	1.43E-84	0.76565	-2.413	2.13E-55	0.662698	-2.715	1.60E-24	0.8
SEMA3D	223117	-2.828	1.17E-35	0.672131	-3.1844	5.21E-55	0.76565	-3.199	3.39E-49	0.797619	-3.358	2.94E-19	0.8
PRKAR2B	5577	NA	NA	NA	-2.1151	2.89E-41	0.76565	-2.06	1.87E-30	0.77381	-2.219	1.04E-07	0.9
CRB2	286204	NA	NA	NA	-2.4031	6.58E-41	0.76244	-2.242	1.16E-27	0.753968	-2.799	1.69E-08	0.9
ZFP36	7538	NA	NA	NA	-2.3122	5.45E-61	0.760835	-2.169	3.91E-40	0.722222	-2.932	9.52E-17	0.85
NPAS4	266743	NA	NA	NA	-2.0179	1.67E-42	0.75923	-2.182	6.91E-45	0.81746	-2.374	4.14E-11	0.85

GRRP1	79927	-2.027	2.10E-37	0.622951	-2.4807	3.67E-69	0.756019	-2.119	1.32E-35	0.619048	-2.095	2.65E-13	0.7
EDNRB	1910	-2.361	1.37E-57	0.655738	-2.6632	4.83E-79	0.754414	-2.502	3.61E-55	0.690476	-2.829	1.10E-32	0.9
CDH12	1010	NA	NA	NA	-2.0013	3.22E-34	0.754414	NA	NA	NA	NA	NA	NA
C7	730	-2.671	7.84E-19	0.644809	-3.9941	7.95E-33	0.754414	-3.15	1.68E-22	0.68254	-4.065	1.60E-15	0.8
SOX17	64321	NA	NA	NA	-2.2884	8.64E-72	0.752809	NA	NA	NA	-2.265	5.36E-14	0.75
IGF1	3479	-2.5	2.19E-34	0.677596	-3.0803	3.28E-53	0.752809	-2.592	2.06E-38	0.690476	-3.84	1.79E-24	0.9
GPR97	222487	-2.175	1.24E-24	0.699454	-2.3835	6.76E-44	0.752809	-2.322	5.89E-31	0.734127	NA	NA	NA
LCN10	414332	-2.307	2.50E-25	0.63388	-2.7943	3.88E-47	0.751204	-2.412	6.89E-29	0.65873	-2.972	8.78E-18	0.8
RIMBP2	23504	-2.276	4.77E-27	0.68306	-2.6164	1.13E-42	0.749599	-2.53	2.85E-40	0.68254	-2.842	1.49E-18	0.7
COL4A6	1288	-2.441	8.82E-22	0.677596	-2.998	2.06E-31	0.749599	-2.939	5.11E-30	0.746032	-3.651	1.51E-13	0.75
FCN2	2220	NA	NA	NA	-2.1822	8.71E-22	0.749599	NA	NA	NA	NA	NA	NA
RBMS3	27303	-2.577	3.06E-47	0.677596	-2.7844	1.34E-71	0.747994	-2.519	4.51E-56	0.734127	-2.91	2.34E-23	0.85
ABCB1	5243	-2.302	8.23E-37	0.63388	-2.8656	3.10E-59	0.747994	-2.477	2.73E-37	0.654762	-3.136	1.29E-19	0.8
ISM1	140862	-2.472	2.06E-47	0.672131	-2.8698	1.04E-64	0.746388	-2.553	1.30E-44	0.678571	-3.009	1.74E-20	0.75
KLHL4	56062	NA	NA	NA	-2.2231	5.66E-46	0.744783	-2.005	1.04E-31	0.68254	-2.219	1.11E-08	0.85
DBX2	440097	-2.375	1.67E-36	0.655738	-2.7452	1.06E-55	0.743178	-2.349	5.49E-36	0.650794	-3.028	7.81E-19	0.75
TGFBR2	7048	-2.244	5.26E-58	0.655738	-2.4958	3.33E-96	0.741573	-2.268	1.25E-61	0.654762	-2.66	4.11E-26	0.8
SEMA6D	80031	-2.279	1.53E-40	0.644809	-2.708	2.94E-54	0.741573	-2.547	2.46E-48	0.714286	-2.97	2.52E-22	0.8
TWIST2	117581	-2.119	5.26E-27	0.63388	-2.3966	3.57E-48	0.741573	-2.088	1.80E-30	0.666667	-2.297	1.16E-10	0.7
ABI3BP	25890	-2.476	1.10E-29	0.666667	-2.9172	5.75E-45	0.741573	-2.517	8.47E-35	0.686508	-3.477	8.92E-19	0.8
PM20D1	148811	NA	NA	NA	-2.0072	2.55E-41	0.738363	NA	NA	NA	-2.605	1.51E-09	0.8
ST8SIA2	8128	NA	NA	NA	-2.0937	3.14E-20	0.738363	NA	NA	NA	NA	NA	NA
HP	3240	NA	NA	NA	-2.0608	7.34E-13	0.738363	NA	NA	NA	NA	NA	NA
TMEM220	388335	-2.388	7.30E-71	0.672131	-2.6639	4.27E-101	0.736758	-2.686	2.42E-84	0.77381	-2.926	2.98E-37	0.8
FAM70A	55026	-2.639	1.36E-45	0.699454	-2.7892	2.64E-68	0.736758	-2.543	1.97E-52	0.734127	-2.797	4.30E-20	0.7
VWCE	220001	-2.175	2.59E-39	0.677596	-2.3707	1.85E-67	0.736758	-2.202	1.07E-42	0.662698	-2.045	9.56E-12	0.75
SLC47A2	146802	NA	NA	NA	-2.1043	4.81E-50	0.736758	-2.03	1.63E-40	0.694444	-2.262	1.10E-16	0.8
CALCR	799	NA	NA	NA	-2.1177	9.88E-35	0.736758	NA	NA	NA	NA	NA	NA
SCN7A	6332	-2.675	9.04E-25	0.677596	-3.3781	7.72E-38	0.735152	-2.72	6.51E-26	0.678571	-4.092	2.26E-18	0.85
PDLIM3	27295	NA	NA	NA	-2.1172	3.58E-47	0.733547	-2.031	2.71E-38	0.746032	NA	NA	NA
CYP26B1	56603	NA	NA	NA	-2.12	5.01E-40	0.731942	-2.366	1.88E-38	0.793651	NA	NA	NA
CALN1	83698	NA	NA	NA	-2.5209	3.14E-36	0.731942	-2.425	1.23E-31	0.72619	-2.806	1.32E-13	0.8
IGJ	3512	-2.557	9.89E-15	0.68306	-2.8182	4.13E-22	0.731942	-2.5	1.37E-17	0.678571	-3.8	3.54E-08	0.75
FAM162B	221303	-2.313	5.69E-50	0.672131	-2.5892	2.07E-78	0.730337	-2.3	3.16E-48	0.623016	-2.341	6.79E-20	0.7

WDR86	349136	-2.483	1.62E-31	0.672131	-2.664	2.39E-49	0.730337	-2.292	1.25E-26	0.65873	-2.386	3.61E-10	0.7
KCNA6	3742	-2.25	8.15E-33	0.666667	-2.6246	1.39E-48	0.730337	-2.474	2.06E-39	0.694444	-2.792	1.68E-17	0.7
CTSG	1511	-2.417	2.76E-19	0.655738	-2.9139	1.51E-32	0.730337	-2.437	8.35E-22	0.674603	-3.482	3.98E-13	0.85
TCN1	6947	NA	NA	NA	-2.3553	9.06E-10	0.730337	-2.768	3.49E-12	0.793651	-3.677	1.21E-05	0.8
CXCR2	3579	NA	NA	NA	-2.0092	6.71E-48	0.728732	NA	NA	NA	-2.082	1.40E-11	0.85
HRNR	388697	-2.026	1.68E-29	0.661202	-2.2976	1.04E-43	0.728732	-2.068	2.84E-29	0.638889	-2.768	6.99E-13	0.75
ZNF676	163223	-2.366	5.32E-22	0.688525	-2.7279	5.80E-34	0.728732	-2.46	1.10E-29	0.706349	-3.198	1.39E-19	0.75
CDH23	64072	-2.178	7.43E-37	0.644809	-2.5237	1.29E-56	0.727127	-2.183	6.41E-38	0.650794	-2.255	4.00E-12	0.7
SV2B	9899	-2.505	4.02E-38	0.68306	-2.6973	3.44E-48	0.727127	-2.39	6.46E-38	0.642857	-2.62	1.73E-17	0.75
GPRASP1	9737	-2.342	2.76E-65	0.693989	-2.554	1.48E-88	0.725522	-2.369	1.04E-60	0.650794	-2.608	9.62E-25	0.75
GPR109B	8843	-2.188	6.87E-41	0.688525	-2.3848	5.29E-60	0.725522	-2.241	2.98E-44	0.65873	-2.641	4.75E-17	0.8
LOC643763	643763	NA	NA	NA	-2.1828	1.50E-29	0.725522	-2.27	8.88E-30	0.718254	-2.245	6.80E-09	0.75
C5orf4	10826	-2.642	1.80E-47	0.699454	-2.883	1.04E-63	0.722311	-2.669	3.64E-53	0.662698	-3.44	1.04E-34	0.8
GRM7	2917	NA	NA	NA	-2.1866	7.47E-41	0.722311	NA	NA	NA	-2.537	4.94E-16	0.85
CPB1	1360	NA	NA	NA	-2.1537	5.70E-05	0.722311	NA	NA	NA	NA	NA	NA
IGFN1	91156	NA	NA	NA	-2.0558	5.89E-26	0.720706	NA	NA	NA	-2.516	6.47E-10	0.85
CSMD1	64478	NA	NA	NA	-2.306	1.01E-21	0.720706	-2.299	1.91E-20	0.690476	NA	NA	NA
AQP1	358	-2.28	7.57E-62	0.63388	-2.549	6.03E-86	0.719101	-2.268	2.53E-57	0.650794	-2.344	6.22E-27	0.75
SHE	126669	-2.171	1.10E-53	0.628415	-2.4358	2.45E-82	0.719101	-2.095	2.75E-51	0.599206	-2.499	2.16E-22	0.75
CREB5	9586	-2.229	5.66E-49	0.661202	-2.4804	1.63E-74	0.719101	-2.474	3.73E-57	0.72619	-2.639	3.15E-20	0.7
C11orf53	341032	NA	NA	NA	-2.1558	6.93E-35	0.719101	NA	NA	NA	-2.369	3.06E-09	0.75
WISP3	8838	-2.318	3.81E-16	0.677596	-2.6163	6.69E-24	0.719101	-2.515	1.45E-20	0.738095	-2.002	0.000126	0.65
LDB2	9079	-2.192	2.71E-68	0.655738	-2.4026	9.19E-104	0.717496	-2.111	3.23E-62	0.59127	-2.35	3.66E-26	0.8
PEAR1	375033	-2.267	5.95E-57	0.661202	-2.4627	1.07E-82	0.717496	-2.172	4.85E-54	0.626984	-2.424	3.46E-25	0.7
CNR1	1268	-2.3	7.06E-27	0.639344	-2.7186	4.29E-33	0.717496	-2.613	1.02E-28	0.698413	-2.807	1.57E-11	0.75
SYT8	90019	-2.041	1.07E-08	0.68306	-2.4315	3.72E-15	0.717496	-2.537	1.41E-12	0.730159	-2.947	0.000149	0.75
GNG11	2791	-2.082	1.22E-56	0.677596	-2.2683	7.31E-97	0.715891	-2.013	1.43E-59	0.607143	-2.083	1.70E-18	0.7
PIP	5304	NA	NA	NA	-2.5829	6.19E-08	0.715891	-2.458	6.77E-07	0.761905	NA	NA	NA
HYAL1	3373	-2.021	1.74E-43	0.595628	-2.287	4.64E-68	0.714286	NA	NA	NA	NA	NA	NA
AQP5	362	NA	NA	NA	-2.3267	1.76E-10	0.714286	-2.907	9.35E-15	0.777778	-2.814	0.000141	0.8
LRRC70	1E+08	-2.053	1.00E-52	0.639344	-2.3539	9.10E-82	0.712681	-2.081	2.64E-49	0.615079	-2.561	1.51E-22	0.8
THSD7B	80731	-2.546	3.82E-35	0.693989	-2.9372	1.16E-47	0.712681	-2.606	1.67E-32	0.638889	-3.573	4.29E-24	0.85
GAS2	2620	NA	NA	NA	-2.1543	1.42E-43	0.712681	-2.282	2.32E-42	0.738095	-2.393	7.52E-12	0.6
C9orf125	84302	-2.288	4.71E-60	0.655738	-2.3926	1.96E-76	0.711075	-2.267	6.78E-63	0.65873	-2.445	4.40E-28	0.9

TFPI	7035	-2.309	2.02E-52	0.68306	-2.3915	3.40E-70	0.711075	-2.16	1.34E-47	0.630952	NA	NA	NA
BMP6	654	-2.116	1.98E-41	0.650273	-2.3488	5.75E-58	0.711075	-2.212	1.69E-44	0.65873	-2.568	3.67E-18	0.8
C8orf79	57604	-2.308	5.14E-33	0.590164	-2.9519	3.32E-47	0.711075	-2.774	1.98E-34	0.646825	-3.523	4.49E-20	0.75
NGFR	4804	-2.496	5.19E-29	0.63388	-2.9866	1.37E-42	0.711075	-2.647	2.72E-28	0.654762	-3.339	6.08E-16	0.8
ODZ2	57451	-2.401	6.11E-19	0.655738	-2.7471	5.48E-30	0.711075	-2.514	2.62E-22	0.72619	-3.079	7.07E-08	0.85
ADAMTS8	11095	NA	NA	NA	-2.1218	7.94E-24	0.70947	NA	NA	NA	-3.155	3.24E-11	0.85
PALM2-AKAP2	445815	-2.047	5.14E-55	0.644809	-2.1713	4.78E-96	0.707865	-2.154	1.90E-65	0.690476	-2.3	3.94E-19	0.65
RASSF9	9182	-2.354	5.63E-49	0.63388	-2.6972	5.94E-63	0.707865	-2.643	1.52E-56	0.75	-3.35	9.52E-32	0.85
KCNIP1	30820	NA	NA	NA	-2.1467	4.58E-39	0.707865	-2.103	2.99E-30	0.650794	-2.674	9.87E-13	0.8
C16orf89	146556	-2.442	1.67E-17	0.606557	-3.4513	3.88E-32	0.707865	-2.64	3.31E-20	0.595238	-3.978	6.60E-25	0.8
NTF3	4908	NA	NA	NA	-2.3332	4.66E-31	0.70626	-2.483	2.99E-33	0.72619	-2.296	1.54E-10	0.75
AGTR1	185	NA	NA	NA	-2.4664	1.43E-17	0.70626	-2.265	9.28E-18	0.678571	-2.166	8.08E-07	0.55
CCDC36	339834	-2.206	4.73E-39	0.693989	-2.1997	3.16E-62	0.704655	NA	NA	NA	-2.049	9.49E-12	0.8
LOC285419	285419	NA	NA	NA	-2.0424	8.23E-49	0.70305	NA	NA	NA	-2.172	2.49E-17	0.7
MAPK10	5602	-2.237	1.70E-26	0.655738	-2.4228	3.55E-35	0.70305	-2.431	9.11E-34	0.734127	NA	NA	NA
BANK1	55024	NA	NA	NA	-2.1287	3.50E-22	0.70305	-2.134	1.41E-21	0.710317	-2.577	3.48E-08	0.85
ATP13A4	84239	NA	NA	NA	-2.1748	3.70E-14	0.70305	-2.213	3.27E-11	0.710317	-2.367	0.001022	0.6
KCNAB1	7881	-2.014	3.38E-50	0.639344	-2.0984	2.09E-68	0.701445	-2.128	2.22E-58	0.666667	-2.478	1.23E-22	0.9
ALDH2	217	NA	NA	NA	-2.046	1.09E-48	0.701445	NA	NA	NA	-2.071	6.29E-09	0.8
PRSS12	8492	-2.758	7.20E-28	0.688525	-2.8589	3.16E-31	0.701445	-3.159	1.57E-44	0.761905	-3.064	1.49E-14	0.75
C6orf142	90523	NA	NA	NA	NA	NA	NA	-2.025	1.94E-28	0.984127	-2.178	2.10E-07	0.95
GDA	9615	NA	NA	NA	NA	NA	NA	-2.01	9.98E-26	0.960317	-2.122	4.08E-05	1
KRT79	338785	NA	NA	NA	NA	NA	NA	-2.095	7.46E-18	0.876984	-2.424	3.21E-05	0.9
MATN4	8785	NA	NA	NA	NA	NA	NA	-2.119	3.88E-26	0.825397	NA	NA	NA
UTS2D	257313	NA	NA	NA	NA	NA	NA	-2.032	5.98E-43	0.813492	-2.197	1.11E-10	0.95
ABCA13	154664	NA	NA	NA	NA	NA	NA	-2.177	1.97E-14	0.809524	-2.206	0.000355	0.8
CXCL6	6372	NA	NA	NA	NA	NA	NA	-2.139	5.47E-21	0.777778	-2.254	1.41E-06	0.75
SOX6	55553	-2.017	2.55E-20	0.68306	-2.18	1.88E-25	0.669342	-2.637	2.87E-36	0.765873	-2.968	1.07E-22	0.9
C6orf15	29113	NA	NA	NA	NA	NA	NA	-2.043	2.20E-15	0.765873	-2.11	0.000185	0.65
XDH	7498	NA	NA	NA	NA	NA	NA	-2.117	1.34E-14	0.765873	NA	NA	NA
DAPL1	92196	NA	NA	NA	NA	NA	NA	-2.297	1.27E-18	0.757937	-3.219	1.22E-08	0.85
TMEM71	137835	NA	NA	NA	-2.2472	8.47E-37	0.680578	-2.297	2.79E-40	0.753968	-2.966	1.53E-18	0.85
ANXA8L2	244	-2.206	1.30E-11	0.677596	-2.5123	5.00E-16	0.688604	-2.669	5.68E-17	0.746032	-3.634	6.04E-08	0.8
KCTD14	65987	-2.245	1.68E-24	0.693989	-2.5266	1.53E-36	0.698234	-2.587	2.32E-33	0.738095	-2.785	3.05E-11	0.7

AMY1A	276	NA	NA	NA	NA	NA	NA	-2.118	4.37E-19	0.734127	NA	NA	NA
SERPINB5	5268	-2.063	1.09E-08	0.672131	-2.2019	2.43E-11	0.65008	-2.8	1.81E-14	0.734127	-3.254	0.000205	0.7
PKDCC	91461	NA	NA	NA	NA	NA	NA	-2.116	1.48E-36	0.730159	-2.169	1.40E-08	0.8
DSG3	1830	-2.421	1.03E-09	0.688525	-2.7345	8.24E-13	0.690209	-2.976	5.43E-14	0.730159	-4.064	4.24E-06	0.75
RIC3	79608	-2.531	1.95E-24	0.562842	-3.1269	1.92E-37	0.675762	-3.177	9.15E-37	0.72619	-3.671	1.14E-16	0.6
COL4A3	1285	NA	NA	NA	-2.0831	8.26E-24	0.698234	-2.159	2.96E-21	0.72619	-2.487	5.85E-07	0.75
MAML2	84441	-2.49	1.76E-44	0.68306	-2.4423	7.67E-59	0.677368	-2.539	1.97E-52	0.722222	-2.998	1.97E-23	0.85
KLK11	11012	-2.471	1.92E-09	0.688525	-2.7003	3.55E-12	0.683788	-3.119	3.50E-13	0.722222	-3.487	8.52E-06	0.65
STAC	6769	-2.269	3.50E-28	0.644809	-2.2384	7.51E-27	0.638844	-2.621	1.88E-38	0.718254	-2.913	4.28E-19	0.75
SLC26A4	5172	-2.08	1.59E-33	0.677596	-2.03	3.79E-32	0.634029	-2.129	5.00E-31	0.718254	-2.039	2.58E-12	0.65
SOX8	30812	NA	NA	NA	NA	NA	NA	-2.167	1.90E-22	0.718254	-2.242	1.12E-07	0.75
DSC3	1825	-2.348	1.11E-11	0.644809	-2.7391	1.78E-16	0.659711	-2.852	5.12E-17	0.718254	-4.181	9.29E-08	0.7
KIAA1045	23349	-2.116	4.01E-26	0.639344	-2.3491	1.97E-34	0.683788	-2.532	5.98E-39	0.714286	-3.072	4.48E-23	0.85
GDF5	8200	-2.081	6.36E-22	0.655738	-2.2866	4.92E-23	0.667737	-2.641	1.93E-34	0.714286	-2.239	8.55E-11	0.6
PTH2R	5746	-2.15	2.84E-25	0.693989	-2.1494	1.19E-23	0.661316	-2.2	6.66E-28	0.714286	-2.081	1.12E-07	0.7
STX19	415117	NA	NA	NA	NA	NA	NA	-2.09	1.91E-22	0.714286	-2.135	3.93E-05	0.6
GAS2L2	246176	-2.38	1.20E-26	0.677596	-2.5612	4.67E-36	0.695024	-2.553	8.00E-32	0.710317	-2.367	1.84E-10	0.65
CACHD1	57685	-2.578	6.16E-62	0.699454	-2.6282	1.45E-78	0.696629	-2.546	1.19E-65	0.706349	-2.896	1.41E-28	0.65
KLK10	5655	-2.333	8.88E-11	0.699454	-2.425	4.35E-12	0.651685	-2.775	2.54E-14	0.706349	-3.736	2.79E-08	0.75
KRT6B	3854	NA	NA	NA	NA	NA	NA	-2.702	3.93E-11	0.706349	-3.731	7.06E-05	0.75
FRMD3	257019	-2.197	6.84E-30	0.672131	-2.3157	5.20E-34	0.664526	-2.52	9.72E-39	0.702381	-3.255	1.22E-20	0.85
TGM5	9333	NA	NA	NA	NA	NA	NA	-2.182	1.75E-18	0.702381	-2.491	5.55E-06	0.7
SLC5A1	6523	NA	NA	NA	-2.3598	6.82E-13	0.656501	-2.61	1.52E-13	0.702381	NA	NA	NA
LDB3	11155	NA	NA	NA	NA	NA	NA	NA	NA	NA	-2.171	5.15E-09	1
LOC415056	415056	NA	NA	NA	NA	NA	NA	NA	NA	NA	-2.005	4.49E-09	0.95
GABRG1	2565	NA	NA	NA	NA	NA	NA	NA	NA	NA	-2.207	8.83E-09	0.95
FOXE1	2304	NA	NA	NA	NA	NA	NA	NA	NA	NA	-2.129	1.88E-07	0.95
PEBP4	157310	NA	NA	NA	NA	NA	NA	NA	NA	NA	-2.5	2.09E-07	0.95
GPR112	139378	NA	NA	NA	NA	NA	NA	NA	NA	NA	-2.139	5.14E-07	0.95
PEG3AS	1E+08	NA	NA	NA	NA	NA	NA	NA	NA	NA	-2.237	2.10E-06	0.95
ABCB11	8647	NA	NA	NA	NA	NA	NA	NA	NA	NA	-2.065	9.25E-06	0.95
PNLIPRP3	119548	NA	NA	NA	NA	NA	NA	NA	NA	NA	-2.006	0.00167	0.95
F3	2152	-2.046	1.44E-34	0.590164	-2.4183	2.76E-52	0.632424	-2.285	1.10E-40	0.650794	-3.044	7.94E-29	0.9
CAPN3	825	NA	NA	NA	NA	NA	NA	NA	NA	NA	-2.024	9.07E-14	0.9

ESR2	2100	NA	NA	NA	NA	NA	NA	NA	NA	NA	-2.18	1.95E-10	0.9
RYR1	6261	NA	NA	NA	NA	NA	NA	NA	NA	NA	-2.374	1.03E-09	0.9
GALR1	2587	NA	NA	NA	NA	NA	NA	NA	NA	NA	-2.041	3.42E-09	0.9
PLN	5350	NA	NA	NA	NA	NA	NA	NA	NA	NA	-2.018	1.92E-07	0.9
CYP24A1	1591	NA	NA	NA	NA	NA	NA	NA	NA	NA	-2.083	0.001673	0.9
DUSP1	1843	-2.122	3.77E-37	0.622951	-2.3872	1.85E-56	0.683788	-2.21	3.92E-40	0.619048	-3.113	1.35E-23	0.85
NTRK3	4916	-2.367	6.56E-23	0.628415	-3.0373	1.46E-32	0.693419	-2.976	1.97E-29	0.674603	-3.451	3.10E-21	0.85
EDN1	1906	NA	NA	NA	-2.0346	9.18E-36	0.553772	NA	NA	NA	-2.684	1.74E-17	0.85
ARC	23237	-2.251	2.53E-17	0.666667	-2.5585	8.00E-29	0.669342	-2.094	2.15E-17	0.650794	-3.372	3.47E-16	0.85
ZSCAN23	222696	NA	NA	NA	-2.185	1.55E-27	0.59069	-2.193	1.09E-25	0.619048	-2.905	1.76E-14	0.85
TTPA	7274	NA	NA	NA	-2.0563	2.24E-26	0.664526	-2.049	3.20E-21	0.646825	-2.826	5.40E-13	0.85
PTGER3	5733	NA	NA	NA	-2.2122	9.47E-24	0.573034	NA	NA	NA	-2.609	2.93E-12	0.85
CDH22	64405	NA	NA	NA	-2.0095	1.76E-23	0.688604	NA	NA	NA	-2.749	6.00E-12	0.85
FRMPD4	9758	NA	NA	NA	NA	NA	NA	NA	NA	NA	-2.266	3.43E-11	0.85
AGXT	189	NA	NA	NA	NA	NA	NA	NA	NA	NA	-2.013	3.97E-07	0.85
BPI	671	NA	NA	NA	NA	NA	NA	NA	NA	NA	-2.134	1.71E-06	0.85
DMBT1	1755	NA	NA	NA	NA	NA	NA	NA	NA	NA	-2.255	0.000141	0.85
EREG	2069	NA	NA	NA	NA	NA	NA	NA	NA	NA	-2.325	0.000731	0.85
PROS1	5627	-2.209	8.68E-57	0.562842	-2.4208	1.90E-84	0.664526	-2.23	1.29E-62	0.59127	-2.624	1.73E-33	0.8
PARK2	5071	-2.09	4.78E-60	0.595628	-2.2907	1.29E-63	0.613162	-2.247	5.98E-56	0.630952	-2.825	2.14E-31	0.8
EMCN	51705	NA	NA	NA	-2.3432	7.60E-76	0.664526	-2.031	1.52E-48	0.551587	-2.431	2.68E-27	0.8
PLSCR4	57088	-2.085	1.09E-52	0.568306	-2.4284	3.46E-87	0.678973	-2.292	2.54E-59	0.642857	-2.542	3.48E-27	0.8
SPRY1	10252	NA	NA	NA	-2.206	3.17E-71	0.609952	NA	NA	NA	-2.26	4.53E-27	0.8
ST6GALNAC1	55808	-2.38	5.25E-30	0.63388	-2.6159	8.08E-32	0.691814	-2.311	1.76E-25	0.626984	-3.435	1.58E-26	0.8
S1PR1	1901	-2.015	6.26E-49	0.584699	-2.2982	3.03E-77	0.698234	-2.032	3.15E-45	0.619048	-2.448	9.33E-22	0.8
LOC401093	401093	NA	NA	NA	NA	NA	NA	-2.024	8.18E-61	0.634921	-2.346	2.01E-21	0.8
SELP	6403	-2.13	1.69E-22	0.530055	-3.0906	1.30E-35	0.670947	-2.505	1.09E-24	0.56746	-3.278	8.67E-21	0.8
GPR44	11251	-2.003	4.18E-36	0.63388	-2.1455	2.40E-49	0.670947	-2.035	7.38E-37	0.587302	-2.361	1.33E-20	0.8
ADHFE1	137872	-2.006	1.31E-41	0.595628	-2.3801	1.04E-56	0.677368	-2.313	6.94E-44	0.65873	-2.703	4.88E-20	0.8
PTPRB	5787	NA	NA	NA	-2.1361	1.81E-62	0.619583	NA	NA	NA	-2.399	8.48E-19	0.8
TEK	7010	NA	NA	NA	-2.1257	7.65E-58	0.605136	NA	NA	NA	-2.269	3.31E-17	0.8
CXCL1	2919	NA	NA	NA	-2.1116	1.02E-16	0.629213	-2.644	7.53E-27	0.694444	-3.08	2.74E-15	0.8
PRUNE2	158471	NA	NA	NA	-2.0131	1.82E-37	0.642055	-2.019	9.72E-39	0.626984	-2.559	4.41E-15	0.8
DIRAS3	9077	NA	NA	NA	NA	NA	NA	NA	NA	NA	-2.881	1.43E-13	0.8

PKIA	5569	NA	NA	NA	NA	NA	NA	NA	NA	NA	-2.113	1.43E-13	0.8
CYR61	3491	NA	NA	NA	NA	NA	NA	NA	NA	NA	-2.399	4.16E-13	0.8
PZP	5858	NA	NA	NA	NA	NA	NA	NA	NA	NA	-2.133	2.58E-12	0.8
PAR5	8123	NA	NA	NA	-2.2472	5.38E-29	0.619583	-2.093	4.10E-23	0.615079	-3.226	5.83E-12	0.8
CLEC10A	10462	NA	NA	NA	-2.2668	1.12E-21	0.592295	-2.012	1.68E-17	0.563492	-2.587	7.27E-12	0.8
SLC25A18	83733	NA	NA	NA	NA	NA	NA	NA	NA	NA	-2.234	2.18E-11	0.8
ABCC6P1	653190	-2.071	1.05E-22	0.655738	-2.2062	3.06E-32	0.651685	-2.181	3.64E-26	0.678571	-2.394	1.00E-10	0.8
LOC10012678	1E+08	NA	NA	NA	-2.0654	1.24E-31	0.662921	NA	NA	NA	-2.395	2.97E-10	0.8
APOLD1	81575	NA	NA	NA	NA	NA	NA	NA	NA	NA	-2.308	5.58E-10	0.8
STX11	8676	NA	NA	NA	NA	NA	NA	NA	NA	NA	-2.176	1.11E-09	0.8
ERVFRDE1	405754	NA	NA	NA	NA	NA	NA	NA	NA	NA	-2.323	1.28E-09	0.8
TP53TG3B	729355	NA	NA	NA	NA	NA	NA	NA	NA	NA	-2.204	3.35E-08	0.8
KLK13	26085	NA	NA	NA	NA	NA	NA	NA	NA	NA	-2.384	1.02E-07	0.8
CHST4	10164	NA	NA	NA	NA	NA	NA	NA	NA	NA	-2.198	1.13E-07	0.8
ANXA8	653145	NA	NA	NA	-2.2898	1.73E-13	0.621188	-2.467	1.33E-14	0.68254	-3.431	2.43E-07	0.8
MPPED1	758	NA	NA	NA	NA	NA	NA	NA	NA	NA	-2.264	4.96E-07	0.8
IL1RAPL2	26280	NA	NA	NA	NA	NA	NA	NA	NA	NA	-2.454	5.50E-07	0.8
OR7A5	26659	NA	NA	NA	NA	NA	NA	NA	NA	NA	-2.013	1.48E-06	0.8
NPR3	4883	NA	NA	NA	NA	NA	NA	NA	NA	NA	-2.636	5.51E-06	0.8
MUC16	94025	-2.312	6.97E-10	0.699454	NA	NA	NA	-2.17	1.99E-09	0.674603	-2.844	1.83E-05	0.8
LGALS7	3963	NA	NA	NA	NA	NA	NA	NA	NA	NA	-2.113	0.000195	0.8
JAM2	58494	-2.186	3.05E-56	0.579235	-2.4234	8.16E-79	0.656501	NA	NA	NA	-2.527	3.59E-33	0.75
PELI2	57161	-2.023	4.82E-48	0.480874	-2.25	1.82E-64	0.589085	-2.012	7.50E-50	0.5	-2.649	6.05E-30	0.75
psiTPTE22	387590	NA	NA	NA	-2.297	5.55E-28	0.585875	-2.113	3.73E-21	0.559524	-3.025	1.46E-29	0.75
LHFP	10186	-2.079	5.73E-62	0.562842	-2.3441	3.88E-95	0.675762	-2.064	1.05E-56	0.559524	-2.248	1.12E-25	0.75
PRIMA1	145270	-2.497	3.15E-36	0.650273	-2.5424	7.18E-41	0.64687	-2.603	5.39E-39	0.65873	-3.514	1.06E-24	0.75
EFEMP1	2202	-2.361	1.24E-39	0.617486	-2.6191	1.24E-49	0.672552	-2.38	1.61E-51	0.694444	-2.904	1.96E-24	0.75
PRDM16	63976	NA	NA	NA	-2.2211	1.53E-47	0.642055	NA	NA	NA	-2.745	1.27E-22	0.75
NOVA1	4857	-2.164	2.38E-18	0.595628	-2.8339	9.54E-27	0.685393	-2.305	1.62E-22	0.611111	-3.217	1.46E-22	0.75
FLRT2	23768	-2.119	8.61E-37	0.519126	-2.3893	6.34E-52	0.622793	-2.127	8.87E-42	0.551587	-2.551	2.12E-22	0.75
IRS2	8660	NA	NA	NA	NA	NA	NA	NA	NA	NA	-2.311	2.36E-22	0.75
MYCT1	80177	NA	NA	NA	-2.1722	1.57E-82	0.654896	NA	NA	NA	-2.199	5.87E-21	0.75
LEPREL1	55214	NA	NA	NA	-2.1144	1.72E-49	0.606742	-2.116	2.34E-46	0.571429	-2.36	6.67E-21	0.75
PVRL3	25945	-2.221	9.81E-41	0.601093	-2.3517	3.52E-52	0.634029	-2.237	1.54E-41	0.607143	-2.735	2.49E-20	0.75

C10orf128	170371	-2.311	9.79E-37	0.655738	-2.5214	8.99E-57	0.670947	-2.311	7.86E-38	0.611111	-2.61	8.69E-20	0.75
FAT4	79633	-2.11	2.61E-40	0.568306	-2.3742	3.01E-52	0.616372	-2.144	1.95E-39	0.575397	-2.587	8.91E-20	0.75
FGF1	2246	-2.331	2.96E-48	0.622951	-2.6084	3.65E-74	0.698234	-2.349	8.48E-55	0.654762	-2.579	1.59E-19	0.75
LAMA3	3909	-2.39	4.31E-27	0.612022	-2.7278	9.54E-39	0.670947	-2.692	5.02E-35	0.678571	-3.695	8.47E-19	0.75
CXCL14	9547	NA	NA	NA	-2.2583	1.07E-17	0.544141	NA	NA	NA	-3.107	1.09E-18	0.75
L3MBTL4	91133	-2.037	3.49E-29	0.601093	-2.1246	5.29E-32	0.600321	-2.379	1.86E-40	0.650794	-2.697	9.02E-18	0.75
COL14A1	7373	-2.125	4.57E-26	0.535519	-2.5522	8.79E-40	0.634029	-2.014	2.23E-26	0.543651	-2.871	3.28E-17	0.75
RCAN1	1827	-2.058	1.20E-38	0.672131	-2.1004	1.88E-54	0.648475	-2.234	1.11E-45	0.670635	-2.521	6.80E-17	0.75
GPR116	221395	NA	NA	NA	NA	NA	NA	NA	NA	NA	-2.105	6.96E-17	0.75
PTGIS	5740	-2.011	2.58E-25	0.639344	-2.2746	2.49E-43	0.682183	-2.049	1.73E-34	0.662698	-3.197	9.77E-17	0.75
CNGA1	1259	-2.312	2.86E-24	0.650273	-2.489	2.26E-32	0.670947	-2.762	6.81E-35	0.68254	-3.132	1.32E-16	0.75
PPP1R16B	26051	NA	NA	NA	-2.2738	1.37E-49	0.666132	-2.186	2.52E-37	0.619048	-2.519	4.14E-16	0.75
CLEC4F	165530	NA	NA	NA	-2.0672	6.24E-27	0.598716	-2.106	2.12E-25	0.571429	-2.872	5.52E-16	0.75
GRIN2A	2903	NA	NA	NA	-2.0032	3.82E-24	0.621188	NA	NA	NA	-2.843	6.57E-16	0.75
DLC1	10395	NA	NA	NA	NA	NA	NA	NA	NA	NA	-2.141	1.04E-15	0.75
ALDH1A3	220	-2.012	4.53E-26	0.601093	-2.3442	2.77E-44	0.654896	-2.359	3.78E-37	0.666667	-2.607	2.53E-15	0.75
HMGCLL1	54511	-2.216	1.99E-20	0.584699	-2.3678	3.46E-22	0.616372	-2.523	1.74E-26	0.670635	-3.158	2.70E-15	0.75
TMEM22	80723	-2.011	1.23E-49	0.68306	-2.0974	7.88E-78	0.690209	NA	NA	NA	-2.211	2.71E-15	0.75
PGM5P2	595135	NA	NA	NA	NA	NA	NA	NA	NA	NA	-2.1	4.20E-15	0.75
SLC7A4	6545	NA	NA	NA	-2.194	8.73E-12	0.577849	-2.24	7.00E-12	0.583333	-3.448	6.50E-15	0.75
PPP2R2B	5521	NA	NA	NA	-2.0399	1.48E-37	0.600321	-2.135	2.46E-37	0.630952	-2.568	6.86E-15	0.75
ZNF366	167465	NA	NA	NA	-2.1815	9.61E-43	0.59069	NA	NA	NA	-2.377	1.17E-14	0.75
SLIT3	6586	-2.216	1.35E-41	0.628415	-2.4598	1.03E-58	0.683788	-2.098	1.90E-43	0.623016	-2.245	5.60E-14	0.75
KLRF1	51348	NA	NA	NA	NA	NA	NA	NA	NA	NA	-2.26	3.33E-13	0.75
LAMB3	3914	-2.162	2.23E-27	0.63388	-2.3281	1.28E-33	0.627608	-2.538	1.58E-32	0.678571	-2.737	4.49E-13	0.75
ABO	28	NA	NA	NA	NA	NA	NA	NA	NA	NA	-2.493	4.02E-12	0.75
HYMAI	57061	NA	NA	NA	NA	NA	NA	NA	NA	NA	-2.076	4.88E-12	0.75
AGPAT9	84803	NA	NA	NA	NA	NA	NA	NA	NA	NA	-2.09	6.12E-12	0.75
THSD7A	221981	NA	NA	NA	NA	NA	NA	NA	NA	NA	-2.097	6.79E-12	0.75
AFF2	2334	NA	NA	NA	NA	NA	NA	NA	NA	NA	-2.303	9.63E-12	0.75
WFIKKN2	124857	NA	NA	NA	NA	NA	NA	NA	NA	NA	-2.021	1.43E-11	0.75
GNG2	54331	NA	NA	NA	NA	NA	NA	NA	NA	NA	-2.067	4.80E-11	0.75
P2RY14	9934	NA	NA	NA	NA	NA	NA	NA	NA	NA	-2.054	1.15E-10	0.75
SEMA3A	10371	NA	NA	NA	-2.0644	2.57E-41	0.608347	NA	NA	NA	-2.017	1.52E-10	0.75

ENPP6	133121	NA	NA	NA	NA	NA	NA	NA	NA	NA	-2.243	1.61E-10	0.75
PDZRN4	29951	-2.042	1.77E-18	0.666667	-2.1184	2.79E-21	0.675762	-2.016	1.45E-17	0.666667	-2.606	3.75E-10	0.75
ANO4	121601	NA	NA	NA	NA	NA	NA	NA	NA	NA	-2.148	7.51E-10	0.75
DCHS2	54798	NA	NA	NA	NA	NA	NA	-2.222	1.10E-30	0.68254	-2.387	1.72E-09	0.75
CRHR1	1394	NA	NA	NA	-2.0563	1.65E-16	0.688604	-2.405	2.83E-23	0.686508	-2.64	7.20E-08	0.75
ENPP3	5169	NA	NA	NA	NA	NA	NA	NA	NA	NA	-2.102	1.47E-07	0.75
SLC35F3	148641	NA	NA	NA	-2.393	9.64E-21	0.678973	-2.471	6.72E-19	0.686508	-3.454	1.53E-07	0.75
LEFTY2	7044	NA	NA	NA	-2.1644	2.58E-18	0.674157	-2.005	6.81E-17	0.646825	-2.26	6.08E-07	0.75
MT1A	4489	NA	NA	NA	-2.0033	9.37E-17	0.627608	NA	NA	NA	-2.065	9.56E-07	0.75
HCN1	348980	NA	NA	NA	NA	NA	NA	NA	NA	NA	-2.022	3.41E-06	0.75
MUC4	4585	NA	NA	NA	NA	NA	NA	NA	NA	NA	-2.375	3.83E-06	0.75
BCL11A	53335	NA	NA	NA	NA	NA	NA	NA	NA	NA	-2.564	3.09E-05	0.75
FRMPD2	143162	NA	NA	NA	NA	NA	NA	NA	NA	NA	-2.036	0.000863	0.75
PDZK1	5174	NA	NA	NA	NA	NA	NA	NA	NA	NA	-2.09	0.001675	0.75
FOXI1	2299	NA	NA	NA	NA	NA	NA	NA	NA	NA	-2.294	0.003693	0.75
SPARCL1	8404	NA	NA	NA	-2.2343	1.56E-68	0.568218	NA	NA	NA	-2.502	1.70E-31	0.7
PTPN21	11099	NA	NA	NA	NA	NA	NA	NA	NA	NA	-2.124	1.21E-29	0.7
PCDHGB7	56099	-2.166	2.67E-37	0.508197	-2.5509	5.09E-51	0.59069	-2.282	9.65E-40	0.52381	-2.711	1.25E-28	0.7
CYYR1	116159	-2.047	1.25E-62	0.535519	-2.2442	8.19E-78	0.65008	-2.226	3.62E-64	0.638889	-2.292	1.12E-27	0.7
NFIB	4781	-2.386	3.15E-42	0.639344	-2.3281	2.18E-49	0.58748	-2.329	1.99E-53	0.603175	-2.335	1.60E-27	0.7
PPAP2B	8613	-2.001	4.49E-65	0.535519	-2.1118	1.22E-105	0.597111	-2.032	3.34E-66	0.543651	-2.168	1.78E-27	0.7
FAM126A	84668	-2.203	1.61E-55	0.612022	-2.31	6.10E-74	0.622793	-2.285	2.22E-56	0.634921	-2.671	1.32E-26	0.7
PLAGL1	5325	-2.231	1.32E-45	0.601093	-2.2042	2.89E-59	0.5939	-2.171	3.49E-47	0.595238	-2.445	1.32E-25	0.7
ZNF204P	7754	-2.102	3.30E-35	0.508197	-2.322	5.13E-41	0.518459	-2.334	3.25E-36	0.543651	-3.159	4.80E-25	0.7
RND3	390	NA	NA	NA	NA	NA	NA	NA	NA	NA	-2.55	4.38E-23	0.7
ROBO3	64221	-2.047	1.46E-42	0.568306	-2.2762	3.23E-64	0.632424	-2.073	1.30E-36	0.575397	-2.262	8.33E-22	0.7
BOC	91653	-2.135	1.39E-44	0.595628	-2.2383	2.81E-54	0.600321	-2.143	4.84E-45	0.599206	-2.265	1.93E-21	0.7
RUNDC3B	154661	-2.003	4.33E-46	0.513661	-2.2142	1.22E-58	0.5939	NA	NA	NA	-2.397	3.41E-21	0.7
NEDD9	4739	NA	NA	NA	NA	NA	NA	NA	NA	NA	-2.235	5.45E-21	0.7
WASF3	10810	-2.349	2.27E-44	0.628415	-2.5128	7.41E-67	0.674157	-2.562	6.76E-50	0.674603	-2.745	9.21E-21	0.7
PDE7B	27115	NA	NA	NA	NA	NA	NA	NA	NA	NA	-2.108	1.92E-20	0.7
TNNI3K	51086	-2.043	1.23E-26	0.551913	-2.0696	7.03E-29	0.552167	-2.267	1.32E-34	0.587302	-2.948	2.39E-20	0.7
PCDHGB6	56100	-2.099	1.15E-36	0.540984	-2.1318	8.90E-41	0.568218	-2.033	3.16E-35	0.543651	-2.335	2.92E-20	0.7
SORBS2	8470	NA	NA	NA	NA	NA	NA	NA	NA	NA	-2.48	4.58E-20	0.7

SOCS2	8835	NA	NA	NA	-2.0943	6.58E-39	0.573034	NA	NA	NA	-2.308	4.75E-20	0.7
TAL1	6886	NA	NA	NA	-2.2419	2.84E-75	0.662921	NA	NA	NA	-2.201	5.19E-20	0.7
TM4SF18	116441	NA	NA	NA	-2.1486	3.13E-57	0.654896	-2.274	8.65E-57	0.690476	-2.258	5.27E-20	0.7
MMRN2	79812	NA	NA	NA	-2.1105	3.47E-82	0.638844	NA	NA	NA	-2.015	1.90E-19	0.7
NMT2	9397	NA	NA	NA	-2.0109	6.86E-79	0.624398	-2.067	1.11E-66	0.630952	-2.217	2.47E-19	0.7
PRCD	768206	-2.091	2.09E-51	0.666667	-2.1429	9.13E-78	0.666132	NA	NA	NA	-2.051	3.02E-19	0.7
EPHB6	2051	-2.058	1.79E-27	0.551913	NA	NA	NA	-2.225	8.07E-33	0.603175	-2.402	3.15E-19	0.7
F8	2157	NA	NA	NA	NA	NA	NA	NA	NA	NA	-2.149	4.07E-19	0.7
SOX7	83595	NA	NA	NA	-2.0413	1.62E-65	0.629213	NA	NA	NA	-2.236	5.11E-19	0.7
TMEM88	92162	NA	NA	NA	-2.3217	3.20E-73	0.682183	-2.012	1.47E-41	0.575397	-2.064	5.21E-19	0.7
CELF2	10659	NA	NA	NA	-2.1305	2.90E-61	0.629213	-2.074	2.02E-50	0.623016	-2.51	6.64E-19	0.7
MRGPRF	116535	-2.148	3.61E-38	0.584699	-2.5001	1.24E-64	0.680578	-2.148	8.63E-42	0.599206	-2.359	8.07E-19	0.7
NBLA00301	79804	-2.287	2.35E-45	0.644809	-2.3685	1.50E-61	0.642055	-2.236	4.39E-42	0.611111	-2.545	1.07E-18	0.7
STEAP4	79689	NA	NA	NA	-2.2233	2.54E-22	0.5939	-2.199	2.92E-28	0.583333	-2.666	2.48E-18	0.7
TACC1	6867	-2.001	1.81E-46	0.551913	-2.088	7.02E-59	0.600321	NA	NA	NA	-2.301	2.98E-18	0.7
DAAM2	23500	NA	NA	NA	NA	NA	NA	NA	NA	NA	-2.246	4.10E-18	0.7
BACH2	60468	NA	NA	NA	NA	NA	NA	NA	NA	NA	-2.509	8.85E-18	0.7
ZNF488	118738	NA	NA	NA	NA	NA	NA	NA	NA	NA	-2.633	4.48E-17	0.7
MAP7D3	79649	-2.034	5.16E-43	0.617486	-2.1096	3.31E-55	0.629213	-2.092	6.70E-52	0.634921	-2.204	1.03E-16	0.7
ST6GALNAC3	256435	NA	NA	NA	-2.1243	4.80E-69	0.666132	-2.071	5.18E-54	0.646825	-2.13	1.23E-16	0.7
RUNX1T1	862	-2.301	4.09E-37	0.590164	-2.6051	2.70E-49	0.656501	-2.263	8.86E-38	0.615079	-2.504	1.47E-16	0.7
MRAP2	112609	-2.015	1.09E-20	0.535519	-2.1925	3.44E-27	0.561798	-2.477	8.66E-29	0.619048	-3.065	2.25E-16	0.7
SCAND3	114821	NA	NA	NA	NA	NA	NA	NA	NA	NA	-3.451	2.61E-16	0.7
PCDHGA3	56112	-2.235	1.78E-42	0.661202	-2.3869	1.01E-49	0.686998	-2.121	6.54E-37	0.59127	-2.283	3.06E-16	0.7
SPTBN1	6711	NA	NA	NA	NA	NA	NA	NA	NA	NA	-2.005	4.22E-16	0.7
LAMA2	3908	-2.074	2.27E-27	0.551913	-2.4355	3.05E-41	0.619583	NA	NA	NA	-2.597	5.25E-16	0.7
CCDC158	339965	NA	NA	NA	-2.5258	3.13E-33	0.627608	-2.599	1.02E-31	0.686508	-2.988	5.71E-16	0.7
TLN2	83660	NA	NA	NA	NA	NA	NA	NA	NA	NA	-2.02	3.79E-15	0.7
GCNT4	51301	NA	NA	NA	-2.0789	5.04E-31	0.582665	NA	NA	NA	-2.373	4.77E-15	0.7
ADAMTSL4	54507	-2.065	5.48E-39	0.666667	-2.1192	2.44E-52	0.667737	-2.02	1.15E-43	0.634921	-2.278	7.76E-15	0.7
NCALD	83988	-2.269	3.65E-45	0.693989	-2.1431	2.60E-45	0.667737	-2.19	5.71E-46	0.68254	-2.158	8.40E-15	0.7
SUGT1P1	441394	NA	NA	NA	NA	NA	NA	NA	NA	NA	-2.003	9.35E-15	0.7
CYP4F12	66002	NA	NA	NA	-2.4011	9.06E-22	0.632424	-2.464	5.78E-24	0.646825	-3.03	1.17E-14	0.7
CPAMD8	27151	-2.065	2.08E-26	0.606557	-2.131	2.41E-31	0.58748	-2.101	3.05E-26	0.551587	-2.41	1.97E-14	0.7

PCDH18	54510	NA	NA	NA	-2.1378	1.54E-58	0.635634	NA	NA	NA	-2.196	2.45E-14	0.7
C6orf123	26238	NA	NA	NA	NA	NA	NA	NA	NA	NA	-2.036	4.49E-14	0.7
MYH3	4621	NA	NA	NA	NA	NA	NA	NA	NA	NA	-2.133	1.62E-13	0.7
ANO5	203859	NA	NA	NA	-2.0753	1.72E-16	0.64366	-2.185	3.09E-18	0.674603	-2.791	3.34E-13	0.7
SYT15	83849	NA	NA	NA	-2.2416	1.01E-53	0.667737	-2.064	1.18E-34	0.595238	-2.269	3.39E-13	0.7
VGLL3	389136	NA	NA	NA	-2.0983	2.57E-45	0.603531	NA	NA	NA	-2.403	3.60E-13	0.7
DDIT4L	115265	NA	NA	NA	NA	NA	NA	NA	NA	NA	-2.286	2.07E-12	0.7
ZNF727	442319	-2.342	3.10E-24	0.612022	-2.5301	3.06E-36	0.659711	-2.446	9.06E-31	0.654762	-2.54	3.37E-12	0.7
C4orf31	79625	-2.114	3.36E-23	0.57377	-2.5865	4.65E-36	0.661316	-2.268	8.12E-28	0.623016	-2.145	8.29E-12	0.7
CNNM1	26507	-2.161	5.14E-20	0.63388	-2.0467	5.45E-19	0.616372	-2.162	1.68E-22	0.638889	-2.553	1.34E-11	0.7
FERMT1	55612	-2.105	2.36E-16	0.601093	-2.0679	6.16E-15	0.579454	-2.409	1.96E-21	0.662698	-3.106	4.93E-11	0.7
PHYHIPL	84457	NA	NA	NA	-2.237	1.81E-35	0.675762	-2.178	1.94E-26	0.678571	-2.527	1.59E-10	0.7
LOC10013354	1E+08	NA	NA	NA	-2.0566	5.27E-37	0.675762	NA	NA	NA	-2.131	1.97E-10	0.7
CDKL2	8999	-2.055	4.96E-17	0.622951	-2.2384	4.43E-25	0.662921	-2.146	1.59E-19	0.650794	-2.66	5.29E-09	0.7
ITIH3	3699	NA	NA	NA	NA	NA	NA	NA	NA	NA	-2.035	7.78E-09	0.7
ANKK1	255239	NA	NA	NA	NA	NA	NA	NA	NA	NA	-2.247	4.40E-08	0.7
HEPACAM2	253012	NA	NA	NA	-2.152	1.59E-11	0.622793	-2.302	2.51E-12	0.662698	-2.742	3.07E-05	0.7
CYP4F22	126410	NA	NA	NA	NA	NA	NA	-2.026	2.13E-09	0.678571	-2.864	3.95E-05	0.7
FAM3B	54097	-2.027	3.27E-10	0.655738	NA	NA	NA	NA	NA	NA	-2.287	7.50E-05	0.7
RNF183	138065	NA	NA	NA	NA	NA	NA	NA	NA	NA	-2.099	0.000112	0.7
VWDE	221806	NA	NA	NA	NA	NA	NA	NA	NA	NA	-2.415	0.000248	0.7
KRT16	3868	NA	NA	NA	NA	NA	NA	NA	NA	NA	-2.102	0.00129	0.7
LOC642587	642587	NA	NA	NA	NA	NA	NA	NA	NA	NA	-2.685	0.001317	0.7
PPP4R4	57718	NA	NA	NA	NA	NA	NA	NA	NA	NA	-2.183	0.001629	0.7

Note: NA denotes the gene is not down regulated in that cancer stage.

Supplementary Table 4. Amplified regions

Cytoband	Start_position	End_position
1p11.2	120600000	121500000
1q21.1	142600000	147000000
1q21.2	147000000	150300000
1q21.3	150300000	155000000
1q22	155000000	156500000
1q23.1	156500000	159100000
1q23.2	159100000	160500000
1q23.3	160500000	165500000
1q24.1	165500000	167200000
1q24.2	167200000	170900000
1q24.3	170900000	172900000
1q25.1	172900000	176000000
1q25.2	176000000	180300000
1q25.3	180300000	185800000
1q31.1	185800000	190800000
1q31.2	190800000	193800000
1q31.3	193800000	198700000
1q32.1	198700000	207200000
1q32.2	207200000	211500000
1q32.3	211500000	214500000
1q41	214500000	224100000
1q42.11	224100000	224600000
1q42.12	224600000	227000000
1q42.13	227000000	230700000
1q42.2	230700000	234700000
1q42.3	234700000	236600000
1q43	236600000	243700000
1q44	243700000	249250621
16p13.3	0	7900000
16p13.2	7900000	10500000
16p13.13	10500000	12600000
16p13.12	12600000	14800000
16p13.11	14800000	16800000
16p12.3	16800000	21200000
16p12.2	21200000	24200000
16p12.1	24200000	28100000
16p11.2	28100000	34600000
20q11.21	29400000	32100000
20q11.22	32100000	34400000
20q11.23	34400000	37600000
20q12	37600000	41700000
20q13.11	41700000	42100000
20q13.12	42100000	46400000
20q13.13	46400000	49800000
20q13.2	49800000	55000000

20q13.31	55000000	56500000
20q13.32	56500000	58400000
20q13.33	58400000	63025520
8p11.1	43100000	45600000
8q11.1	45600000	48100000
8q11.21	48100000	52200000
8q11.22	52200000	52600000
8q11.23	52600000	55500000
8q12.1	55500000	61600000
8q12.2	61600000	62200000
8q12.3	62200000	66000000
8q13.1	66000000	68000000
8q13.2	68000000	70500000
8q13.3	70500000	73900000
8q21.11	73900000	78300000
8q21.12	78300000	80100000
8q21.13	80100000	84600000
8q21.2	84600000	86900000
8q21.3	86900000	93300000
8q22.1	93300000	99000000
8q22.2	99000000	101600000
8q22.3	101600000	106200000
8q23.1	106200000	110500000
8q23.2	110500000	112100000
8q23.3	112100000	117700000
8q24.11	117700000	119200000
8q24.12	119200000	122500000
8q24.13	122500000	127300000
8q24.21	127300000	131500000
8q24.22	131500000	136400000
8q24.23	136400000	139900000
8q24.3	139900000	146364022

Supplementary Table 5a. Evaluations on the concordance between expression and CNA for up-regulated genes located in amplification bands in overall patients.

GeneName	GeneID	fold CNA-associated	FDR	cytoband	Pearson's correlation	Status
TPX2	22974	0.979955674	5.48E-36	20q11.21	0.331729825	drive by CNA
NUF2	83540	0.963225162	3.27E-24	1q23.3	0.32846242	drive by CNA
IQGAP3	128239	0.899340901	3.70E-23	1q22	0.313480744	drive by CNA
AURKA	6790	1.191872753	2.34E-61	20q13.2	0.480852097	driven by CNA
CCDC78	124093	1.199164513	4.77E-24	16p13.3	0.340447821	driven by CNA
GNG13	51764	1.614567685	9.52E-23	16p13.3	0.328336648	driven by CNA
UBE2C	11065	1.119444797	1.42E-34	20q13.12	0.311759672	driven by CNA
FAM83D	81610	1.032255021	7.24E-30	20q11.23	0.285806576	driven by CNA
MYBL2	4605	1.173765095	6.23E-28	20q13.12	0.261383604	driven by CNA
NEK2	4751	0.991649695	1.51E-30	1q32.3	0.296687961	not driven by CNA
HAGHL	84264	0.79610432	1.00E-15	16p13.3	0.277643457	not driven by CNA
DTL	51514	0.783509964	4.04E-23	1q32.3	0.27704762	not driven by CNA
EXO1	9156	0.857428416	2.54E-18	1q43	0.257424837	not driven by CNA
UBE2T	29089	0.787948542	3.39E-22	1q32.1	0.247741982	not driven by CNA
CCDC19	25790	0.870449182	1.17E-15	1q23.2	0.233037511	not driven by CNA
CENPF	1063	0.780688164	3.17E-19	1q41	0.231821563	not driven by CNA
FAM72D	728833	0.629567591	6.69E-13	1q21.1	0.204378016	not driven by CNA
KIF14	9928	0.869788504	1.23E-15	1q32.1	0.202207022	not driven by CNA
FAM72B	653820	0.544826124	7.49E-11	1p11.2	0.199997426	not driven by CNA
ASPM	259266	0.804741752	1.01E-15	1q31.3	0.196334855	not driven by CNA
HIST2H3C	126961	0.683988011	1.62E-10	1q21.2	0.177584871	not driven by CNA
C16orf59	80178	0.422625679	6.87E-10	16p13.3	0.14657828	not driven by CNA
SYNGR3	9143	0.476030702	2.68E-06	16p13.3	0.133863326	not driven by CNA
SRMS	6725	0.098005799	0.435872	20q13.33	0.099239201	not driven by CNA
HIST3H2BB	128312	0.408423075	0.004316	1q42.13	0.091339237	not driven by CNA
PKMYT1	9088	0.336009792	7.29E-05	16p13.3	0.069630728	not driven by CNA
CTHRC1	115908	0.186764281	0.017664	8q22.3	0.061521603	not driven by CNA
WISP1	8840	-0.088551891	0.35029	8q24.22	-0.030220721	not driven by CNA
PLK1	5347	0.069919371	0.400561	16p12.2	-0.04532341	not driven by CNA

Supplementary Table 5b. Evaluations on the coordence between expression and CNA for genes located in amplification bands in TNBC patients.

GeneName	GeneID	fold CNA-associated change	FDR	cytoband	Pearson's correlation	Status
PLK1	5347	0.230190395	0.182694	16p12.2	0.037959785	not driven by CNA
UBE2C	11065	0.828438538	3.60E-06	20q13.12	0.310224168	driven by CNA
MYBL2	4605	0.616039529	0.001169	20q13.12	0.204560091	not driven by CNA
TPX2	22974	0.669849397	2.53E-05	20q11.21	0.317537322	driven by CNA
IQGAP3	128239	1.624494179	4.16E-12	1q22	0.552582547	driven by CNA
NEK2	4751	1.217385626	4.61E-12	1q32.3	0.400655959	driven by CNA
ASPM	259266	1.227593745	2.39E-09	1q31.3	0.376586574	driven by CNA
EXO1	9156	1.211741576	6.61E-11	1q43	0.461163326	driven by CNA
PKMYT1	9088	0.428662926	0.021959	16p13.3	0.115722141	not driven by CNA
AURKA	6790	0.686110218	5.96E-07	20q13.2	0.396639356	driven by CNA
FAM72B	653820	0.930680332	3.29E-06	1p11.2	0.33406944	driven by CNA
NUF2	83540	1.46355143	3.69E-11	1q23.3	0.512662819	driven by CNA
FAM83D	81610	0.691262899	8.35E-05	20q11.23	0.239039736	not driven by CNA
KIF14	9928	1.091691227	6.51E-08	1q32.1	0.333663067	driven by CNA
UBE2T	29089	0.979660003	1.28E-08	1q32.1	0.369047368	driven by CNA
FAM72A	729533	1.087675795	4.38E-07	1q32.1	0.28610451	driven by CNA
CENPF	1063	1.005608927	7.67E-08	1q41	0.332179927	driven by CNA
ZNF695	57116	1.500517352	2.98E-11	1q44	0.456820726	driven by CNA
FAM72D	728833	0.859930322	0.000847	1q21.1	0.277856135	not driven by CNA
E2F1	1869	0.51341676	0.000762	20q11.22	0.270471923	not driven by CNA
DTL	51514	0.80119728	3.56E-07	1q32.3	0.31588731	driven by CNA
RECQL4	9401	1.086202123	5.55E-09	8q24.3	0.450395601	driven by CNA
CTHRC1	115908	0.748109897	0.000961	8q22.3	0.272490435	not driven by CNA
HIST2H3C	126961	0.661723954	0.014653	1q21.2	0.223542703	not driven by CNA
SH2D2A	9047	0.259575072	0.214219	1q23.1	0.173552499	not driven by CNA
KIF26B	55083	-0.082370301	0.786051	1q44	-0.088879566	not driven by CNA
COL9A3	1299	1.37353883	0.003369	20q13.33	0.293835504	not driven by CNA
C16orf59	80178	0.482220842	0.006602	16p13.3	0.158356804	not driven by CNA
MEX3A	92312	1.832922188	1.04E-08	1q22	0.371195164	driven by CNA
PAQR4	124222	0.550295268	0.000191	16p13.3	0.267152833	not driven by CNA
CCNE2	9134	1.021716379	3.55E-05	8q22.1	0.192865627	driven by CNA

KREMEN2	79412	0.730572952	0.024182	16p13.3	0.176155242	not driven by CNA
HORMAD1	84072	3.164363379	0.000535	1q21.3	0.331586422	driven by CNA
C1orf106	55765	0.215822165	0.442202	1q32.1	0.111433413	not driven by CNA
HIST2H2AA3	8337	0.042984967	0.874929	1q21.2	0.092448681	not driven by CNA
SLC39A4	55630	0.815020686	0.000391	8q24.3	0.32521318	driven by CNA

Supplementary Table 6a. 143 EEA genes in overall patients

GeneName	GeneID
COL10A1	1300
MMP11	4320
IBSP	3381
NEK2	4751
PKMYT1	9088
COL11A1	1301
KIF4A	24137
PPAPDC1A	196051
CCL11	6356
CDC25C	995
COMP	1311
CST4	1472
GABRD	2563
PLAC1	10761
MMP13	4322
KIAA0101	9768
IQGAP3	128239
HAGHL	84264
NUF2	83540
TROAP	10024
GJB2	2706
MELK	9833
SPC25	57405
TPX2	22974
UHRF1	29128
PLK1	5347
SCT	6343
SPC24	147841
CST1	1469
PITX1	5307
KIF20A	10112
HSD17B6	8630
SLC24A2	25769
OR2B6	26212
WBSCR28	135886
WISP1	8840
LRRC15	131578
TDO2	6999
EPYC	1833
MMP1	4312
CILP2	148113
PBK	55872
HJURP	55355
DLGAP5	9787
BUB1	699

NEIL3	55247
CEP55	55165
EXO1	9156
ASF1B	55723
UBE2T	29089
KIF18B	146909
ANLN	54443
KIF2C	11004
PTTG1	9232
NUSAP1	51203
HIST1H2BO	8348
C16orf59	80178
HIST1H2AM	8336
CDC20	991
KIF14	9928
NDC80	10403
RRM2	6241
CDK1	983
SKA3	221150
CCNB2	9133
KIFC1	3833
CNIH2	254263
HMMR	3161
CKAP2L	150468
FOXM1	2305
DTL	51514
TOP2A	7153
CDKN3	1033
CDC45	8318
CST2	1470
CHRNA6	8973
PROC	5624
ADAMTS14	140766
ASPM	259266
SGOL1	151648
CENPF	1063
BIRC5	332
SKA1	220134
DEPDC1B	55789
RDM1	201299
AURKB	9212
CENPA	1058
MKI67	4288
CDCA8	55143
TK1	7083
GTSE1	51512
NCAPG	64151

CDCA5	113130
RAD54L	8438
HIST2H3C	126961
GRM4	2914
CDCA3	83461
DEPDC1	55635
GAGE12D	100132399
HIST1H3H	8357
HCN2	610
HIST1H3J	8356
HOXC13	3229
SAMD14	201191
AIM1L	55057
SRMS	6725
CCDC64	92558
FABP6	2172
CSAG1	158511
LHX2	9355
CTHRC1	115908
FAM72D	728833
S100P	6286
HIST1H3D	8351
HOXC11	3227
VGF	7425
FAM64A	54478
CCDC19	25790
MCM10	55388
ZWINT	11130
PAFAH1B3	5050
CKS2	1164
HOXC10	3226
FAM72B	653820
HIST3H2BB	128312
CNTNAP2	26047
HAPLN1	1404
CENPM	79019
TUBB3	10381
MAPK8IP2	23542
SYNGR3	9143
ESPL1	9700
CTXN1	404217
NCAPH	23397
OIP5	11339
ERCC6L	54821
RAD51	5888
CCNA2	890
UBE2S	27338

KIF23	9493
RTBDN	83546
CCNB1	891

Supplementary Table 6b. 221 EEA genes in TNBC patients

GeneName	GeneID
KIF4A	24137
MYBL2	4605
PLK1	5347
CCNB2	9133
DLGAP5	9787
CENPA	1058
MELK	9833
CDC45	8318
FOXM1	2305
ANLN	54443
RRM2	6241
CDC20	991
PKMYT1	9088
TROAP	10024
HJURP	55355
KIF2C	11004
NDC80	10403
CEP55	55165
CDCA2	157313
PBK	55872
AURKB	9212
KIF20A	10112
SKA3	221150
SKA1	220134
SPC25	57405
NEIL3	55247
CDKN3	1033
DEPDC1B	55789
GTSE1	51512
BUB1	699
DEPDC1	55635
KIF18B	146909
CDC25C	995
RAD54L	8438
KIFC1	3833
HMMR	3161
PTTG1	9232
CDCA5	113130
ASF1B	55723
CDCA3	83461
CCNA2	890
SPC24	147841
TTK	7272
NUSAP1	51203
IBSP	3381

NCAPG	64151
CDCA8	55143
BIRC5	332
UHRF1	29128
CDK1	983
FAM83D	81610
SGOL1	151648
CKAP2L	150468
NCAPH	23397
COL10A1	1300
SHCBP1	79801
ERCC6L	54821
MKI67	4288
RAD51	5888
OIP5	11339
KIAA0101	9768
MCM10	55388
CCNE1	898
MND1	84057
CENPM	79019
CENPE	1062
KIF23	9493
FAM64A	54478
TACC3	10460
TK1	7083
TOP2A	7153
PIF1	80119
TDO2	6999
KIF15	56992
SLC7A5	8140
MMP11	4320
COL11A1	1301
CKS2	1164
VGF	7425
CCL11	6356
CDC6	990
SPAG5	10615
PPAPDC1A	196051
E2F2	1870
E2F1	1869
OR2B6	26212
CLSPN	63967
CDT1	81620
PRC1	9055
POLQ	10721
AIM1L	55057
ACTL8	81569

EZH2	2146
KIF11	3832
CCNB1	891
FANCA	2175
CBX2	84733
ESPL1	9700
HIST1H2BO	8348
UBE2S	27338
CNIH2	254263
TRIP13	9319
FAM72D	728833
HIST1H2AM	8336
TUBB3	10381
E2F8	79733
FAM111B	374393
LMNB1	4001
CA9	768
CASC5	57082
HIST2H3C	126961
SH2D2A	9047
LHX2	9355
MFAP2	4237
MMP1	4312
MMP13	4322
GPR19	2842
GPRIN1	114787
GJB2	2706
KIF18A	81930
IL4I1	259307
EPYC	1833
RAD51AP1	10635
BLM	641
NMU	10874
ESCO2	157570
CLEC5A	23601
E2F7	144455
PRAME	23532
ZIC1	7545
CBS	875
CXCL10	3627
TNFRSF9	3604
TYMS	7298
KIAA1199	57214
MKRN3	7681
CCL7	6354
HSD17B6	8630
HAPLN1	1404

S100P	6286
RAET1K	646024
HMGB3	3149
CDC25A	993
C16orf59	80178
HIST1H3B	8358
PITX1	5307
CENPI	2491
GINS1	9837
ZWINT	11130
WDR62	284403
ECE2	9718
STIL	6491
PAQR4	124222
KREMEN2	79412
B3GNT4	79369
A2ML1	144568
KCNK12	56660
ADAMDEC1	27299
TCAM1P	146771
SCT	6343
CTHRC1	115908
CST1	1469
ADAMTS14	140766
ENTHD1	150350
WBSCR28	135886
CST4	1472
CLEC6A	93978
COMP	1311
COL9A3	1299
LRRC15	131578
KIF26B	55083
CCR8	1237
RLTPR	146206
CHST6	4166
ETV7	51513
DIAPH3	81624
ULBP1	80329
EME1	146956
RDM1	201299
ARHGAP11A	9824
C2orf48	348738
KIF4B	285643
CDCA7	83879
PRR11	55771
MAD2L1	4085
LRP8	7804

GSG2	83903
TLX1	3195
CXCL11	6373
KPNA2	3838
TFR2	7036
PSRC1	84722
RCOR2	283248
TLX1NB	100038246
HN1	51155
TLX3	30012
CENPW	387103
CIT	11113
SOX11	6664
GAL	51083
OTX1	5013
MFSD2B	388931
STMN1	3925
HPDL	84842
DMRT1	1761
C18orf56	494514
CCDC150	284992
CDC7	8317
C1orf106	55765
KIAA1524	57650
HMGA1	3159
POC1A	25886
ZIC4	84107
STRA8	346673
SMC1B	27127
GBX2	2637
HIST2H2AA3	8337
SIX2	10736
H2AFX	3014

Supplementary Table 7a. Gene list in cluster 1

GeneName	GeneID
IBSP	3381
COL10A1	1300
MMP1	4312
MMP11	4320
PLK1	5347
COL11A1	1301
CEP55	55165
ANLN	54443
KIF4A	24137
CDC20	991
MYBL2	4605
MELK	9833
FOXM1	2305
MMP13	4322
PPAPDC1A	196051
DEPDC1B	55789
KIF2C	11004
SPC24	147841
CDCA2	157313
DLGAP5	9787
PKMYT1	9088
CDC45	8318
CCL11	6356
GJB2	2706
CKAP2L	150468
TDO2	6999
OR2B6	26212
SHCBP1	79801
HMMR	3161
ASF1B	55723
RRM2	6241
PBK	55872
EPYC	1833
BUB1	699
CDKN3	1033
IL4I1	259307
VGF	7425
UHRF1	29128
CLEC5A	23601
TNFRSF9	3604
CLSPN	63967
ADAMDEC1	27299
SCT	6343
TUBB3	10381
AIM1L	55057

ACTL8	81569
KIAA1199	57214
CASC5	57082
CTHRC1	115908
S100P	6286
MFAP2	4237
HSD17B6	8630
CST1	1469
LHX2	9355
ENTHD1	150350
ADAMTS14	140766
CST4	1472
HIST1H2AM	8336
CLEC6A	93978
CBS	875
ZIC1	7545
CXCL10	3627
CA9	768
HIST1H2BO	8348
CCR8	1237
LRRC15	131578
HAPLN1	1404
KIF26B	55083
CHST6	4166
COL9A3	1299
MKRN3	7681
COMP	1311
RLTPR	146206
PITX1	5307
CXCL11	6373
TFR2	7036
KREMEN2	79412
C1orf106	55765
B3GNT4	79369
STRA8	346673
GBX2	2637
HIST2H2AA3	8337
SIX2	10736

Supplementary Table 7b. Gene list in cluster 2

GeneName	GeneID
IBSP	3381
COL10A1	1300
MMP1	4312
MMP11	4320
PLK1	5347
COL11A1	1301
CEP55	55165
ANLN	54443
KIF4A	24137
CDC20	991
MYBL2	4605
MELK	9833
CCNB2	9133
HJURP	55355
CDCA3	83461
SKA1	220134
CENPA	1058
FOXM1	2305
MMP13	4322
NDC80	10403
PPAPDC1A	196051
TROAP	10024
DEPDC1B	55789
KIF2C	11004
SPC24	147841
CDCA2	157313
NUSAP1	51203
KIF20A	10112
SPC25	57405
DLGAP5	9787
MKI67	4288
PKMYT1	9088
CDC45	8318
TACC3	10460
CDCA8	55143
GTSE1	51512
PTTG1	9232
CCNA2	890
FAM83D	81610
CCL11	6356
CDCA5	113130
DEPDC1	55635
GPR19	2842
GJB2	2706
KIFC1	3833

SKA3	221150
CKAP2L	150468
OIP5	11339
TDO2	6999
SGOL1	151648
OR2B6	26212
NCAPH	23397
RAD54L	8438
AURKB	9212
CDC25C	995
KIF18B	146909
SHCBP1	79801
HMMR	3161
NEIL3	55247
ASF1B	55723
TTK	7272
RRM2	6241
PBK	55872
CDK1	983
EPYC	1833
CENPE	1062
PIF1	80119
NCAPG	64151
BUB1	699
CDKN3	1033
KIF15	56992
ERCC6L	54821
FAM64A	54478
IL4I1	259307
VGF	7425
KIF23	9493
BIRC5	332
TOP2A	7153
KIAA0101	9768
MND1	84057
UHRF1	29128
SLC7A5	8140
CENPM	79019
PRC1	9055
GPRIN1	114787
SPAG5	10615
RAD51	5888
CKS2	1164
CLEC5A	23601
CNIH2	254263
ADAMDEC1	27299
FAM111B	374393

CCNE1	898
FAM72D	728833
SCT	6343
MCM10	55388
EZH2	2146
E2F1	1869
TUBB3	10381
NMU	10874
TK1	7083
LMNB1	4001
AIM1L	55057
E2F8	79733
ACTL8	81569
FANCA	2175
TCAM1P	146771
E2F2	1870
CTHRC1	115908
S100P	6286
MFAP2	4237
RAD51AP1	10635
KIF11	3832
HSD17B6	8630
CST1	1469
KIF18A	81930
LHX2	9355
CDC6	990
HIST2H3C	126961
ESPL1	9700
ENTHD1	150350
ADAMTS14	140766
WBSCR28	135886
HIST1H2AM	8336
CDT1	81620
CBS	875
ZIC1	7545
CCNB1	891
UBE2S	27338
TRIP13	9319
CBX2	84733
CXCL10	3627
CA9	768
SH2D2A	9047
TYMS	7298
POLQ	10721
HIST1H2BO	8348
CCL7	6354
PRAME	23532

ETV7	51513
LRRC15	131578
HAPLN1	1404
KIF26B	55083
CHST6	4166
RAET1K	646024
COL9A3	1299
MKRN3	7681
COMP	1311
RLTPR	146206
C16orf59	80178
CDC25A	993
DIAPH3	81624
CENPI	2491
PITX1	5307
ULBP1	80329
HMGB3	3149
EME1	146956
HIST1H3B	8358
ARHGAP11A	9824
GINS1	9837
RDM1	201299
ZWINT	11130
KIF4B	285643
C2orf48	348738
WDR62	284403
ECE2	9718
CDCA7	83879
MAD2L1	4085
LRP8	7804
KPNA2	3838
CXCL11	6373
TLX1	3195
PSRC1	84722
TFR2	7036
HN1	51155
RCOR2	283248
TLX1NB	100038246
PAQR4	124222
CENPW	387103
CIT	11113
TLX3	30012
STMN1	3925
MFSD2B	388931
OTX1	5013
GAL	51083
SOX11	6664

KREMEN2	79412
HPDL	84842
CCDC150	284992
C18orf56	494514
DMRT1	1761
CDC7	8317
POC1A	25886
HMGA1	3159
C1orf106	55765
B3GNT4	79369
KCNK12	56660
A2ML1	144568
ZIC4	84107
STRA8	346673
GBX2	2637
SMC1B	27127
H2AFX	3014
HIST2H2AA3	8337
SIX2	10736

Supplementary Table 7c. Gene list in cluster 3

GeneName	GeneID
IBSP	3381
COL10A1	1300
MMP1	4312
MMP11	4320
PLK1	5347
COL11A1	1301
CEP55	55165
ANLN	54443
KIF4A	24137
CDC20	991
MYBL2	4605
MELK	9833
CCNB2	9133
HJURP	55355
CDCA3	83461
SKA1	220134
CENPA	1058
FOXM1	2305
MMP13	4322
NDC80	10403
PPAPDC1A	196051
TROAP	10024
DEPDC1B	55789
KIF2C	11004
SPC24	147841
CDCA2	157313
NUSAP1	51203
KIF20A	10112
SPC25	57405
DLGAP5	9787
MKI67	4288
PKMYT1	9088
CDC45	8318
TACC3	10460
CDCA8	55143
GTSE1	51512
PTTG1	9232
CCNA2	890
FAM83D	81610
CCL11	6356
CDCA5	113130
DEPDC1	55635
GPR19	2842
GJB2	2706
KIFC1	3833

SKA3	221150
CKAP2L	150468
OIP5	11339
TDO2	6999
SGOL1	151648
OR2B6	26212
NCAPH	23397
RAD54L	8438
AURKB	9212
CDC25C	995
KIF18B	146909
SHCBP1	79801
HMMR	3161
NEIL3	55247
ASF1B	55723
TTK	7272
RRM2	6241
PBK	55872
CDK1	983
EPYC	1833
CENPE	1062
PIF1	80119
NCAPG	64151
BUB1	699
CDKN3	1033
KIF15	56992
ERCC6L	54821
FAM64A	54478
IL4I1	259307
VGF	7425
KIF23	9493
BIRC5	332
TOP2A	7153
KIAA0101	9768
MND1	84057
UHRF1	29128
SLC7A5	8140
CENPM	79019
PRC1	9055
GPRIN1	114787
SPAG5	10615
RAD51	5888
CKS2	1164
CLEC5A	23601
TNFRSF9	3604
CNIH2	254263
CLSPN	63967

ADAMDEC1	27299
FAM111B	374393
CCNE1	898
FAM72D	728833
SCT	6343
MCM10	55388
EZH2	2146
E2F1	1869
TUBB3	10381
NMU	10874
TK1	7083
BLM	641
LMNB1	4001
AIM1L	55057
E2F8	79733
ACTL8	81569
FANCA	2175
TCAM1P	146771
E2F2	1870
KIAA1199	57214
CASC5	57082
ESCO2	157570
CTHRC1	115908
S100P	6286
MFAP2	4237
RAD51AP1	10635
KIF11	3832
HSD17B6	8630
CST1	1469
KIF18A	81930
LHX2	9355
CDC6	990
HIST2H3C	126961
ESPL1	9700
E2F7	144455
ENTHD1	150350
ADAMTS14	140766
CST4	1472
WBSCR28	135886
HIST1H2AM	8336
CDT1	81620
CLEC6A	93978
CBS	875
ZIC1	7545
CCNB1	891
UBE2S	27338
TRIP13	9319

CBX2	84733
CXCL10	3627
CA9	768
SH2D2A	9047
TYMS	7298
POLQ	10721
HIST1H2BO	8348
CCL7	6354
CCR8	1237
PRAME	23532
ETV7	51513
LRRC15	131578
HAPLN1	1404
KIF26B	55083
CHST6	4166
RAET1K	646024
COL9A3	1299
MKRN3	7681
COMP	1311
RLTPR	146206
C16orf59	80178
CDC25A	993
DIAPH3	81624
CENPI	2491
PITX1	5307
ULBP1	80329
HMGB3	3149
HIST1H3B	8358
ARHGAP11A	9824
GINS1	9837
RDM1	201299
ZWINT	11130
KIF4B	285643
C2orf48	348738
WDR62	284403
ECE2	9718
STIL	6491
MAD2L1	4085
LRP8	7804
PRR11	55771
GSG2	83903
KPNA2	3838
CXCL11	6373
TLX1	3195
PSRC1	84722
TFR2	7036
RCOR2	283248

TLX1NB	100038246
PAQR4	124222
CENPW	387103
CIT	11113
MFSD2B	388931
OTX1	5013
GAL	51083
SOX11	6664
KREMEN2	79412
HPDL	84842
CCDC150	284992
DMRT1	1761
CDC7	8317
HMGA1	3159
KIAA1524	57650
C1orf106	55765
B3GNT4	79369
KCNK12	56660
A2ML1	144568
ZIC4	84107
STRA8	346673
GBX2	2637
SMC1B	27127
HIST2H2AA3	8337
SIX2	10736

Supplementary Table 7d. Gene list in cluster 4

GeneName	GeneID
IBSP	3381
COL10A1	1300
MMP1	4312
MMP11	4320
PLK1	5347
COL11A1	1301
CEP55	55165
ANLN	54443
KIF4A	24137
CDC20	991
MYBL2	4605
MELK	9833
CCNB2	9133
HJURP	55355
CDCA3	83461
SKA1	220134
CENPA	1058
FOXM1	2305
MMP13	4322
NDC80	10403
PPAPDC1A	196051
TROAP	10024
DEPDC1B	55789
KIF2C	11004
SPC24	147841
CDCA2	157313
NUSAP1	51203
KIF20A	10112
SPC25	57405
DLGAP5	9787
MKI67	4288
PKMYT1	9088
CDC45	8318
TACC3	10460
CDCA8	55143
GTSE1	51512
PTTG1	9232
CCNA2	890
FAM83D	81610
CCL11	6356
CDCA5	113130
DEPDC1	55635
GPR19	2842
GJB2	2706
KIFC1	3833

SKA3	221150
CKAP2L	150468
OIP5	11339
TDO2	6999
SGOL1	151648
OR2B6	26212
NCAPH	23397
RAD54L	8438
AURKB	9212
CDC25C	995
KIF18B	146909
SHCBP1	79801
HMMR	3161
NEIL3	55247
ASF1B	55723
TTK	7272
RRM2	6241
PBK	55872
CDK1	983
EPYC	1833
CENPE	1062
PIF1	80119
NCAPG	64151
BUB1	699
CDKN3	1033
KIF15	56992
ERCC6L	54821
FAM64A	54478
IL4I1	259307
VGF	7425
KIF23	9493
BIRC5	332
TOP2A	7153
KIAA0101	9768
MND1	84057
UHRF1	29128
SLC7A5	8140
CENPM	79019
PRC1	9055
GPRIN1	114787
SPAG5	10615
RAD51	5888
CKS2	1164
CLEC5A	23601
TNFRSF9	3604
CNIH2	254263
CLSPN	63967

ADAMDEC1	27299
FAM111B	374393
CCNE1	898
FAM72D	728833
SCT	6343
MCM10	55388
EZH2	2146
E2F1	1869
TUBB3	10381
NMU	10874
TK1	7083
BLM	641
LMNB1	4001
AIM1L	55057
E2F8	79733
ACTL8	81569
FANCA	2175
TCAM1P	146771
E2F2	1870
KIAA1199	57214
CASC5	57082
ESCO2	157570
CTHRC1	115908
S100P	6286
MFAP2	4237
RAD51AP1	10635
KIF11	3832
HSD17B6	8630
CST1	1469
KIF18A	81930
LHX2	9355
CDC6	990
HIST2H3C	126961
ESPL1	9700
E2F7	144455
ENTHD1	150350
ADAMTS14	140766
CST4	1472
WBSCR28	135886
HIST1H2AM	8336
CDT1	81620
CLEC6A	93978
CBS	875
ZIC1	7545
CCNB1	891
UBE2S	27338
TRIP13	9319

CBX2	84733
CXCL10	3627
CA9	768
SH2D2A	9047
TYMS	7298
POLQ	10721
HIST1H2BO	8348
CCL7	6354
CCR8	1237
PRAME	23532
ETV7	51513
LRRC15	131578
HAPLN1	1404
KIF26B	55083
CHST6	4166
RAET1K	646024
COL9A3	1299
MKRN3	7681
COMP	1311
RLTPR	146206
C16orf59	80178
CDC25A	993
DIAPH3	81624
CENPI	2491
PITX1	5307
ULBP1	80329
HMGB3	3149
EME1	146956
HIST1H3B	8358
ARHGAP11A	9824
GINS1	9837
RDM1	201299
ZWINT	11130
KIF4B	285643
C2orf48	348738
WDR62	284403
ECE2	9718
STIL	6491
CDCA7	83879
MAD2L1	4085
LRP8	7804
PRR11	55771
GSG2	83903
KPNA2	3838
CXCL11	6373
TLX1	3195
PSRC1	84722

TFR2	7036
HN1	51155
RCOR2	283248
TLX1NB	100038246
PAQR4	124222
CENPW	387103
CIT	11113
TLX3	30012
STMN1	3925
MFSD2B	388931
OTX1	5013
GAL	51083
SOX11	6664
KREMEN2	79412
HPDL	84842
CCDC150	284992
C18orf56	494514
DMRT1	1761
CDC7	8317
POC1A	25886
HMGA1	3159
KIAA1524	57650
C1orf106	55765
B3GNT4	79369
KCNK12	56660
A2ML1	144568
ZIC4	84107
STRA8	346673
GBX2	2637
SMC1B	27127
H2AFX	3014
HIST2H2AA3	8337
SIX2	10736

Supplementary Table 8a. Analyses of cluster 1 genes and their association with CIN or TME

Section	Cluster 1 Gene	Entrez Gene ID	Process	Hypoxia Response?	PubmedID	Summary
TME	HMMR	3161	Spindle Formation, Migration	Yes	12808028, 8634437	RHAMM localizes to the centrosome and functions in the maintenance of spindle integrity. Interacts with Hyaluronan a glycosaminoglycan present in the ECM
TME	CA9	768	Metabolism, Hypoxia	Yes	24146383	Hypoxia-induced upregulation is a component of the complex response of cancer cells to microenvironment
TME	CEMIP	57214	Migration, Hypoxia	Yes	28819426	Overexpression protects tumour from microenvironment in hypoxia by stimulating the Wnt, EGFR, FGFR pathway, role in cytokine pathway
TME	CTHRC1	115908	Extracellular Matrix, Angiogenesis, Hypoxia	Yes	16778098, 27686285, 25263696	Involved in angiogenesis and Wnt signalling, activates hypoxia-inducible factor 1α (HIF-1α) and vascular endothelial growth factor (VEGF)
TME	CXCL11	6373	Immune Response	Yes	24940911	Chemokine expression in the tumor microenvironment regulates different processes
TME	MMP1	4312	Extracellular Matrix, Hypoxia	Yes	26343184	Hypoxia-induced expression accompanied by stabilization of hypoxia-inducible transcription factors (HIF)-1α and HIF-2α, and a rise in intracellular ROS
TME	TNFRSF9	3604	Lymphocyte Activation	Yes	8262389	Member of the human nerve-growth-factor/tumor-necrosis-factor receptor family
TME	COL11A1	1301	Extracellular Matrix	Yes	10486316	Collagen protein in extracellular matrix
TME	DEPDC1B	55789	Adhesion		25458010	Coordinates de-adhesion events and cell-cycle progression at mitosis
TME	S100P	6286	DNA Damage Response		26967060, 25614008, 19187537	Binds and inactivates p53. Present in microenvironment
TME	ADAMDEC1	27299	Immune Response		12037602	Secreted metalloprotease involved in immune response
TME	ADAMTS14	140766	Extracellular Matrix		25636539	Secreted protein with important role in extracellular matrix (ECM) assembly and degradation

TME	CARMIL2	146206	Migration		19846667, 26466680	Regulates actin assembly and actin-based cell motility and localizes to vimentin
TME	CBS	875	Angiogenesis, Metabolism, Hypoxia		24730679, 24328859	Production of hydrogen sulfide has important role in cellular bioenergetics, migration, and angiogenesis, and hypoxia-inducible factors regulate expression
TME	CCL11	6356	Migration, Inflammation		19351767	Cancer-related inflammation and migration can be directly regulated by chemokines
TME	CCR8	1237	Migration, Inflammation		23363815	Cancer-related inflammation and migration can be directly regulated by chemokines
TME	CLEC5A	23601	Immune Response		12570670, 10449773	Binds DAP12, involved in immune response
TME	CLEC6A	93978	Immune Response		16423983	C-type lectin-like receptor carbohydrate recognition by immune cells
TME	COL10A1	1300	Extracellular Matrix		22894674	Colleagen protein expressed in micronenvironment
TME	CXCL10	3627	Immune Response, Hypoxia		28592115, 24799675	Present in tumor environment, may have role in signalling under hypoxic conditions
TME	HAPLN1	1404	Extracellular Matrix		22159717	Extracellular matrix component secreted by tumour cells
TME	IL4I1	259307	Metabolism, Immune Response		28405502, 19436310	Limits the CD8+ T cell mediated immune response
TME	KIF26B	55083	Adhesion, Migration		28581513, 25652119	Regulation of adhesion in mesenchymal cell, and inhibits VEGF signaling pathway to promote metastasis
TME	LHX2	9355	Transcription		24423492	Involved in autocrine PDGF-B/PDGFRβ siganlling loop required for cell migration, invasion and metastatic dissemination and paracrine PDGF-B/PDGFRβ signaling to support blood vessel functionality
TME	MMP11	4320	Extracellular Matrix		26892540	Matrix metalloproteinase family have capacity to remodel extracellular matrix

TME	MMP13	4322	Extracellular Matrix		22992737, 20371345	Matrix metalloproteinase family have capacity to remodel extracellular matrix, can promote angiogenesis
TME	TDO2	6999	Metabolism, Immune Response		25628622	Key regulator of innate and adaptive immunity plays a role in cancer-induced immunosuppression
TME	PITX1	5307	Transcription, Hypoxia		25558831	A transcription factor that helps promote specificity in HIF-1α dependent target gene activation
TME	TFR2	7036	Angiogenesis, Hypoxia		20360937	In vitro hypoxic conditions induce a significant TfR2 up-regulation suggesting a role in tumor angiogenesis
TME	COL9A3	1299	Extracellular Matrix		8660302	Collagen protein in extracellular matrix
TME	COMP	1311	Extracellular Matrix		16051604	Cartilage oligomeric matrix protein extracellular matrix component
TME	CST1	1469	Protease Inhibitors		UniProt	Cysteine-type endopeptidase inhibitor activity, same family as CST4
TME	CST4	1472	Protease Inhibitors		3488317	Cysteine-type endopeptidase inhibitor activity
TME	EPYC	1833	Extracellular Matrix		11292372	Cartilage proteoglycan in extracellular matrix
TME	LRRC15	131578	Extracellular Matrix		16098915	May contribute to regulation of cell-matrix adhesion interactions in astrocytes
TME	MFAP2	4237	Extracellular Matrix		26167404	Component of extracellular microfibrils
TME-R	GJB2	2706	Cell-cell Communicatio n		22514560	Cell adhesion and gap junction associated
TME-R	IBSP	3381	Extracellular Matrix		24103036	Extracellular matrix component that can bind to integrin
TME-R	SCT	6343	Endocrine Signalling		11060443	Endocrine hormone with possible neuroendocrine function
TME-R	VGF	7425	Endocrine Signalling		19194657	VGF is a precursor of multiple bioactive peptides with diverse neuroendocrine functions
MISC	B3GNT4	79369	Glycolsylation	Yes		

MISC	ACTL8	81569	NA			
MISC	CHST6	4166	Glycosaminoglycan metabolism			
MISC	CRYBG2	55057	NA			
MISC	ENTHD1	150350	NA			
MISC	GBX2	2637	Transcription			
MISC	HSD17B	8630	Metabolism			
MISC	INAVA	55765	NA			
MISC	MKRN3	7681	Ubiquitinaton			
MISC	OR2B6	26212	Olfactory Receptor			
MISC	PLPP4	196051	Metabolism			
MISC	SIX2	10736	Transcription			
MISC	ZIC1	7545	Transcription			
CIN-R	HIST1H2	8336	Chromatin	Yes	Uniprot	Histone protein involved in nucleosome remodelling
CIN-R	HIST2H2	8337	Chromatin	Yes	Uniprot	Histone protein involved in nucleosome remodelling
CIN-R	HIST1H2	8348	Chromatin		Uniprot	Histone protein involved in nucleosome remodelling
CIN-R	KREMEN2	79412	Signalling		12050670	Dickkopf receptors that regulate Wnt/beta-catenin signalling
CIN-R	MYBL2	4605	Transcription		9840932, 23842645	Expression is controlled by the DREAM multiprotein complex, which is crucial in coordinating cell cycle dependent gene expression
CIN-R	STRA8	346673	Meiosis		18799751	Regulates meiotic initiation in both spermatogenesis and oogenesis
CIN	ASF1B	55723	Chromatin	Yes	11897662	Histone chaperones involved in replication-dependent nucleosome assembly
CIN	CDC20	991	Spindle Assembly Checkpoint	Yes	19528295, 15525512	Loss of spindle assembly checkpoint-mediated inhibition of Cdc20 promotes tumorigenesis

CIN	ANLN	54443	Cytokinesis		16040610, 20732437	Actin-binding protein localized at the cleavage furrow required for cytokinesis
CIN	BUB1	699	Spindle Assembly Checkpoint		21646403, 19487456, 16760428	Kinetochore-bound protein kinase is essential for spindle checkpoint signaling and correct chromosome alignment
CIN	CDC45	8318	DNA Replication		23643534	Helicase essential for DNA replication, critical regulator of Myc-driven DNA replication stress
CIN	CDCA2	157313	Chromatin		21820363, 16998479	Coordinates chromosomal reorganization with nuclear envelope reassembly during mitotic exit
CIN	CDKN3	1033	Cell Cycle		23775190	Spindle checkpoint phosphatase essential for normal mitosis and G1/S transition
CIN	CEP55	55165	Cytokinesis		16198290, 16406728, 25915844	A direct transcriptional target of FOXM1, and PLK1 phosphorylation of CEP55 regulates CEP55 recruitment to the midbody
CIN	CKAP2L	150468	Cell Cycle		24260314, 17376772	Mitotic spindle protein that functions in cell division of neural progenitors, degraded by APC/C-Cdh1 during mitotic exit
CIN	CLSPN	63967	DNA Replication		12766152, 11090622	Inhibits Chk1 and ATR, downregulation of Claspin increases premature chromatin condensation
CIN	DLGAP5	9787	Spindle Formation		17118403, 15987997	Microtubule-associated protein required for correct spindle formation
CIN	FOXM1	2305	Transcription, Hypoxia		23768511, 19097132	FOXM1 for the maintenance of genomic stability and chromosomal integrity and regulates the expression of several cell cycle genes. Binding of hypoxia-inducible factor 1 (HIF-1) to the HIF-1 binding sites in the FoxM1 promoter
CIN	KIF4A	24137	Cytokinesis, Spindle Organization		15625105, 15326200, 15297875	Functions in chromosome condensation and segregation
CIN	KNL1	57082	Kinetochore		24344188	Required for Aurora B activity at kinetochores and kinetochore-microtubule attachment
CIN	MELK	9833	Mitotic Progression		15908796, 24844244	Oncogenic kinase essential for mitotic progression in basal-like breast cancer cells

CIN	PBK	55872	DNA Damage Response		17482142	A mitotic kinase that downregulates p53 by direct physical interaction
CIN	PKMYT1	9088	Mitotic Progression		12738781, 10504341, 7569953	Plk1 substrate that prevents catastrophic premature entry into mitosis
CIN	PLK1	5347	Spindle Assembly Checkpoint		18615013, 17376779, 16837776	Functions include the regulation of centrosome maturation and spindle assembly, the removal of cohesins from chromosome arms, inactivation of anaphase-promoting complex/cyclosome inhibitors, and the regulation of mitotic exit and cytokinesis
CIN	RRM2	6241	DNA Damage Repair		20159953	Ribonucleotide reductase rapidly accumulates at double-strand break sites
CIN	SHCBP1	79801	Cytokinesis		27129942, 25486361	Interacts with centralspindlin and is required for completion of cytokinesis
CIN	SPC24	147841	Kinetochore		14699129, 15961401, 27713128	Kinetochore component for the stable kinetochore-microtubule interactions required for chromosome segregation
CIN	UHRF1	29128	DNA Methylation		24486181	DNA hypomethylation contributes to oncogenesis through multiple mechanisms, including chromosomal instability
CIN	TUBB3	10381	Cytoskeleton, Hypoxia		23321512, 18178340	Contributes to Microtubule dynamics. TUBB3 increase upon hypoxia in A2780 cells
CIN	KIF2C	11004	Chromosome Segregation		23098759, 19060894, 14960279	Microtubule-dependent molecular motor protein with critical role in chromosome movement and segregation. Antigen peptide presentation via MHC class II

Supplementary Table 8b. Upstream regulators of cluster 1 genes and their association with CIN or TME

Complex	Upstream	Entrez	Interacts with CIN/TME/Both	Hypoxia
	AJUBA	84962		
	ATF3	467		
	BARX2	8538		
	BRCA1	672		
	BUB1B	701		
	BUB3	9184		
	CASP7	840		
	CCNB1	891		
	CDC27	996		
	CDC7	8317		
	CDK1	983		
	CDK2	1017		
	CELF1	10658		
	CKS1B	1163		
	DRAP1	10589		
	DVL2	1856		
	FBXO5	26271		
	FBXW5	54461		
	FZR1	51343		
	HDAC1	3065		
	HERC2	8924		
	LATS1	9113		
	LIN28A	79727		
	MAD1L1	8379		
	MAD2L1	4085		
	MAD2L1BP	9587		
	MAP2K1	5604		
	MBD2	8932		
	MIIP	60672		
	MIS12	79003		
	MSH6	2956		
	MXI1	4601		
	NCAPG	64151		
	NDC80	10403		
	NEDD1	121441		
	NINL	22981		
	NOTCH3	4854		
	NPM1	4869		
	NUF2	83540		
	NUPR1	26471		
	ORC2	4999		
	OTUB1	55611		
	PAK1	5058		
	PARP1	142		

	PMS2	5395		
	POLR2A	5430		
	PPP1CC	5501		
	PRC1	9055		
	PSMA1	5682		
	PSMA2	5683		
	PSMA3	5684		
	PSMA4	5685		
	PSMA5	5686		
	PSMA6	5687		
	PSMA7	5688		
	PSMB1	5689		
	PSMB3	5691		
	PSMB4	5692		
	PSMC2	5701		
	PTEN	5728		
	RAE1	8480		
	RARA	5914		
	RASSF1	11186		
	REST	5978		
	RRM1	6240		
	RRM2B	50484		
	RUNX1	861		
	SATB1	6304		
	SEPT9	10801		
	SKP2	6502		
	SMAD2	4087		
	SMAD4	4089		
	SMAD7	4092		
	SMARCA2	6595		
	SMC2	10592		
	SOX11	6664		
	SPAG9	9043		
	SRSF1	6426		
	STEAP3	55240		
	STK10	6793		
	SUMO1	7341		
	SUZ12	23512		
	TAL1	6886		
	TERF1	7013		
	TERT	7015		
	TOPBP1	11073		
	TTK	7272		
	UBE2S	27338		
	UHRF1BP1	54887		
	USP28	57646		
	USP9X	8239		

	WDHD1	11169		
	WEE1	7465		
	YAP1	10413		
	YY1	7528		
	ZBED6	1E+08		
	ZNF24	7572		
	ZNF622	90441		
	ZWINT	11130		
	ARNT	405		
	BACH1	571		
	BCL6	604		
	BHLHE41	79365		
	BMPR1A	657		
	CDKN2A	1029		
	CEBPB	1051		
	CITED2	10370		
	CLTC	1213		
	CTNNB1	1499		
	CXCR3	2833		
	DNAJB6	10049		
	ELF3	1999		
	ELK1	2002		
	EPAS1	2034		
	ERG	2078		
	ETS1	2113		
	ETS2	2114		
	FOSL1	8061		
	FOXA1	3169		
	FZD6	8323		
	GATA2	2624		
	GLI1	2735		
	GLI2	2736		
	GRN	2896		
	HDAC3	8841		
	HEXIM1	10614		
	HIF1A	3091		
	HNRNPK	3190		
	IFI16	3428		
	IRF1	3659		
	IRF3	3661		
	IRF7	3665		
	IRF9	10379		
	JUNB	3726		
	JUND	3727		
	KEAP1	9817		
	LANCL1	10314		
	LONP1	9361		

	NANOG	79923		
	NF1	4763		
	NFE2L2	4780		
	NFYA	4800		
	NFYB	4801		
	NR3C1	2908		
	OCLN	1.01E+08		
	PGR	5241		
	PIAS3	10401		
	POU5F1	5460		
	PRDM5	11107		
	PRMT5	10419		
	RBPJ	3516		
	RORC	6097		
	RUNX2	860		
	S100A4	6275		
	S100PBP	64766		
	SMARCA4	6597		
	SMARCD3	6604		
	SOX2	6657		
	SP100	6672		
	SP3	6670		
	SRA1	10011		
	SREBF1	6720		
	STAT1	6772		
	STAT2	6773		
	STAT6	6778		
	T	6862		
	TCF12	6938		
	TCF7L2	6934		
	TFRC	7037		
	THRB	7068		
	TNFSF9	8744		
	TPX2	22974		
	TWIST1	7291		
	TYROBP	7305		
	USF1	7391		
	WNT5A	7474		
	YWHAZ	7534		
	ZNF217	7764		
	CBX5	23468		
	CCND1	595		
	ESR1	2099		
	ESR2	2100		
	EZH2	2146		
	FOXO3	2309		
	MYC	4609		

	SMAD3	4088		
	STAT3	6774		
	STAT5A	6776		
	TP53	7157		
	TP63	8626		
	WT1	7490		
	ADAMTS7	11173		
	AKAP12	9590		
	ANAPC2	29882		
	ANAPC7	51434		
	APC	324		
	AR	367		
	ARX	170302		
	ATF6	22926		
	ATRX	546		
	AURKA	6790		
	AURKB	9212		
	BARD1	580		
	BCL2	596		
	BCL2L1	598		
	BCL2L14	79370		
	BIRC5	332		
	BRCA2	675		
	CCL1	6346		
	CCNA1	8900		
	CCNA2	890		
	CD44	960		
	CDC14A	8556		
	CDC25B	994		
	CDC25C	995		
	CEP170	9859		
	CHEK1	1111		
	CHUK	1147		
	CUEDC2	79004		
	CUL1	8454		
	CYLD	1540		
	DAXX	1616		
	DSN1	79980		
	DUSP1	1843		
	EGFR	1956		
	EIF4B	1975		
	EPOR	2057		
	ERCC6L	54821		
	ETV1	2115		
	FBXL14	144699		
	FOXO1	2308		
	HAT1	8520		

	HAUS8	93323		
	HDAC2	3066		
	HFE	3077		
	HNF4A	3172		
	HSPA8	3312		
	IPO4	79711		
	IRF4	3662		
	ITGA2	3673		
	ITGB1	3688		
	KAT5	10524		
	KDM5B	10765		
	KIF23	9493		
	KIFC1	3833		
	KLF4	9314		
	KMT2E	55904		
	LIN37	55957		
	LIN9	286826		
	LRRK2	120892		
	MAPK10	5602		
	MAPK8	5599		
	MAVS	57506		
	MCM7	4176		
	MDM2	4193		
	MITF	4286		
	MMP25	64386		
	NASP	4678		
	NEUROG1	4762		
	NFKBIA	4792		
	ODF2	4957		
	PIN1	5300		
	PLEKHG6	55200		
	PRKDC	5591		
	RAF1	5894		
	RBBP4	5928		
	SIN3B	23309		
	SP1	6667		
	SPC25	57405		
	TIAL1	7073		
	XRCC5	7520		
	XRCC6	2547		
Ap1 complex	FOS	2353		
Ap1 complex	JUN	3725		
Cpla2 complex	PLA2G4A	5321		
Cpla2 complex	JMJD7-PLA2G4B	8681		

Cpla2 complex	PLA2G10	8399		
Cpla2 complex	PLA2G12A	81579		
Cpla2 complex	PLA2G12B	84647		
Cpla2 complex	PLA2G1B	5319		
Cpla2 complex	PLA2G2A	5320		
Cpla2 complex	PLA2G2D	26279		
Cpla2 complex	PLA2G2E	30814		
Cpla2 complex	PLA2G2F	64600		
Cpla2 complex	PLA2G3	50487		
Cpla2 complex	PLA2G4B	1E+08		
Cpla2 complex	PLA2G4C	8605		
Cpla2 complex	PLA2G4D	283748		
Cpla2 complex	PLA2G4E	123745		
Cpla2 complex	PLA2G4F	255189		
Cpla2 complex	PLA2G5	5322		
Cpla2 complex	PLA2G6	8398		
Creb complex	ATF2	1386		
Creb complex	ATF4	468		
Creb complex	CREB3	10488		
Creb complex	CREB3L3	84699		
Creb complex	CREB3L4	148327		
Creb complex	CREB5	9586		
Creb complex	CREB1	1385		
Creb complex	CREBBP	1387		
Creb complex	EP300	2033		
Cyclin E	CCNE1	898		
Cyclin E	CCNE2	9134		
E2f	E2F1	1869		
E2f	E2F2	1870		
E2f	E2F3	1871		
E2f	E2F4	1874		

E2f	E2F5	1875		
E2f	E2F6	1876		
E2f	E2F7	144455		
E2f	E2F8	79733		
ERK1/2	MAPK1	5594		
ERK1/2	MAPK3	5595		
Histone h3	H3F3A	3020		
Histone h3	H3F3B	3021		
Histone h3	HIST1H3C	8352		
Histone h3	HIST2H3C	126961		
Histone h3	HIST3H3	8290		
Histone h4	HIST1H4J	8363		
Hsp90	HSP90AA1	3320		
Hsp90	HSP90B1	7184		
Hsp90	HSP90AB1	3326		
N-cor	NCOR2	9612		
N-cor	NCOR1	9611		
NFkB (complex)	NFKB1	4790		
NFkB (complex)	RELA	5970		
NFkB (complex)	NFKB2	4791		
NFkB (complex)	REL	5966		
NFkB (complex)	RELB	5971		
Rb	RBL1	5933		
Rb	RBL2	5934		
Rb	RB1	5925		
Smad1/5/8	SMAD1	4086		
Smad1/5/8	SMAD5	4090		
Smad1/5/8	SMAD9	4093		
Top2	PTPMT1	114971		
Top2	TOP2A	7153		
Top2	TOP2B	7155		

Supplementary Table 8c. Causal network genes associated with cluster 1 genes along with their association with CIN or TME and hypoxia responsiveness

Complex	Upstream causal gene	Entrez Gene ID	Interacts with CIN/TME/Both	Hypoxia Response
	CD24	100133941		
	CDKN1A	1026		
	DDR2	4921		
	ETV1	2115		
	IRF4	3662		
	KDM5B	10765		
	LILRB4	11006		
	NAMPT	10135		
	PTGS2	5743		
	RAC1	5879		
	RUNX2	860		
	SUMO3	6612		
	ACVR1	90		
	ARX	170302		
	DPAGT1	1798		
	FAS	355		
	GBP1	2633		
	GPX4	2879		
	HFE	3077		
	HIF1AN	55662		
	HNF4A	3172		
	IFNL4	101180976		
	IL11	3589		
	IL18	3606		
	LAMA3	3909		
	RARRES3	5920		
	S100A9	6280		
	SAFB	6294		
	SDR9C7	121214		
	TBK1	29110		
	TIAL1	7073		
	TICAM1	148022		
	ATF6	22926		
	ERBB2	2064		
	MITF	4286		
	SP1	6667		
	TRAF2	7186		
	BSG	682		
	FOXO1	2308		
	HDAC2	3066		
	RABL6	55684		
	RAF1	5894		
	E2F6	1876		

	MDM2	4193		
	OGT	8473		
	SEL1L	6400		
	AR	367		
	AREG	374		
	BTRC	8945		
	DVL2	1856		
	FOXM1			
	NELFE	7936		
	NUPR1	26471		
	PPP2CB	5516		
	REST	5978		
	SVIL	6840		
E2f complex	E2F3	1871		
E2f complex	E2F4	1874		
E2f complex	E2F1	1869		
E2f complex	E2F2	1870		
E2f complex	E2F5	1875		
E2f complex	E2F7	144455		
E2f complex	E2F8	79733		
IL1 complex	IL1A	3552		
IL1 complex	IL1RN	3557		
IL1 complex	IL33	90865		
IL1 complex	IL36G	56300		
IL1 complex	IL1B	3553		
IL1 complex	IL1F10	84639		
IL1 complex	IL36A	27179		
IL1 complex	IL36B	27177		
IL1 complex	IL36RN	26525		
IL1 complex	IL37	27178		
Jnk complex	MAP2K4	6416		
Jnk complex	MAPK10	5602		
Jnk complex	MAPK12	6300		
Jnk complex	MAPK8	5599		
Jnk complex	MAPK9	5601		
NFkB complex	NFKB1	4790		
NFkB complex	NFKB2	4791		
NFkB complex	REL	5966		
NFkB complex	RELA	5970		
NFkB complex	RELB	5971		
PLC complex	PLCB4	5332		
PLC complex	PLCE1	51196		
PLC complex	PLCL1	5334		
PLC complex	PLCL2	23228		
PLC complex	NOTUM	147111		
PLC complex	PDIA3	2923		
PLC complex	PLCB1	23236		

PLC complex	PLCB2	5330		
PLC complex	PLCB3	5331		
PLC complex	PLCD1	5333		
PLC complex	PLCD3	113026		
PLC complex	PLCD4	84812		
PLC complex	PLCG1	5335		
PLC complex	PLCG2	5336		
PLC complex	PLCH1	23007		
PLC complex	PLCH2	9651		
PLC complex	PLCZ1	89869		
Ras complex	KRAS	3845		
Ras complex	RRAS2	22800		
Ras complex	HRAS	3265		
Ras complex	MRAS	22808		
Ras complex	NRAS	4893		
Ras complex	RRAS	6237		
SAA complex	SAA1	6288		
SAA complex	SAA2	6289		
SAA complex	SAA3	6290		
SAA complex	SAA4	6291		

Supplementary Table 9. The number of gene in each subtype and stage.

Stage/Subtype	ER+	TNBC	HER2 amplified	Overall
Stage 1	143	29	2	174
Stage 2	459	104	25	588
Stage 3	201	25	11	237
Stage 4	16	2	1	19
Total number	819	160	39	1018

SUPPLEMENTARY TABLE LEGENDS:

Supplementary Table 1: Up/down-regulated genes across all stages in overall patients, TNBC patients and ER+ patients, respectively. Table 1a, and 1b show up/down -regulated genes in different tumor stages for overall and TNBC cases, respectively.

Supplementary Table 2: Stage-specific up/down regulated genes in at least 70% of TNBC patients. Table 2a and 2b show up and down regulated genes in at least 70% of TNBC patients in each stage, respectively, as well as the fold change, frequency and FDR information.

Supplementary Table 3: TNBC stage-specific differentially expressed genes in at least 70% of overall patients. Table 3a and 3b show up and down regulated genes in at least 70% of overall patients in each stage, respectively, as well as the fold change, frequency and FDR information.

Supplementary Table 4: Identified amplified regions. Table 4 shows the identified chromosomal amplification regions.

Supplementary Table 5: Evaluations on the concordances between gene expression and copy number amplification for up-regulated genes on amplified regions. Table 5a and 5b show Pearson's correlation coefficient between gene expression and CNA, and fold CNA-associated change with FDR, and cytoband for up-regulated genes in overall and TNBC patients, respectively.

Supplementary Table 6: EEA genes in overall and TNBC patients. Table 6a and 6b show EEA genes in overall and TNBC patients, respectively.

Supplementary Table 7: Gene lists in four clusters. Table 7a, 7b, 7c and 7d show genes in cluster 1, 2, 3 and 4, respectively.

Supplementary Table 8: Analyses of cluster 1 genes and its interactions. Table 8a lists the cluster 1 genes and their association with CIN or TME. Table 8b and 8c enlist the upstream regulator and causal network genes respectively, along with their association with CIN or TME and hypoxia responsiveness.

Supplementary Table 9: The number of genes in each tumor stage and subtype. Table 9 shows the number of ER+, HER2+, TNBC, and overall patients in different stages.
